# Aluminum Amidinates: Insights into Alkyne Hydroboration

**DOI:** 10.1021/acs.inorgchem.1c00619

**Published:** 2021-07-16

**Authors:** Katie Hobson, Claire J. Carmalt, Clare Bakewell

**Affiliations:** Department of Chemistry, University College London, 20 Gordon Street, London WC1H 0AJ, United Kingdom

## Abstract

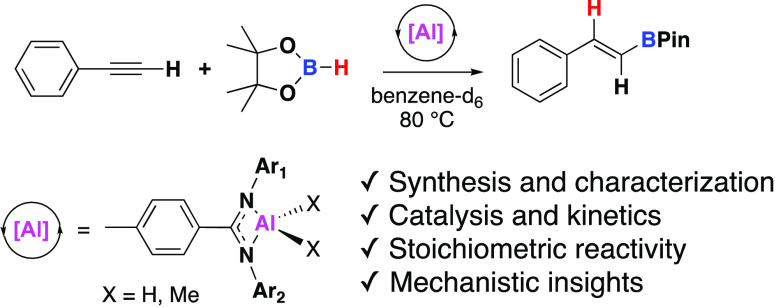

The mechanism of the aluminum-mediated hydroboration of terminal alkynes was investigated using a series of novel aluminum amidinate hydride and alkyl complexes bearing symmetric and asymmetric ligands. The new aluminum complexes were fully characterized and found to facilitate the formation of the (*E*)-vinylboronate hydroboration product, with rates and orders of reaction linked to complex size and stability. Kinetic analysis and stoichiometric reactions were used to elucidate the mechanism, which we propose to proceed via the initial formation of an Al-borane adduct. Additionally, the most unstable complex was found to promote decomposition of the pinacolborane substrate to borane (BH_3_), which can then proceed to catalyze the reaction. This mechanism is in contrast to previously reported aluminum hydride-catalyzed hydroboration reactions, which are proposed to proceed via the initial formation of an aluminum acetylide, or by hydroalumination to form a vinylboronate ester as the first step in the catalytic cycle.

## Abstract 2

This work focuses on the synthesis and characterization of a series of aluminum amidinate hydride and alkyl complexes. The compounds have been studied as hydroboration catalysts for the reaction of HBpin and phenylacetylene, and detailed kinetic and mechanistic investigations were carried out to elucidate possible reaction mechanisms. We propose the reaction to proceed via a HBpin adduct, in contrast to related literature on aluminum-catalyzed hydroboration reactions.

## Introduction

**Figure 1:**
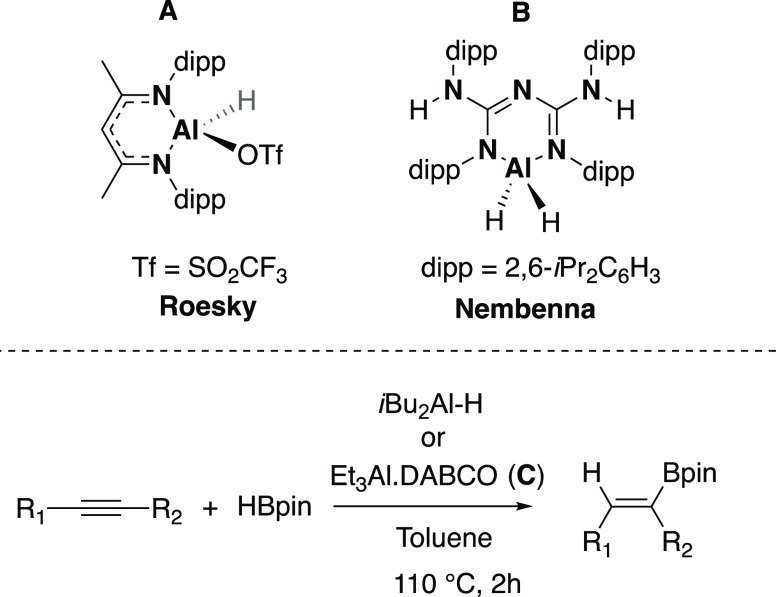
Examples of catalysts or precatalysts for hydroboration reactions.

The application of main-group metals in catalysis has flourished over recent years, largely driven by the need to alleviate global demand on conventional precious metal systems and find more sustainable alternatives.^1^ Main-group compounds have been widely shown to mimic transition metal behavior and, as they usually react via different mechanistic pathways, can offer divergent reactivity. Areas where such main-group systems have shown promise include catalytic dehydrocoupling, hydroamination, hydroboration, and hydrosilylation reactions, as well as examples of stoichiometric oxidative addition and reductive eliminations.^1−5^ Hydroboration reactions are of particular interest as organoborane compounds are widely exploited as synthetic intermediates, owing to their versatility in a range of carbon–carbon and carbon–heteroatom bond formation reactions.^6^ Over the past decade, main-group systems have been shown to efficiently catalyze a host of hydroboration reactions.^1,7−10^ The first example of aluminum-mediated hydroboration dates back to 2000, where a combination of LiAlH_4_, 1,1′-bi-2-naphthol (BINOL), and methanol was found to stoichiometrically reduce acetophenone with HBcat.^11^ However, it was not until 2015 that Roesky, Parameswaran, and Yang reported the first example of aluminum-catalyzed hydroboration.^12^ Here, aluminum β-diketiminate **A** was found to be proficient at catalyzing the room temperature hydroboration of aldehydes and ketones (Figure 1). Catalysis proceeds through an aluminum hydride, with initial hydroalumination of carbonyl carbon, generating aluminum alkoxide, followed by σ-bond metathesis to regenerate **A**. In the intervening years, numerous examples of structurally diverse aluminum catalysts have been reported for the hydroboration of terminal alkynes, nitriles, and alkenes such as conjugated bis-guanidinate-supported aluminum dihydrides (**B**), reported by Nembenna and co-workers (Figure 1).^13−17^ In 2016, the substrate scope of aluminum-catalyzed hydroboration was expanded by Cowley, Thomas, and co-workers to include disubstituted alkynes, using the commercially available bench stable complex triethylaluminium–1,4-diazabicyclo[2.2.2]octane (Et_3_Al–DABCO) (**C**, Figure 1).^18^ High conversions were achieved in 2 h at 110 °C, with a range of different symmetric and asymmetric, aromatic, and aliphatic alkynes found to be tolerated.

However, in both transition metal and main-group catalyzed hydroboration reactions, questions have arisen about the nature of the “true” catalytic species. It has been proposed that boranes, formed in situ, may catalyze hydroboration reactions and that the metal complexes are instead facilitating pinacolborane (4,4,5,5-tetramethyl-1,3,2-dioxaborolane (HBpin)) decomposition/borane formation,^19−22^ although this is more widely documented for HBCat. Recently, a variety of boranes have been shown to be competent hydroboration catalysts for alkynes and alkenes.^23−25^ In 2020, the significance of hidden borane catalysis was extensively investigated by Thomas and co-workers, with nucleophile promoted (inc. LiAlH_4_) HBpin decomposition investigated.^19^ Despite the implications of borane species in catalysis, this had not previously been investigated for any other aluminum catalysts in the literature. We became interested in the mechanism of aluminum-catalyzed hydroboration reactions, and the possibility of in situ borane formation. This was particularly driven by the prohibitively high activation barriers calculated for the aluminum dihydride-promoted hydroboration of alkynes (also noted by Cowley and Thomas), which was proposed to proceed via an initial dehydrogenation reaction to form an acetylide in situ.^13,26^ We aimed to design a series of structurally related complexes whose reactivity could be used to probe the mechanism of alkyne hydroboration through stoichiometric and kinetic analysis. Herein, we present the synthesis of novel aluminum hydride and alkyl complexes bearing amidinate ligands and investigate their use in the catalytic hydroboration of phenylacetylene.

## Synthesis and Reactivity Studies

### Complex Synthesis

**Figure 2:**
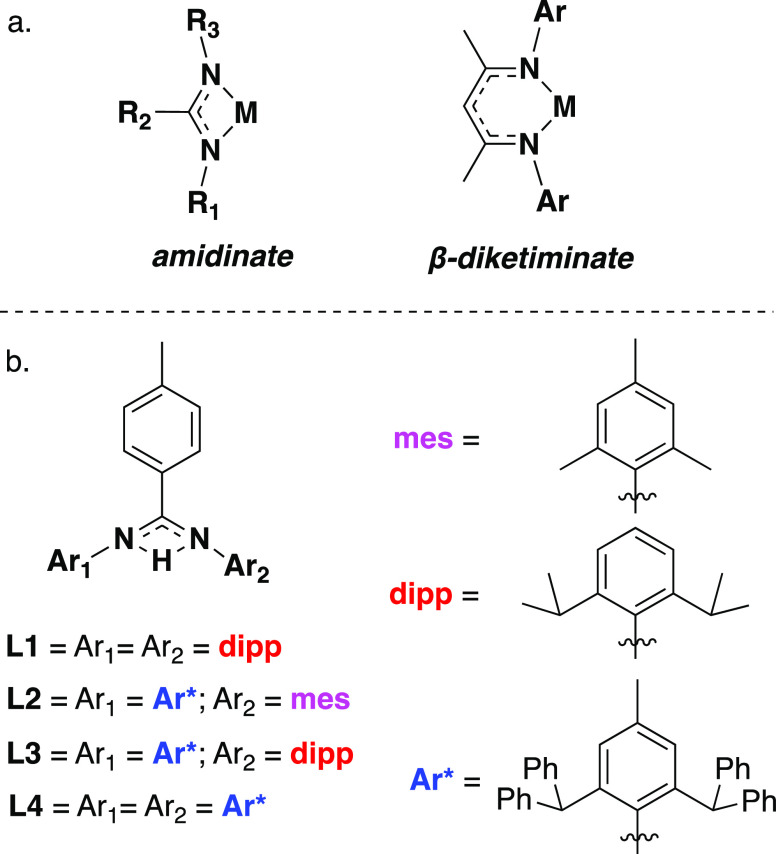
(a) General representation of an amidinate complex (left) and a β-diketiminate complex (right); (b) pro-ligands **L1**–**L4**.

Amidinates are a ubiquitous class of ligand commonly employed in organometallic chemistry (for select examples of group 13 amidinate and related guanidinate complexes, see detailed review articles by Jones and Růžička),^27,28^ with the general structure [R_1_NC(R_2_)NR_3_] (Figure 2a). Their modular synthesis allows for independent tuneability of the substituent on the nitrogen atoms and the substituent at the bridgehead carbon, all of which can be either aromatic or aliphatic. Upon coordination to a metal center, they form a four-membered chelate, though other coordination modes are possible, with a narrow N–M–N bite angle leaving much of the coordinated metal center exposed. As such, their use in the stabilization of highly reactive metal species has been somewhat limited in comparison with the more widely employed β-diketiminate ligand system (Figure 2a). However, recent work has shown that it is possible to use sterically demanding aryl substituents (e.g., 2,6-bis(diphenylmethyl)phenyl) to stabilize a range of highly reactive species, including magnesium and strontium hydrides and an iron(IV) nitride (related guanidinate ligand).^29−31^ We chose to target a series of amidinate ligands with varying degrees of sterically demanding substituents at the R_1_ and R_3_ positions. A 4-methylphenyl group was chosen as the R_2_ group as it aided solubility during ligand synthesis. Pro-ligands **L1**–**L4** (Figure 2b) were synthesized in a modular fashion via modified literature procedures leading to a series of novel symmetric and asymmetric amidinates (for full experimental details, see the Supporting Information (SI)).^32^

**Figure 3:**
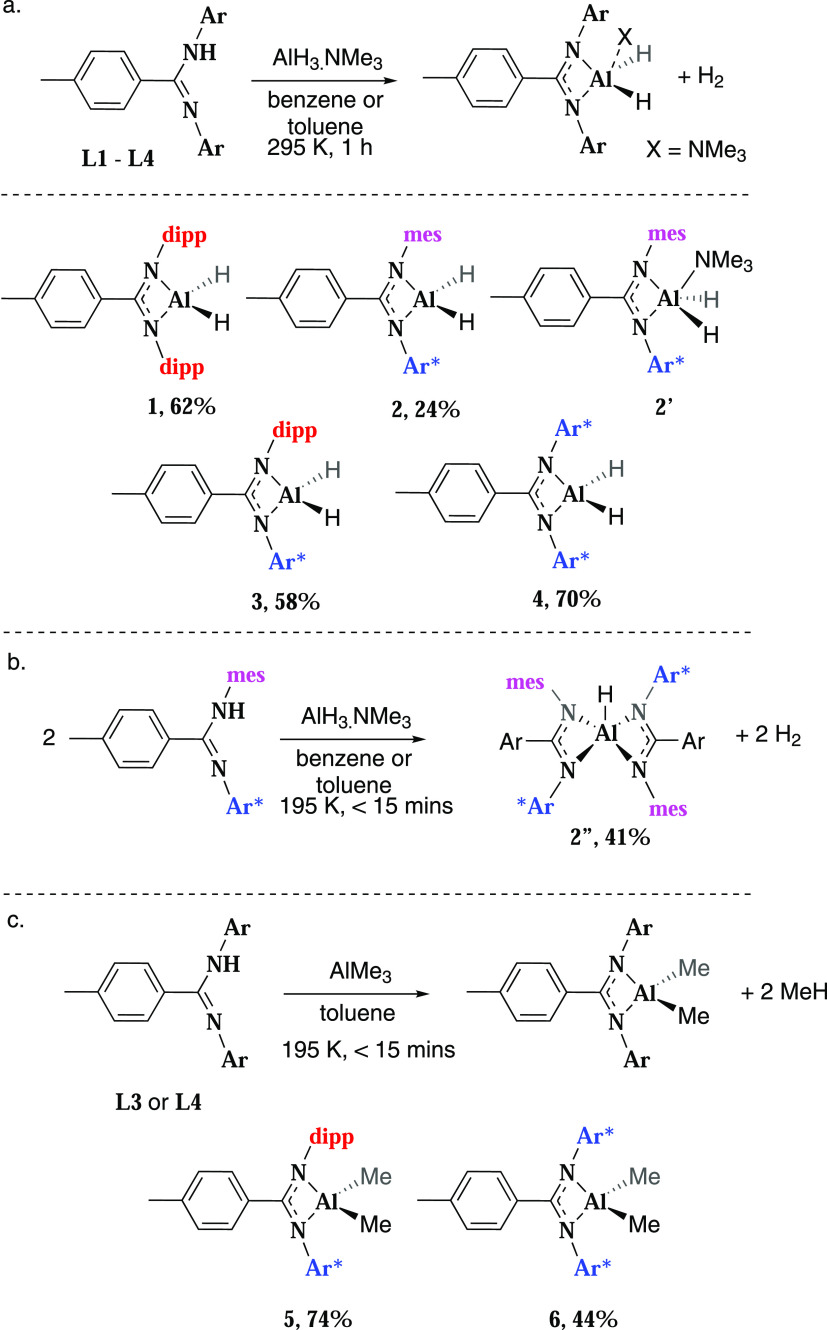
Synthesis of (a) aluminum dihydrides **1**–**4** and **2′**. (b) Aluminum monohydride **2″**. (c) Aluminum dimethyl compounds **5** and **6**.

**Figure 4:**
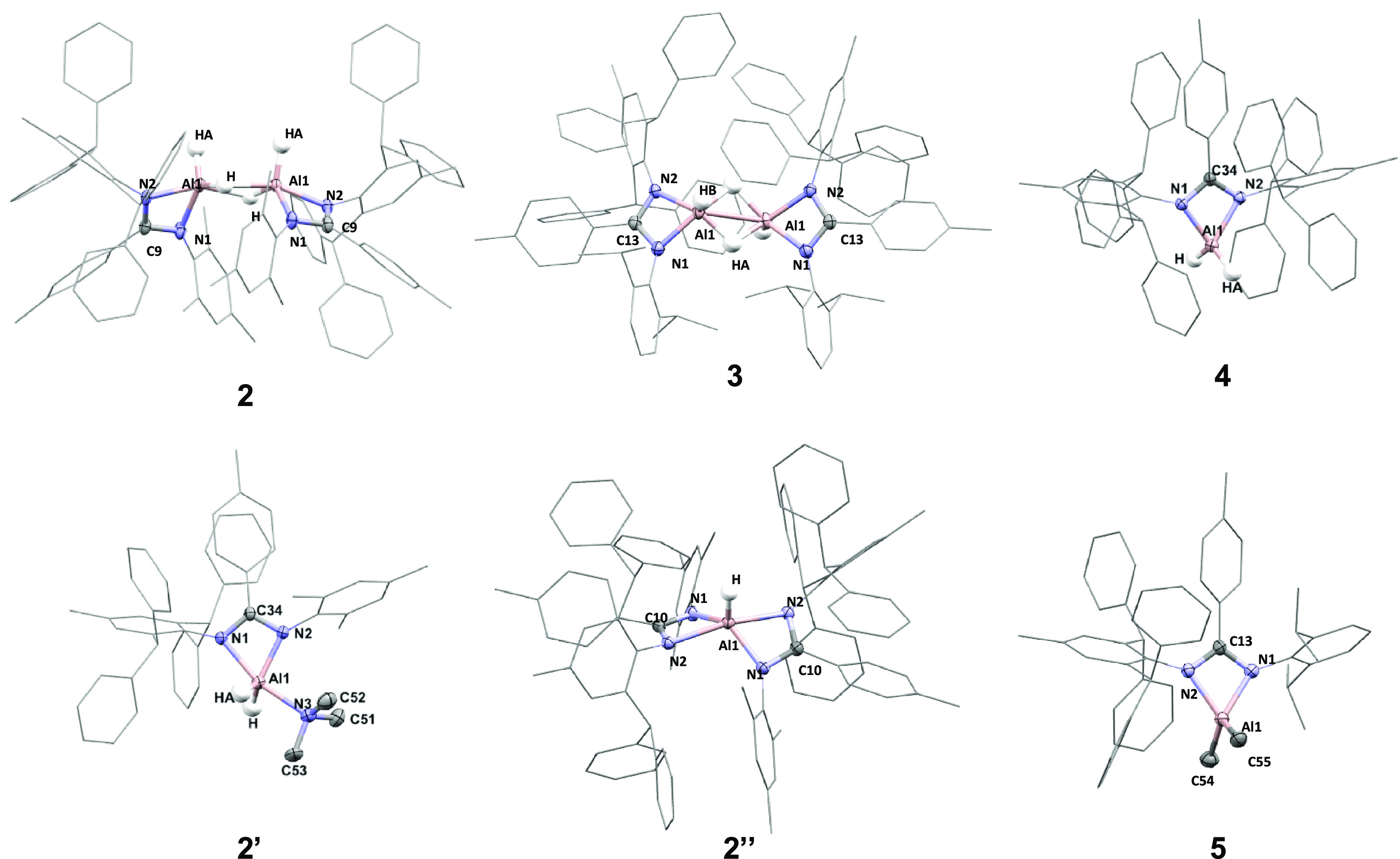
Solid-state structures of compounds **2**, **2′**, **2″**, and **3**–**5**. See the SI for a table of key bond lengths and bond angles (Table S1).

The reactivity of the ligands toward aluminum precursors was then explored. Pro-ligands **L1**–**L4** were added to 1.2 equiv of trimethylamine alane (AlH_3_·NMe_3_) in either benzene-*d*_6_ or toluene at −78 °C (Figure 3a). Hydrogen gas was seen to evolve immediately upon addition, and ^1^H nuclear magnetic resonance (NMR) analysis showed complete consumption of the ligand starting material in 1 h. The ^1^H NMR spectra of the reaction of **L1**, **L3**, and **L4** with the alane precursor revealed the presence of a single set of peaks corresponding to the amidinate ligand and, in all cases, a distinct broad resonance integrating to two hydrides was observed between 3.6 and 5.1 ppm. This strongly indicates the formation of the mono-ligated aluminum dihydride complexes, **1**, **3**, and **4** (Al–*H*_2_: **1** 5.06 ppm; **3** 4.77 ppm; **4** 3.63 ppm). Crystals of **3** and **4** suitable for single-crystal X-ray diffraction were grown from a benzene or benzene/hexane solution. Complex **3** crystallized as a hydride-bridged dimer, in an orthorhombic *Aea*2 space group (Figure 4 and Table S1). The complex, whose hydrides were freely refined, has a slight asymmetry, with one bridging Al–H longer than the other (Al1–HA 1.63904(4) Å; Al1–H 1.74959(4) Å) and is comparable with other related aluminum dihydride species.^33^ In contrast, **4** crystallized as a monomeric aluminum dihydride, with a distorted tetrahedral geometry in a *P*1̅ space group. A distinct interaction between the hydride ligands and two of the amidinate phenyl groups is observed, with through space hydride···phenyl distances of ∼3 Å (Figure S1). Both Al–N and Al–H bond lengths are near identical, with terminal Al–H bond lengths of 1.46(2) Å. This is the first example of a monomeric aluminum dihydride bearing an amidinate ligand in the solid state, which is undoubtably driven by the sterically demanding Ar* ligand substituents. Investigation into the reaction of **L2** with AlH_3_·NMe_3_ revealed an asymmetric ligand environment, a broad singlet at 4.54 ppm corresponding to two Al–*H*, as well as an additional peak at 1.93 ppm integrating to nine protons in the ^1^H NMR spectrum, indicating the presence of trimethylamine. This was confirmed by the solid-state structure which revealed a molecule of trimethylamine datively bound to the aluminum metal center in addition to amidinate ligand (**2′**). Compound **2′** crystallized as a monomeric aluminum dihydride in the *P*1̅ space group. The NMe_3_ group coordinates in the same plane as amidinate nitrogen atoms, approximately orthogonal to the N-terminal Al–H bonds, which are elongated versus the terminal hydrides in **4** (Al–HA 1.47(2) Å; Al–H 1.54(2) Å) and the bound NMe_3_ group render the Al–N bonds of the amindiate ligand slightly asymmetric. The formation of the NMe_3_ adduct is facilitated by the reduced steric bulk of the mesityl substituent versus the more sterically demanding 2,6-diisopropylphenyl (dipp) and 2,6-bis(diphenylmethyl)-4-methylphenyl) groups. No evidence of an amine adduct was observed in the reaction of **L1**, **L3**, and **L4** under any conditions investigated. The formation of the amine free analogue could be achieved by altering the reaction work-up. After removal of solvent, the crude material was subjected to heat (40 °C) and vacuum for 4 h, which led to the elimination of the trimethylamine group and resulted in full conversion to **2**. A distinctive shift in the Al–H signal was observed (4.37 ppm) as well as a restoration of symmetry to the ligand environment. Complex **2** crystallized as a dimer in a *C*2/*c* space group, with the *C*2 axis lying between the two aluminum centers. The terminal and bridging hydrides were freely located, with the terminal aluminum hydrides both facing in the same direction, approximately parallel to one another. The amidinate ligands on each aluminum center are contorted away from one another on the opposing side of the molecule. This is an unusual structure as all other examples of crystallographically characterized bridging aluminum dihydrides have terminal hydrides facing in opposite directions to one another, as in **3**. The Al–H bond lengths to the two bridging hydrides are highly asymmetric, at 1.54(2) and 1.90(2) Å.

Interestingly, a further derivative of **L2** could also be formed. The addition of 2 equiv of **L2** to trimethylamine alane in toluene at −78 °C led to the formation of the bis-ligated product, **2″**. At room temperature, the ^1^H NMR spectrum was broad. This is likely due to steric crowding in the N–C–N region and indicates restricted conformation in solution as well as slow exchange on the NMR time scale. A ^1^H NMR experiment conducted at 70 °C significantly resolved the spectrum, showing five different CH_3_ signals each integrating to six protons. The Al–H resonance was not observed, as is the case with related bis-ligated amidinate aluminum hydrides.^34−36^ The formation of the bis-ligated product was somewhat surprising, given the sterically demanding nature of the 2,6-bis(diphenylmethyl)phenyl substituent. The steric profile of the ligand is highlighted in the solid-state structure (Figure 4), where **2″** is seen to crystallize in a *C*2/*c* space group with a severely distorted trigonal pyramidal geometry and τ value of 0.68 (1 for an ideal trigonal pyramidal geometry).^37^ The Al–N bond lengths were unsymmetrical, with the N–2,4,6-trimethylphenyl (mes) bond length being significantly shorter than the N–Ar* bond length, 1.89(9) versus 2.13(9) Å. This is also observed in related complexes with symmetrical ligands, but to a much lesser extent.^36,38−40^ The terminal aluminum hydride could be freely located and has a bond length of 1.46(2) Å. Attempts to form bis-ligated products with other amidinate ligands discussed were not possible. However, coordination investigations using amidinate ligands could be extended to other aluminum precursors, with the facile formation of the aluminum dimethyl compounds **5** and **6** by reaction of **L3** and **L4** with 1.2 equiv of trimethylaluminum in toluene at −78 °C. ^1^H NMR analysis of **5** and **6** showed a distinctive upfield resonance corresponding to the six Al–Me protons at −0.28 and −0.88 ppm, respectively. The single-crystal X-ray structure of **5** showed a distorted tetrahedral geometry and Al–N and Al–C bond lengths were comparable with other literature compounds.^41^

### Solution versus Solid-State Structures

**Figure 5:**
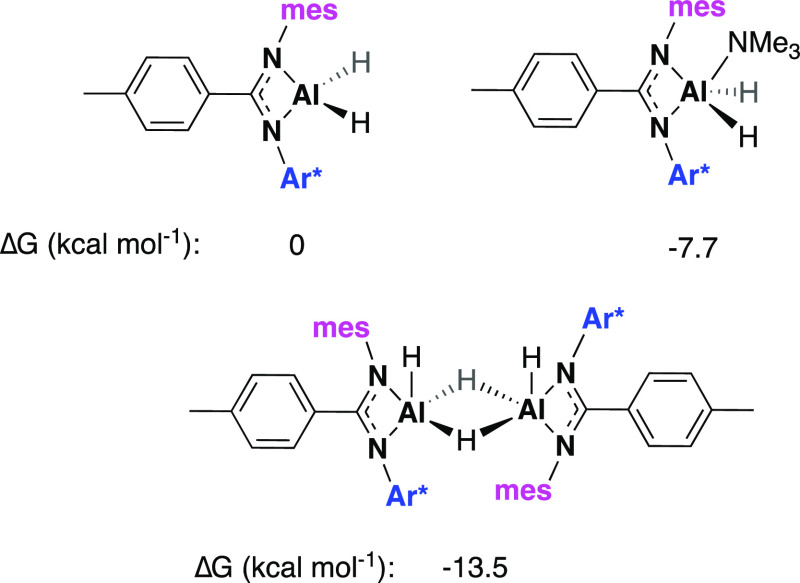
Gibbs free energies of compounds **2**-mono, **2′**, and **2**-dimer.

Compounds **1**–**6**, **2′**, and **2″** show the range of complexes that can be formed across the amidinate ligand series. Solid-state structures reveal that a mixture of monomeric and dimeric structures can form depending on the nature of the ligand employed. However, it is the solution-state structure of aluminum hydrides that dictates their reactivity.^42^ While the ^1^H NMR spectra of complexes **1**–**4** and **2′** show a range of chemical shifts corresponding to the aluminum dihydrides, no definitive correlation between structure and Al–*H* shift could be made (Table S2). For instance, the relatively upfield resonance of **4** at 3.63 ppm is likely due to shielding by the pendant phenyl groups (Figure S2). Diffusion-ordered NMR spectroscopy (DOSY) was therefore used to obtain diffusion coefficients and calculate hydrodynamic radii across the series (Table S2). The monomeric dimethyl complexes **5** and **6** have hydrodynamic radii of 5.8 and 6.8 Å, respectively, demonstrating that Ar* has a significantly greater solution volume than the dipp substituent. The hydrodynamic radii of **5** and **6** are in good agreement with structurally related monomeric complexes reported in the literature.^43−45^ Comparatively, the dihydride complexes **3** and **4** both have slightly larger hydrodynamic radii than their analogous counterparts (1.4× and 1.3× respectively), indicating a solution-based monomer–dimer equilibrium that lies toward a monomeric structure but with some dimeric character. In contrast, compound **2** was found to have the largest radius across the series (7.6 Å), despite having a smaller ligand than **3** and **4**, suggesting a larger degree of dimeric character in solution. **2′**, whose additional NMe_3_ ligand disfavors dimerization, has a significantly smaller hydrodynamic radius. Density functional theory (DFT) calculations were used to further probe the most stable solution-based structure of the series of mesityl compounds. These indicate that it is most thermodynamically favorable for **2** to exist in its dimeric form (Figure 5), which is in line with experimental observations.^a^ Compounds **2**, **2′**, and **3** are the first examples of aluminum hydride complexes bearing asymmetric amidinate ligands, and the family of compounds represents a rare series of structurally distinct aluminum complexes. With this in mind, we were keen to explore the reactivity of the compounds and discern trends across this series.

### Catalysis

The series of structurally related aluminum alkyl and hydride compounds **1**–**6** were used to investigate the mechanism of aluminum-catalyzed alkyne hydroboration. Previous work has shown both aluminum alkyls and hydrides to be effective hydroboration catalysts.^14,42,46,47^ The reaction of aluminum hydrides **1**–**4** and aluminum alkyls **5** and **6** with phenylacetylene and pinacolborane (HBpin) at 0.25 M concentration in benzene-*d*_6_ was monitored over time using ^1^H NMR spectroscopy. At room temperature, the slow formation of a hydroboration product was observed, with 38% conversion obtained in 12 h (**3**). However, the rates of reaction were seen to dramatically increase when reactions were conducted at 80 °C, as such all complexes were tested under these conditions. All compounds were found to be catalytically active toward the hydroboration reaction, with the exclusive formation of the (*E*)-vinylboronate ester product via anti-Markovnikov addition observed in all cases (Table 1). The reactions took between 6 and 95 h to reach high conversion (>88%), depending on the structure of the aluminum complex employed. Using compound **1**, the reaction went to completion in 6 h, while **6** was the most sluggish with high conversions only reached after 95 h. To gain a better understanding of how the reactions progress over time, they were monitored at regular intervals using ^1^H and ^11^B NMR spectroscopy.

**Table 1:**
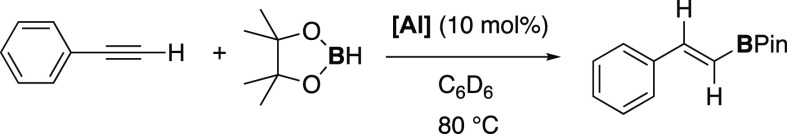
Catalytic Hydroboration of Phenylacetylene with HBpin^a^

cat.	time (h)	conv. (%)^b^
**1**	6	89
**2**	22	89
**3**	11	89
**4**	56	89
**5**	12	90
**6**	95	89

a Reactions conducted in benzene-*d*_6_, 0.25 M [HBpin], and [phenylacetylene], 10 mol % cat. at 80 °C.

b Calculated by ^1^H NMR, mesitylene was used as an internal standard.

Analysis of the plot of [HBpin] versus time for hydroboration reactions employing aluminum hydride compounds **1**–**4** revealed two different catalytic regimes, one at low conversion and a second at high conversion (>75% conversion, Figures S45–S48). This indicates that at high conversion an alternative pathway, side reactions, or catalyst decomposition starts to become more prominent. Comparison of the aluminum hydrides **1**–**4** reveals significant differences in the rate of reaction across the series, with reactivity following the overall trend **1** > **3** > **2** > **4** (Table 1 and Figures S52 and S53). There is a general trend linking sterics and the reaction rate, with the most sterically encumbered compound **4** exhibiting the slowest initial rate. However, compound **2**, which contains a mesityl substituent in its ligand framework, is significantly slower than **3** (22 versus 11 h to reach 89% conversion), which contains a more sterically demanding diisopropylphenyl group in the same position. The alkyl compounds **5** and **6** were found to exhibit significantly different reaction kinetics. In both cases, there was a lag period before any hydroboration product was observed to form (1 h **5**, 7 h **6**, Figures S47 and S48), indicating the slow formation of an active catalytic species. Also, in neither case was an obvious second regime observed. Comparison between the hydride and alkyl complexes showed high conversions were reached on a similar time scale for **3** and **5**, but that **6** was significantly slower than **4**. The differing kinetic profiles between the hydride and alkyl complexes suggest that different reaction mechanisms are in play. Similarly, the differences in the rate of reaction across the series indicate that catalyst structure is important and potentially contravenes the suggestion that a common catalytic species may be in operation, though it could also suggest that they are formed at different rates. The activity of AlH_3_·NMe_3_ toward the hydroboration reaction was also investigated, with 48% of the (*E*)-vinylboronate ester observed after 2 h under the same conditions. However, after this point, the reaction slowed and did not proceed to completion (13 h, 54%, Figure S51). This is unsurprising given the temperature-sensitive nature of AlH_3_·NMe_3_ and suggests that the compound degrades under the reaction conditions.

**Figure 6:**
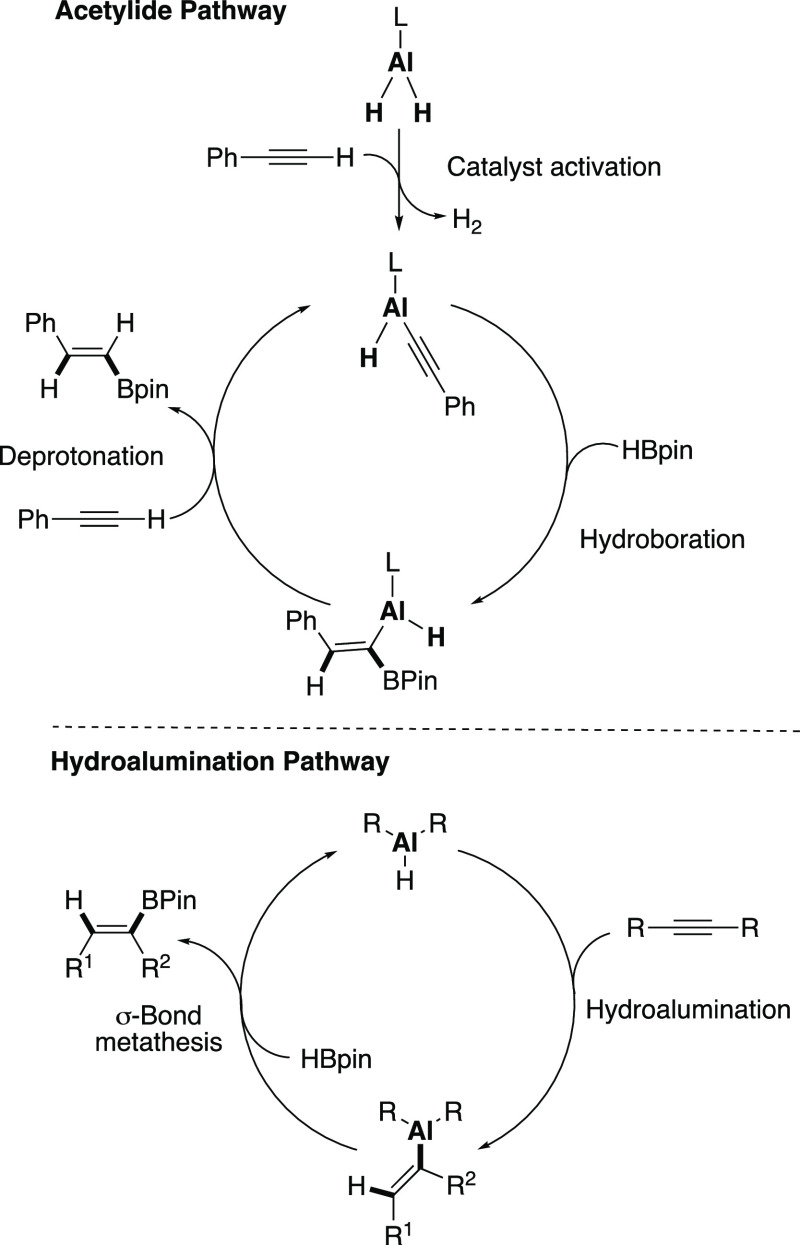
Previously proposed mechanisms for aluminum-catalyzed hydroboration of alkynes: acetylide pathway (top) and hydroalumination pathway (bottom).

**Figure 7:**
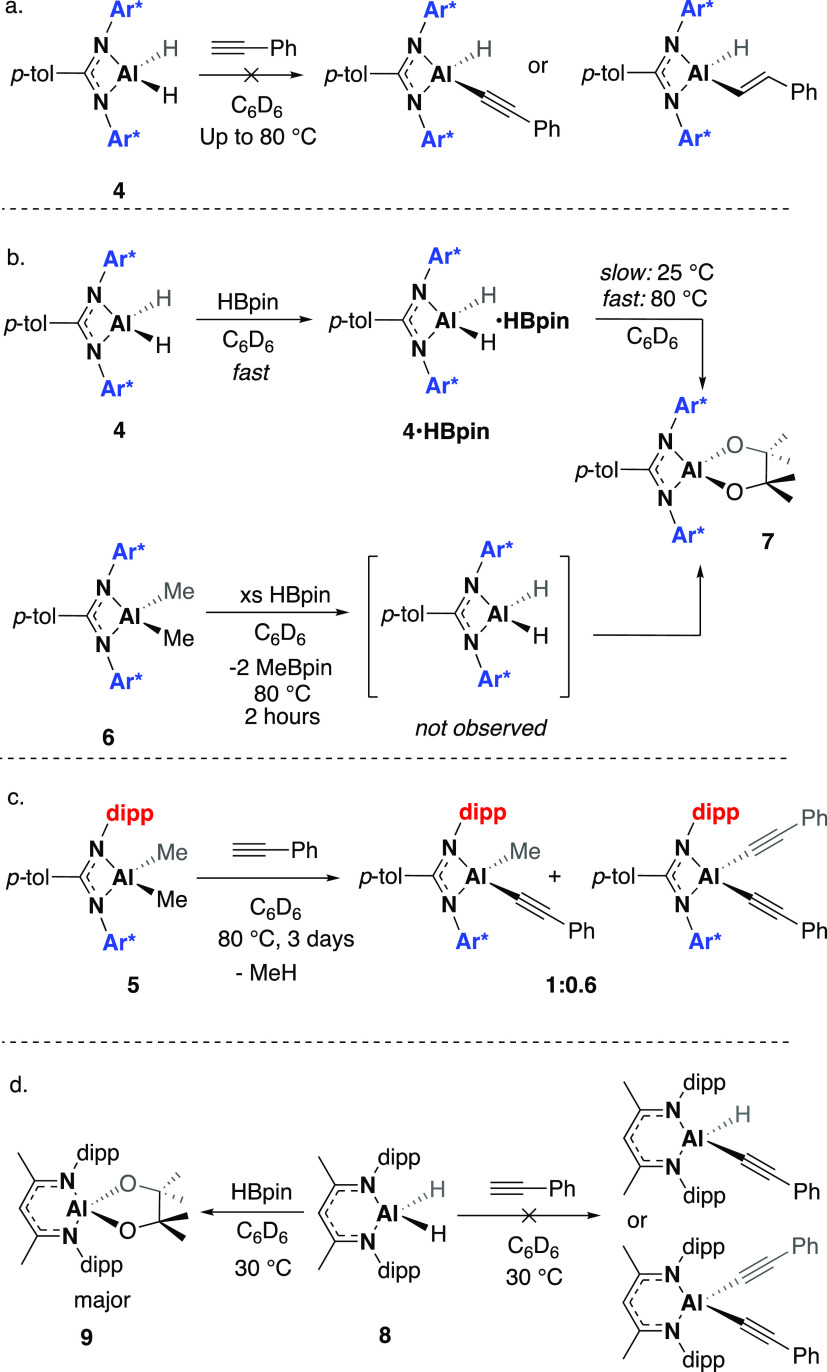
Stoichiometric reactions with **4**–**6** and **8**.

**Figure 8:**
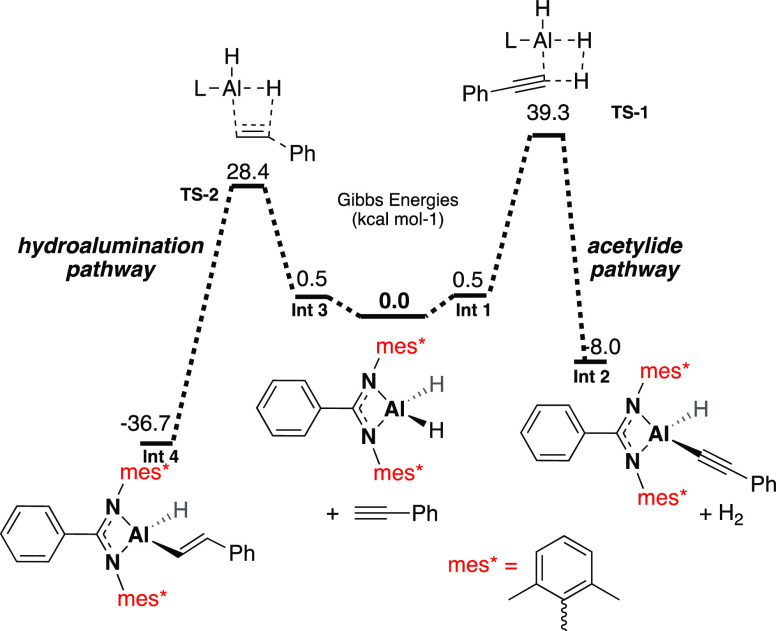
Calculated reaction pathway for the initial steps of the hydroalumination pathway (left-hand side, LHS) and the acetylide pathway (right-hand side, RHS).

**Figure 9:**
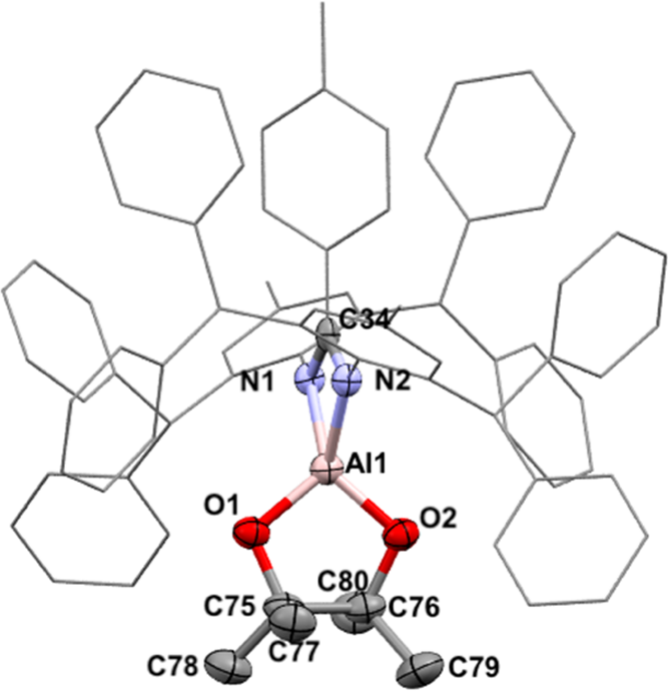
X-ray structure of **7** with hydrogen atoms omitted for clarity. Selected bond lengths (Å) and angles (deg): N1–Al1 1.904(1), N2–Al1 1.895(1), Al1–O1 1.721(1), and Al1–O2 1.718(1); N1–Al1–N2 70.59(6), O1–Al1–O2 98.47(6).

Previous studies have proposed two plausible reaction mechanisms for the aluminum-catalyzed hydroboration of acetylenes; (a) the dehydrogenative formation of the active aluminum acetylide catalyst, followed by hydroboration and subsequent protonation of the alkenyl group (acetylide pathway, Figure 6 top) or (b) the hydroalumination of acetylene followed by a σ-bond metathesis with HBpin to form the vinylboronate ester product and regeneration of the aluminum hydride catalyst (hydroalumination pathway, Figure 6 bottom).^13,18^ Cowley and co-workers used the latter mechanism to explain the ability of their aluminum precursors to hydroborate internal alkynes.^18^ To help determine the most likely mechanism(s) in play, a series of stoichiometric reactions was conducted. First, the stability of the substrates and catalysts were explored; heating the compounds at 80 °C in benzene-*d*_6_ for 16 h saw no decomposition observed in any instance. Similarly, heating the substrates in the absence of an aluminum complex under the same conditions saw no reaction or formation of product (Figures S4 and S5). NMR-scale reactions between **3**/**4** and phenylacetylene, in benzene-*d*_6_, were used to probe the possible acetylide pathway or hydroalumination pathway (Figure 7a).^b^ However, no reaction was observed at room temperature, and after heating at 80 °C for 4 h, only a small amount of decomposition was observed (Figures S6–S8). Heating for longer periods of time (24–120 h) showed further decomposition and trace amounts of hydrogen formation. This is in contrast to other aluminum dihydride systems, which report the formation of either the dehydrogenation or insertion product.^26,48^ DFT calculations were conducted to investigate the viability of the two proposed reaction pathways (Figure 8).^a^ A simplified aluminum complex was used, and transition states were located for the initial stage of both pathways. The insertion (hydroalumination) of acetylene into the Al–H bond was found to be both kinetically and thermodynamically favorable, with a Δ*G* of activation of 28.4 kcal mol^–1^. However, this is still a significant energy barrier as the hydroboration reaction does proceed slowly at room temperature; therefore, we would expect a lower associated energy. In contrast, addition of HBpin to **4** saw the immediate formation of a new product, proposed to be a HBpin adduct **4·HBpin** (Figures 7b, S9, and S10). Here, the Al–*H* signal is shifted to 3.40 ppm, and the methyl groups of the pinacolate group resonate as two signals at 1.20 and 1.32 ppm. A broad signal ∼−10 ppm is observed in the ^11^B NMR spectrum (Figure S11). The formation of a Lewis acidic adduct was ruled out owing to the observed loss of symmetry of methyl pinacolate groups. ^1^H–^11^B NMR correlation spectroscopy was attempted to further investigate the structure of this adduct, but experiments were unsuccessful due to the short-lived nature of the adduct at room temperature. Heating the reaction at 80 °C for 30 min led to the formation of a second species, which was identified as the aluminum pinacolate **7** (Figure 7b), and the disappearance of the resonance at −10 ppm in the ^11^B NMR spectrum (Figure S12).^c^ A distinctive signal at 1.45 ppm was observed in the ^1^H NMR spectrum, corresponding to the 12 protons of the pinacolate group. The solid-state structure shows a puckered pinacolate moiety, with roughly equal Al–O bond lengths (1.718(1)/1.721(1) Å) and an O–Al–O angle of 98.5° (Figure 9). A contraction of Al–N bonds is observed compared to the parent dihydride **4** (Al–N **7**: 1.897(1)/1.904(1) Å; Al–N **4**: 1.943(10)/1.939(10) Å). A related β-diketiminate complex revealed nearly identical Al–N bond lengths (1.897(1)/1.904(1) Å) but possessed longer Al–O bonds (1.718(1)/1.721(1) Å) and a smaller O–Al–O angle (92.7°).^49^ Monitoring the reaction in situ showed a quintet at −34.6 ppm in the ^11^B NMR spectrum, corresponding to a [BH_4_]^−^ species. Similar reactivity was observed with **3**, but the isolation of the proposed pinacolate was not possible (Figures S13 and S14).

The transfer of the pinacolate group from boron to aluminum must occur with the generation of BH_3_ which then further reacts to form a [BH_4_]^−^ species. While BH_3_ generated could act as a catalytic species, formation of **7** was not observed during any catalytic reactions. It is also worth noting that **7** does not react further with HBpin, and it does not catalyze the reaction. Thus, BH_3_/[BH_4_]^−^ formation was ruled out in the presence of phenylacetylene via this pathway. A handful of aluminum pinacolates have been previously reported; however, there are no reported examples of pinacolate formation from HBpin.^49,50^ In fact, to the best of our knowledge, this is the first example of pinacolate transfer from boron to a metal center, though partial pinacolate transfer has been observed with a magnesium–magnesium dimer, magnesium hydride, and a scandium hydride species.^51,52^

The stoichiometric reactivity of **2** followed a slightly different pattern. The reaction of **2** with 1 equiv of phenylacetylene at 80 °C led to the formation of an unidentified product along with decomposition, as well as the formation of styrene and trace H_2_ (Figure S15). The reaction of **2** with HBpin at 298 K also showed the formation of an unidentified product (no new pinacolate signal is observed) in addition to the production of BH_3_, as confirmed by a quartet at −7.0 ppm in the ^11^B NMR spectrum. This product decomposed slowly at room temperature and at an increased rate at 80 °C (Figures S16 and S17). Reactions were also conducted at room temperature with an analogous β-diketiminate (**8**, Figure 7d). No reaction was observed between **8** and phenylacetylene over the course of 24 h (Figures S18 and S19). As with **4**, the reaction of **8** with HBpin proceeded quickly at 30 °C in both C_6_D_6_ and CDCl_3_. In both cases, the immediate formation of an intermediate (likely the adduct) was observed, followed by the slower formation of a second product, which was identified as the pinacol transfer product **9**, with concomitant formation of a [BH_4_]^−^ species (Figures S20–S22).^d^ In combination, these results suggest neither the initial step of the acetylide pathway nor the hydroalumination pathway accurately describe our system. The different reactivity of **2** and **3**/**4** also hints that these complexes may catalyze the reaction via different mechanisms.

The alkyl complexes were found to be significantly more stable at high temperatures; the reaction of **5** with phenylacetylene (80 °C, benzene-*d*_6_, 24 h) led to the formation of a major and minor product in the ^1^H NMR spectrum (Figures S23 and S24). Desymmetrization of methine protons and formation of methane gas suggested the formation of the aluminum acetylide complex. A second symmetrical product was also observed and is proposed to be the bisacetylide complex. In contrast, after 5 days, at 80 °C, **6** only showed minor reactivity, with a new resonance at −0.92 and methane formation indicating the slow formation of an analogous acetylide complex (Figure S25). Attempts to grow single crystals of any products were unsuccessful. The reaction of **6** with 1 equiv. HBpin led to a mixture of products; HBpin was fully consumed in 2 h (80 °C) with the formation of MeBpin and the aluminum pinacolate **7**, along with unreacted **6** (Figures S26 and S27).^53^ It is presumed that this reaction proceeds via the aluminum dihydride intermediate, **4**, which can then react further with HBpin. Indeed, when **6** was reacted with an excess of HBpin, complete formation of **7** was achieved after 7 h at 80 °C (Figures S28 and S29).

## Mechanistic Discussion

The stoichiometric reactions suggest that the acetylide pathway or hydroalumination pathway pathways previously proposed by Roesky and Cowley and Thomas, respectively, are not applicable to our system. There was no evidence for the reaction between any of the aluminum dihydride complexes **1**–**4** and phenylacetylene, the first step in each catalytic pathway, only decomposition of the complex over time. It is also worth noting that no reaction was observed with complexes **2**, **3**, or **6** when phenylacetylene was replaced with diphenylacetylene. While there is a clear reaction between compounds **3** and **4** with HBpin, we have seen no evidence for the formation of the pinacolate species **7** or [BH_4_]^−^ under catalytic conditions. This suggests that the formation of the hydroboration product is more favorable.

Analyzing the catalytic reaction mixture by ^11^B NMR provides further insight. In addition to the starting material (doublet, 28 ppm) and the hydroboration product (broad singlet, 30 ppm), several other peaks were observed to form during the reaction. Reactions employing compounds **1**, **3**, and **4** all contain a small peak at 22 ppm which develops over time, whereas reactions using **2**, **5**, and **6** contain peaks at 22 and 24 ppm. The precise identity of these peaks remains unaccounted for; however, related compounds with “N–Bpin” bonds have been reported in this region (21–25 ppm).^54^ The formation of a peak at 22 ppm when **3** is mixed with diphenylacetylene and HBpin also supports the proposed formation of a “N–Bpin” bond. Interestingly, the point at which these new species begin to form is the point at which the reaction kinetics start to deviate.

The lack of reactivity between the aluminum dihydrides **1**, **3**, and **4** and phenylacetylene in the absence of HBpin leads us to propose that these reactions are more likely to proceed via a HBpin adduct. Adduct formation has previously been proposed by Rueping and co-workers for the hydroboration of alkynes; here, the active species is proposed to be HBpin bound to BuMg–H via a pinacolate oxygen.^55^ However, in the case of our complexes, this would create a coordinatively saturated aluminum center, thus prohibiting catalytic turnover. Indeed, attempts to use DFT to calculate such a compound were unsuccessful. We therefore propose that the HBpin instead either (a) binds to Al through oxygen and causes the ligand to partially de-coordinate or (b) coordinates directly to the ligand framework in some fashion. As the reaction progresses, these adducts start to degrade to form the proposed “N–Bpin” species observed in the ^11^B NMR. The formation of these “N–Bpin” compound(s) is supported by the rather complex kinetic profiles observed in these catalytic reactions.

The hydroboration reaction proceeded significantly slower in the presence of **2** than the more sterically encumbered **3**. While **2** was shown to have a more dimeric solution character, which could account for this reduced rate, reactions using compound **2** also showed more complicated reactivity, with a distinctive quartet observed at −7 ppm in the ^11^B NMR spectrum, corresponding to the formation of BH_3_. The BH_3_ signal increased in intensity throughout the course of the reaction and was also observed in the stoichiometric reaction of **2** with HBpin. It was noted in the stoichiometric reactions that compound **2** did not follow the same reactivity as **3** and **4**. This hints at the possibility of an alternate reaction mechanism, whereby BH_3_, formed in situ via the aluminum hydride promoted decomposition of HBpin, is contributing to the catalyst.^20,21,56^ Borane can then react with the alkyne substrate (hydroboration) to form an alkenylborane intermediate which then undergoes transborylation with a further molecule of HBpin to yield the desired product and regenerate the BH_3_ catalyst.^19^ In reality, in the presence of **2**, the reaction is probably proceeding by multiple catalytic species including **2** (assuming the slow formation of BH_3_), BH_3_, and any additional aluminum decomposition products formed. The observed formation of BH_3_ from **2**, but not **1**, **3**, and **4** points toward a lack of stability of the complex, which contains a smaller mesityl substituent. Although it is possible that BH_3_ may form at different rates for different catalysts, the marked difference in the solution and solid-state structure of **2** supports the trend of divergent reactivity. To further rule out BH_3_ formation, **4** was reacted with 10 equiv of HBpin to mirror catalytic reaction conditions. No difference in reactivity was observed when reacted with 1 or 10 equiv (Figures S29–S31).

Analysis of reactions using **5** and **6** shows that they have an initial lag period before any hydroboration product is formed. As in stochiometric reactions with HBpin, MeBpin formation was observed, in addition to a peak at 22 ppm indicating the presence of a small amount of a “N–Bpin” compound which grows in gradually through the course of the reactions (vide supra). “N–Bpin” formation does not appear to coincide with a drop in the rate of reaction as with dihydride compounds. Product formation coincided with MeBpin production, suggesting that an aluminum hydride generated in situ is required for catalysis to occur. However, the significantly different reaction kinetics led us to rule out the formation of dihydrides **3** and **4**. Instead, it is possible that a mixed alkyl-hydride species is formed, which could then go onto react via the acetylide pathway. This is supported by the formation of trace amounts of hydrogen in the reaction mixture, which indicated the formation of an aluminum acetylide species, but requires further investigation.

## Conclusions

In summary, we have reported a series of structurally varied aluminum hydride and alkyl complexes, including the first example of an unsupported monomeric aluminum amindinate dihydride. All compounds have been fully characterized, and the solid state and solution structures compared. The solution-state monomer–dimer equilibrium revealed that **1**, **3**, and **4** exist predominantly as monomers, whereas **2** retains much of its dimeric character in solution. The aluminum hydrides **1**–**4** and aluminum alkyls **5** and **6** were all found to mediate the hydroboration of phenylacetylene, with rates of reaction linked to structure, size, and stability.

Detailed stoichiometric and kinetic analysis revealed different reaction mechanisms across the series. The aluminum dihydrides **1**, **3**, and **4** all led to the same kinetic profile, and stoichiometric reactions with phenylacetylene showed no reaction. It is therefore proposed that these complexes proceed via the formation of a HBpin adduct, which becomes the active catalyst. This is in contrast to previous reports using analogous β-diketiminate complexes, which propose an aluminum acetylide, formed by a dehydrogenation reaction between an aluminum dihydride and acetylene, to be the active catalytic species. Interestingly, complex **2** was found to be less stable under the reaction conditions (as observed in stoichiometric reactions) and promoted the degradation of HBpin, with BH_3_ formation observed during catalysis. This is in agreement with recent reports of borane-catalyzed hydroboration and highlights the importance of complex stability in mechanistic investigation.^19,57^ The variation in reaction rate across the series, combined with in situ ^11^B NMR analysis of the catalytic reaction mixtures, indicates that the reactions are mediated by the aluminum species (with the exception of **2**) and not via hidden borane species. The precise nature of the proposed aluminum–HBpin active catalyst is currently under investigation computationally and will be the subject of further work. We will also continue to probe the complex kinetics displayed by a seemingly simple reaction system.

The structure of the catalyst has proved critical in determining the mechanism, even across a closely related series of compounds. Relatively, subtle differences in ligand structure have been found to have profound effects on catalysis. Additionally, different co-ligands Al–H versus Al–Me have been shown to operate by distinct mechanisms. This highlights how nuanced complex structure can be in determining reactivity, but also offers the opportunity to access divergent reaction pathways through simple synthetic manipulations.

## Experimental Section

### General Procedures

All reactions were carried out using standard Schlenk-line and glovebox techniques under an inert atmosphere of argon. An MBraun Unilab Pro glovebox was used. Solvents were obtained from a Grubbs solvent purification system (SPS), degassed and stored on 3 Å molecular sieves prior to use. Anhydrous benzene-*d*_6_ was obtained from Sigma and was degassed, and stored on 3 Å molecular sieves. NMR-scale reactions were conducted in J. Young’s tap tubes and prepared in a glovebox. All heating of YT NMR tubes was conducted in a DrySyn NMR tube heating block at the temperature stated. Details of synthesis of ligands **L1**–**L4** can be found in the SI. Trimethylaluminium, 2 M in toluene was obtained from Sigma and used without further purification. Phenylacetylene was purchased from Sigma, distilled using CaH_2_, and stored over 3 Å molecular sieves. 4,4,5,5-Tetramethyl-1,3,2-dioxaborolane (HBpin) and diphenylacetylene were purchased from Sigma and used without further purification. Nuclear magnetic resonance (NMR) spectra were recorded on Bruker Avance 400, 500, and 600 spectrometers operating at 400, 500, and 600 MHz for ^1^H NMR, respectively, and 100, 125, and 150 MHz, respectively, for ^13^C NMR. Spectra were processed and analyzed using Mestrenova and Bruker Topspin software. The following notation system for the ligand moieties has been implemented below: L = *p*-toluidine backbone, mes = 2,4,6-trimethylphenyl substituent, dipp = 2,6-diisopropylphenyl substituent, and Ar* = 2,6-diphenylmethyl-4-methylphenyl substituent. In NMR analysis, Ph refers to aromatic. Italicized o, m, and p refers to the ortho, meta, and para positions, respectively. C^IV^ refers to quaternary carbons.

#### Synthesis of **1**

A solution of **L1** (1.1 mmol, 500 mg) dissolved in toluene (7 mL) was added dropwise at −78 °C to a solution of trimethylamine alane (1.54 mmol, 137 mg) in toluene (7 mL) at −78 °C. Hydrogen gas was seen to evolve immediately, and the reaction was stirred for 1 h at 298 K. The solvent was removed in vacuo before the product was extracted into hexane (2 × 10 mL) and filtered. The solvent was removed in vacuo and the product isolated as a white solid (328 mg, 62%).

^1^H NMR (500 MHz, C_6_D_6_, 298 K) δ (ppm): 1.03 (d, 12H, CH(C**H**_3_)_2_, ^3^*J*_HH_ = 6.5 Hz), 1.31 (d, 6H, CH(C**H**_3_)_2_, ^3^*J*_HH_ = 7.0 Hz), 1.63 (s, 3H, ^L^C**H**_3_), 3.67 (sept, 4H, C**H**(CH_3_)_2_, ^3^*J*_HH_ = 7.0 Hz), 5.06 (s, 2H, Al**H**_2_), 6.38 (d, 2H, ^L^*o*-CH, ^3^*J*_HH_ = 8.5 Hz), and 7.03–7.08 (m, 8H, ^Ph^C**H**); ^13^C{^1^H} NMR (125 MHz, C_6_D_6_, 298 K) δ (ppm): 21.2 (^L^**C**H_3_), 22.4 (CH(**C**H_3_)_2_), 25.9 (CH(**C**H_3_)_2_), 29.3 (**C**H(CH_3_)_2_), 123.4 (^Ph^**C**H), 123.9 (^Ph^**C**H), 126.0 (^Ph^**C**H), 128.6 (^L^*o*-**C**H), 129.9 (^Ph^**C**H), 138.7 (**C**^IV^), 141.3 (**C**^IV^), and 143.5 (**C**^IV^). Anal. Calcd (C_32_H_43_N_2_Al): C, 79.63; H, 8.98; and N, 5.80. Found: C, 79.57; H, 9.08; and N, 5.85.

#### Synthesis of **2**

A solution of **L2** (0.30 mmol, 200 mg) dissolved in toluene (7 mL) was added dropwise at −78 °C to a solution of trimethylamine alane (0.36 mmol, 31 mg) in toluene (7 mL) at −78 °C. Hydrogen gas was seen to evolve immediately, and the solution was stirred for 1 h at 298 K. The solvent was removed in vacuo, and the resultant mixture heated under vaccum (40 °C) for 4 h. The crude product was washed with hexane, filtered, and isolated to yield a white solid (49 mg, 24%).

^1^H NMR (600 MHz, C_6_D_6_, 298 K) δ (ppm): 1.63 (s, 3H, ^L^CH_3_), 1.78 (^Ar*^CH_3_), 2.00 (s, 3H, ^mes^*p*-CH_3_), 2.10 (s, 6H, ^mes^*o*-CH_3_), 5.02 (s, 2H, AlH_2_), 6.02 (d, 2H, ^L^*o*-CH, ^3^*J*_HH_ = 7.8 Hz), 6.22 (br d, 2H, ^L^*m*-CH), 6.39 (s, 2H, CH(Ph_2_)) 6.51 (s, 2H, ^mes^*m*-CH), 6.87–7.19 (m, ^Ph^CH), 6.95 (^Ar*^m-CH), and 7.37 (d, 4H, ^Ph^*o*-CH, ^3^*J*_HH_ = 7.8 Hz); ^13^C{^1^H} NMR (150 MHz, C_6_D_6_, 298 K) δ (ppm): 20.4 (^mes^*m*-CH_3_), 21.0 (^L^CH_3_), 21.0 (^Ar*^CH_3_), 21.1 (^mes^*p*-CH_3_), 21.4 (C^IV^), 53.5 (CH(Ph_2_)), 123.5 (^Ph^CH), 126.0 (^Ph^CH), 126.4 (^Ph^CH), 128.5, 128.6 (^Ph^CH), 128.7 (^Ph^CH), 129.0 (^Ph^CH), 129.3 (^L^*m*-CH), 129.6 (^mes^*m*-CH), 130.1 (^L^*o*-CH), 130.1 (^Ph^*o*-CH), 130.5 (^Ph^CH), 133.9 (C^IV^), 134.5 (C^IV^), 137.4 (C^IV^), 139.1 (C^IV^), 142.2 (C^IV^), 143.0 (C^IV^), 143.8 (C^IV^), and 146.1 (C^IV^). IR (solid-state): n = 1872, (solution): *n* = 1716, 1752 cm^–1^.

#### Synthesis of **2′**

To a solution of trimethylamine alane (0.028 mmol, 2.5 mg) dissolved in benzene-*d*_6_, **L2** (0.028 mmol, 18 mg) was added, and the solution was transferred to a J Young NMR tube; H_2_ gas was seen to evolve. The mixture was left at room temperature for 1 h. Single crystals suitable for X-ray analysis were grown from benzene-*d*_6_/hexane. Attempts to scale up this reaction were unsuccessful.

^1^H NMR (500 MHz, C_6_D_6_, 298 K) δ (ppm): 1.67 (s, 3H, ^L^C**H**_3_), 1.82 (s, 3H, ^Ar*^C**H**_3_), 1.99 (s, 18H, N(C**H**_3_)_3_), 2.00 (s, 3H, ^mes^*p*-C**H**_3_), 2.31 (s, 6H, ^mes^*o*-C**H**_3_), 4.10 (s, 4H, Al**H**_2_), 5.91 (s, 2H, C**H**(Ph)_2_), 6.07 (d, 2H, ^L^*o*-C**H**, ^3^*J*_HH_ = 8.0 Hz), 6.30 (d, 2H, ^L^*m*-C**H**, ^3^*J*_HH_ = 8.0 Hz), 6.61 (s, 2H, ^mes^*m*-C**H**), 7.03–7.20 (m, 12H, C**H**), 7.33 (d, 4H, ^Ar^*o*-C**H**, ^3^*J*_HH_ = 7.5 Hz), and 7.62 (d, 4H, ^Ar^*o*-C**H**, ^3^*J*_HH_ = 7.5 Hz); ^13^C{^1^H} NMR (125 MHz, C_6_D_6_, 298 K) δ (ppm): 18.5 (^L^**C**H_3_), 19.6 (^mes^*o*-**C**H_3_), 20.5 (^mes^*p*-**C**H_3_), 22.0 (^Ar*^CH_3_), 50.5 (^Ph^**C**H), 125.7 (**C**^IV^), 125.9 (^Ph^**C**H), 128.5 (^L^*m*-**C**H_3_), 128.8 (^mes^*m*-**C**H), 129.1 (**C**^IV^), 129.5 (**C**^IV^), 130.0 (^Ph^*o*-**C**H), 130.5 (^Ph^*o*-**C**H), 132.5 (**C**^IV^), 133.9 (**C**^IV^), 134.9 (**C**^IV^), 138.7 (**C**^IV^), 143.7 (**C**^IV^), and 147.4 (**C**^IV^).

#### Synthesis of **2″**

A solution of **L2** (14.5 mmol, 100 mg) dissolved in toluene (7 mL) was added dropwise at −78 °C to a solution of trimethylamine alane (7.3 mmol, 6.5 mg) in toluene (7 mL) at −78 °C. The reaction was stirred for 1 h before the solvent was removed in vacuo. The crude product was washed with hexane, filtered, and isolated as a white solid. Single crystals suitable for X-ray analysis were grown from toluene/hexane (166 mg, 41%).

^1^H NMR (500 MHz, C_6_D_6_, 343 K) δ (ppm): 1.63 (s, 6H, C**H**_3_), 1.85 (s, 6H, C**H**_3_), 1.90 (s, 6H, C**H**_3_), 2.13 (s, 6H, C**H**_3_), 2.46 (s, 6H, C**H**_3_), 5.95 (s, 8H, C**H**), 6.19 (s, 2H, C**H**), 6.35 (s, 2H, C**H**), 6.88–7.12 (bm, 40H, C**H**), and 7.30 (bm, 8H, C**H**). It was not possible to assign ^13^C NMR spectra at 298 or 343 K. IR (solid-state): *n* = 1867 cm^–1^.

#### Synthesis of **3**

A solution of **L3** (0.279 mmol, 200 mg) dissolved in toluene (7 mL) was added dropwise at −78 °C to a solution of trimethylamine alane (0.297 mmol, 25 mg) in toluene (7 mL) at −78 °C. Hydrogen gas was seen to evolve immediately, and the solution was stirred for 1 h at 298 K. The crude product was washed with hexane, filtered, and isolated as a white solid. Single crystals suitable for X-ray analysis were grown from benzene-*d*_6_ at 298 K (120 mg, 58%).

^1^H NMR (500 MHz, C_6_D_6_, 298 K) δ (ppm): 0.79 (d, 6H, CH(C**H**_3_)_2_, ^3^*J*_HH_ = 6.5 Hz), 1.26 (d, 6H, CH(C**H**_3_)_2_, ^3^*J*_HH_ = 6.5 Hz), 1.69 (s, 3H, ^L^C**H**_3_), 1.86 (s, 3H, ^Ar*^C**H**_3_), 3.52 (sept, 2H, C**H**(CH_3_)_2_, ^3^*J*_HH_ = 6.5 Hz), 6.13 (d, 2H, ^L^*o*-C**H**, ^3^*J*_HH_ = 8 Hz), 6.53 (d, 2H, ^L^*m*-C**H**, ^3^*J*_HH_ = 7.5 Hz), 6.79 (s, 2H, C**H**(Ph)_2_), 6.98–7.14 (m, 21 H, ^Ph^C**H**), and 7.47 (d, 4H, ^Ph^C**H**, ^3^*J*_HH_ = 7.5 Hz); ^13^C{^1^H} NMR (125 MHz, C_6_D_6_, 298 K) δ (ppm): 21.3 (^Ar*^CH_3_), 21.6 (^L^**C**H_3_), 23.7 (CH(**C**H_3_)_2_), 25.4 (CH(**C**H_3_)_2_), 28.1 (**C**H(CH_3_)_2_), 51.5 (**C**H(Ph)_2_), 124.6 (^Ph^**C**H), 128.4 (^L^*o*-**C**H), 128.5 (^Ph^**C**H), 128.8 (^Ph^**C**H), 129.7 (^Ph^**C**H), 129.8 (^L^*m*-**C**H), 130.8 (^Ph^**C**H), 130.0 (**C**^IV^), 130.2 (**C**^IV^), 133.2 (**C**^IV^), 138.9 (**C**^IV^), 143.6 (**C**^IV^), 144.3 (**C**^IV^), and 146.1 (**C**^IV^). IR (solid-state): *n* = 1838, 1879 cm^–1^, (solution): *n* = 1813, 1958 cm^–1^. Anal. Calcd (C_53_H_53_N_2_Al): C, 84.42; H, 7.53; and N, 4.10. Found: C, 83.67; H, 7.07; and N, 3.76.

#### Synthesis of **4**

A solution of **L4** (0.204 mmol, 200 mg) in toluene (7 mL) was added dropwise at −78 °C to a solution of trimethylamine alane (0.204 mmol, 18 mg) in toluene (7 mL) at −78 °C. Hydrogen gas was seen to evolve immediately, and the solution was stirred for 1 h at 298 K. The crude product was washed with hexane, filtered, and isolated as a white solid. Single crystals suitable for X-ray analysis were grown from benzene-*d*_6_/hexane at 298 K (145 mg, 70%).

^1^H NMR (500 MHz, C_6_D_6_, 298 K) δ (ppm): 0.79 (d, 6H, CH(C**H**_3_)_2_, ^3^*J*_HH_ = 6.5 Hz), 1.26 (d, 6H, CH(C**H**_3_)_2_, ^3^*J*_HH_ = 6.5 Hz), 1.69 (s, 3H, ^L^C**H**_3_), 1.86 (s, 3H, ^Ar*^C**H**_3_), 3.52 (sept, 2H, C**H**(CH_3_)_2_, ^3^*J*_HH_ = 6.5 Hz), 6.13 (d, 2H, ^L^*o*-C**H**, ^3^*J*_HH_ = 8 Hz), 6.53 (d, 2H, ^L^*m*-C**H**, ^3^*J*_HH_ = 7.5 Hz), 6.79 (s, 2H, C**H**(Ph)_2_), 6.98–7.14 (m, 21 H, ^Ph^C**H**), and 7.47 (d, 4H, ^Ph^C**H**, ^3^*J*_HH_ = 7.5 Hz); ^13^C{^1^H} NMR (125 MHz, C_6_D_6_, 298 K) δ (ppm): 21.3 (^Ar*^CH_3_), 21.6 (^L^**C**H_3_), 23.7 (CH(**C**H_3_)_2_), 25.4 (CH(**C**H_3_)_2_), 28.1 (**C**H(CH_3_)_2_), 51.5 (**C**H(Ph)_2_), 124.6 (^Ph^**C**H), 128.4 (^L^*o*-**C**H), 128.5 (^Ph^**C**H), 128.8 (^Ph^**C**H), 129.7 (^Ph^**C**H), 129.8 (^L^*m*-**C**H), 130.8 (^Ph^**C**H), 130.0 (**C**^IV^), 130.2 (**C**^IV^), 133.2 (**C**^IV^), 138.9 (**C**^IV^), 143.6 (**C**^IV^), 144.3 (**C**^IV^), and 146.1 (**C**^IV^). IR (solid-state): *n* = 1618 cm^–1^, (solution): *n* = 1753 cm^–1^. Anal. Calcd (C_74_H_63_N_2_Al): C, 87.68; H, 6.50; and N, 2.96. Found: C, 86.94; H, 6.18; and N, 2.74.

#### Synthesis of **5**

Ligand **L3** (0.28 mmol, 200 mg) was dried under vacuum for 1 h, dissolved in toluene (10 mL), and cooled to −78 °C. Trimethylaluminum (0.34 mmol, 0.17 mL) was added dropwise, and the reaction was stirred at 298 K overnight. The solvent was removed in vacuo, washed with hot hexane, and recrystallized at −18 °C. The product was filtered, dried, and isolated as a white solid (160 mg, 74%).

^1^H NMR (500 MHz, C_6_D_6_, 298K) δ (ppm): −0.035 (s, 6H, Al(C**H**_3_)_2_), 0.77 (d, 6H, CH(C**H**_3_)_2_, ^3^*J*_HH_ = 7.0 Hz), 1.23 (d, 6H, CH(C**H**_3_)_2_, ^3^*J*_HH_ = 6.5 Hz), 1.72 (s, 3H, ^L^C**H**_3_), 1.81 (s, 3H, ^Ar*^p-C**H**_3_), 3.43 (sept, 2H, C**H**(CH_3_)_2_, ^3^*J*_HH_ = 7.0 Hz), 6.21 (d, 2H, ^L^o-C**H**, ^3^*J*_HH_ = 8.5 Hz), 6.49 (s, 2H, C**H**(Ph)_2_), 6.65 (d, 2H, ^L^*m*-C**H**, ^3^*J*_HH_ = 8.5 Hz), and 6.95–7.28 (m, 25H, ^Ph^C**H**); ^13^C{^1^H} NMR (500 MHz, C_6_D_6_, 298K) δ (ppm): −9.6 (Al(**C**H_3_)_2_), 21.2 (^L^**C**H_3_), 21.2 (^Ar*^CH_3_), 23.2 (CH(**C**H_3_)_2_), 25.7 (CH(**C**H_3_)_2_), 28.3 (**C**H(CH_3_)_2_), 51.6 (C**H**(Ph)_2_), 128.8 (^L^*o*-**C**H), 129.7 (^L^*m*-**C**H), 124.1 (^Ph^**C**H), 125.9 (^Ph^**C**H), 126.1 (^Ph^**C**H), 128.5 (^Ph^**C**H), 128.6 (^Ph^**C**H), 129.4 (^Ph^**C**H), 129.6 (^Ph^**C**H), 130.1 (**C**^IV^), 130.2 (**C**^IV^), 131.4 (**C**^IV^), 134.0 (**C**^IV^), 139.1 (**C**^IV^), 140.6 (**C**^IV^), 143.5 (**C**^IV^), 143.9 (**C**^IV^), and 145.2 (**C**^IV^). Anal. Calcd (C_55_H_57_N_2_Al): C, 84.47; H, 7.80; and N, 3.94. Found: C, 84.84; H, 7.34; and N, 3.58.

#### Synthesis of **6**

Ligand **L4** (0.20 mmol, 200 mg) was dried under vacuum for 1 h, dissolved in toluene (10 mL), and cooled to −78 °C. Trimethylaluminum (0.26 mmol, 0.13 mL) was added dropwise, and the reaction was stirred at 298 K overnight. The solvent was removed in vacuo, and the resultant product was washed with hexane to yield a white solid after filtration (93 mg, 44%).

^1^H NMR (500 MHz, C_6_D_6_, 298K) δ (ppm): −0.88 (s, 6H, Al(C**H**_3_)_2_), 1.73 (s, 6H, ^Ar*^C**H**_3_), 1.82 (s, 3H, ^L^C**H**_3_), 6.06 (d, 2H, ^L^*o*-C**H**, ^3^*J*_HH_ = 8.5 Hz), 6.43 (s, 4H, C**H**(Ph)_2_), 6.96–7.11 (m, 38H, ^Ph^C**H**), and 7.23 (d, 8H, ^Ph^*o*-C**H**, ^3^*J*_HH_ = 8 Hz); ^13^C{^1^H} NMR (500 MHz, C_6_D_6_, 298K) δ (ppm): 21.5 (^Ar*^CH_3_), 51.2 (**C**H(Ph)_2_), 126.0 (^Ph^**C**H), 126.5 (^Ph^**C**H), 128.2 (^L^*m**-*****C**H), 129.0 (^L^*o*-**C**H), 129.4 (^Ph^*o*-**C**H), 130.3 (^Ph^**C**H), 131.5 (**C**^IV^), 134.4 (**C**^IV^), 138.9 (**C**^IV^), 139.5 (**C**^IV^), 144.2 (**C**^IV^), and 145.2 (**C**^IV^). Anal. Calcd (C_76_H_65_N_2_Al): C, 88.17; H, 6.52; and N, 2.71. Found: C, 87.78; H, 6.56; and N, 2.72.

#### Synthesis of **7**

Compound **4** (0.0496 mmol, 50 mg) was dissolved in toluene (5 mL), and HBpin (0.0546 mmol, 8 mL) was added. The reaction was heated for 30 min at 80 °C before the solvent was removed in vacuo. The crude product was recrystallized from toluene/hexane to yield colorless crystals (18 mg, 33%).

^1^H NMR (600 MHz, C_6_D_6_, 298 K) δ (ppm): 1.45 (s, 12H, CH_3_), 1.65 (s, 3H, ^L^CH_3_), 1.69 (s, 6H, ^Ar*^CH_3_), 5.64 (d, 2H, ^L^*o*-CH, ^3^*J*_HH_ = 8.4 Hz), 6.14 (d, 2H, ^L^*m*-CH, ^3^*J*_HH_ = 6.0 Hz), 6.36 (s, 4H, CH(Ph_2_)), 7.00 (s, 4H, ^Ar*^m-CH), 6.80–7.33 (m, ^Ph^CH), and 7.67 (d, 8H, ^Ph^*o*-CH, ^3^*J*_HH_ = 8.4 Hz); ^13^C{^1^H} NMR (150 MHz, C_6_D_6_, 298 K) δ (ppm): 14.2 (C^IV^), 20.8 (^L^CH_3_), 22.9 (^Ar*^CH), 28.0 (CH_3_), 31.0 (C^IV^), 51.2 (CH(Ph)_2_), 77.8 (C^IV^), 125.6 (^Ph^CH), 126.4 (^Ph^CH), 128.2 (^L^*o*-CH), 128.2 (^Ph^CH), 129.4 (^Ph^CH), 129.9 (^L^*m*-CH), 129.9 (^Ph^*o*-CH), 130.1 (C^IV^), 130.2 (C^IV^), 131.2 (^Ph^CH), 131.3 (C^IV^), 134.8 (C^IV^), 136.5 (C^IV^), 139.1 (C^IV^), 142.2 (C^IV^), and 146.6 (C^IV^).

### General Procedure for the Catalytic Hydroboration of Phenylacetylene

Phenylacetylene (0.015 mmol, 0.0165 mL) was added to a solution of catalyst (0.0015 mmol, 10 mol %), 1,3,5-trimethylbenzene (0.01 mL), and HBpin (0.015 mmol, 0.0215 mL) in benzene-*d*_6_ (0.60 mL) and transferred to a J Young NMR tube. A *t* = 0, ^1^H NMR spectrum was recorded, and the sample tube was then heated at 80 °C. Each reaction was monitored over time, with ^1^H NMR spectra recorded at regular time points until >88% completion was achieved. The yield was determined by ^1^H NMR spectroscopy using 1,3,5-trimethylbenzene as an internal standard.

^1^H NMR (500 MHz, C_6_D_6_, 298 K) δ (ppm): 1.13 (s, 12H, C**H**_3_), 6.46 (d, 1H, C**H**, ^3^*J*_HH_ = 18.5 Hz), 6.98–7.04 (m, 3H ^Ph^C**H**), 7.32 (d, ^3^*J*_HH_ = 10.0 Hz), and 7.76 (d, 1H, C**H**, ^3^*J*_HH_ = 18.5 Hz).

## Supporting Information Available

Full details of experiments and analysis; General experimental section; Synthetic procedures; Additional data; Kinetics; X-ray crystallography; Multinuclear NMR data; and XYZ coordinates (PDF)

## Accession Codes

CCDC 2054524–2054530 contain the supplementary crystallographic data for this paper. These data can be obtained free of charge via www.ccdc.cam.ac.uk/data_request/cif, or by emailing data_request@ccdc.cam.ac.uk, or by contacting The Cambridge Crystallographic Data Centre, 12 Union Road, Cambridge CB2 1EZ, UK; fax: +44 1223 336033.

## Footnotes

a The calculations were performed at a C H N (6-31G**)/Al (SDDAll), ωB97X level of theory + Δ*E*_sp_ (ωB97XD) + Δ*E*_solv_ (polarizable continuum model (PCM), benzene). Basis set and functional were benchmarked by comparison to the solid-state structure of **4**.

b Reaction of **3** with phenylacetylene under the same conditions saw significantly more decomposition.

c The product **7** was also shown to form slowly at room temperature.

d Both reactions also saw the formation of a second unidentified minor product (approximately 40%).
