# Supplementary material for: Aluminum Amidinates: Insights into Alkyne Hydroboration
Source: Inorg Chem. 2021 Jul 16;60(15):10958–69. doi: 10.1021/acs.inorgchem.1c00619 (PMC8388121; doi:10.1021/acs.inorgchem.1c00619)
Supplement: Supplementary file 1 — ic1c00619_si_001.pdf [file ic1c00619_si_001.pdf]

Aluminum amidinates: insights into alkyne hydroboration

Katie Hobson, Claire J. Carmalt* and Clare Bakewell*

Supporting Information

Department of Chemistry, University College London, 20 Gordon Street, London, WC1H 0AJ

Table of Contents

**1**	**General Experimental Section**	**S2**
**2**	**Synthetic Procedures**	**S3**
**3**	**Additional Data**	**S6**
**4**	**Kinetics**	**S48**
**5**	**X-ray Crystallography**	**S57**
**6**	**Multinuclear NMR Data**	**S60**
**7**	**References**	**S85**
**8**	**XYZ coordinates**	**S86**

1. General Experimental Section

All reactions were carried out using standard Schlenk-line and glovebox techniques under an inert atmosphere of argon. An MBraun Unilab Pro glovebox was used. Solvents were obtained from a Grubbs solvent purification system (SPS), degassed and stored on 3Å molecular sieves prior to use. Anhydrous benzene-d_6 was obtained from Sigma and was degassed and stored on 3Å molecular sieves. NMR-scale reactions were conducted in J. Young's tap tubes and prepared in a glovebox. All heating of YT NMR tubes was conducted in a DrySyn NMR tube heating block at the temperature stated.

Benzyhdrol, 2,6-diisopropylaniline and trimethyl aluminium, 2M in toluene, were obtained from Sigma and used without further purification. Phenylacetylene was purchased from Sigma, distilled using CaH_2 and stored over 3Å molecular sieves. 4,4,5,5-Tetramethyl-1,3,2-dioxaborolane (HBpin) and diphenylacetylene were purchased from Sigma and used without further purification. *P*-toluoyl chloride and 2,4,6-trimethylaniline and lithium aluminium hydride were obtained from Fisher Scientific Ltd. Trimethylamine hydrochloride was purchased from Aldrich and used without further purification. Trimethyl amine alane was synthesised according to literature procedures.[1]

Nuclear magnetic resonance (NMR) spectra were recorded on Bruker Avance 400, 500 and 600 spectrometers operating at 400, 500 and 600 MHz for ^1H NMR, respectively, and 100, 125 and 150 MHz, respectively, for ^{13}C NMR. Spectra were processed and analysed using Mestrenova and Bruker Topspin software. Elemental analysis has been included for as many compounds as possible.

2. Synthetic Procedures

2.1 Ligand synthesis

The following notation system for the ligand moieties has been implemented below: L = *p*-toluidine backbone, *mes* = 2,4,6-trimethylphenyl substituent, *dipp* = 2,6-diisopropylphenyl substituent, *Ar** = 2,6-diphenylmethyl-4-methylphenyl substituent. In NMR analysis, *Ph* refers to aromatic. Italicised *o, m, p* refers to the *ortho*, *meta* and *para* positions, respectively. C^{IV} refers to quaternary carbons.

2,6-Diphenylmethyl-4-methylaniline

Para-toluidine (40 mmol, 4.36 g) and benzhydrol (80 mmol, 15.0 g) were heated to a melt (150 °C). A solution of zinc dichloride, $ZnCl_2$, (20 mmol, 2.71 g) in hydrochloric acid (12 M, 3 mL) was added dropwise and the reaction was heated at reflux (433 K) for 3 h, then allowed to cool to 298 K. The product was dissolved in dichloromethane (DCM) and washed with ammonium chloride (NH_4Cl) solution and then brine. The product was dried ($NaHCO_3$, silica gel), filtered and the solvent removed *in vacuo*, before washing with minimum amounts of ethyl acetate, to yield a white powder (9.0 g, 59%).[2]

^{1}H NMR: (400 MHz, $CDCl_3$, 298K) δ (ppm): 2.04 (s, 3H, CH_3), 3.29 (s, 2H, $CH(Ph)_2$), 5.47 (s, 2H, NH2), 6.40 (s, 2H, $CH(Ph)_2$), 7.11-7.32 (m, 20H, ^{Ph}CH).

L1: Ar^{dipp}Ar^{dipp}LH

2,6-Diisopropylaniline (13.6 mmol, 2.5 mL), *para*-toluoyl chloride (13.6 mmol, 2.11 g) and triethylamine (NEt_3) (11.0 mmol, 1.55 mL) were dissolved in DCM (100 mL) and stirred at 298 K for 48 h. The product was washed with Na_2CO_3 (3 x 100 mL) and dried ($MgSO_4$). The solvent was removed *in vacuo* and the resultant amide was isolated as a white powder. The crude product was recrystallised from DCM (2.94 g, 77 %). 2,6-Diisopropylphenyl-4-methylbenzamide (6.78 mmol, 2.0 g) and phosphorus pentachloride (8.13 mmol, 1.69 g) were dissolved in toluene (150 mL) under an inert atmosphere and heated at reflux overnight. The solvent was removed *in vacuo* and the reaction mixture was heated at 423 K for 1 hour. The resultant imidoyl chloride was dissolved in toluene (100 mL), 2,6-diisopropylaniline (6.78 mmol, 1.27 mL) and triethylamine (8.08 mmol, 1.22 mL) were added and the reaction was heated at reflux for 48 h. The product was extracted with toluene/ether, washed (1 x $NaHCO_3$, 2 x H_2O), dried ($MgSO_4$) and the solvent removed *in vacuo*. The crude product was recrystallised from ethanol (0.84 g, 27 %).

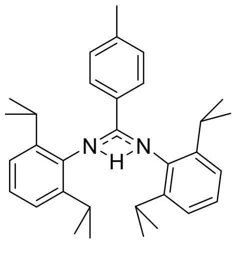

^{1}H NMR (500 MHz, $CDCl_3$, 298 K) δ (ppm): 0.89 (d, 6H, C**H**$_3$, $^3J_{HH}$ = 6.5 Hz), 0.99 (d, 6H, C**H**$_3$, $^3J_{HH}$ = 6.5 Hz), 1.25 (d, 6H, C**H**$_3$, $^3J_{HH}$ = 6.5 Hz), 1.36 (d, 6H, C**H**$_3$, $^3J_{HH}$ = 7.0 Hz), 2.28 (s, 3H, ^{L}C**H**$_3$), 3.22 (m, 4H, C**H**(CH_3)$_2$), 5.68 (s, 1H, N**H**), 6.89 - 7.29 (m, 12H, ^{Ph}C**H**); ^{13}C{^{1}H} NMR (125 MHz, $CDCl_3$, 298 K) δ (ppm): 21.5 (^{L}CH$_3$), 22.3 (**C**H$_3$), 22.7 (**C**H$_3$), 24.9 (**C**H$_3$), 25.3 (**C**H$_3$), 28.5 (C^{IV}), 28.7 (**C**H(CH_3)$_2$), 122.8 (^{Ph}**C**H), 123.3 (^{Ph}**C**H), 123.4 (C^{IV}), 123.7 (^{Ph}**C**H), 127.6 (^{Ph}**C**H), 128.5 (^{Ph}**C**H), 128.8 (C^{IV}), 128.9 (^{Ph}**C**H), 132.3 (C^{IV}), 134.2 (C^{IV}), 139.2 (C^{IV}), 139.5 (C^{IV}), 144.0 (C^{IV}), 145.3 (C^{IV}). Anal. Calc. ($C_{32}H_{42}N_2$): C, 84.53; H, 9.31; N, 6.16. Found: C, 83.70; H, 9.30; N, 6.11.

*L2: Ar*Ar^{mes}LH*

2,6-Diphenylmethyl-4-methylaniline (13.6 mmol, 6.0 g), *para*-toluoyl chloride (13.6 mmol, 2.11 g) and triethylamine (11.0 mmol, 1.55 mL) were dissolved in DCM (100 mL) and stirred at 298 K for 48 h. The product was washed with Na_2CO_3 (3 x 100 mL) and dried ($MgSO_4$). The solvent was removed *in vacuo* and the resultant amide was isolated as a white powder (6.46 g, 84 %). 2,6-Diphenylmethyl-4-methylphenyl-4-methylbenzamide (4.48 mmol, 2.50 g) and phosphorus pentachloride (5.38 mmol, 1.12 g) were dissolved in toluene (150 mL) under an inert atmosphere and heated at reflux overnight. The solvent was removed *in vacuo* and the reaction mixture was heated at 423 K for 1 hour. The resultant imidoyl chloride was dissolved in toluene (100 mL), 2,4,6-trimethylaniline (4.48 mmol, 0.63 mL) and triethylamine (5.82 mmol, 0.80 mL) were added and the reaction was heated at reflux for 48 h. The product was extracted with toluene/ether, washed (1 x $NaHCO_3$, 2 x H_2O), dried ($MgSO_4$) and the solvent removed *in vacuo*. The crude product was recrystallised from hot methanol (1.30 g, 44 %).

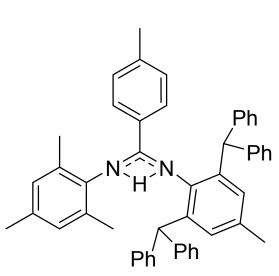

^{1}H NMR (500 MHz, $CDCl_3$, 298K) δ (ppm): 2.13 – 2.20 (m, 15H, C**H**$_3$), 4.75 (s, 1H, N**H**), 6.06 (s, 2H, C**H**(Ph)$_2$), 6.56 (s, 2H, ^{mes}*m*-C**H**), 6.69 (s, 2H, ^{Ph}C**H**), 6.73 (d, 2H, ^{L}*m*-C**H**, $^3J_{HH}$ = 7.5 Hz), 6.82 (d, 2H, C^B**H**, $^3J_{HH}$ = 7 Hz), 6.97 (br s, 4H, ^{Ph}C**H**), 7.15 (br s, 14H, ^{Ph}C**H**); ^{1}H NMR (125 MHz, $CDCl_3$, 333K) δ (ppm): 2.22 (s, 6H, ^{mes}*m*-C**H**$_3$) 2.28 (s, 3H, ^{L}C**H**$_3$), 2.34 (s, 3H, C**H**$_3$), 2.46 (s, 3H, ^{Ph}C**H**$_3$), 4.84 (s, 1H, N**H**), 6.08 (s, 2H, C**H**(Ph)$_2$), 6.89 (s, 2H, ^{mes}*m*-C**H**), 6.77 – 7.28 (m, 25H, ^{Ph}C**H**); ^{13}C{1H} NMR (500 MHz, $CDCl_3$, 298K): 19.6 (**C**H$_3$), 20.8 (**C**H$_3$), 21.4 (**C**H$_3$), 21.9 (^{Ph}CH$_3$), 52.6 (**C**H(Ph)$_2$), 126.3 (^{Ph}CH), 126.8 (^{Ph}**C**H), 128.3 (^{Ph}CH), 128.8 (^{Ph}**C**H), 129.0 (^{Ph}CH), 129.5 (^{Ph}CH), 130.1 (^{Ph}**C**H), 134.3 (C^{IV}), 136.6 (C^{IV}), 139.1 (C^{IV}), 143.3 (C^{IV}), 143.6 (C^{IV}), 143.9 (C^{IV}), 146.1 (C^{IV}). Anal. Calc. ($C_{50}H_{46}N_2$): C, 88.98; H, 6.87; N, 4.15. Found: C, 88.30; H, 6.89; N, 4.16.

*L3: Ar*Ar^{dipp}LH*

2,6-Diphenylmethyl-4-methylphenyl-4-methylbenzamide (8.96 mmol, 5.0 g) and phosphorus pentachloride (10.75 mmol, 2.24 g) were dissolved in toluene (150 mL) under an inert atmosphere and heated at reflux overnight. The solvent was removed *in vacuo* and the reaction mixture was heated at 423 K for 1 h. The resultant imidoyl chloride was dissolved in toluene (100 mL), 2,6-diisopropylaniline (8.96 mmol, 1.6 mL) and triethylamine (11.65 mmol, 1.6 mL) were added and the reaction was heated at reflux for 48 h. The product was extracted with toluene/ether, washed (1 x $NaHCO_3$, 2 x H_2O), dried ($MgSO_4$) and the solvent removed *in vacuo*. The crude product was recrystallised from hot methanol (4.21 g. 62%).

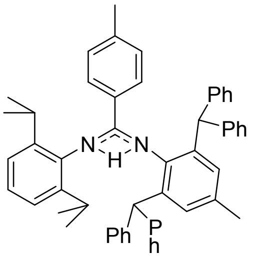

^{1}H NMR (500 MHz, $CDCl_3$, 298K) δ (ppm): 0.81 (d, 6H, CH(C**H**$_3$)$_2$, $^3J_{HH}$ = 6.5 Hz), 1.11 (d, 6H, CH(C**H**$_3$)$_2$, $^3J_{HH}$ = 6.5 Hz), 2.15 (s, 3H, ^{Ar*}C**H**$_3$), 2.19 (s, 3H, ^{L}C**H**$_3$), 3.30 (sept, 2H, C**H**(CH-$_3$)$_2$, $^3J_{HH}$ = 6 Hz), 4.75 (s, 1H, N**H**), 5.92 (s, 2H, C**H**(Ph)$_2$), 6.50 (d, 2H, ^{L}*o*-C**H**, $^3J_{HH}$ = 7 Hz), 6.55 (s, 2H, ^{Ar*}*m*-C**H**), 6.79 (d, 2H, ^{L}*m*-C**H**, $^3J_{HH}$ = 7 Hz), 6.93– 7.15 (m, 21H, ^{Ph}C**H**); ^{13}C{1H} NMR (125 MHz, $CDCl_3$, 298K) δ ppm: 21.3 (^{L}CH$_3$), 21.8 (^{Ar*}**C**H$_3$), 22.9 (CH(**C**H$_3$)$_2$), 24.6 (CH(**C**H$_3$)$_2$), 28.5 (**C**H(CH$_3$)$_2$), 52.8 (**C**H(Ph)$_2$), 122.1 (C^{IV}), 122.9 (^{Ph}CH), 126.3 (C^{IV}), 127.6 (C^{IV}), 128.2 (^{L}*o*-CH), 128.5 (^{L}*m*-CH), 128.7 (^{Ph}CH), 129.0 (^{Ar*}*mes*-**C**H), 129.4 (C^{IV}), 130.1 (C^{IV}), 134.8 (C^{IV}), 136.3 (C^{IV}), 143.2 (C^{IV}), 143.5 (C^{IV}), 143.8 (C^{IV}). Anal. Calc. ($C_{53}H_{52}N_2$): C, 88.78; H, 7.31; N, 3.91. Found: C, 87.24; H, 7.31; N, 3.92.

L4: Ar*Ar*LH

2,6-Diphenylmethyl-4-methylphenyl-4-methylbenzamide (6.7 mmol, 3.5 g) and phosphorus pentachloride (8.0 mmol, 1.67 g) were dissolved in toluene (150 mL) under an inert atmosphere and heated at reflux overnight. The solvent was removed *in vacuo* and the reaction mixture was heated at 423 K for 1 hour. The resultant imidoyl chloride was dissolved in toluene (100 mL) and 2,6-diphenylmethyl-4-methylaniline (6.0 mmol, 2.64 g) was added. The reaction was heated at reflux for 96 h. Triethylamine (8.7 mmol, 1.21 mL) was added and the reaction was heated at reflux for a further 2 h. The product was solubilised in toluene, washed (1 x $NaHCO_3$, 2 x H_2O), dried ($MgSO_4$) and the solvent was removed *in vacuo.* The crude product was recrystallised from methanol/dichloromethane (3.35 g, 51%).

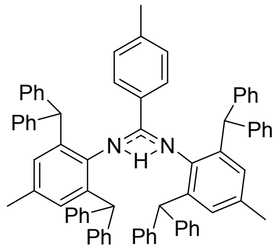

^{1}H NMR (500 MHz, C_6D_6, 298 K) δ (ppm): 1.88 (s, 6H, ^{Ar*}C**H**$_3$), 1.91 (s, 3H, C**H**$_3$), 5.25 (s, 1H, N**H**), 6.20 (d, 2H, ^{L}*o*-C**H**, $^{3}J_{HH}$ = 7 Hz), 6.36 (d, 4H, ^{Ph}*o*-C**H**, $^{3}J_{HH}$ = 22 Hz), 6.67 (d, 2H, ^{L}*m*-C**H**, $^{3}J_{HH}$ = 4.5 Hz); ^{13}C{^{1}H} NMR (125 MHz, C_6D_6, 298 K) δ (ppm): 21.2 (^{Ar*}**C**H), 51.7 (^{Ph}**C**H), 53.5 (^{Ph}**C**H), 125.7 (^{Ph}**C**H), 126.4 (^{Ph}**C**H), 128.9 (^{L}*o*-**C**H), 127.1 (^{L}*m*-**C**H), 129.3 (^{Ph}**C**H), 129.8 (^{Ph}**C**H), 130.1 (**C**^{IV}), 130.4 (**C**^{IV}), 134.7 (**C**^{IV}), 138.4 (**C**^{IV}), 143.1 (**C**^{IV}), 143.7 (**C**^{IV}), 144.1 (**C**^{IV}), 145.9 (**C**^{IV}). Anal. Calc. ($C_{74}H_{62}N_2$): C, 90.76; H, 6.38; N, 2.86. Found: C, 89.51; H, 6.43; N, 2.88.

3. Additional Data

Figure S1: through space hydride-phenyl distances

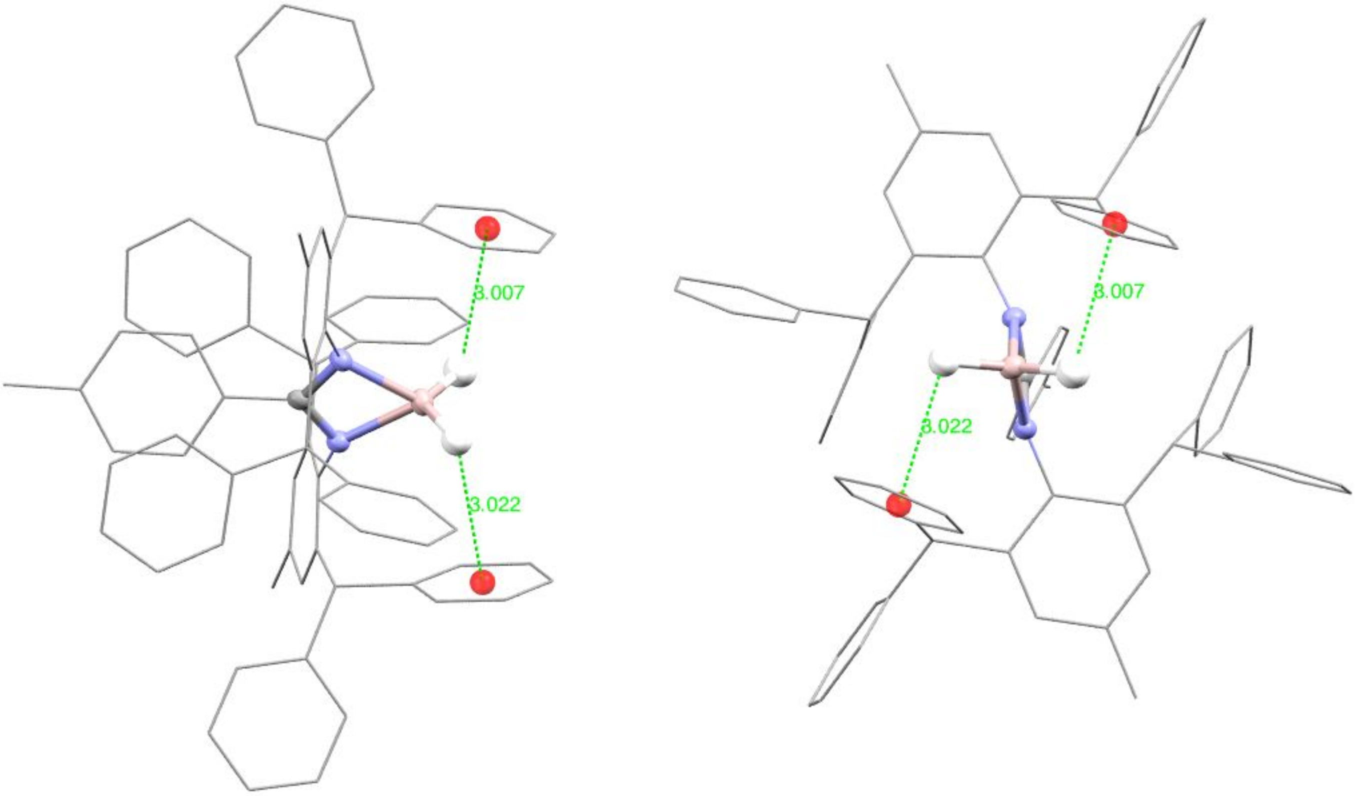

Table S1: Selected Bond Lengths (Å) and Angles (deg) for **2, 2', 2", 3, 4** and **5**.

	**2**	**3**	**4**	**2'**	**5**	**2"**
Al–N1	1.894(13)	1.961(15)	1.943(10)	2.081(10)	1.961(13)	1.899(9)
Al-N2	1.987(12)	1.941(14)	1.939(10)	1.950(10)	1.943(13)	2.126(9)
Al-N3	-	-	-	2.154(11)	-	-
Al–H	1.42 - 1.87	1.63(4), 1.75(4)	1.46(2)	1.47 - 1.54	-	1.46
C9-N1	1.346(18)	-	-	-	-	-
C9-N2	1.335(18)	-	-	-	-	-
C13–N1	-	1.324(2)	-	-	1.337(19)	-
C13-N2	-	1.348(2)	-	-	1.340(18)	-
C34-N1	-	-	1.340(14)	1.321(14)	-	-
C34-N2	-	-	1.340(15)	1.349(14)	-	-
C10-N1	-	-	-	-	-	1.352(14)
C10-N2						1.325(14)
Al-C54	-	-	-	-	1.942(17)	-
Al–C55	-	-	-	-	1.960(17)	-
N–C–N	108.4(12)	108.9(13)	109.0(10)	110.1(1)	109.9(13)	111.2(9)
N–Al–N	68.1(5)	67.7(6)	68.4(4)	65.66(4)	68.22(6)	66.25
H–Al–H	75.0(1)	106.0(1)	121.0(1)	123.0(1)	-	-
C–Al–C	-	-	-	-	117.0(8)	-

Figure S2: ^{1}H NMR spectrum of **4**, with Al-*H* highlighted; solvent peaks marked with asterisks.

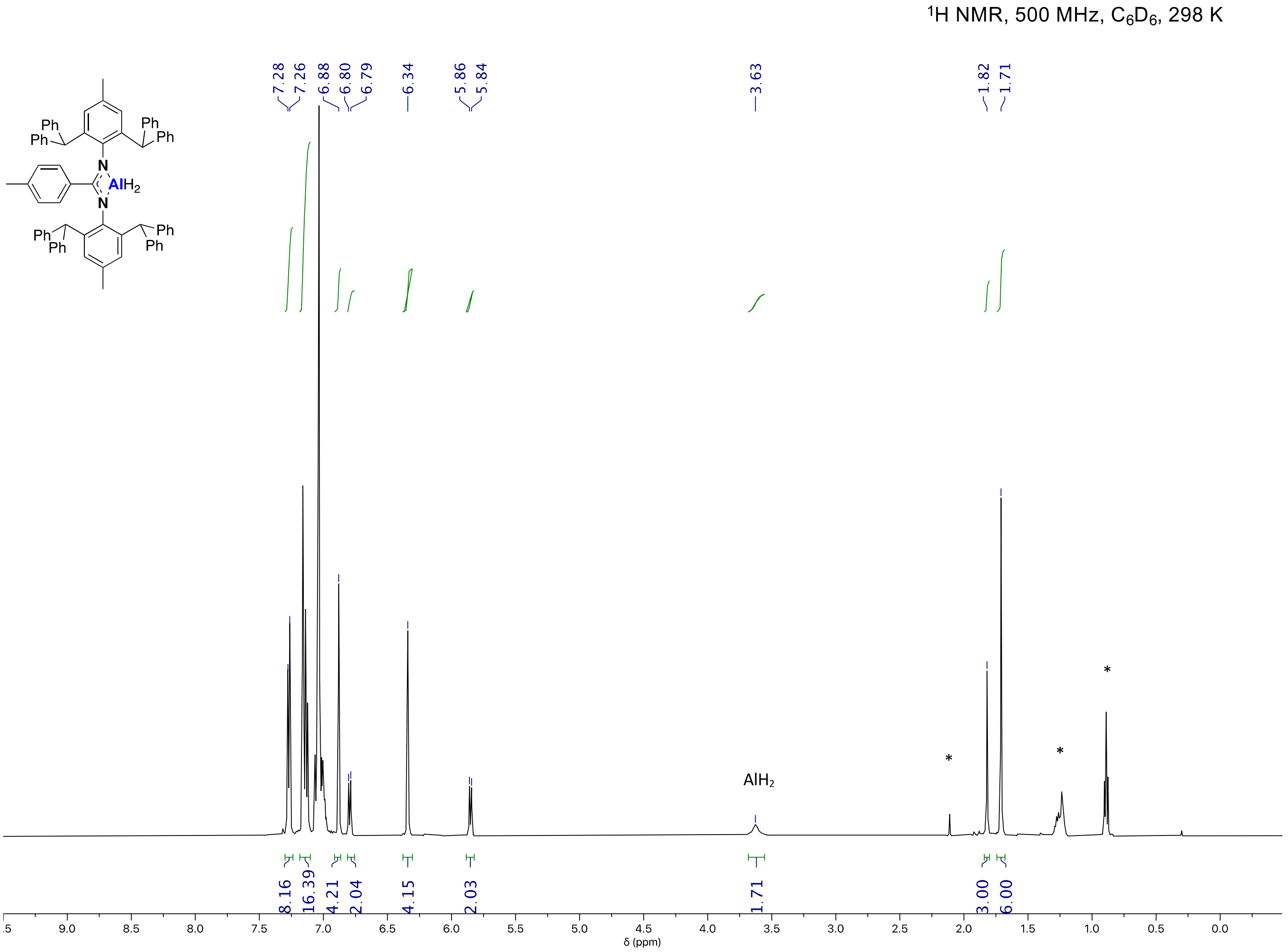

Table S2: Comparison of Al-H ^{1}H NMR shift, diffusion coefficients, hydrodynamic radii calculated and calculated volume assuming a sphere for **1-6** and **2'**.

Compound	Al–H ^{1}H NMR shift	Diffusion Coefficient (10^{-10})	Hydrodynamic Radius (Å)	Volume (Å)
**1**	5.06	7.3	5.0	520
**2**	5.01	4.8	7.6	1840
**3**	4.77	5.6	6.5	1150
**4**	3.63	4.9	7.4	1700
**2'**	4.54	5.4	6.7	1250
**5**	-	6.3	5.8	820
**6**	-	5.3	6.8	1320

Figure S3: Catalytic cycles to show hydroboration *via* an acetylide pathway (left) or hydroalumination (right)

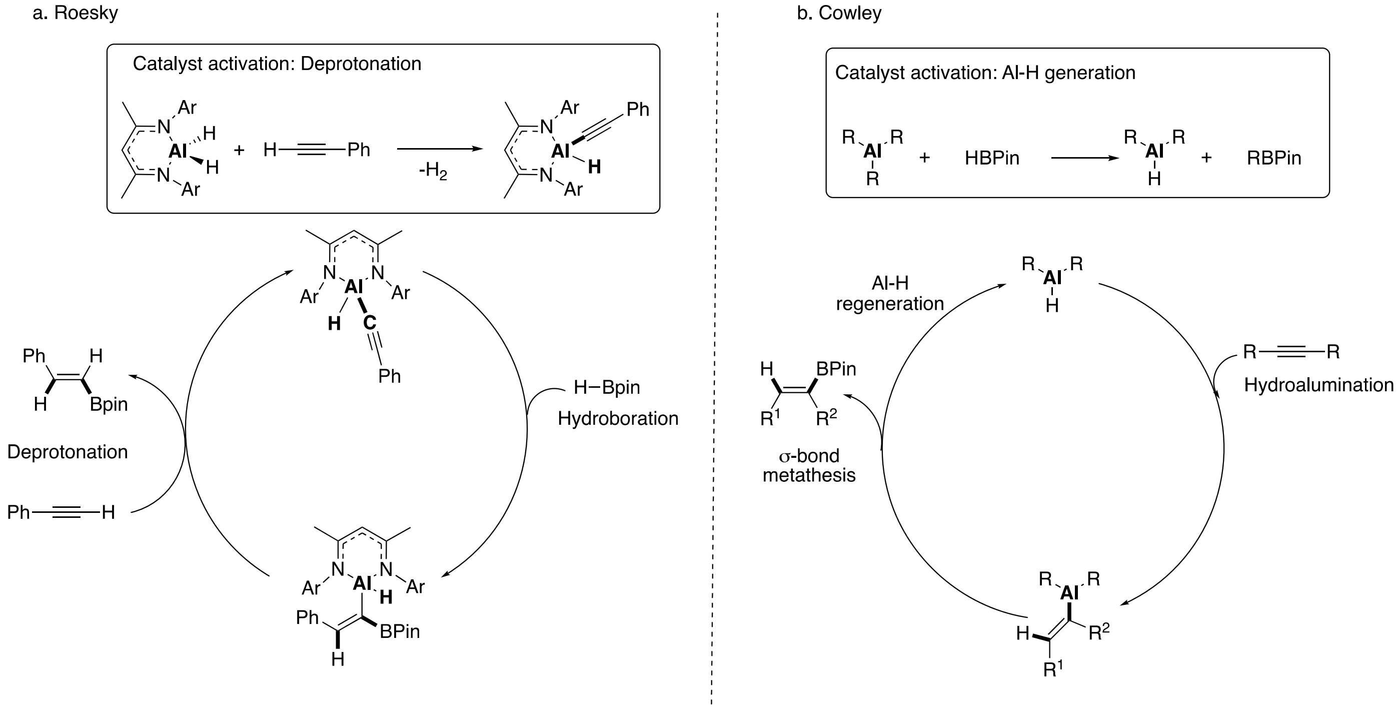

Figure S4: ^{1}H NMR stack of control reaction of HBpin + phenylacetylene heated in C_6D_6 in the absence of catalyst

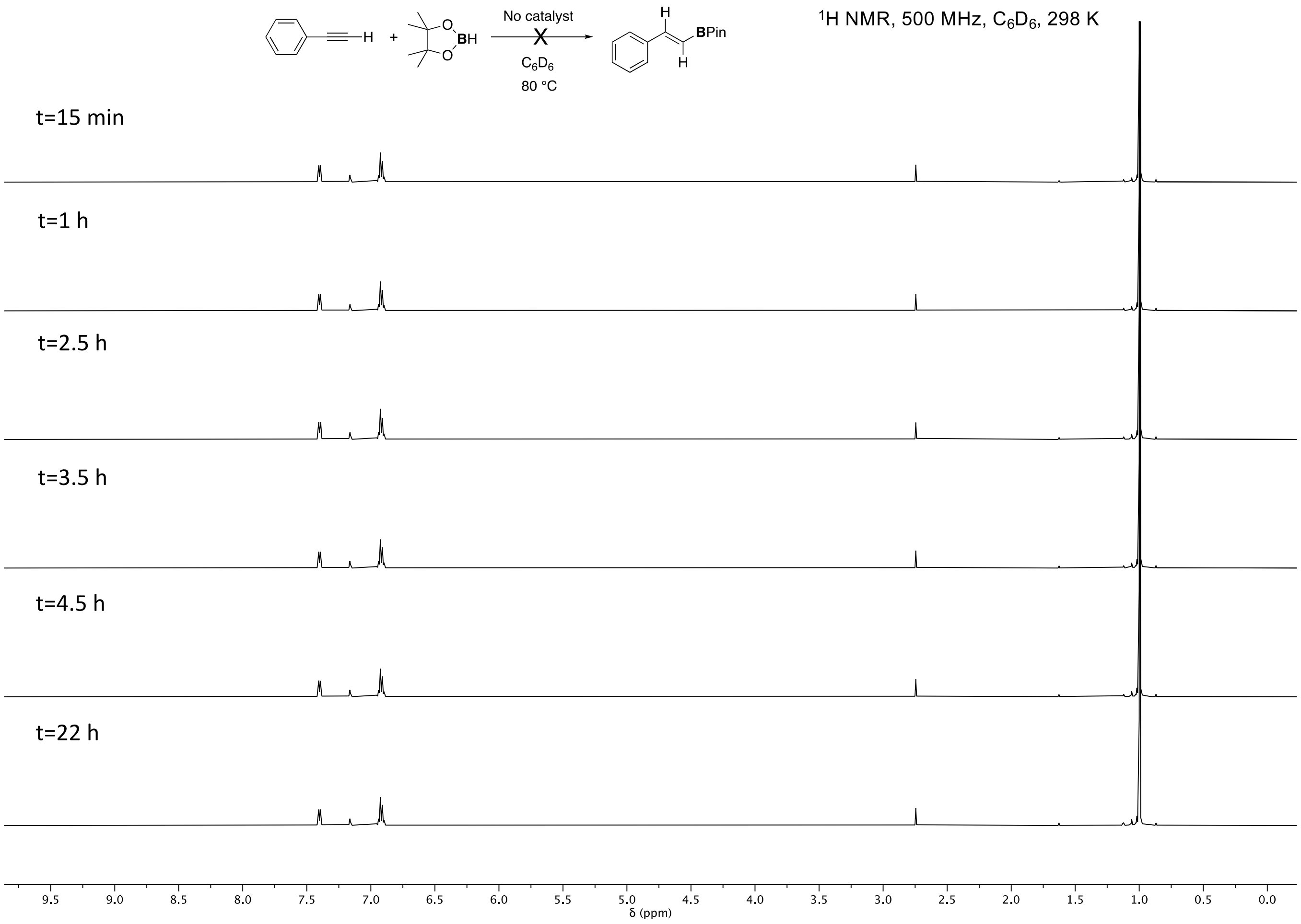

Figure S5: ^{11}B NMR stack of control reaction of HBpin + phenylacetylene heated in C_6D_6 in the absence of catalyst

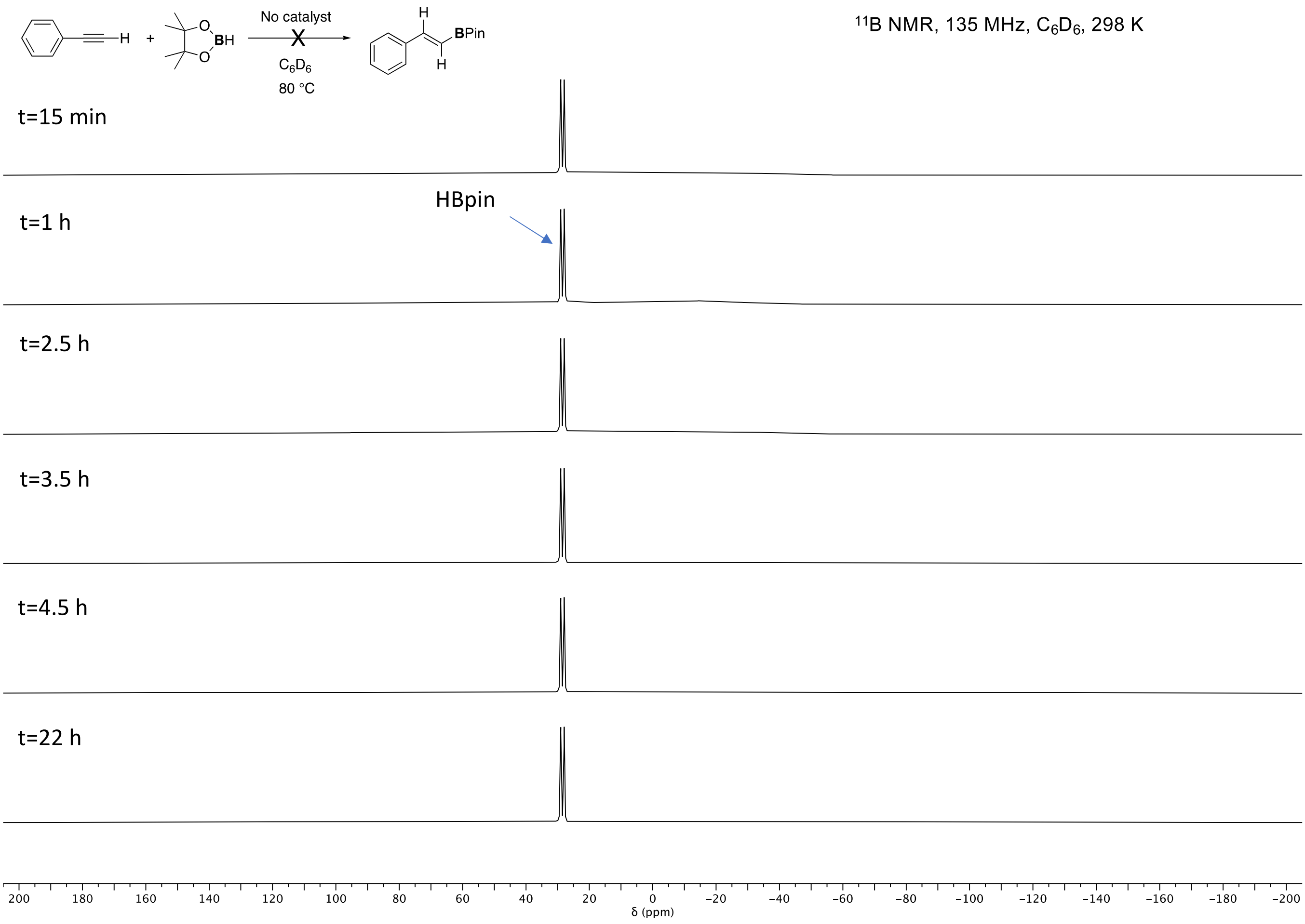

Figure S6: ^{1}H NMR stack of **3** + PhCCH; solvent peaks marked with asterisks.

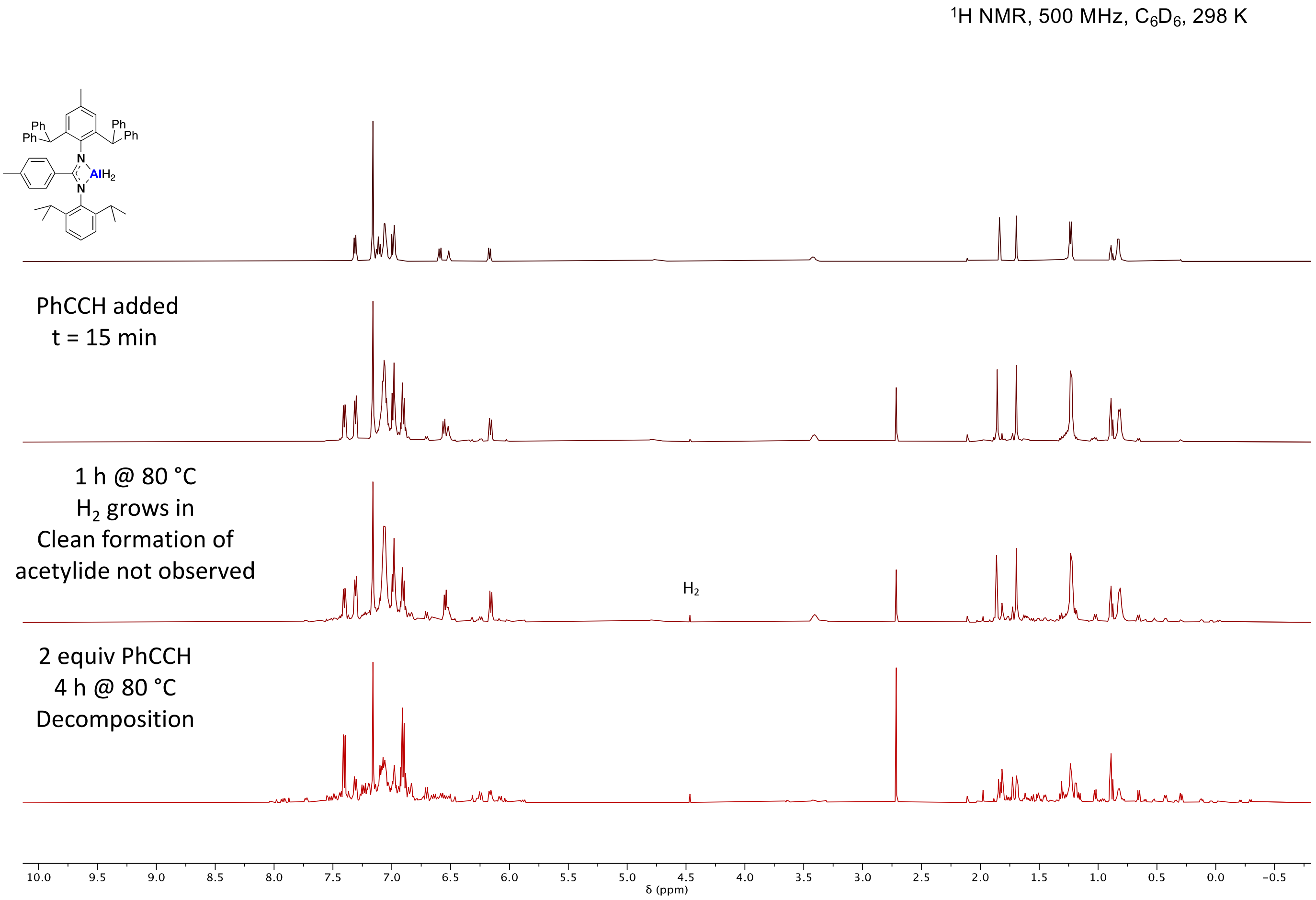

Figure S7: ^{1}H NMR stack of **4** + PhCCH @ 353 K

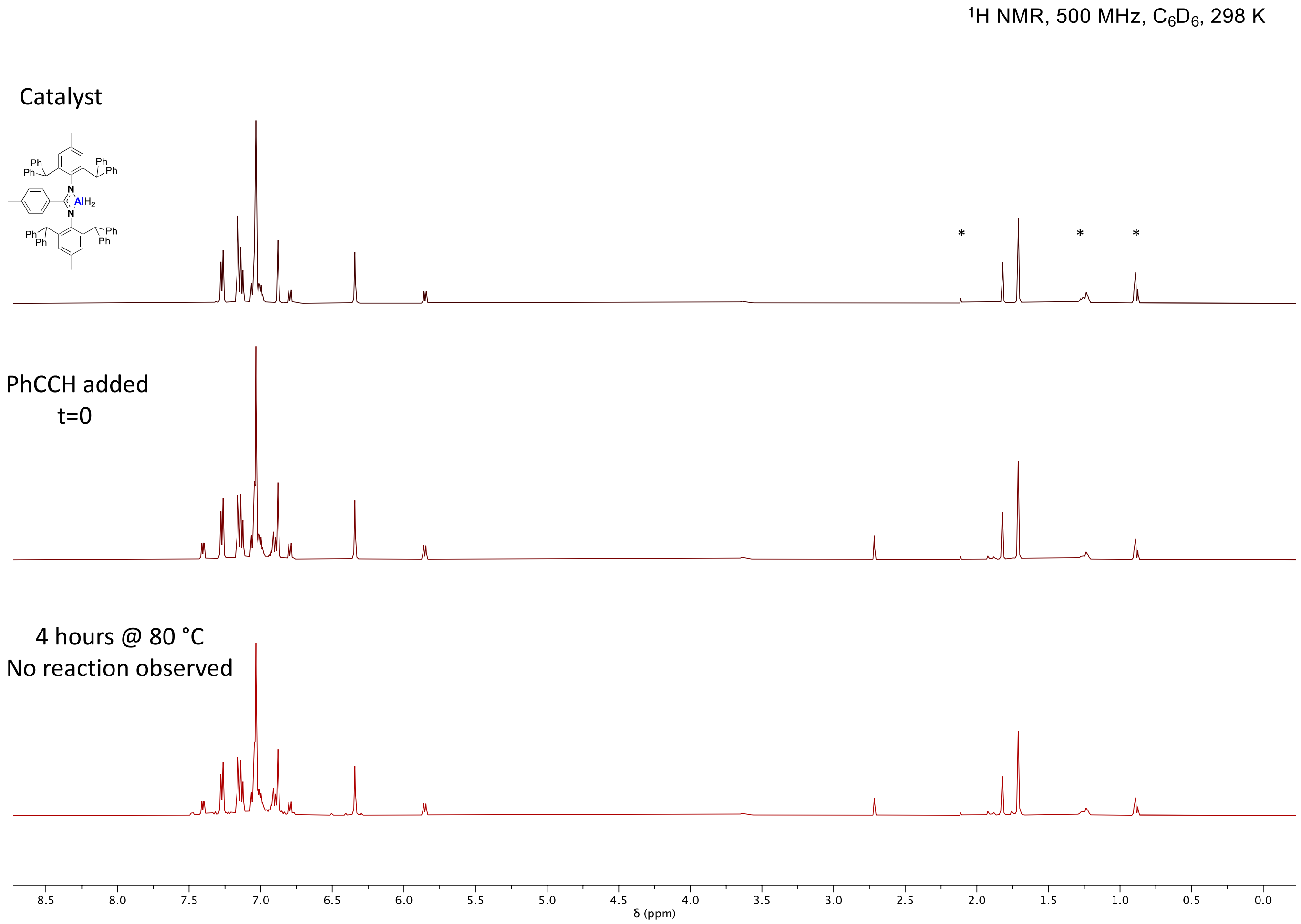

Figure S8: ^{1}H NMR stack of **4** + PhCCH @ 298 K

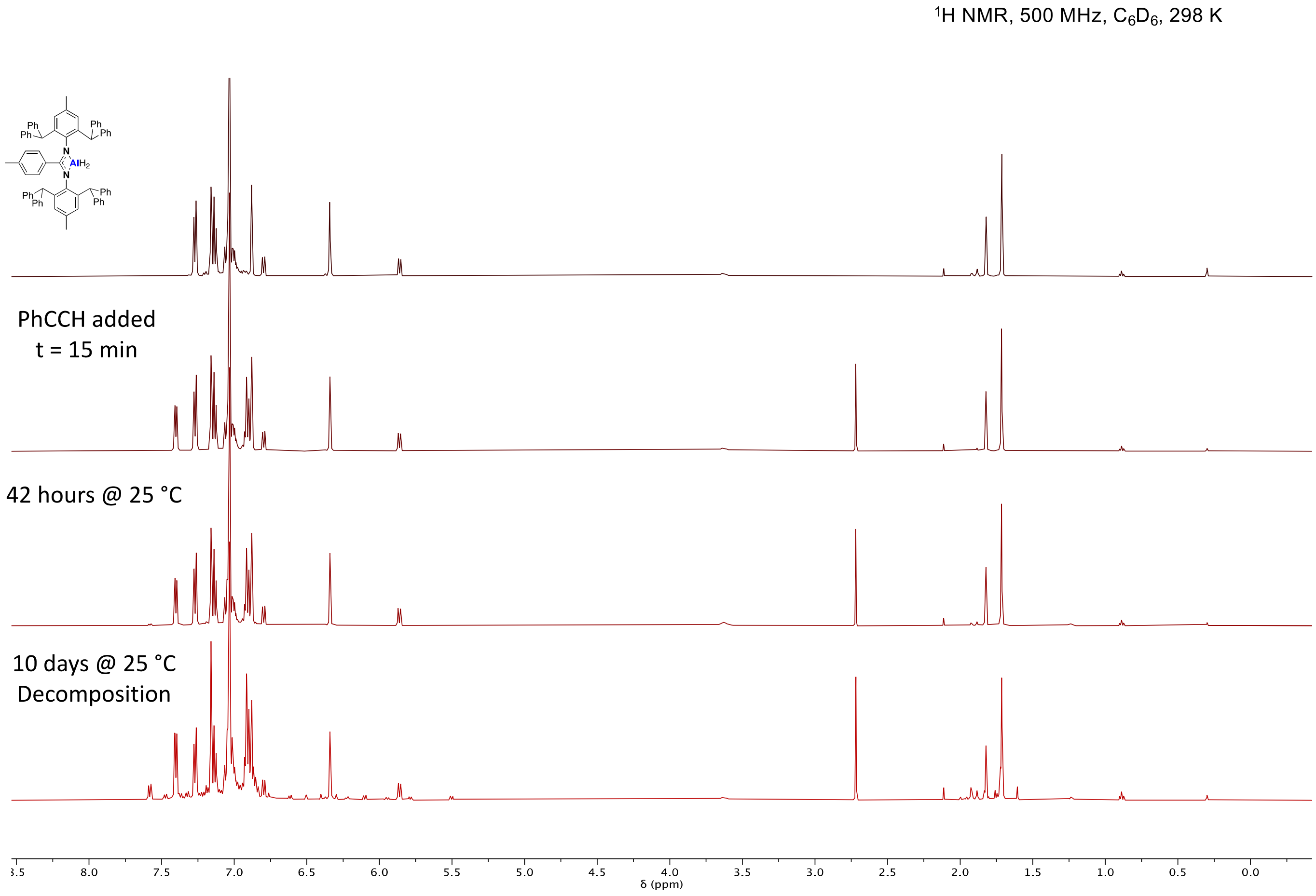

Figure S9: ^{1}H NMR stack of **4** + HBpin

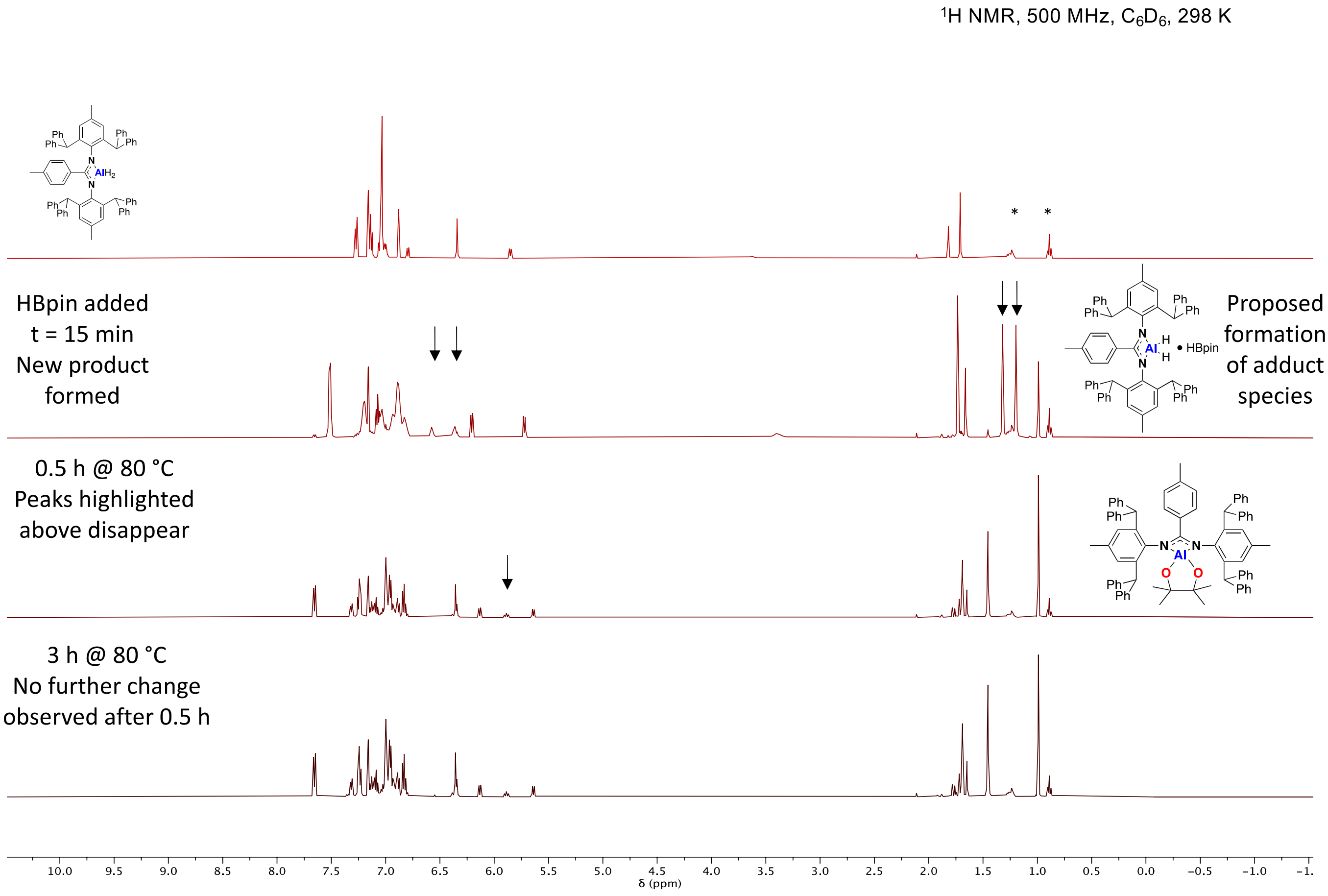

Figure S10: ^{1}H NMR of **4** + HBpin t=15 min showing formation of **4•HBpin**, expanded from figure above

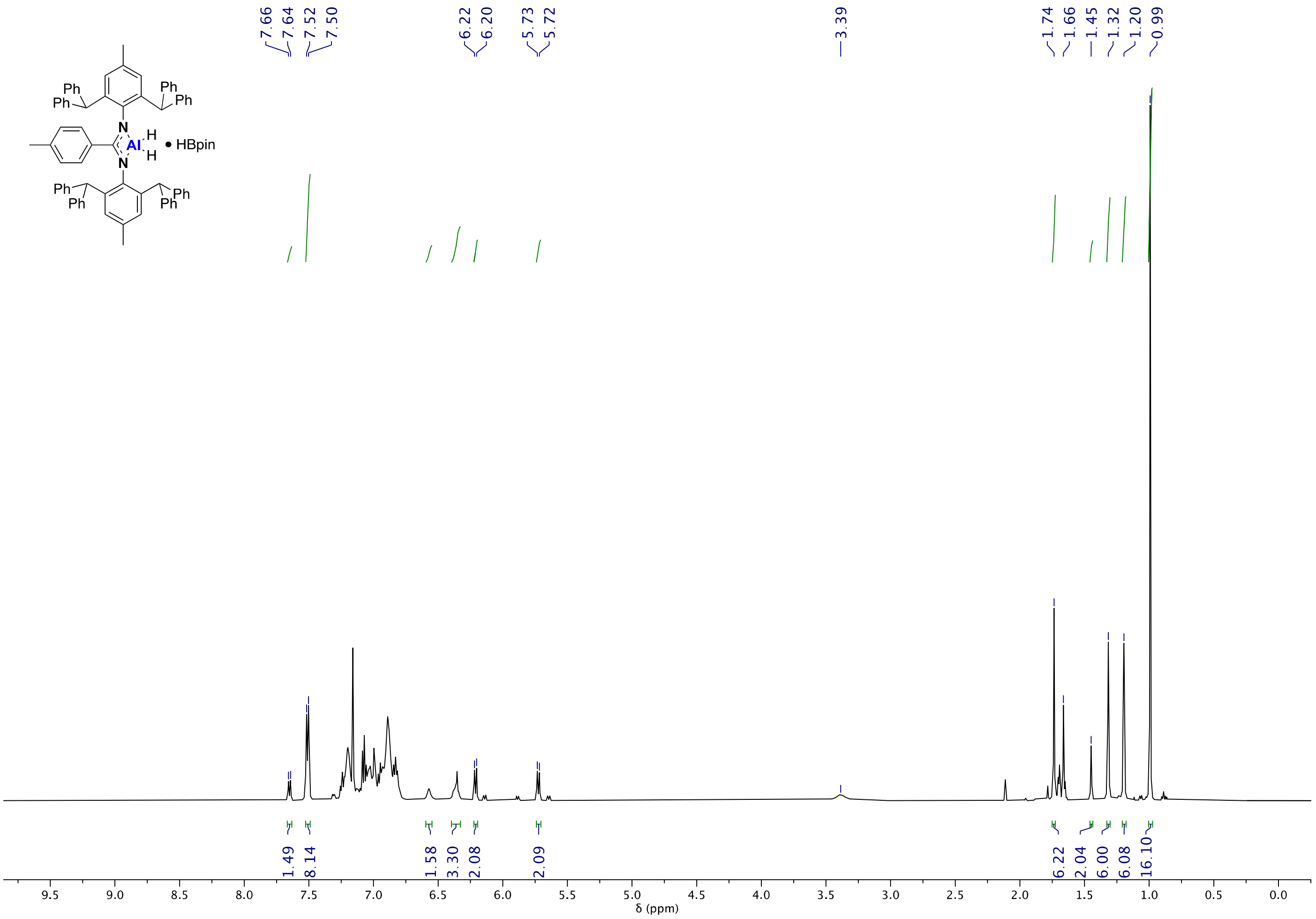

Figure S11: ^{11}B NMR spectrum of **4** + HBpin t=15 min showing the proposed **4•HBpin** adduct

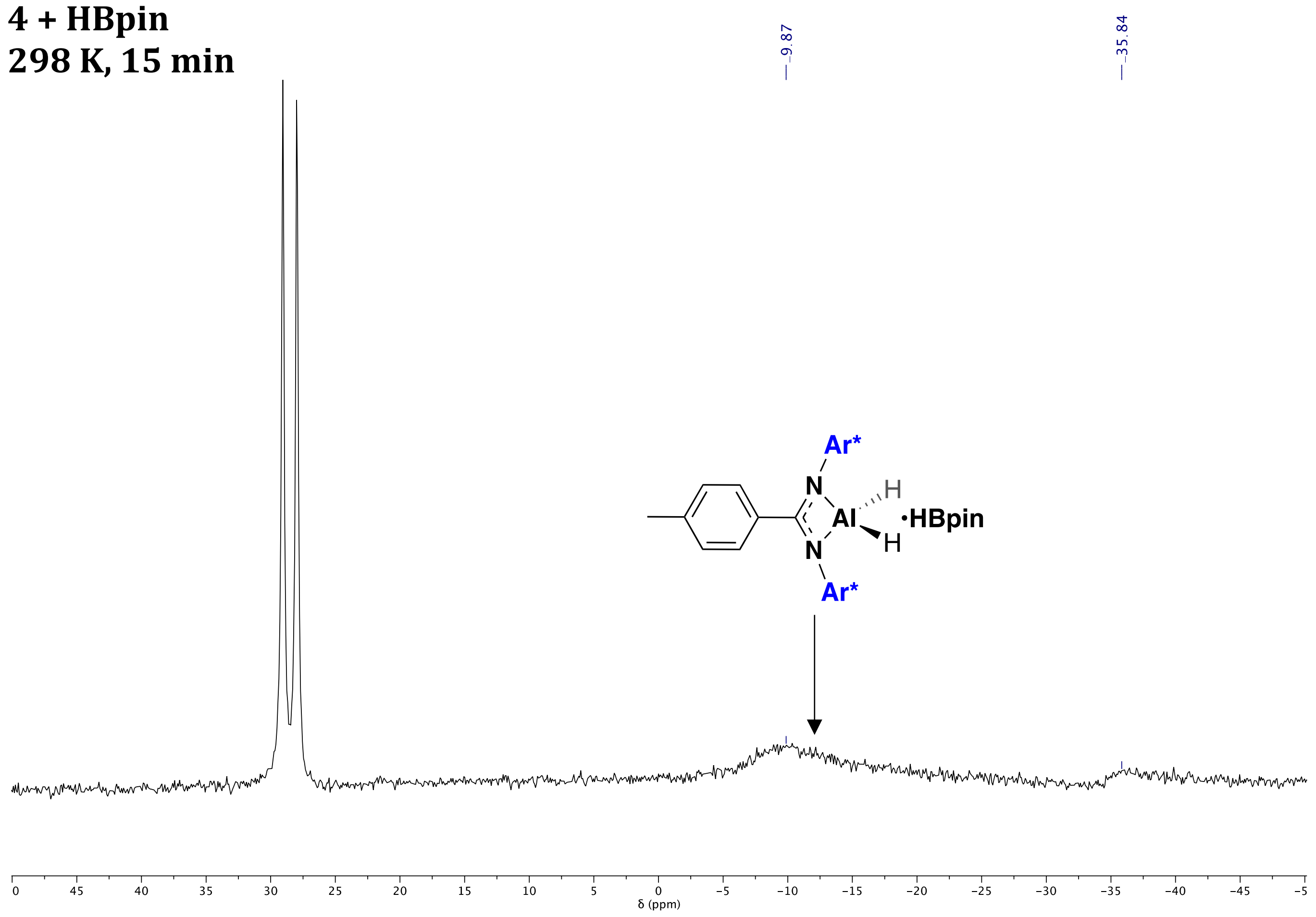

Figure S12: ^{11}B NMR stack of species present in reaction of **4** + HBpin after 15 min (top) and after heating for 30 mins at 80 °C (bottom)

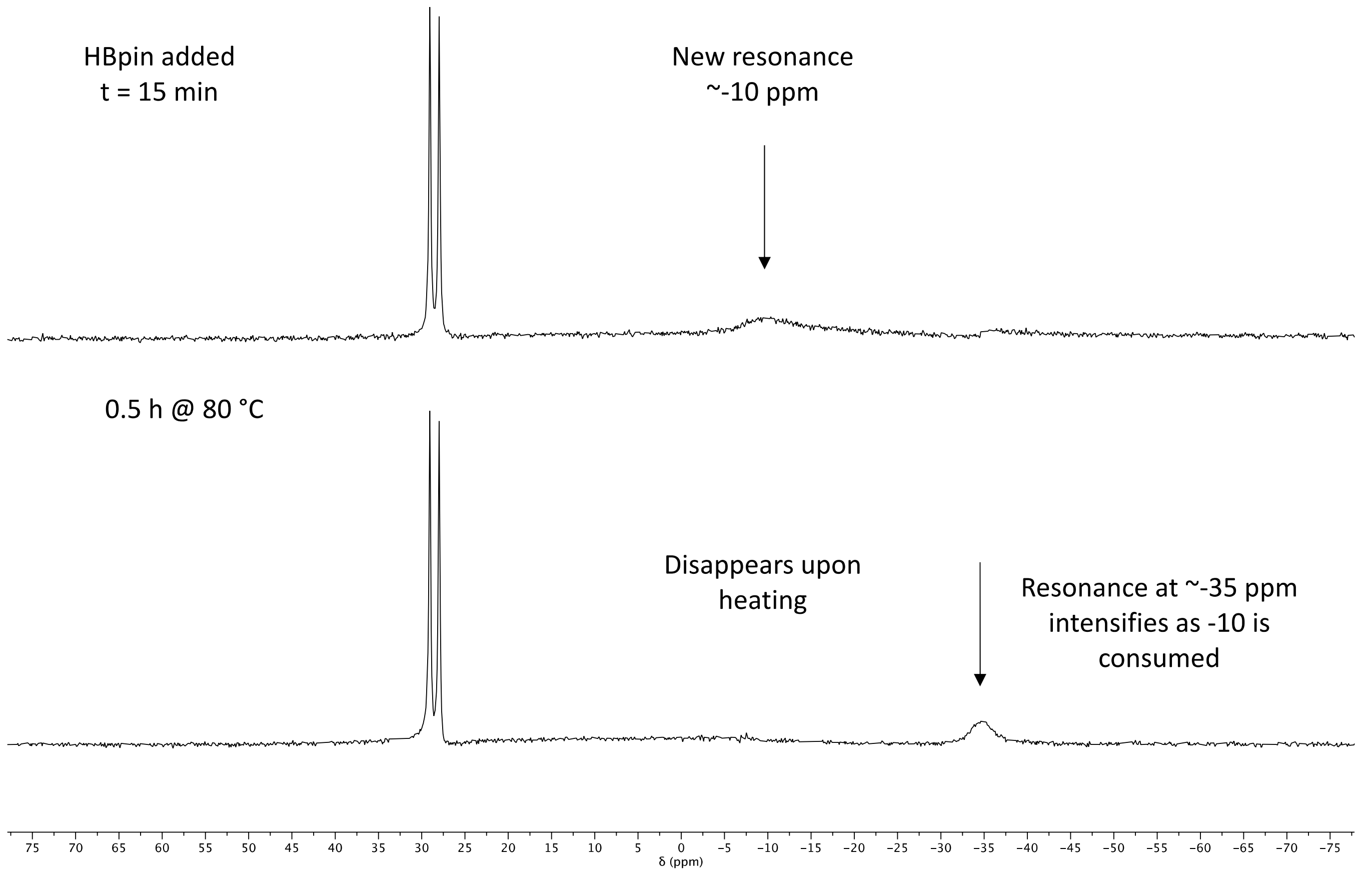

These ^{11}B NMR spectra were recorded at the same time as the middle two spectra in Figure S7 (t=15 min, t=0.5 h @ 80 °C). As the two new singlets at 1.20/1.32 ppm appear, so does a broad resonance at -10 ppm. When heated, these resonances in both the ^{1}H and ^{11}B NMR spectra disappear upon formation of **7**, suggesting these are from the same structure and likely due to the **4•HBpin** adduct.

Figure S13: ^{1}H NMR stack of **3** + HBpin

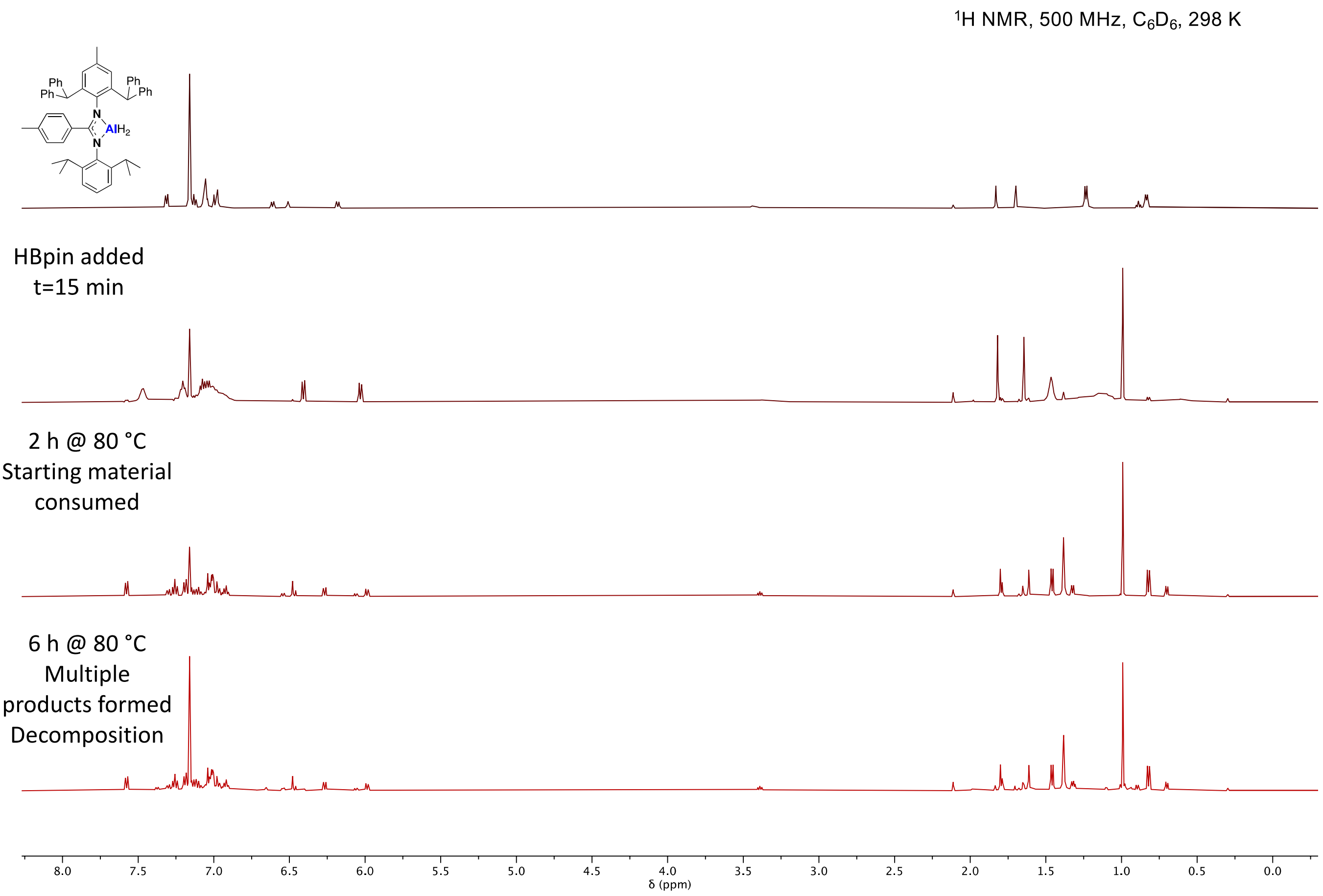

Addition of HBpin to **3** showed an immediate shift in the two singlet resonances of the starting material (1.70/1.85 to 1.65/1.82 ppm) and the disappearance of the Al-*H* resonance. This is proposed to be the pinacolate structure **3•HBpin** analogous to that of **4**. Heating for 2 hours shows the formation of a major and minor product present in a 1:0.4 ratio, with the minor product proposed to be the pinacolate. A new singlet at 1.37 ppm is assigned to the methyl protons of the pinacolate. The ^{11}B NMR spectrum also showed resonances at 28 and -35 ppm as was observed for the reaction of **4** + HBpin.

Figure S14: ^{11}B NMR spectra of species present in the reaction of **3** + HBpin

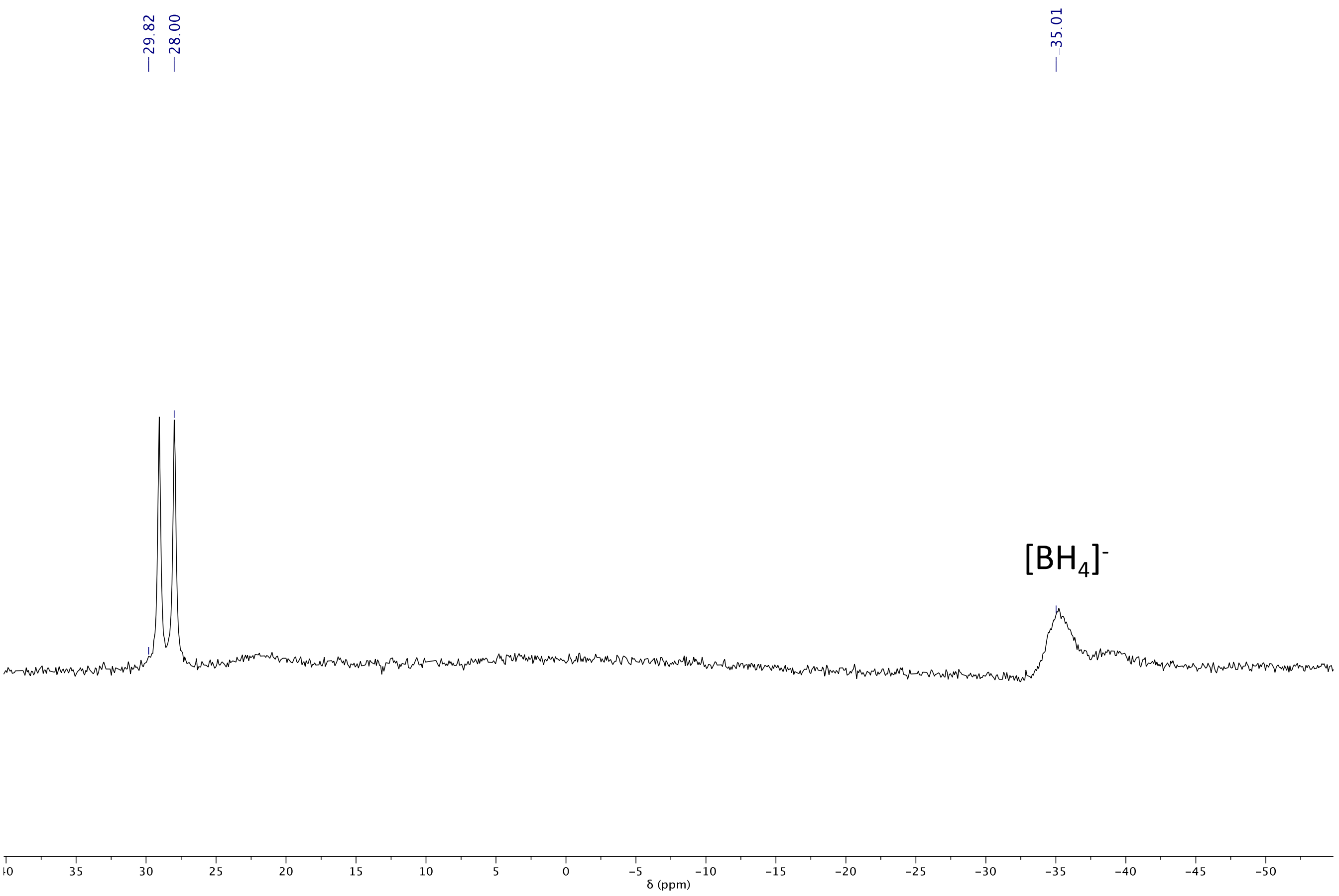

Figure S15: ^{1}H NMR stack of **2** + PhCCH

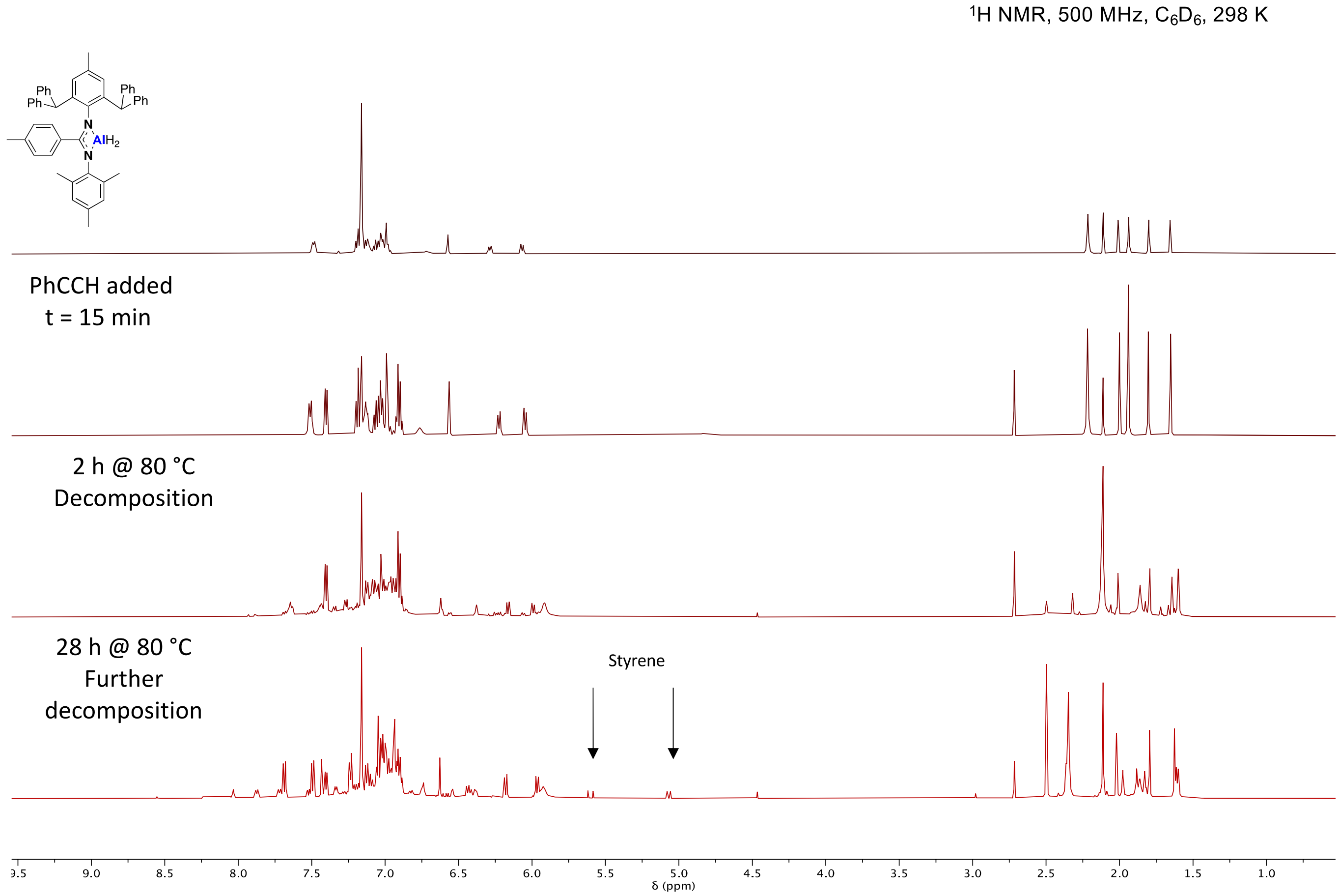

Figure S16: ^1H NMR stack of **2** + HBpin

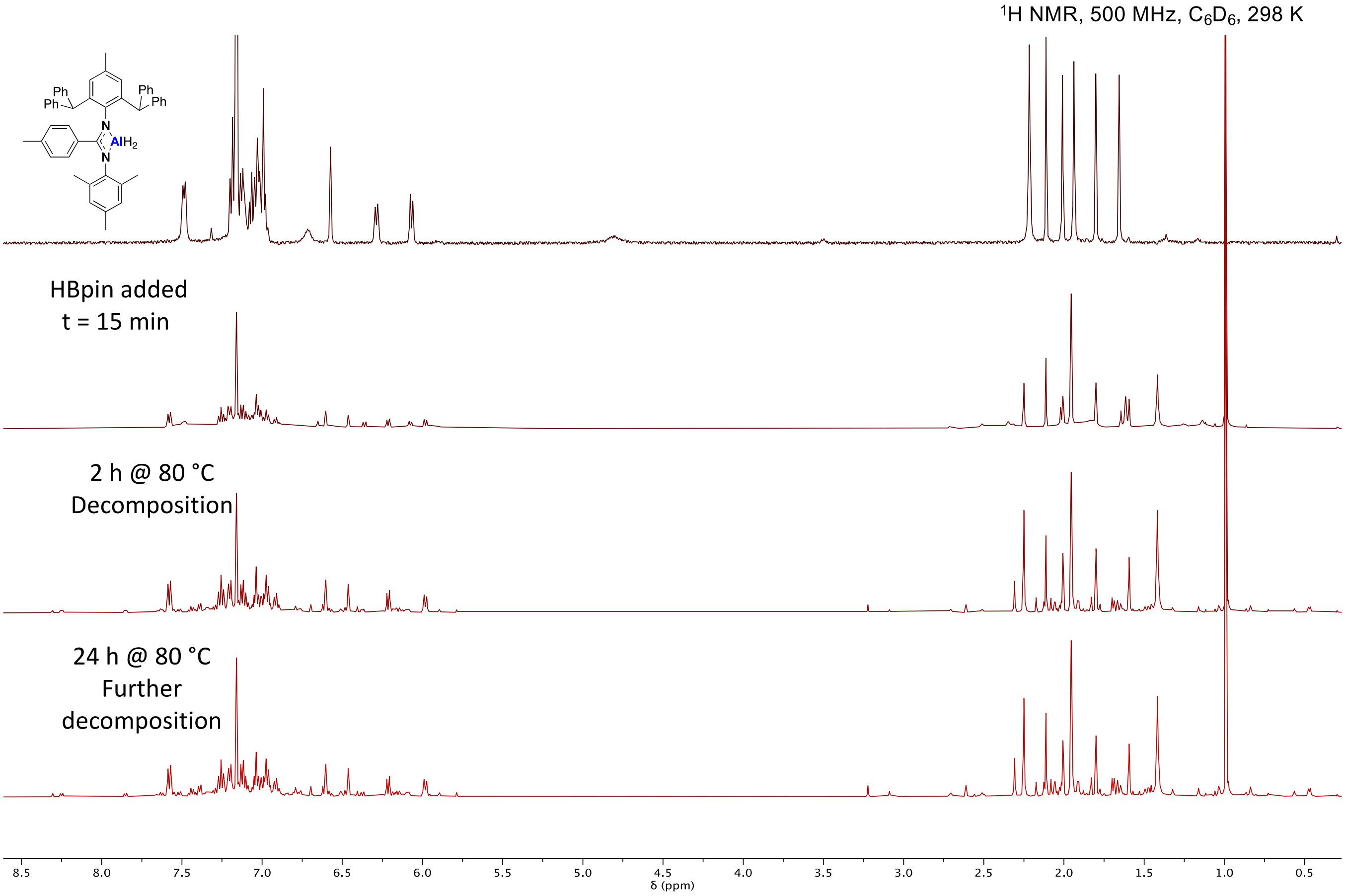

Figure S17: ^{11}B NMR of species present in the reaction of **2** + HBpin

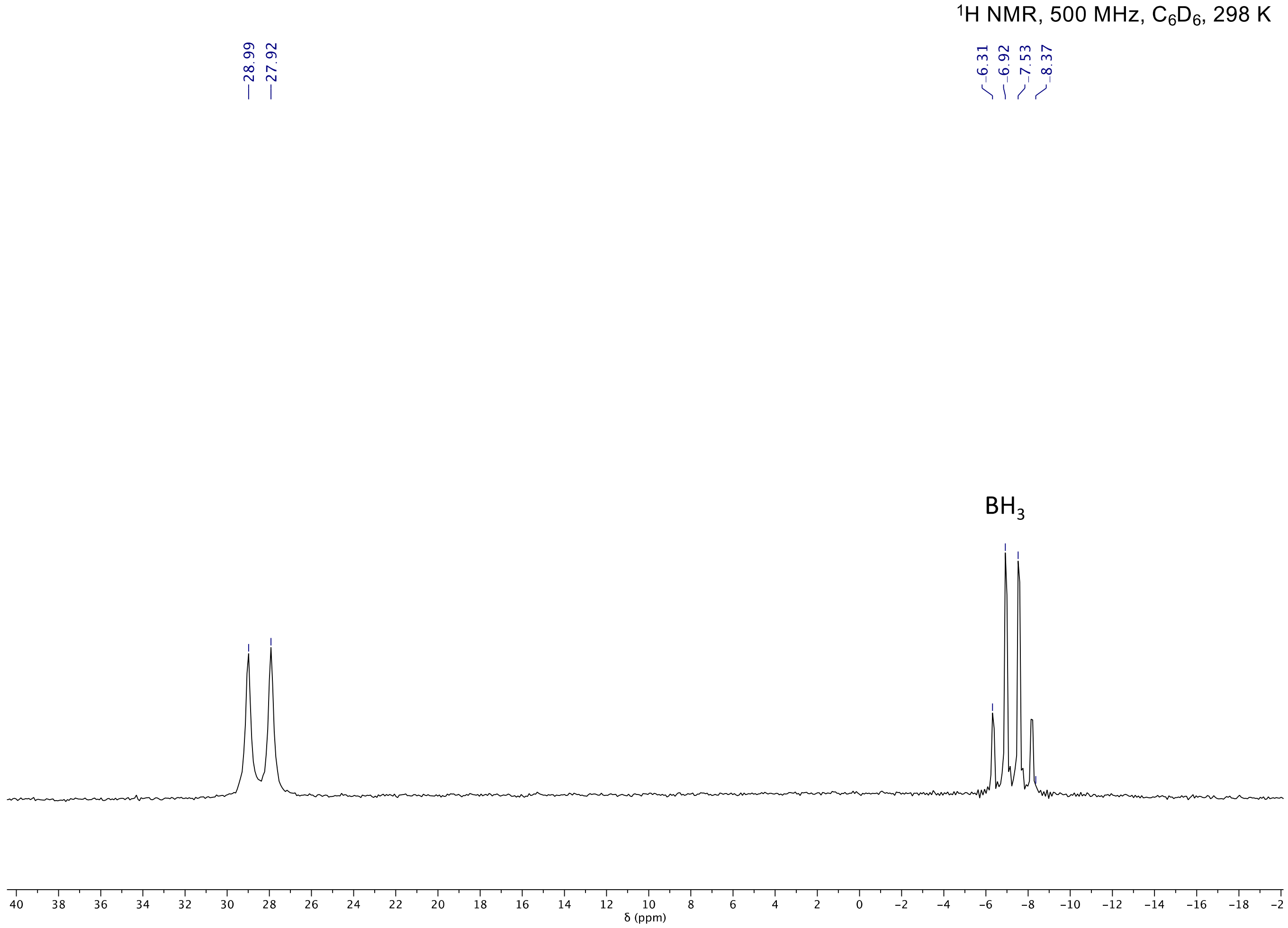

Figure S18: ^{1}H NMR stack of **8** + PhCCH in C_6D_6.

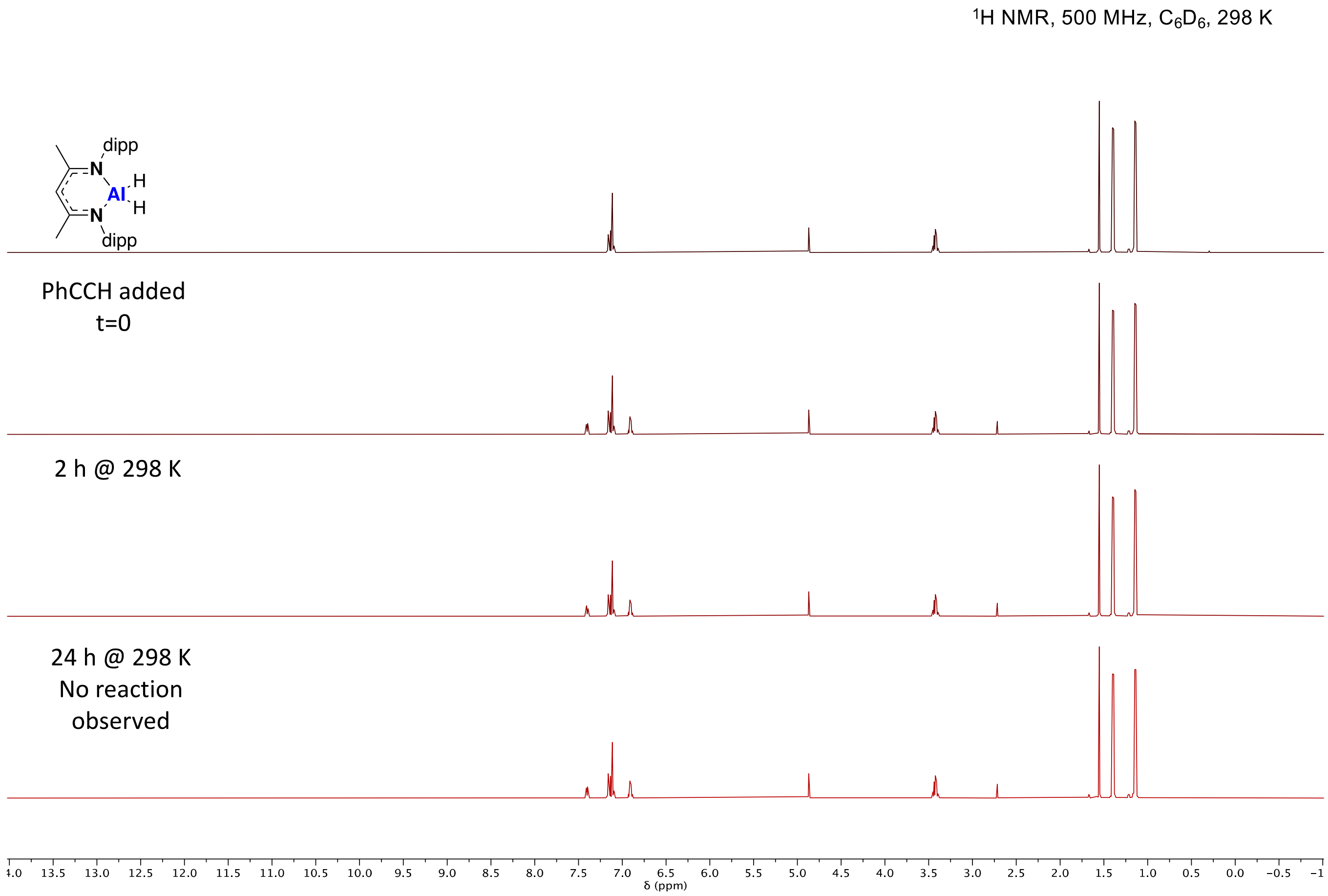

Figure S19: ^1H NMR stack of **8** + PhCCH in $CDCl_3$

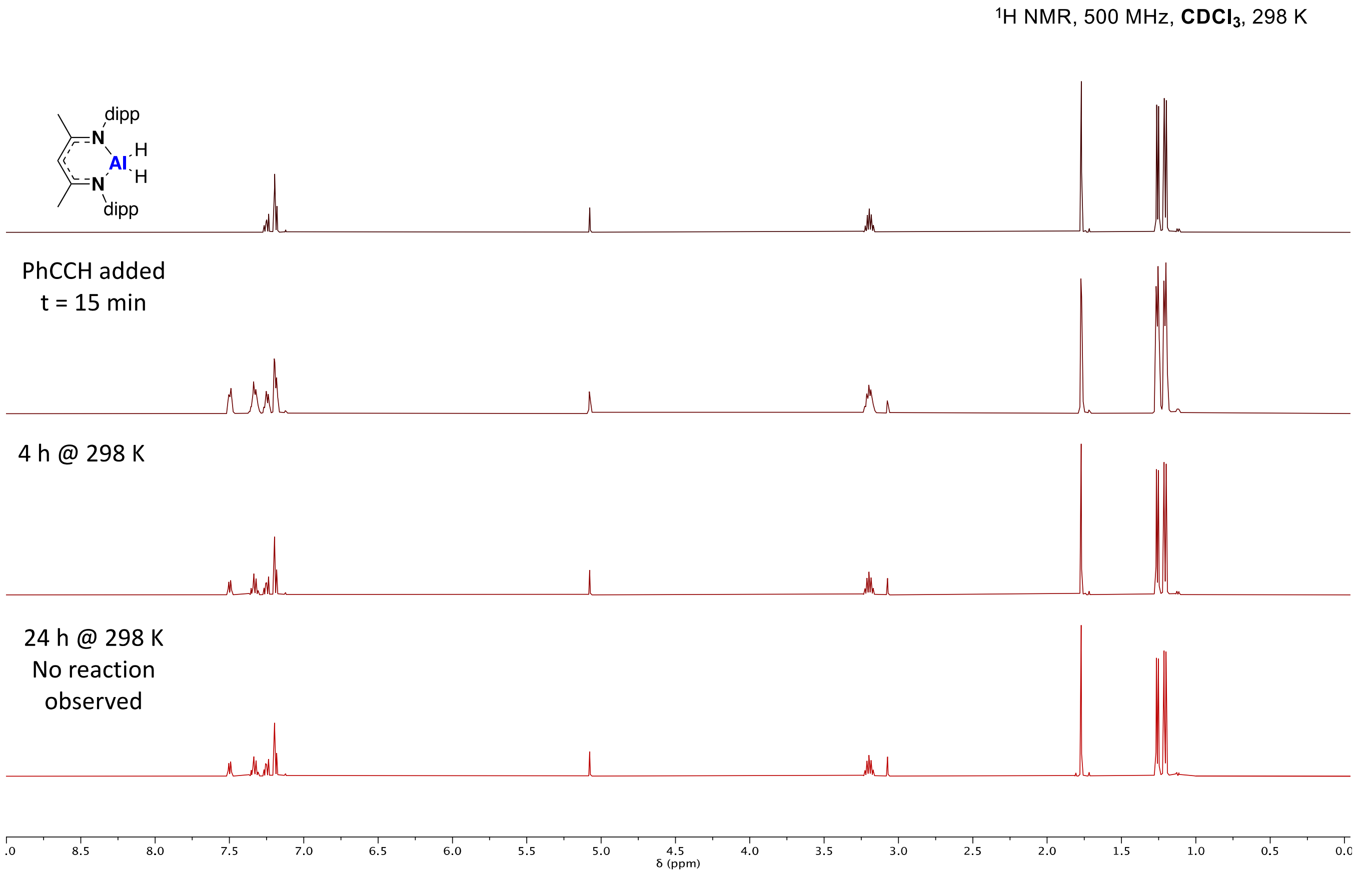

Figure S20: ^{1}H NMR stack of **8** + HBpin in C_6D_6

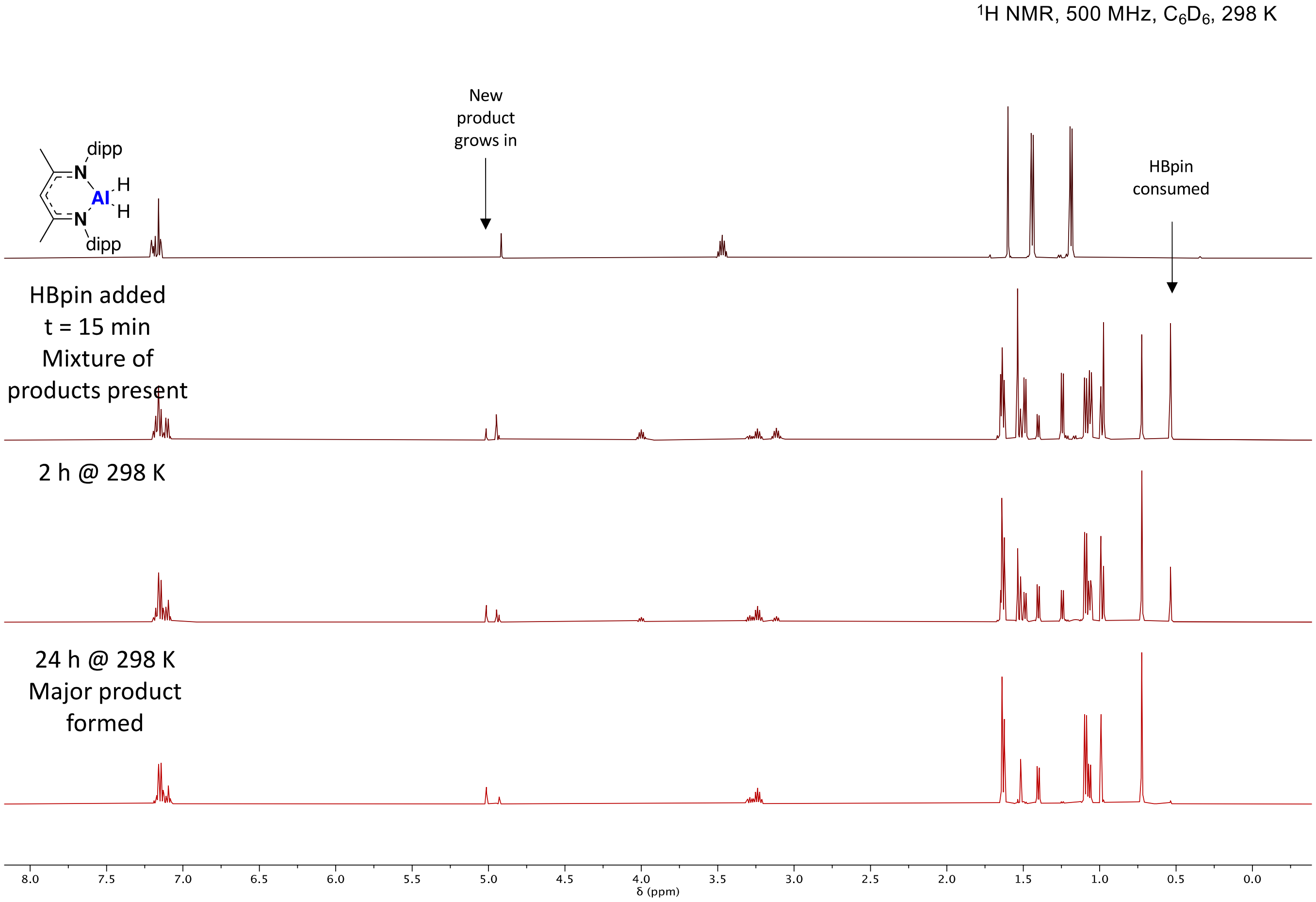

Figure S21: ^{11}B NMR of species present in the rection of **8** + HBpin

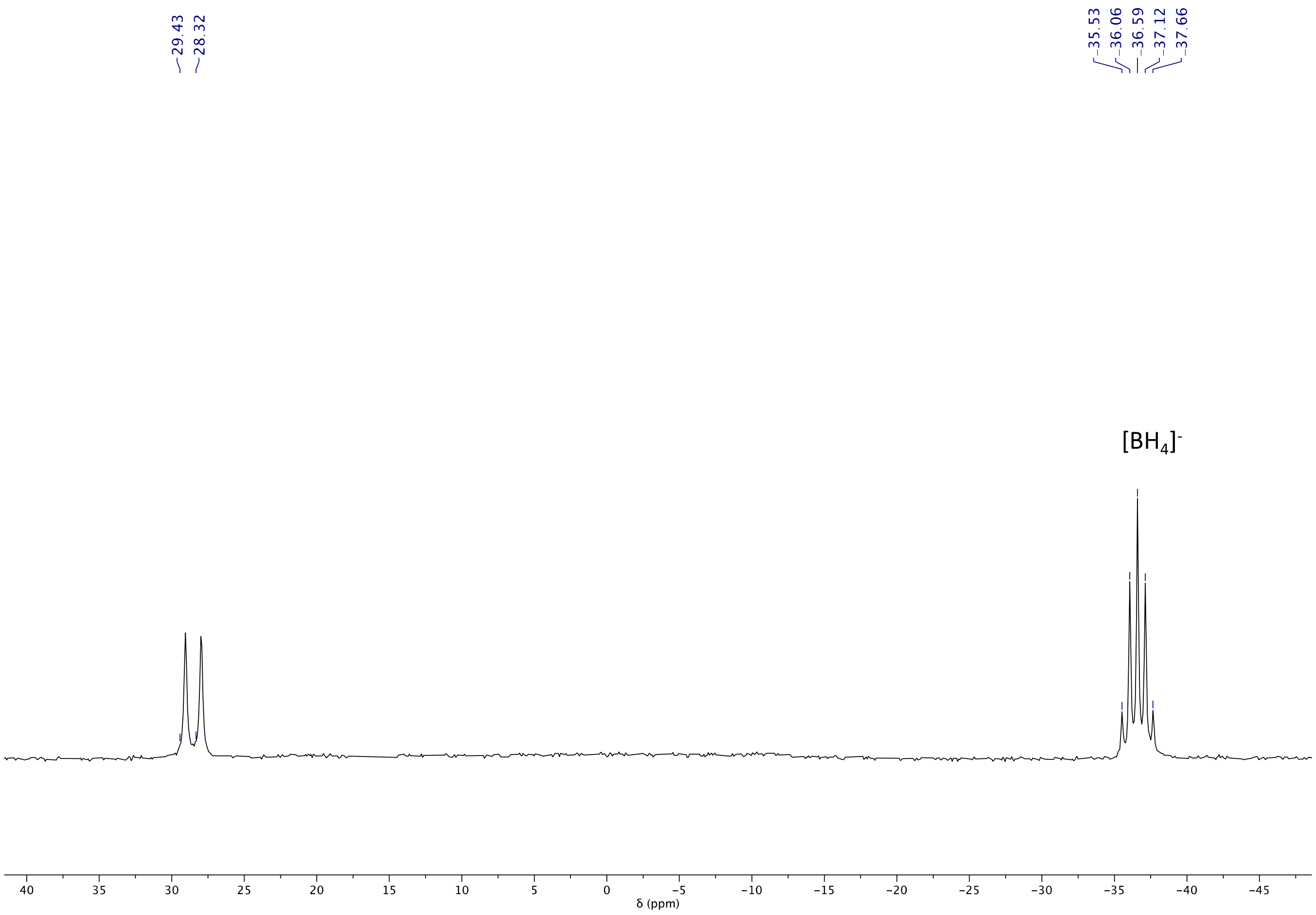

Figure S22: ^{1}H NMR stack of **8** + HBpin in $CDCl_3$

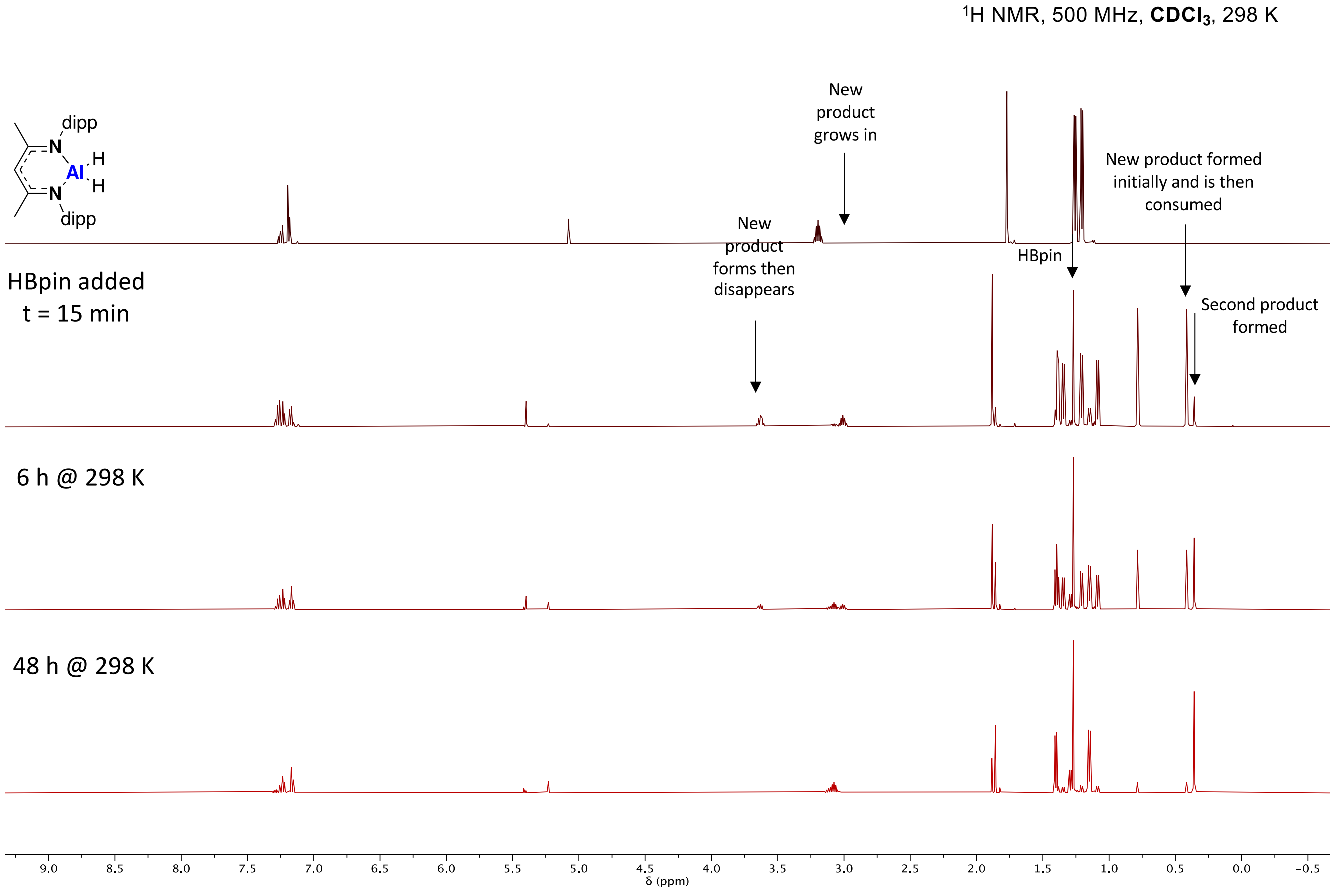

Figure S23: ^{1}H NMR stack of **5** + PhCCH

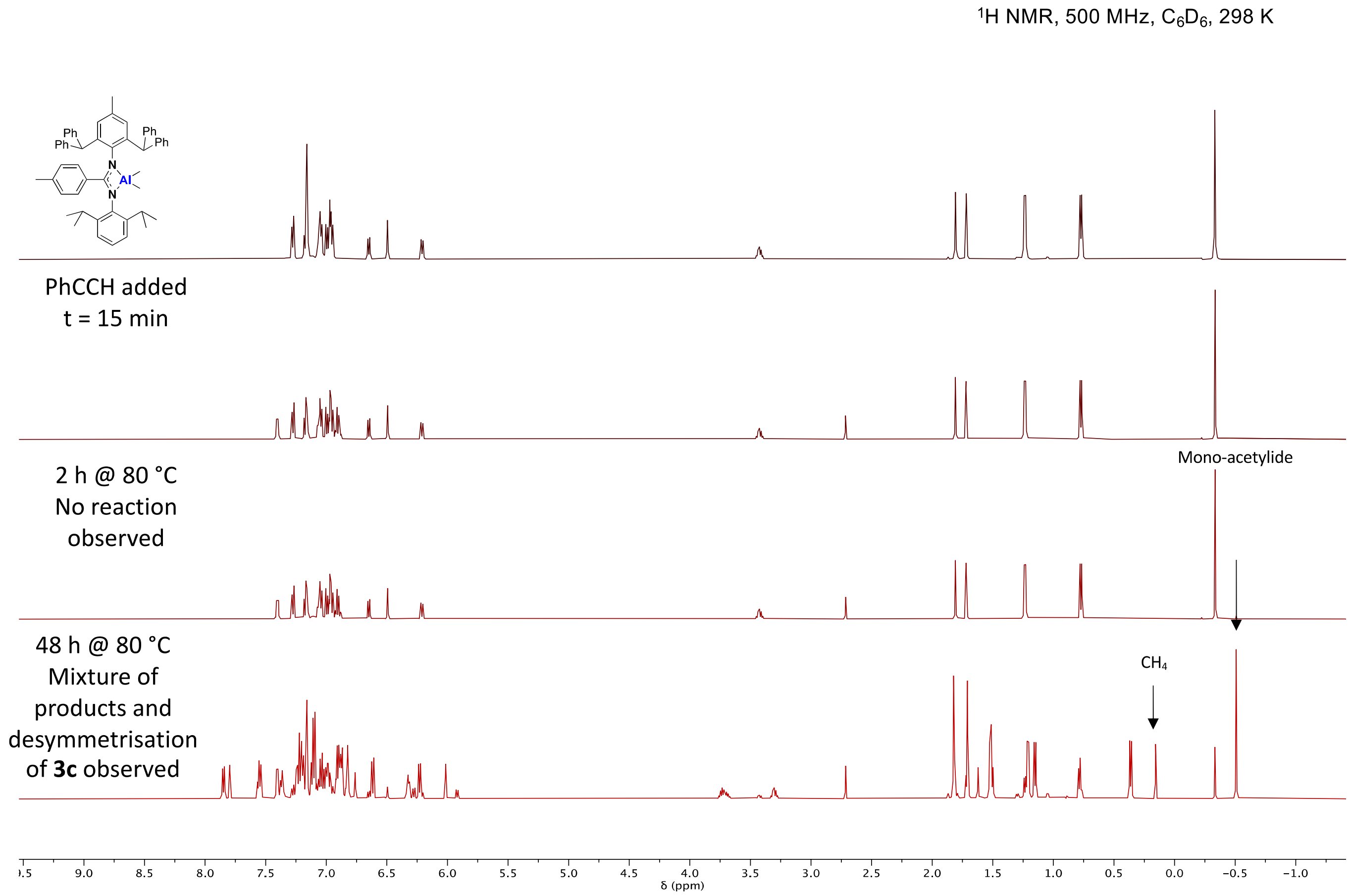

Figure S24: ^{1}H NMR spectrum of the reaction of **5** + PhCCH after 48 h

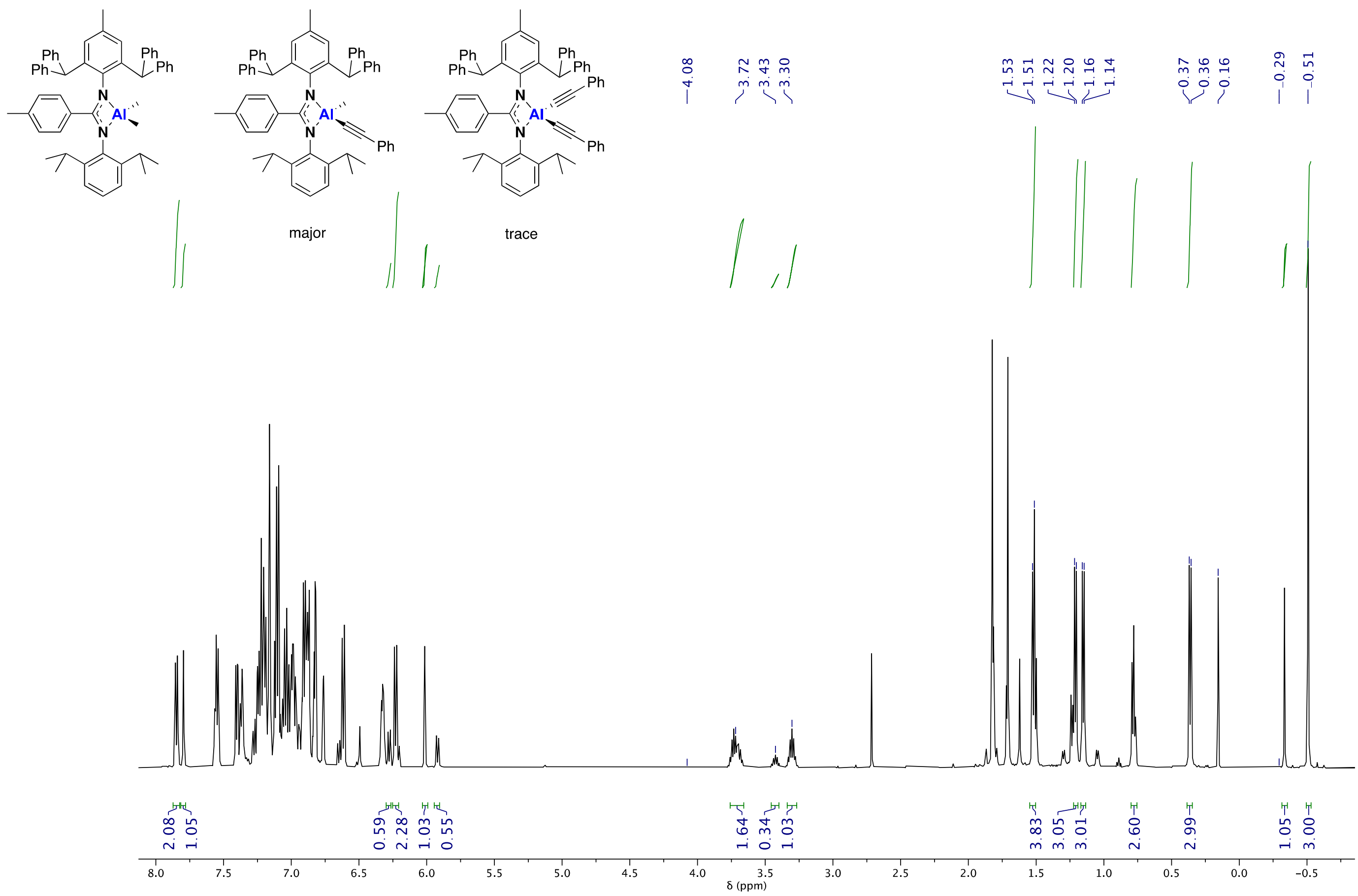

After 48 hours at 80 °C, the ^{1}H NMR spectrum showed a mixture of starting material (approx. 17%), the mono-acetylide complex (major product) and trace amounts of the bis-acetylide. Whilst no signal below 0 ppm would be observed for this bis-acetylide complex, the splitting pattern of the multiplet corresponding to methine protons at 3.30 ppm suggests a third species is present. The formation of methane (0.16 ppm) supports the formation of these complexes. Signals at -0.29 and 3.43 ppm support the presence of **5** in the reaction mixture.

Figure S25: ^{1}H NMR stack of **6** + PhCCH

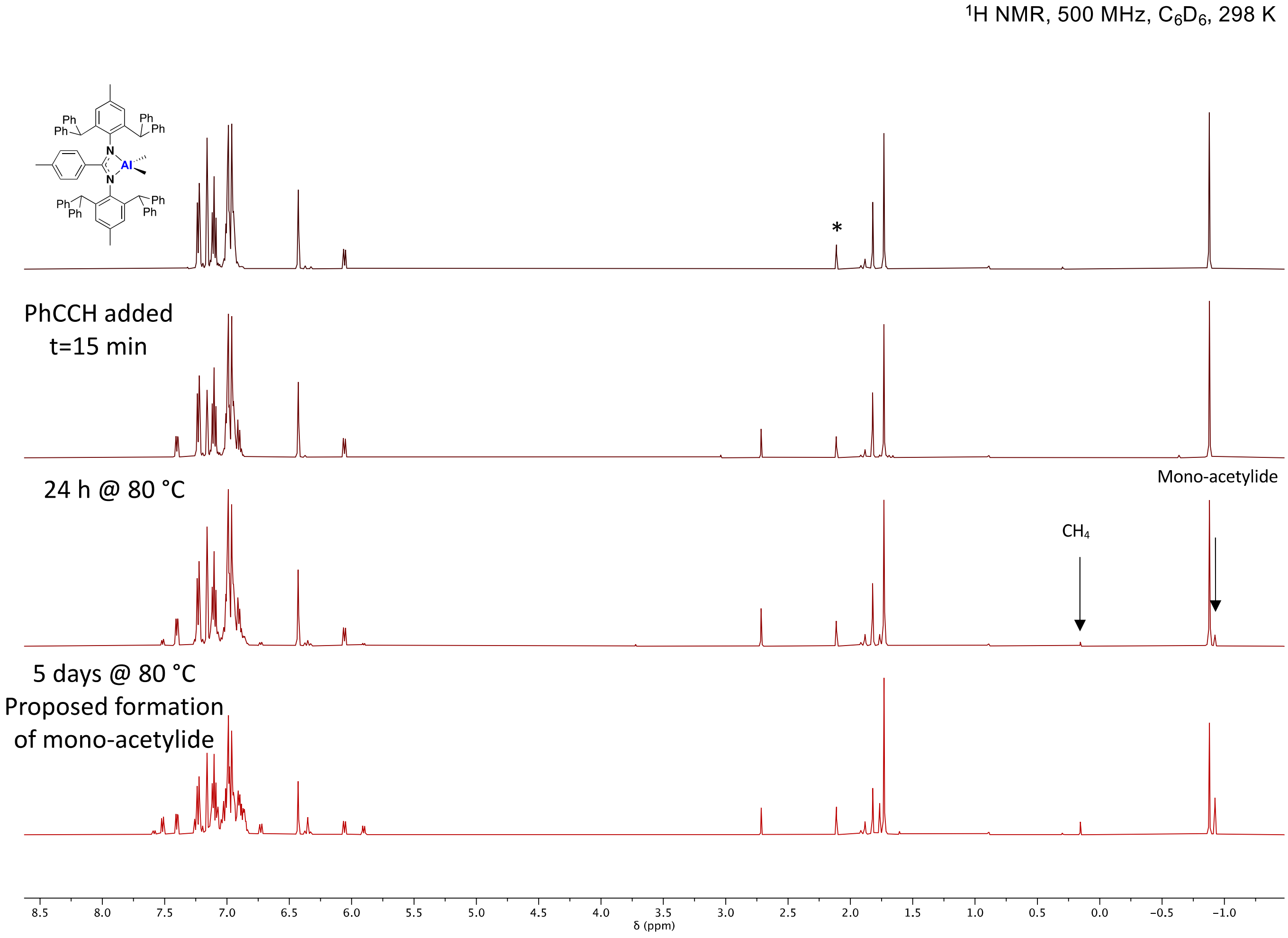

Figure S26: ^{1}H NMR stack of **6** + 1 equiv. HBpin

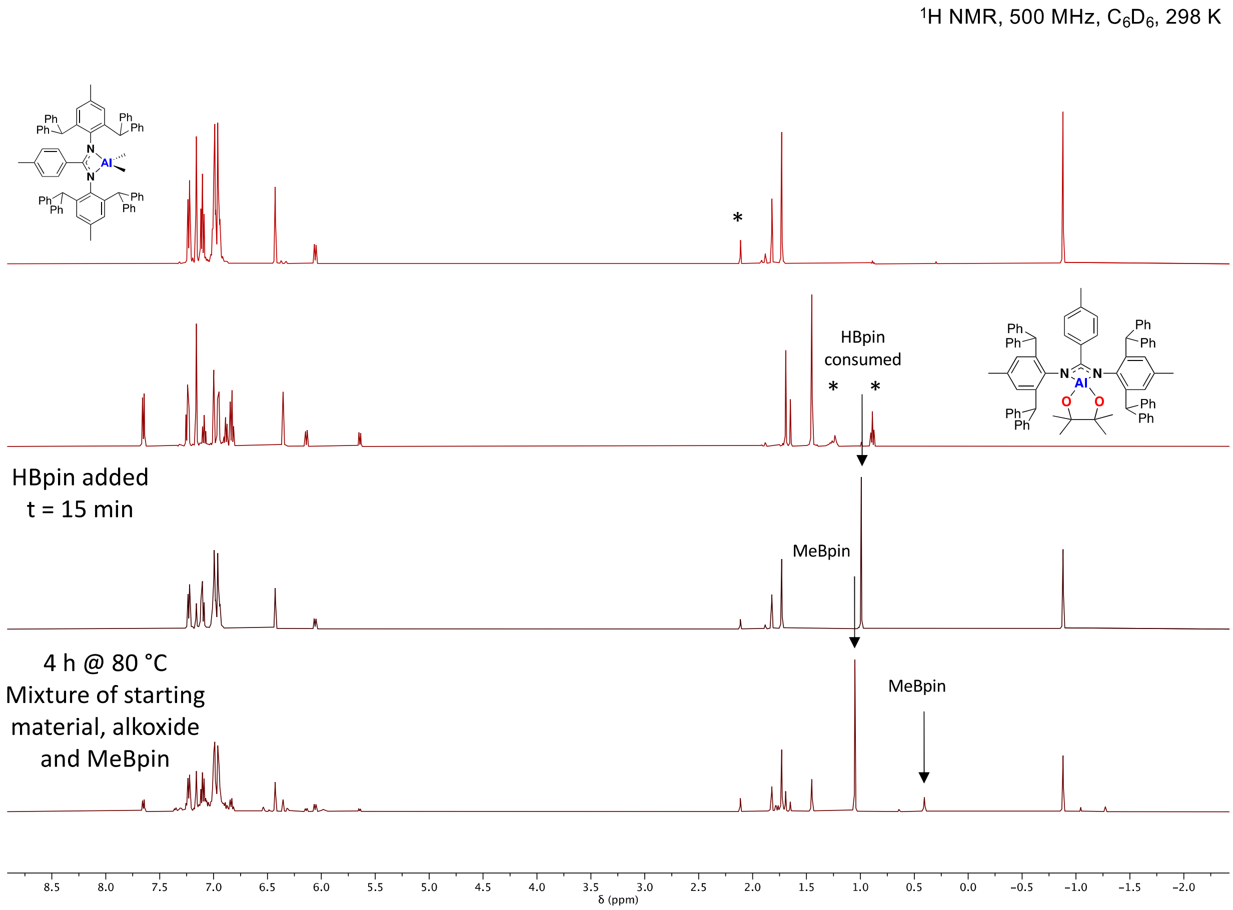

Figure S27: ^{11}B NMR of species present after the reaction of **6** + 1 equiv. HBpin

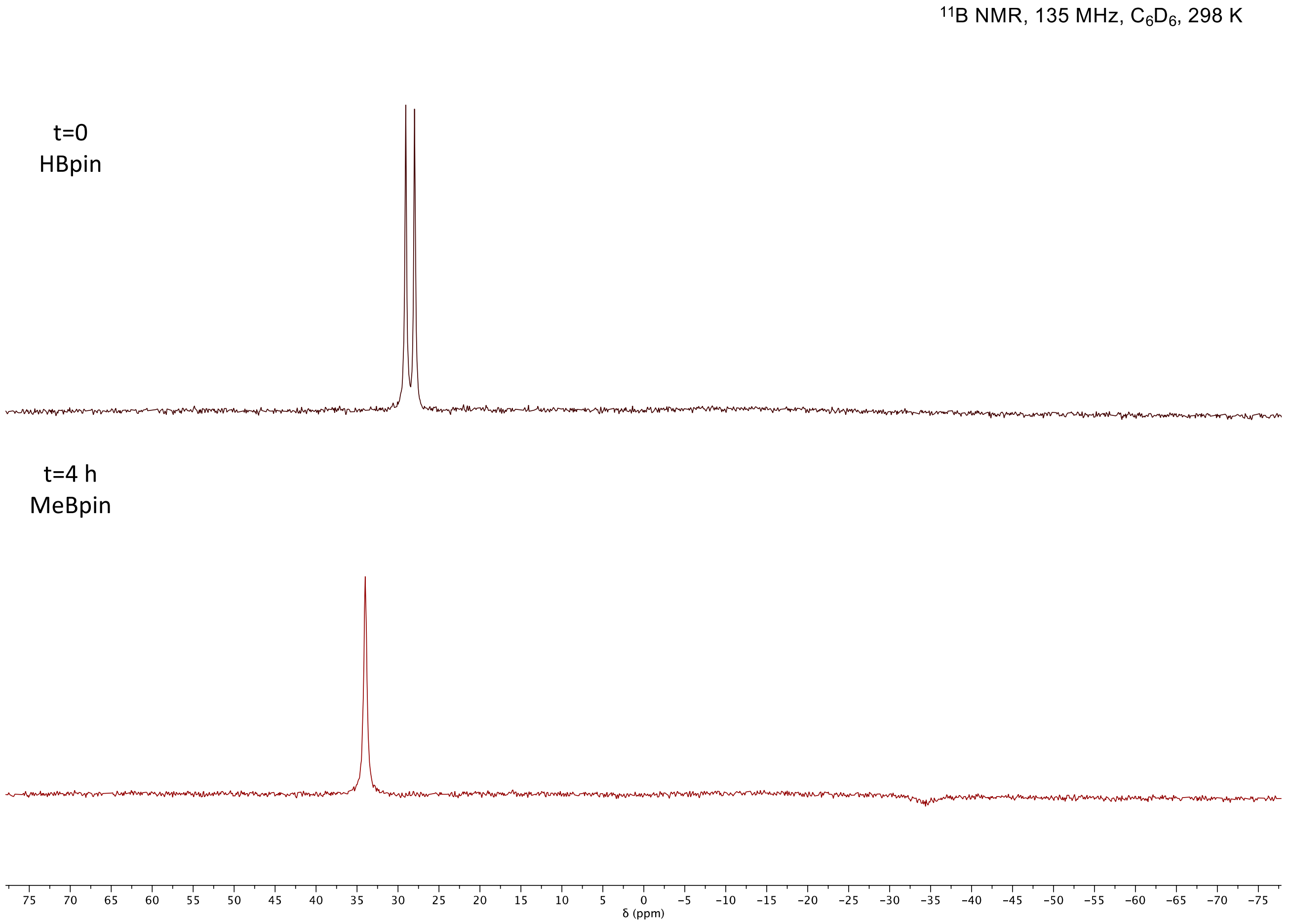

Figure S28: ^{1}H NMR stack of **6** + xs HBpin

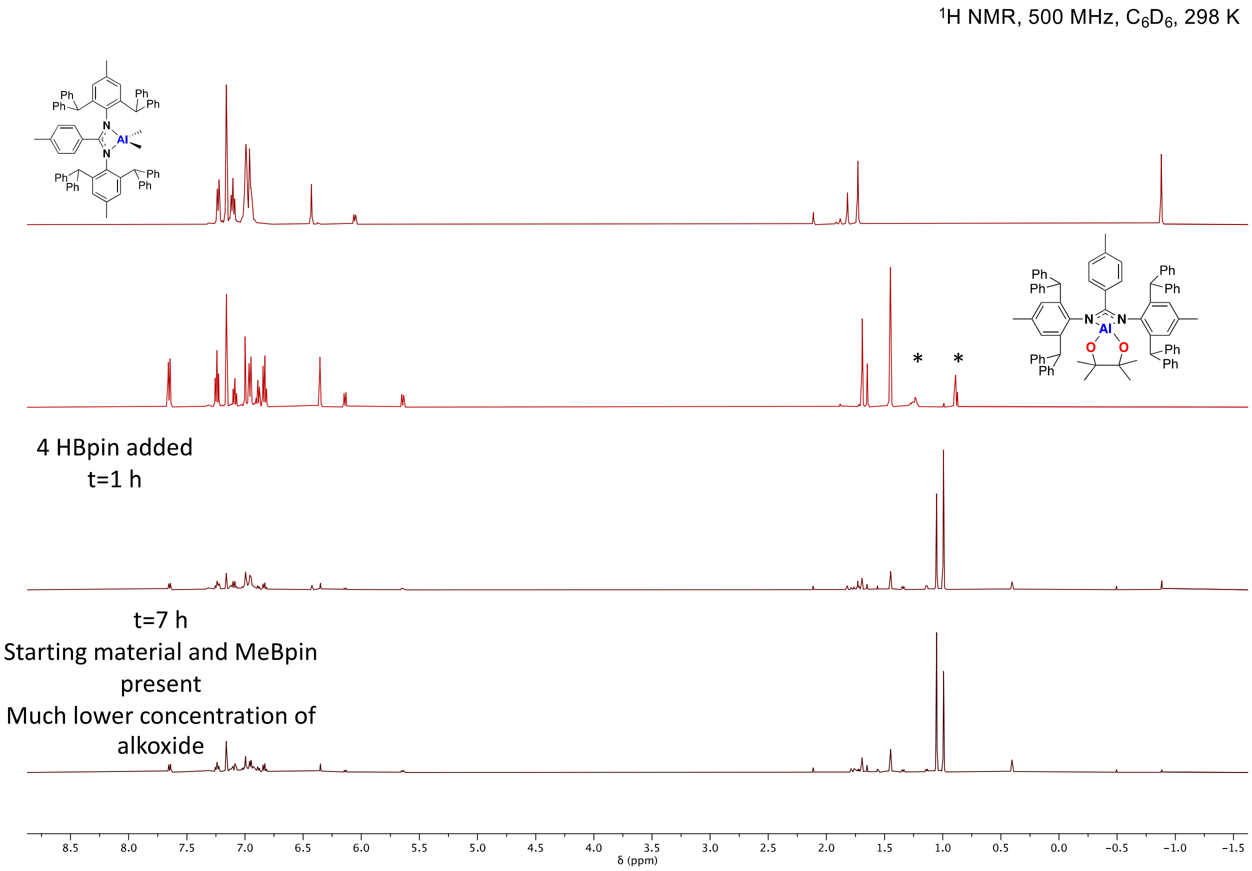

Figure S29: ^{11}B NMR of species present **6** + xs HBpin

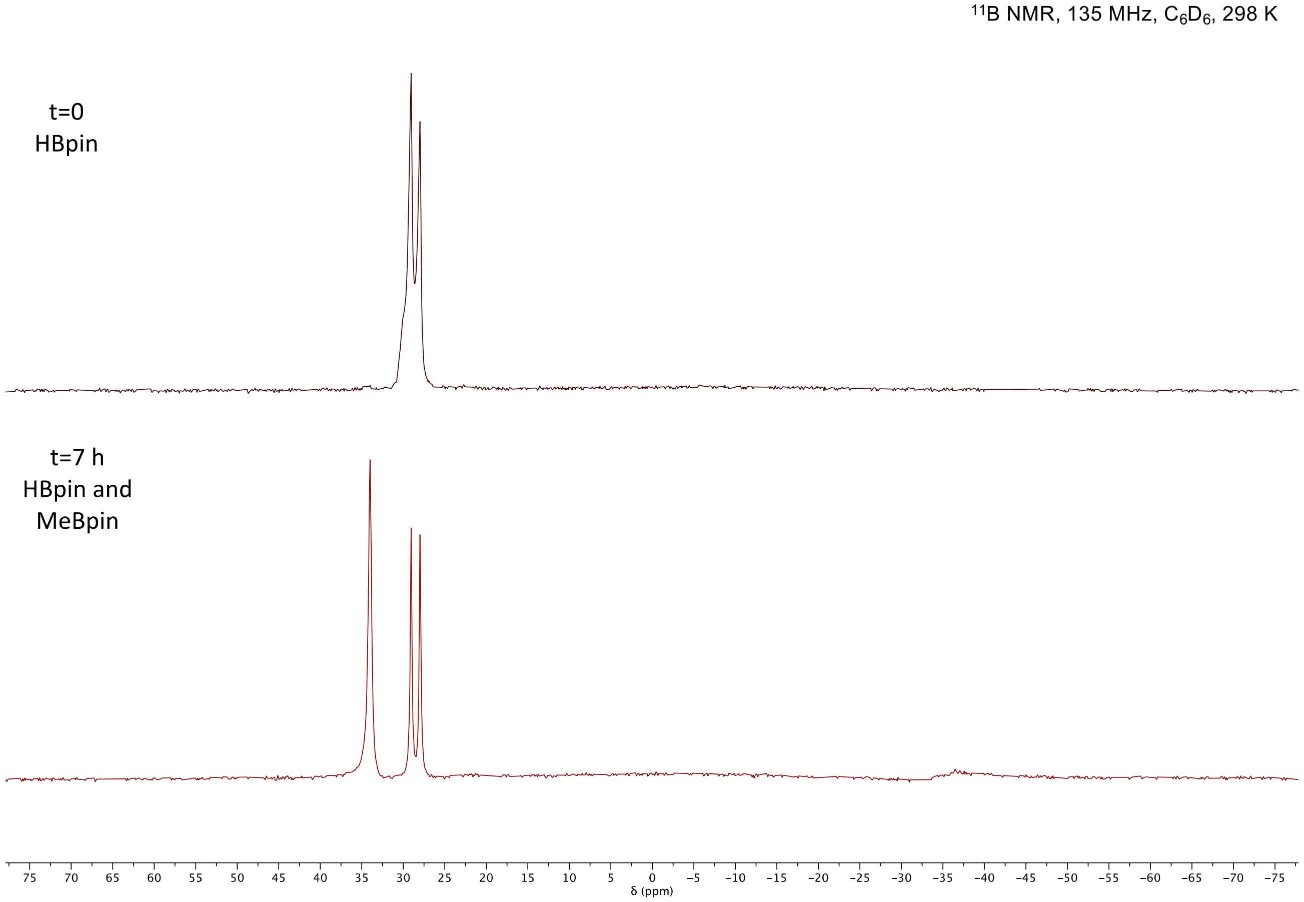

Figure S30: ^{1}H NMR of **4** + 10 HBpin

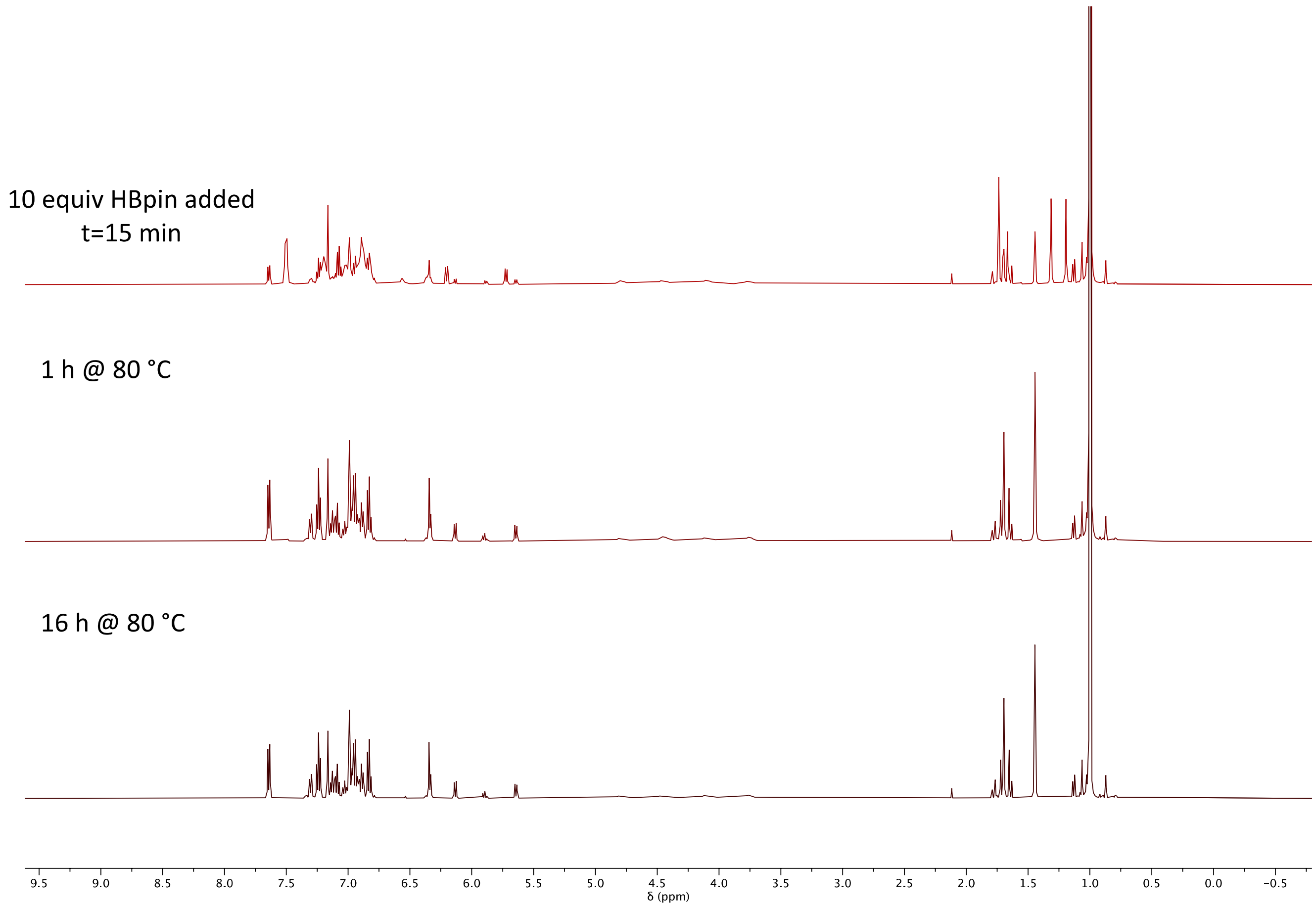

Figure S31: ^{11}B NMR of **4** + 10 HBpin

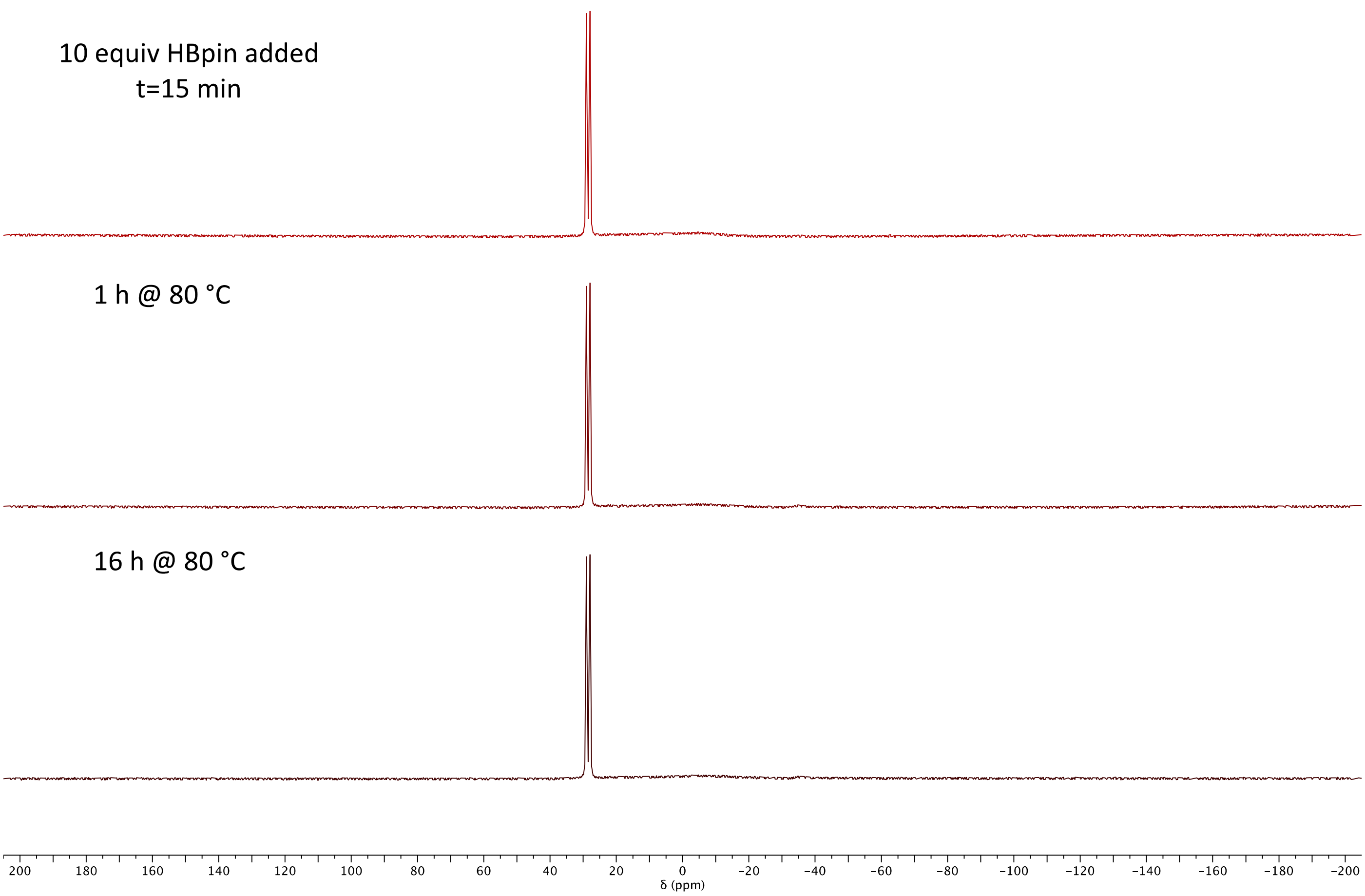

Figure S32: ^{1}H NMR stack of **5** + HBpin

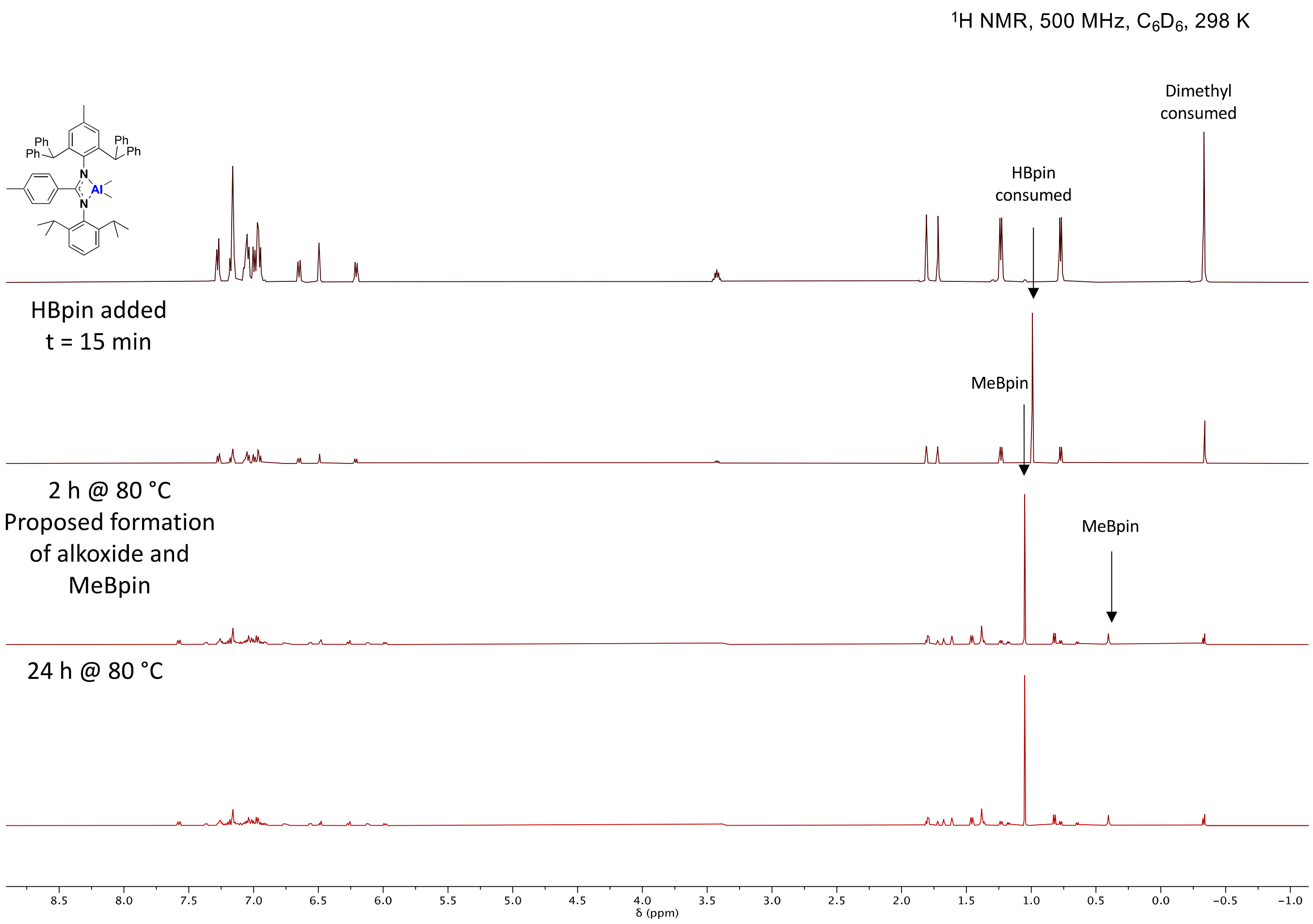

Figure S33: ^{11}B NMR of species present after the reaction of **5** + HBpin

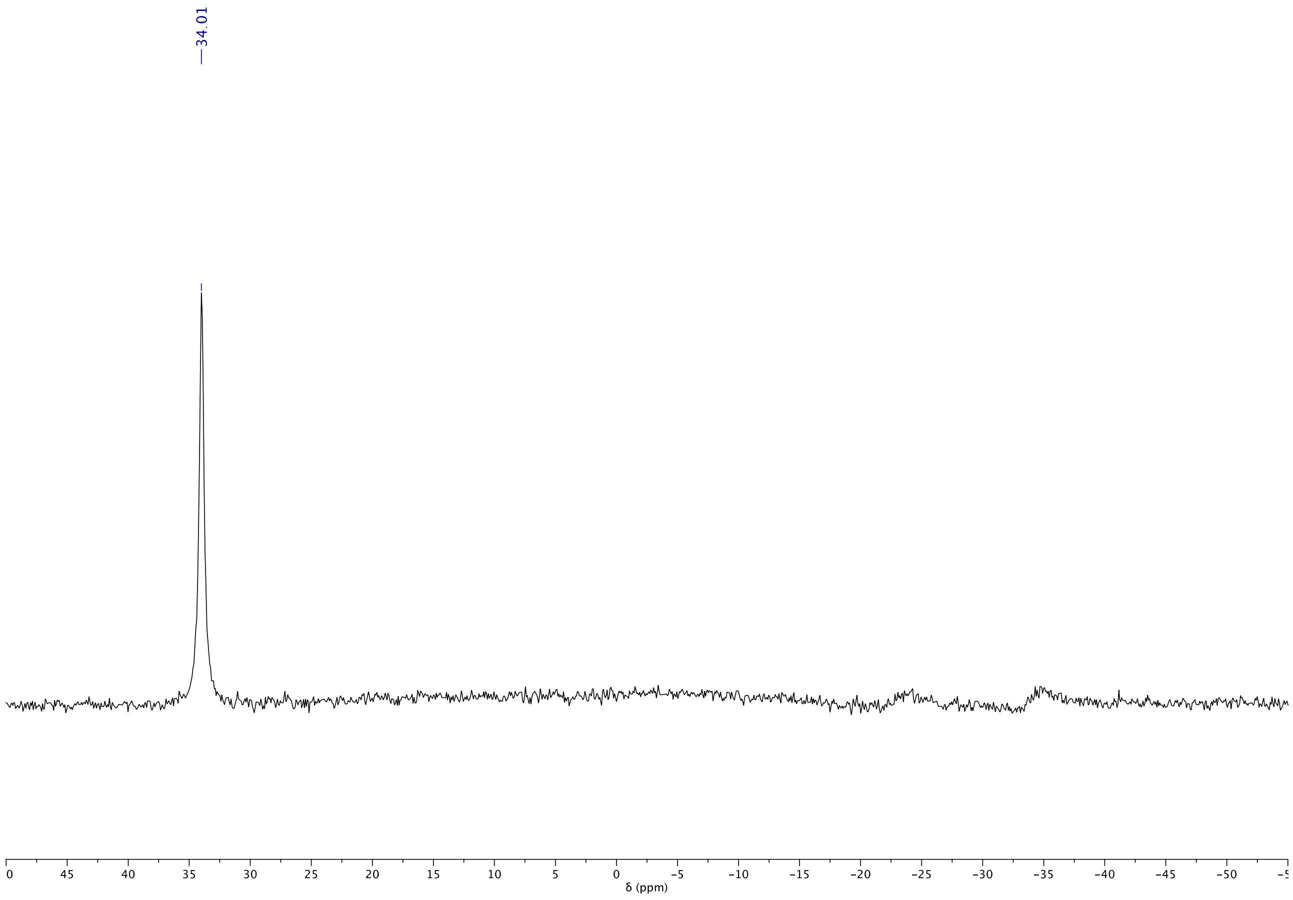

Figure S34: Representative ^{11}B NMR spectra of boron species present during catalysis for compounds **1, 3-4**

1, 3-4

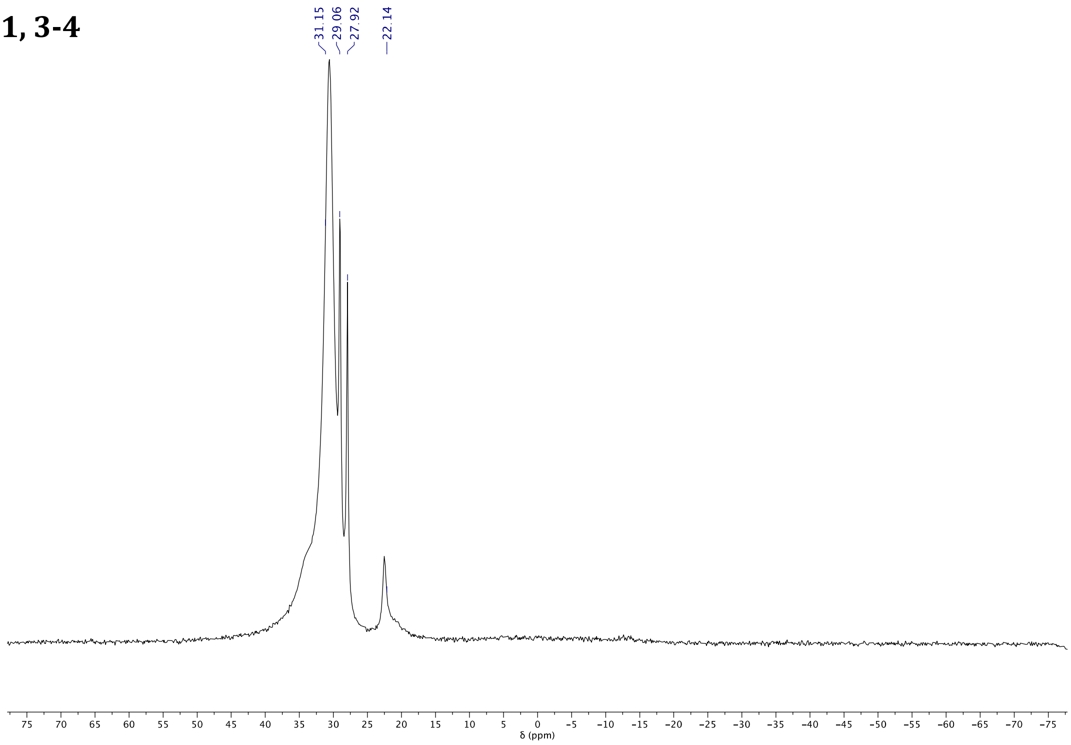

Figure S35: Representative ^{11}B NMR spectra of boron species present during catalysis for compounds **5-6**

5-6

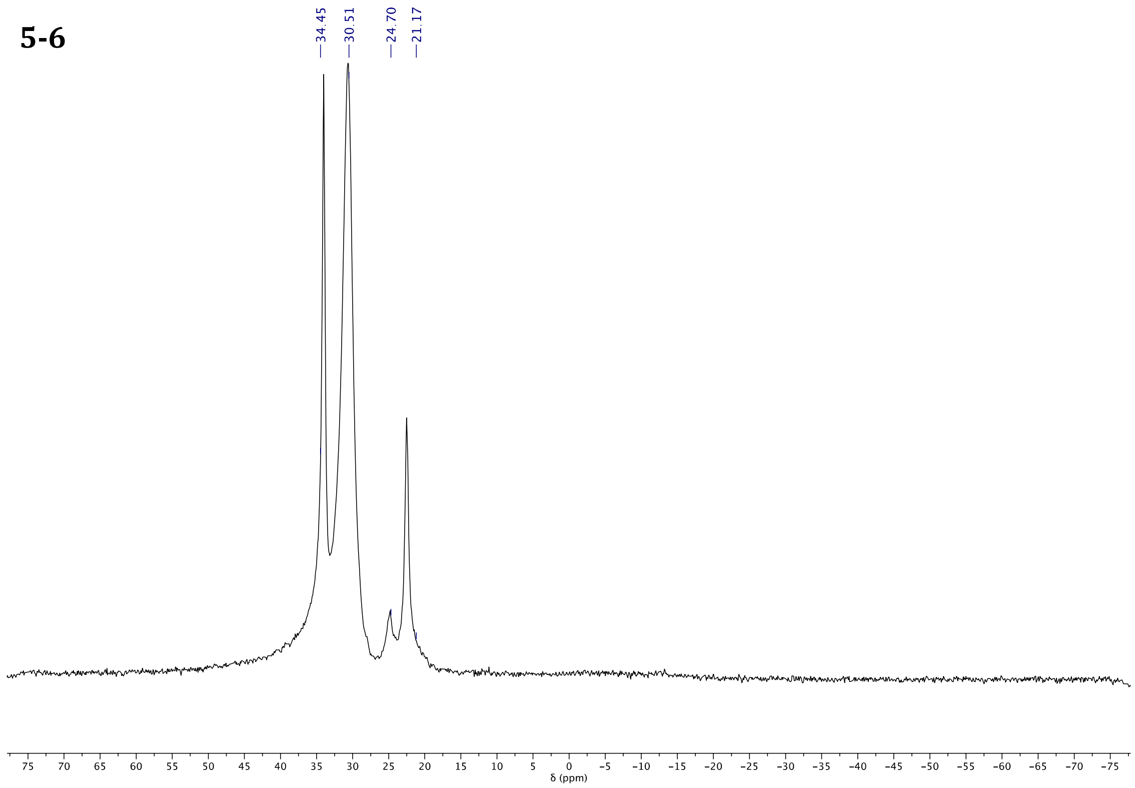

Figure S36: Representative ^{11}B NMR spectra of boron species present during catalysis for compound **2**

2

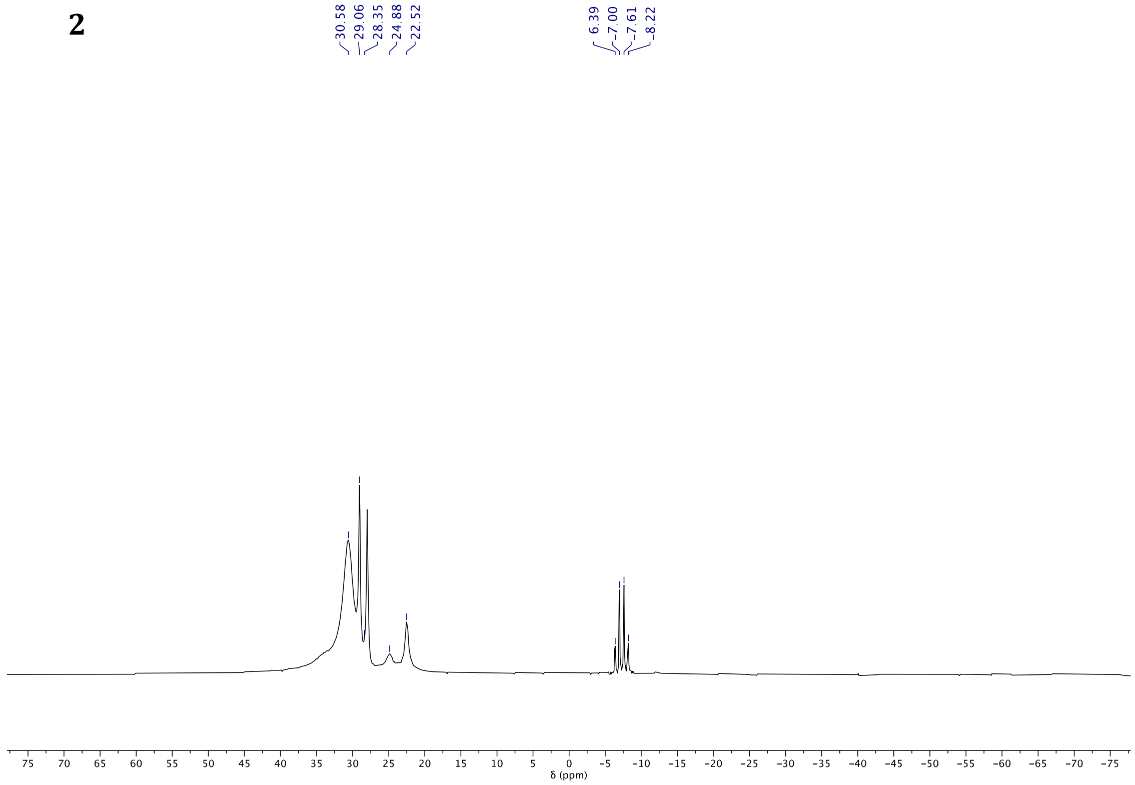

Figure S37: Representative ^{11}B NMR spectra of boron species present during non-catalytic reaction of **5** with HBpin + diphenylacetylene

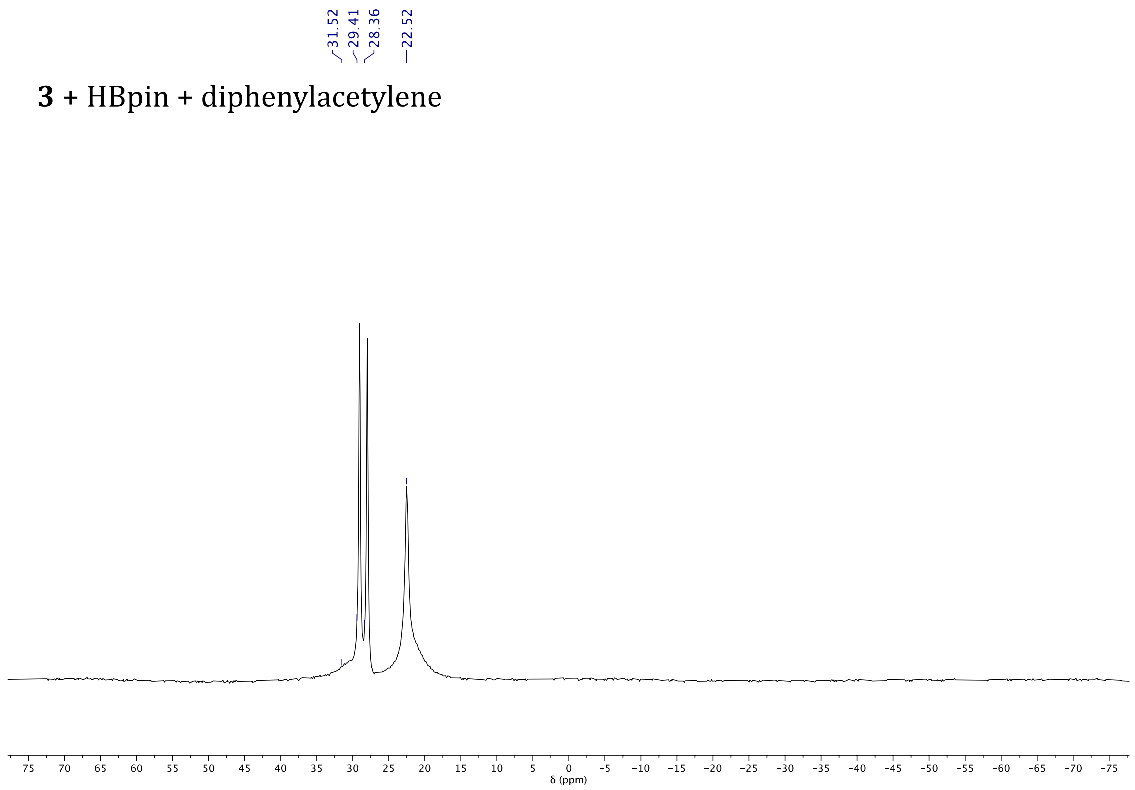

Figure S38: Representative ^{11}B NMR spectra of boron species present during non-catalytic reaction of **5** with HBpin + diphenylacetylene

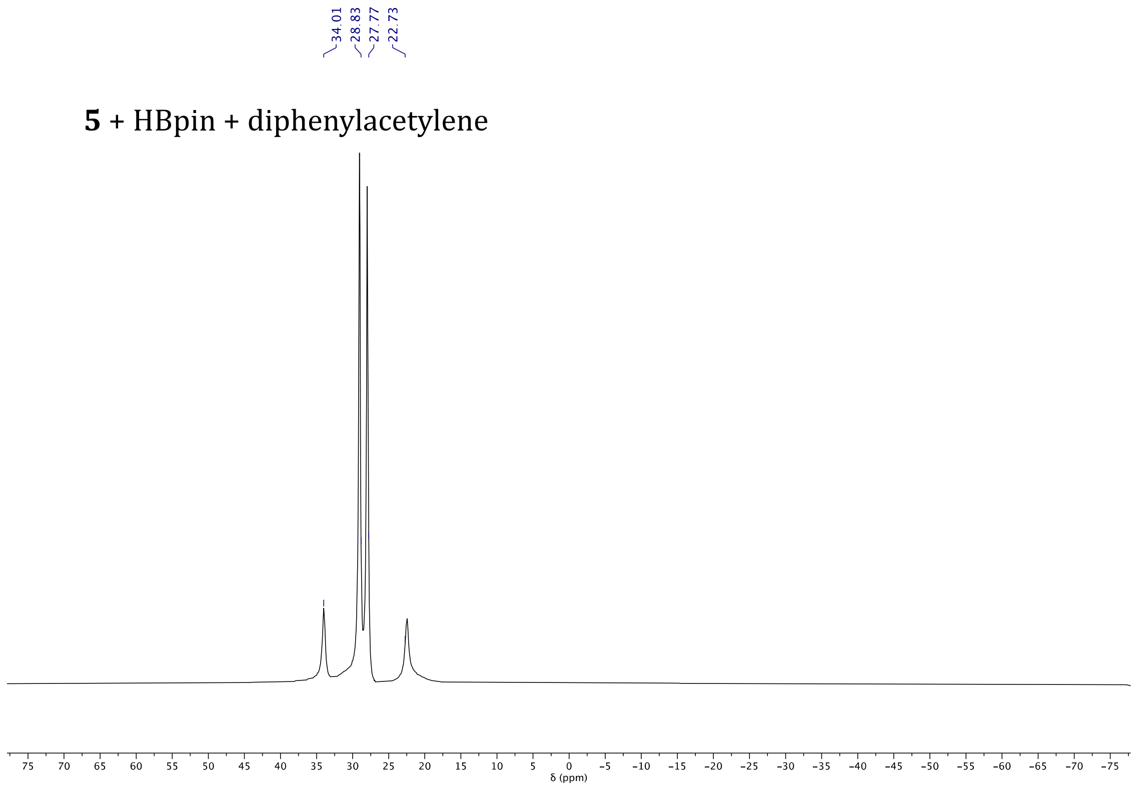

Figure S39: Stack of change in ^{11}B NMR over the course of reaction for $\mathbf{AlH_3{\bullet}NMe_3}$

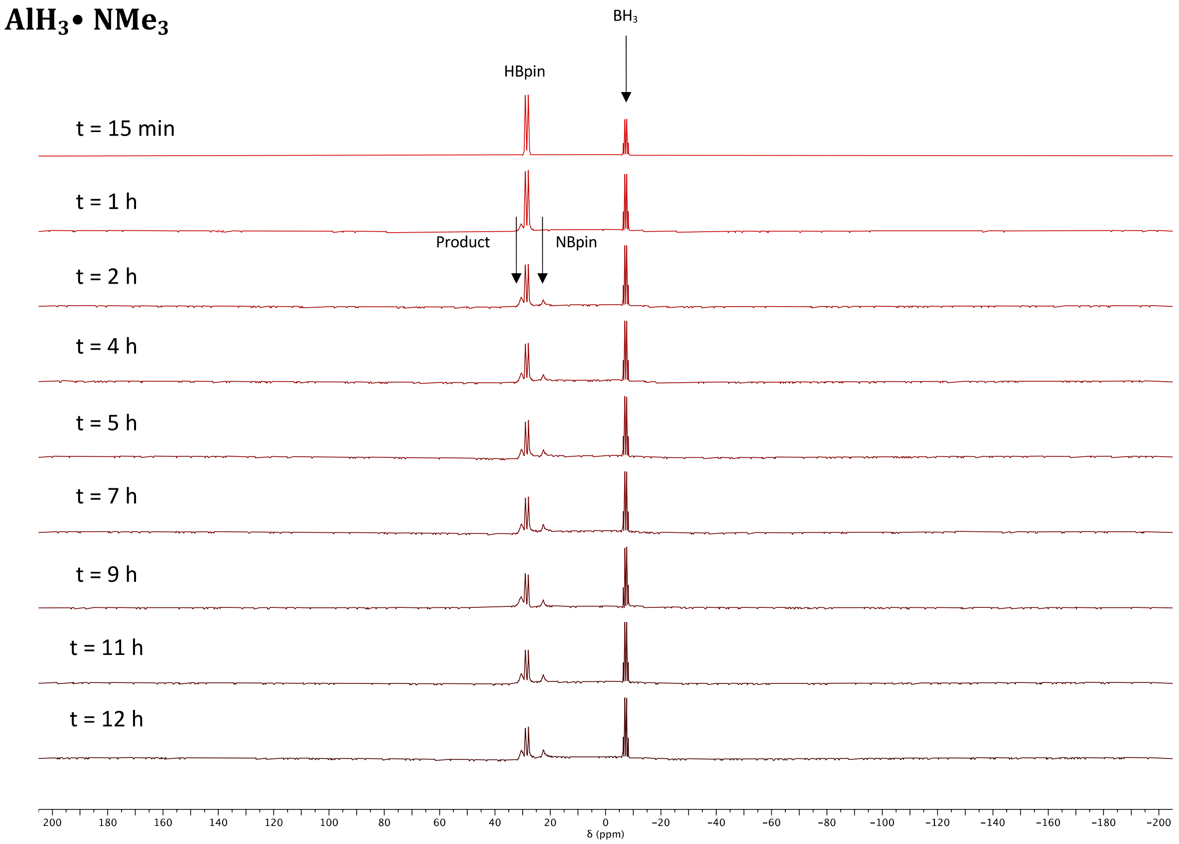

Figure S40: Stack of change in ^{11}B NMR over the course of reaction for catalyst **1**

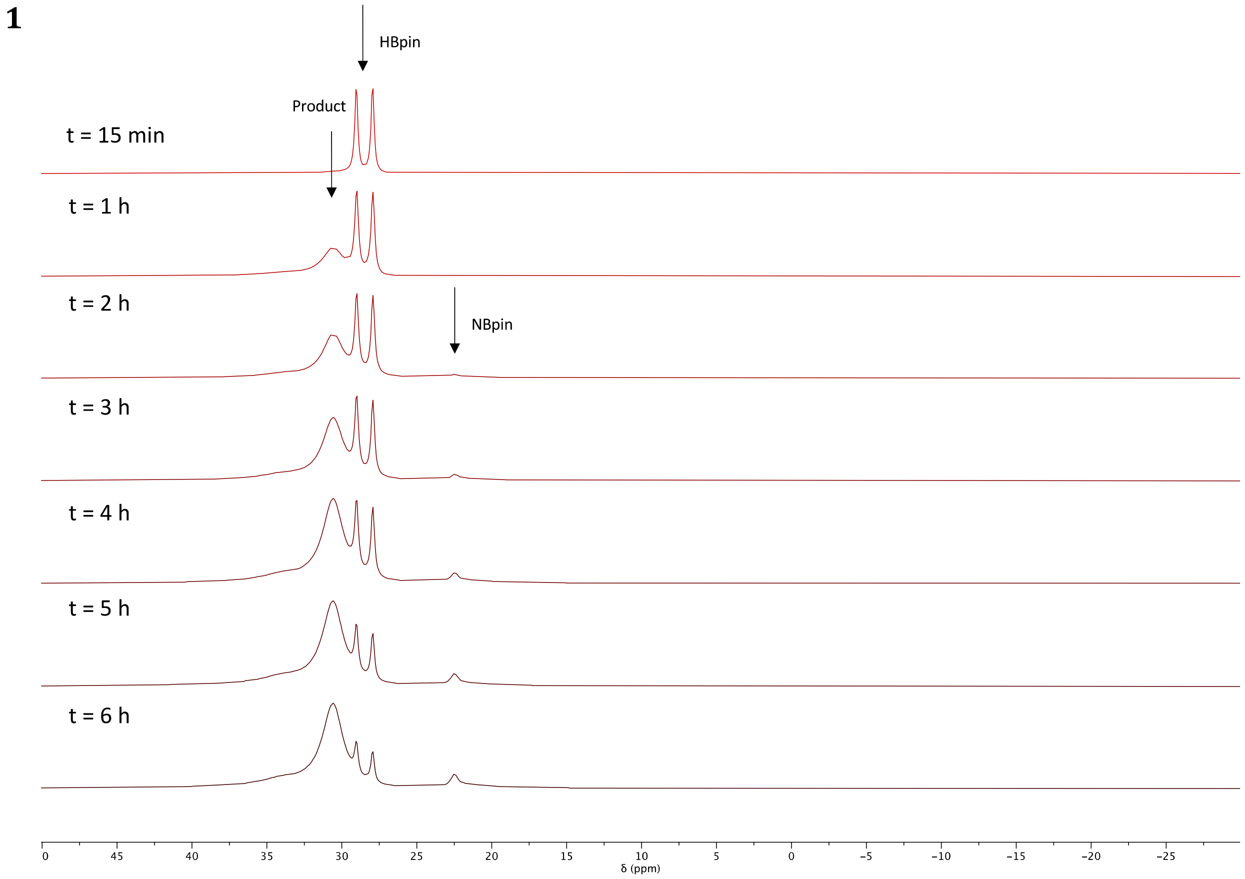

Figure S41: Stack of change in ^{11}B NMR over the course of reaction for catalyst **2**

2

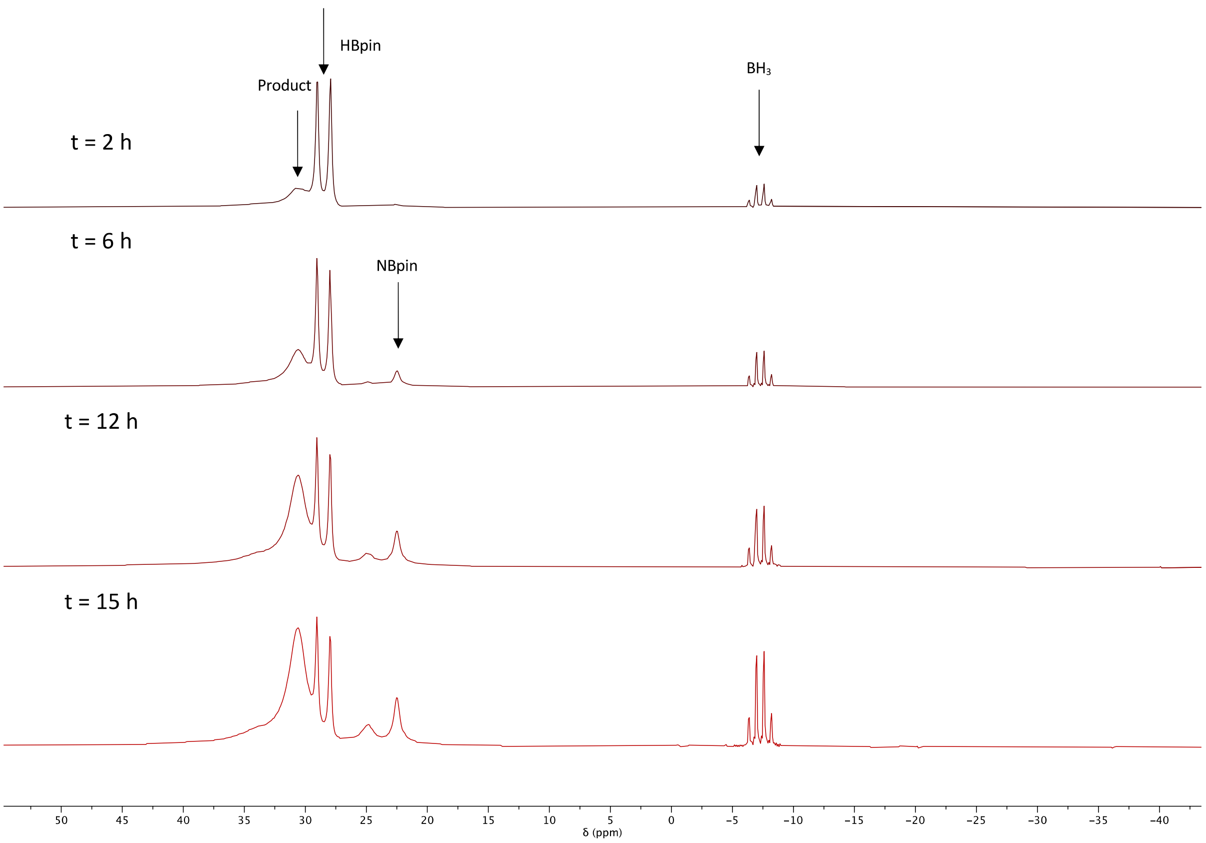

Figure S42: Stack of change in ^{11}B NMR over the course of reaction for catalyst **3**

3

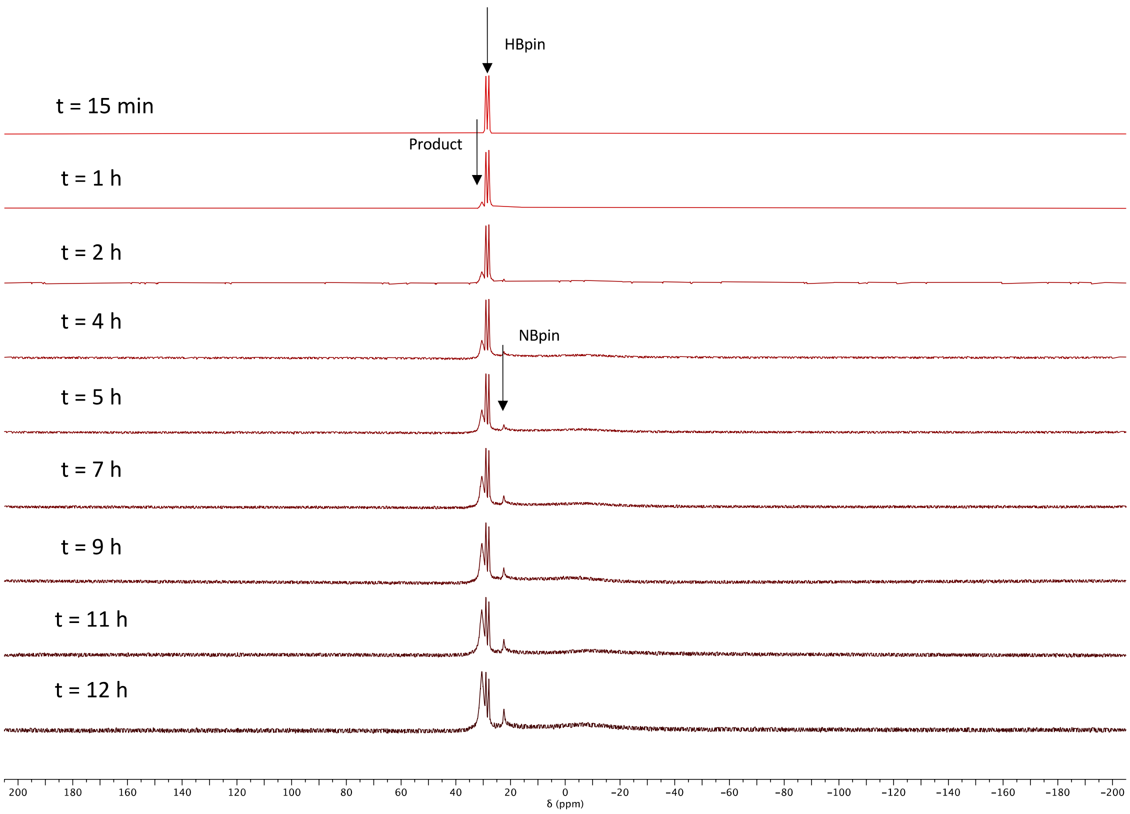

Figure S43: Stack of change in ^{11}B NMR over the course of reaction for catalyst **4**

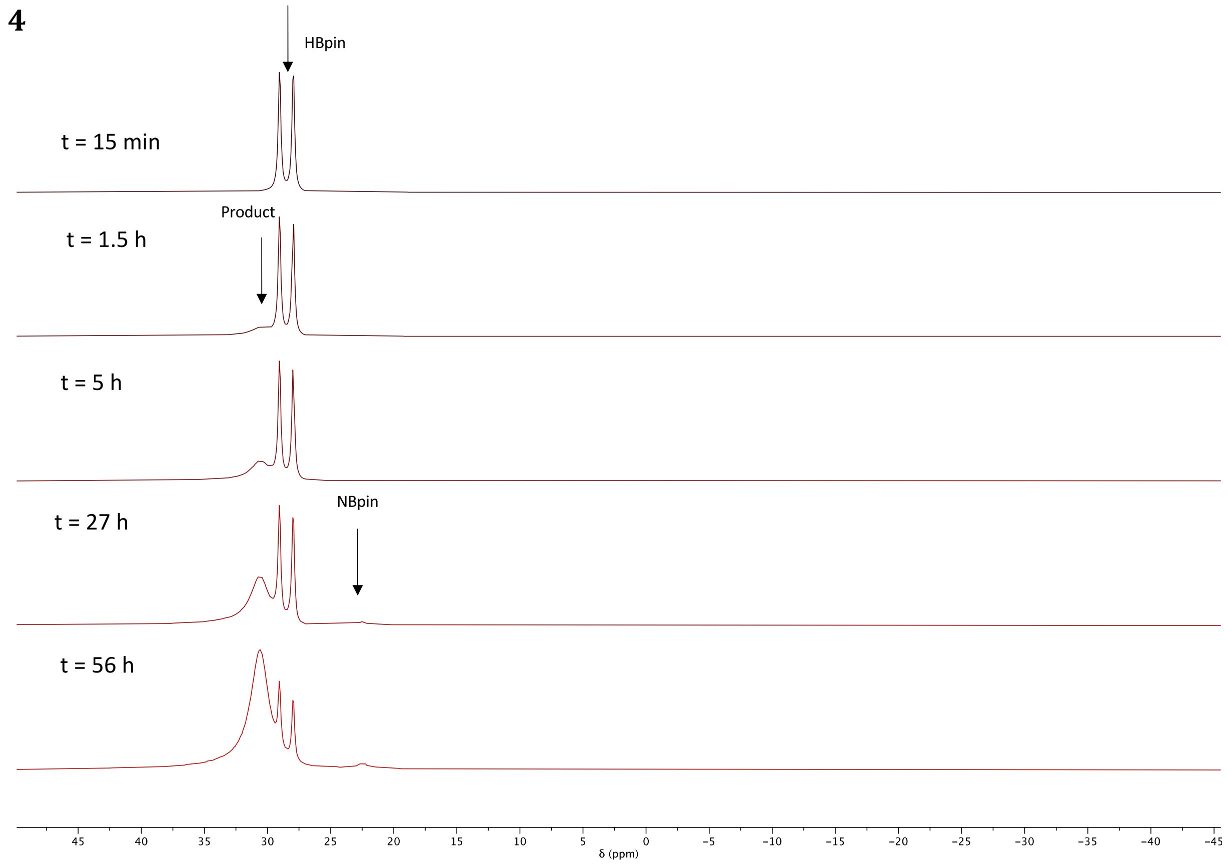

Figure S44: Stack of change in ^{11}B NMR over the course of reaction for catalyst **5**

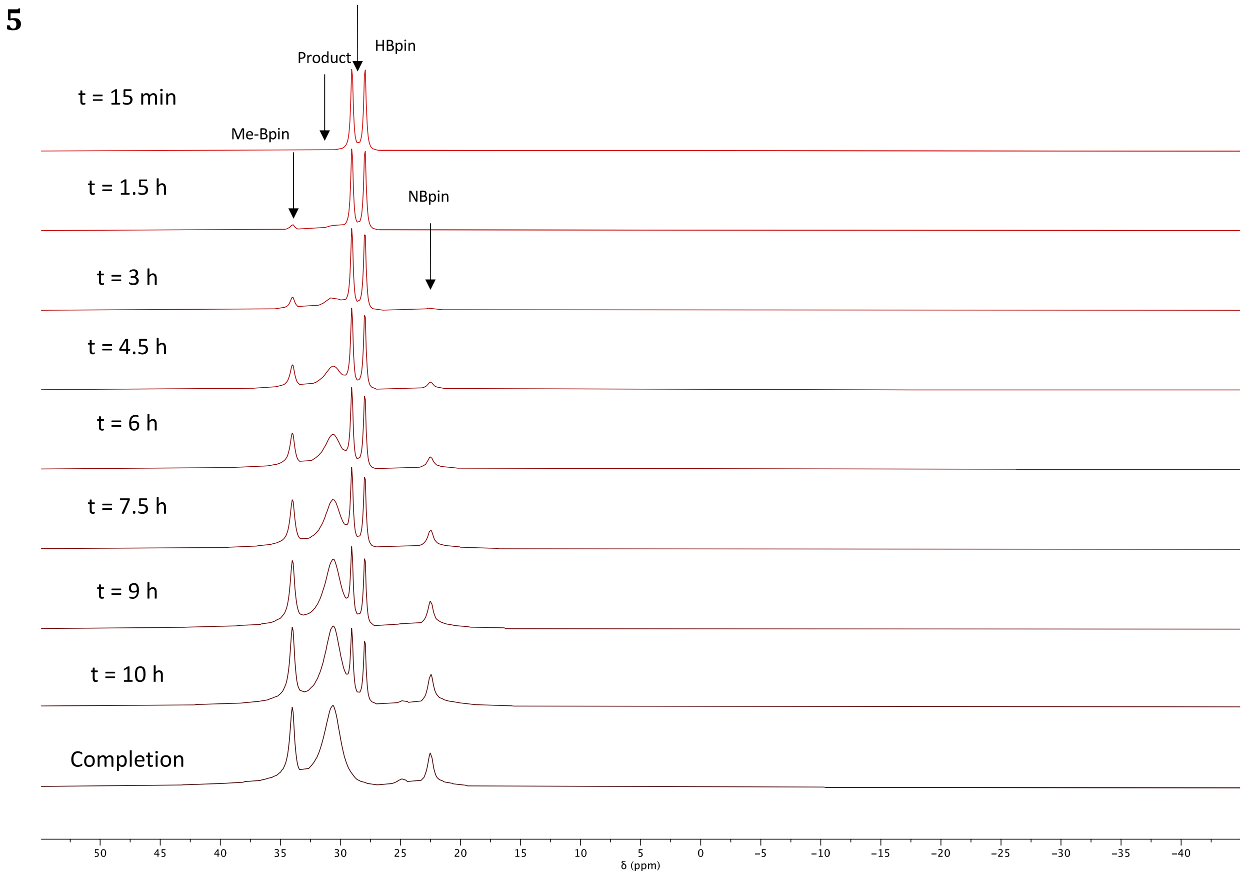

Table S3: Resonances of additional boron species present during catalysis. ^{11}B NMR (138 MHz, C_6D_6, 298 K) δ (ppm): -7 (**B**H_3), 22 (N-**B**pin), 24 (N-**B**pin), 34 (Me-**B**pin)

Cat.	^{11}B NMR resonances (ppm)
**1**	22
**2**	-7, 22, 24
**3**	22
**4**	22
**5**	22, 24, 34
**6**	22, 24, 34

4 Kinetics

Figure S45: Plots to show concentration versus time (a), plot assuming first order kinetics (b) and plot assuming second order kinetics (c) for catalyst **1**. [HBpin] = 0.25 M.

1

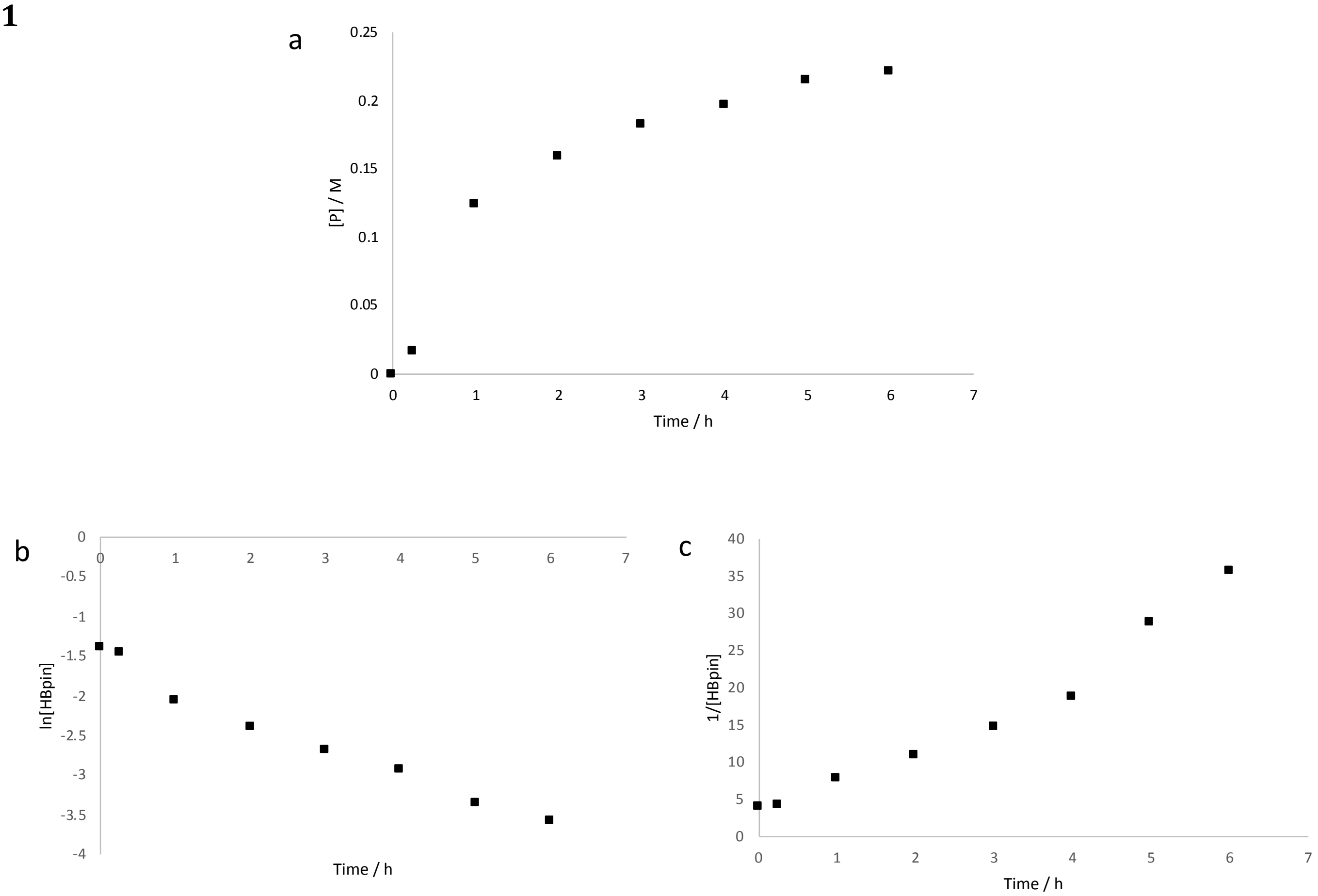

Figure S46: Plots to show concentration versus time (a), plot assuming first order kinetics (b) and plot assuming second order kinetics (c) for catalyst **2**. [HBpin] = 0.25 M.

2

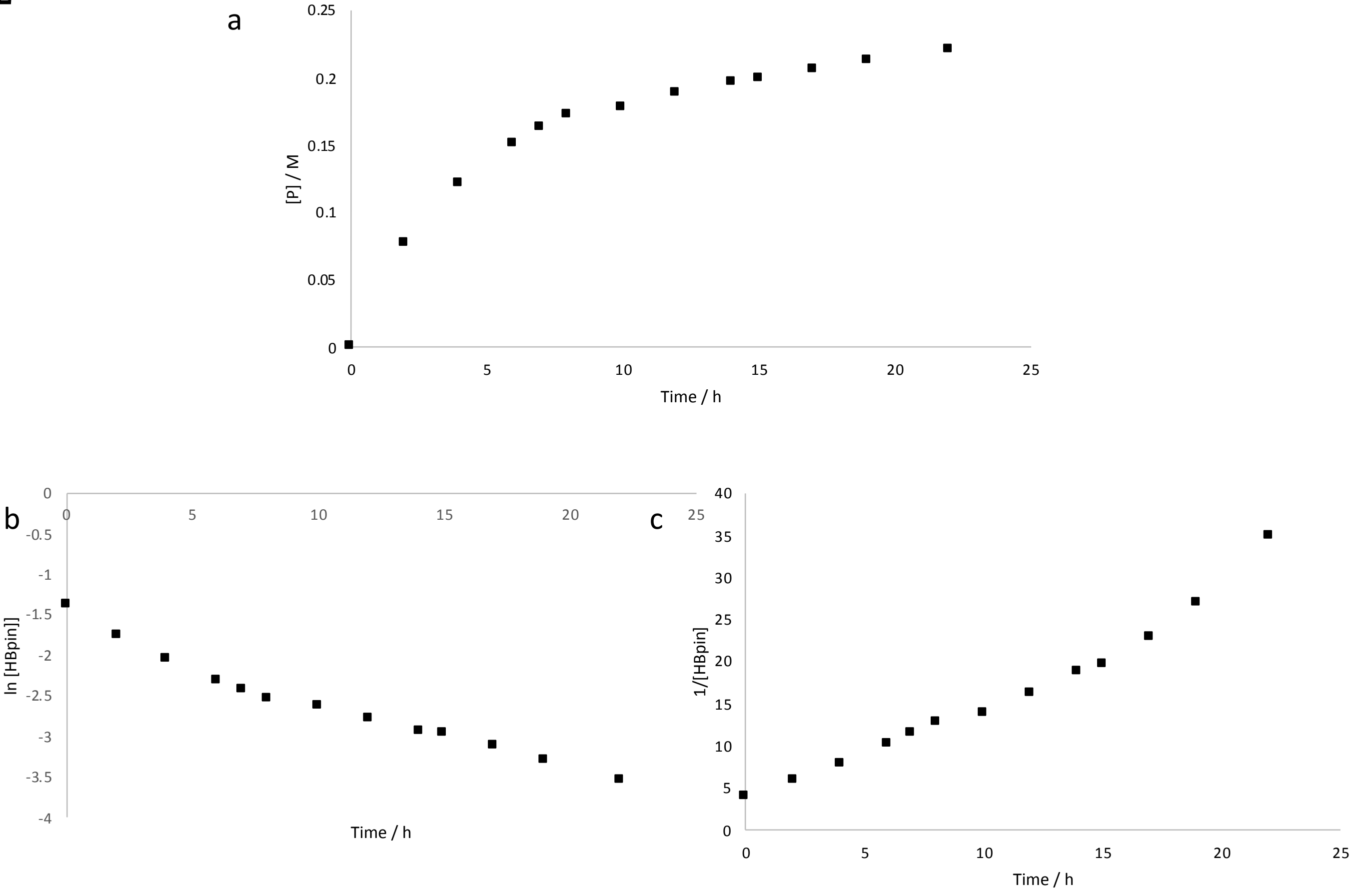

Figure S47: Plots to show concentration versus time (a), plot assuming first order kinetics (b) and plot assuming second order kinetics (c) for catalyst **3**. [HBpin] = 0.25 M.

3

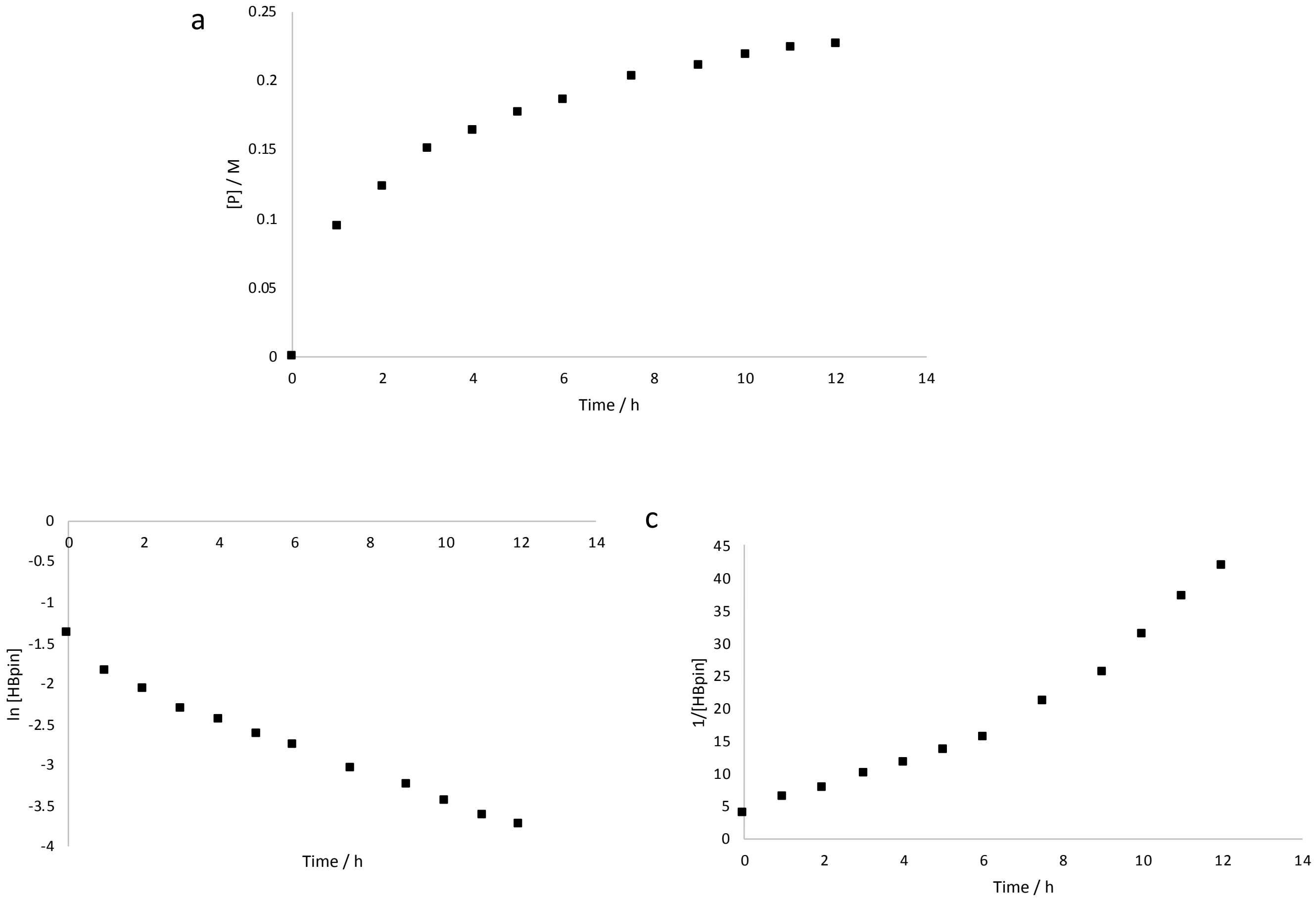

Figure S48: Plots to show concentration versus time (a), plot assuming first order kinetics (b) and plot assuming second order kinetics (c) for catalyst **4**. [HBpin] = 0.25 M.

4

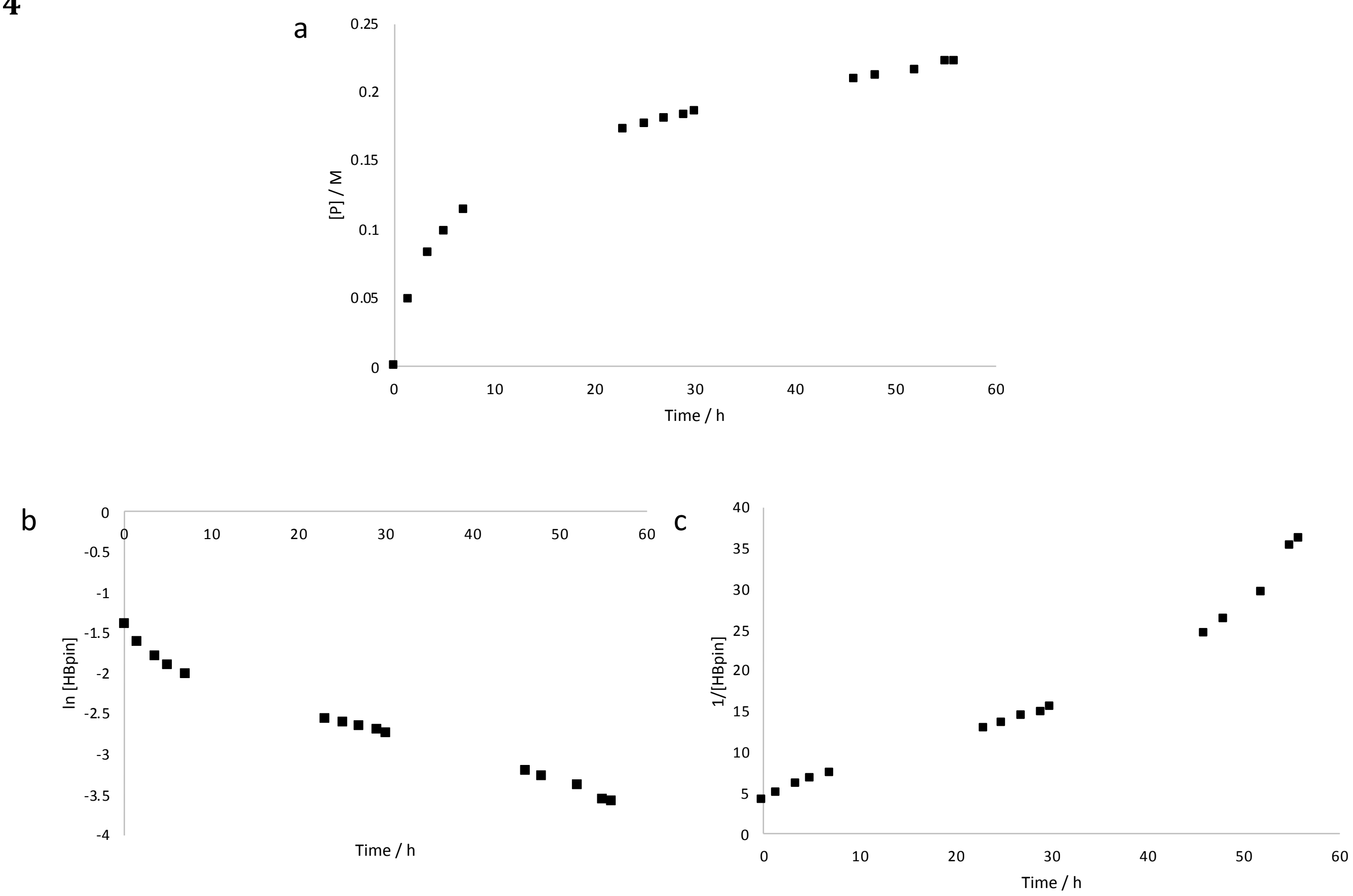

Figure S49: Plots to show concentration versus time (a) and plot assuming first order catalysis (b) for catalyst **5**. [HBpin] = 0.25 M.

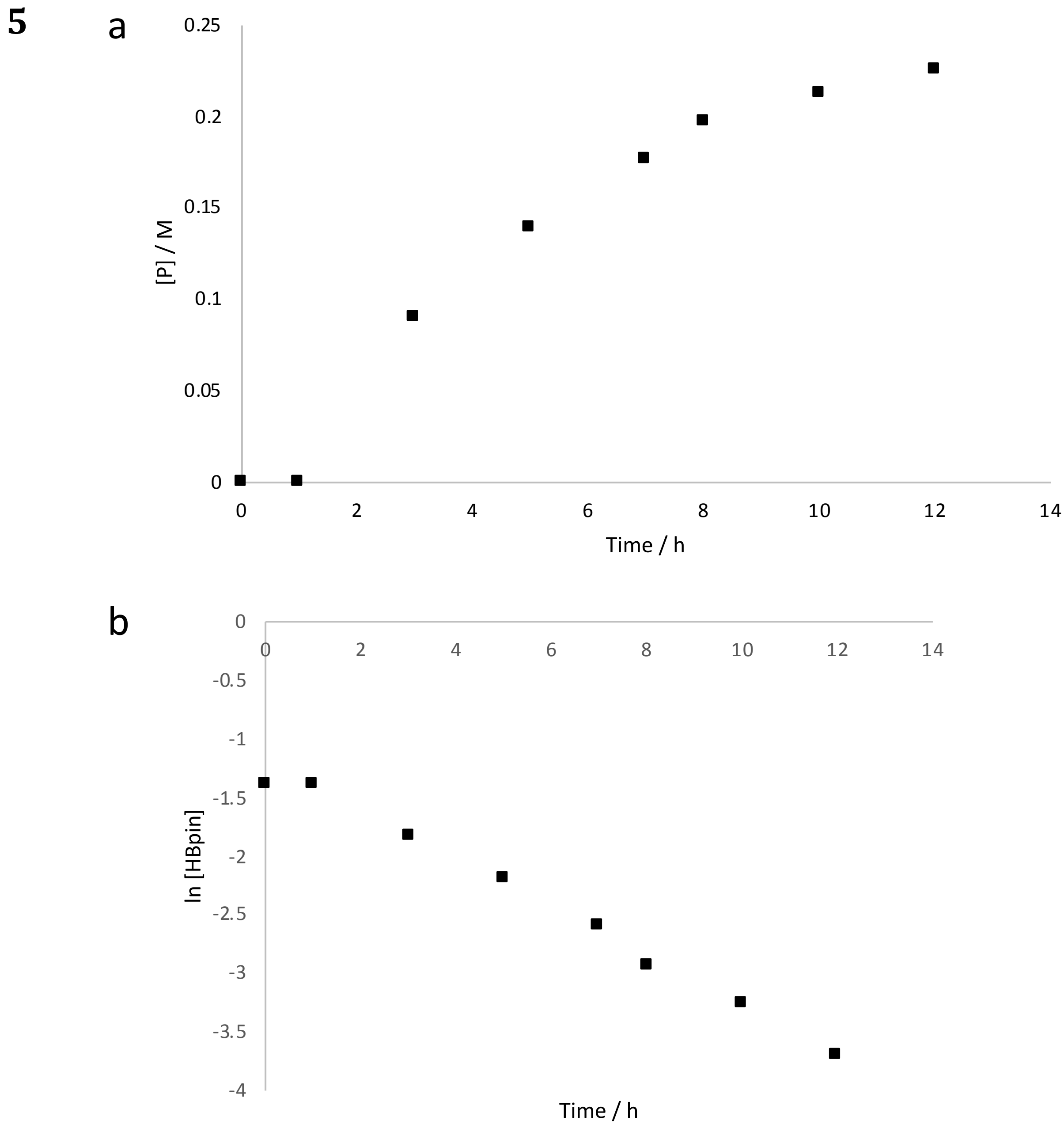

Figure S50: Plots to show concentration versus time (a) and attempted plot assuming first order catalysis (b) for catalyst **6**. [HBpin] = 0.25 M.

6

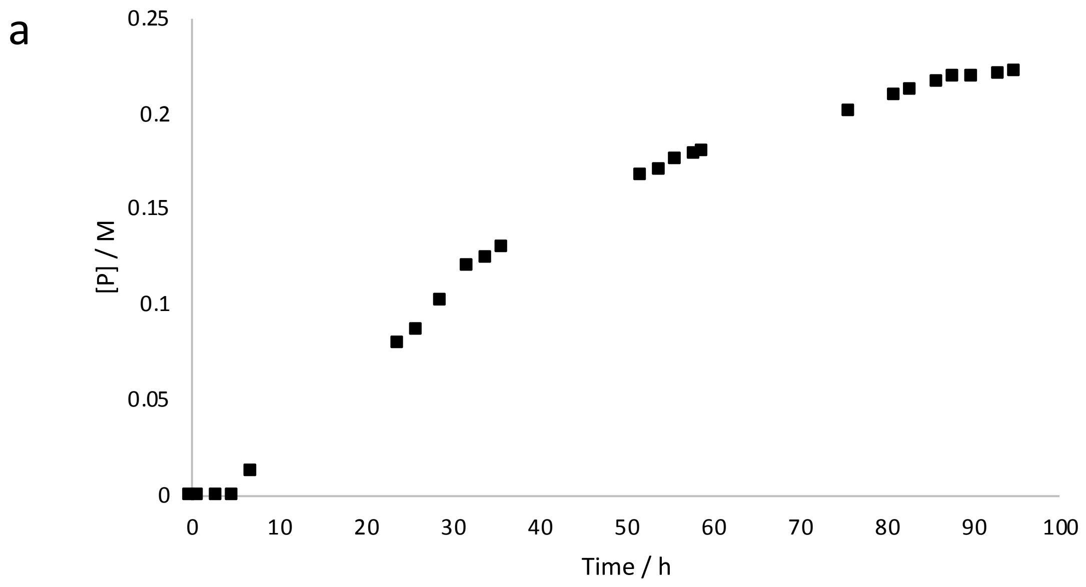

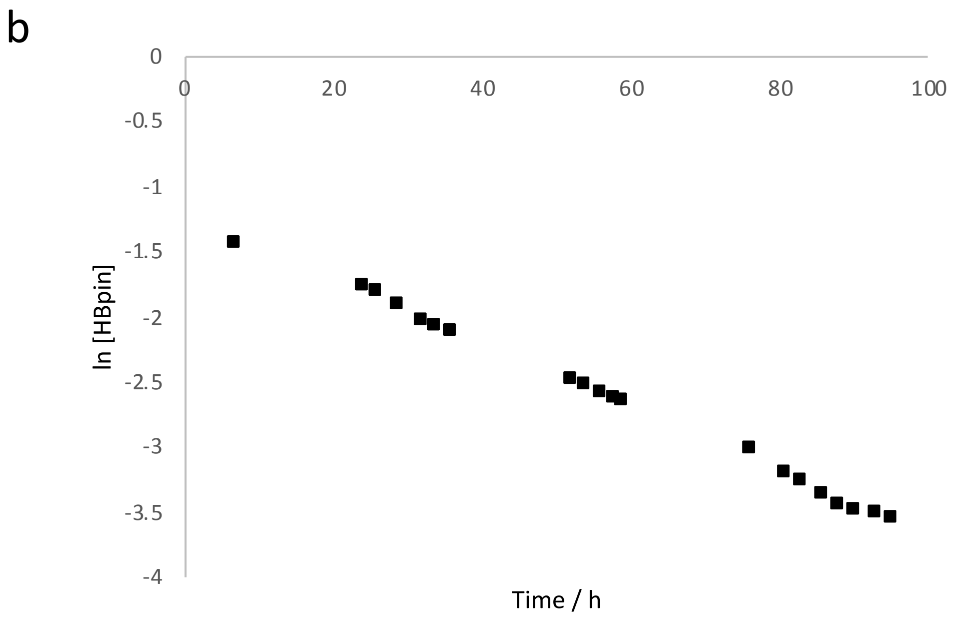

Figure S51: Plots to show concentration versus time (a) and plot assuming second order catalysis (b) for **$AlH_3.NMe_3$** *note - this reaction did not run to completion

AlH_3• NMe_3

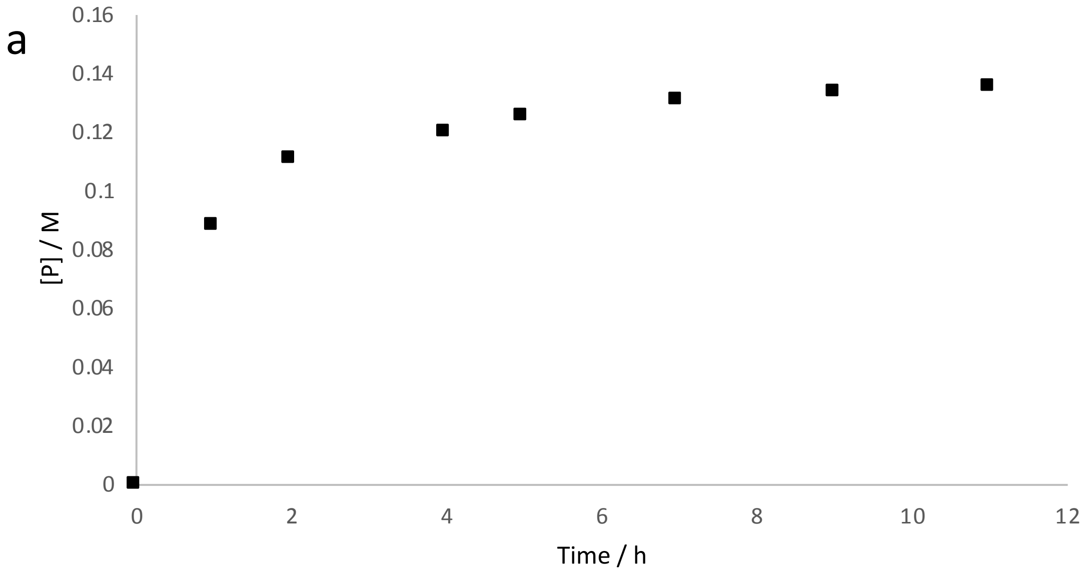

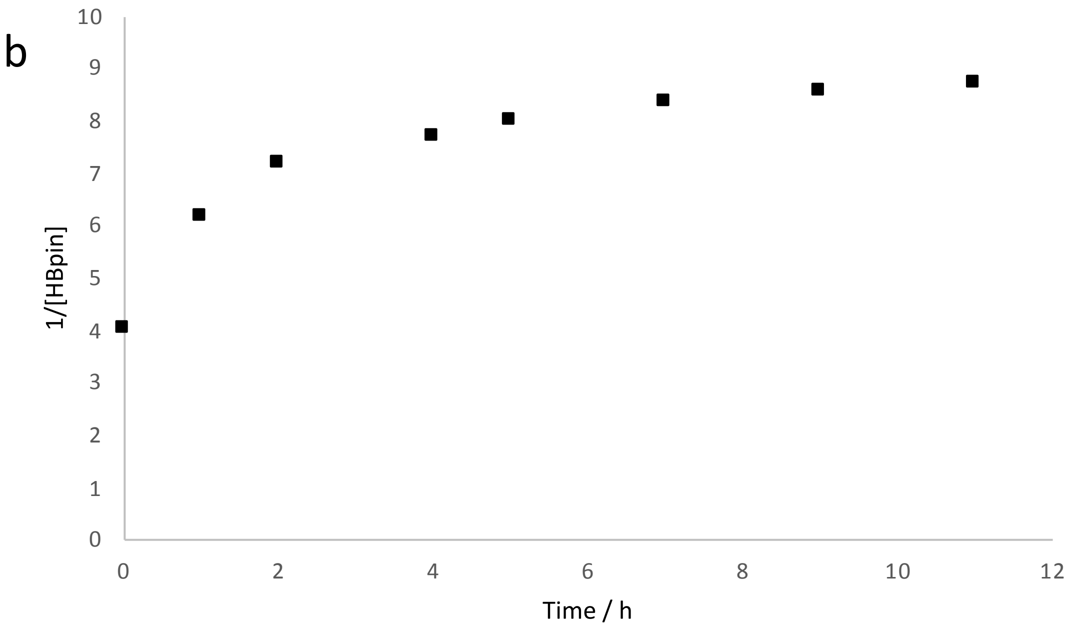

Figure S52: Plot of concentration versus time on the same graph for catalysts **1-3, 5**.

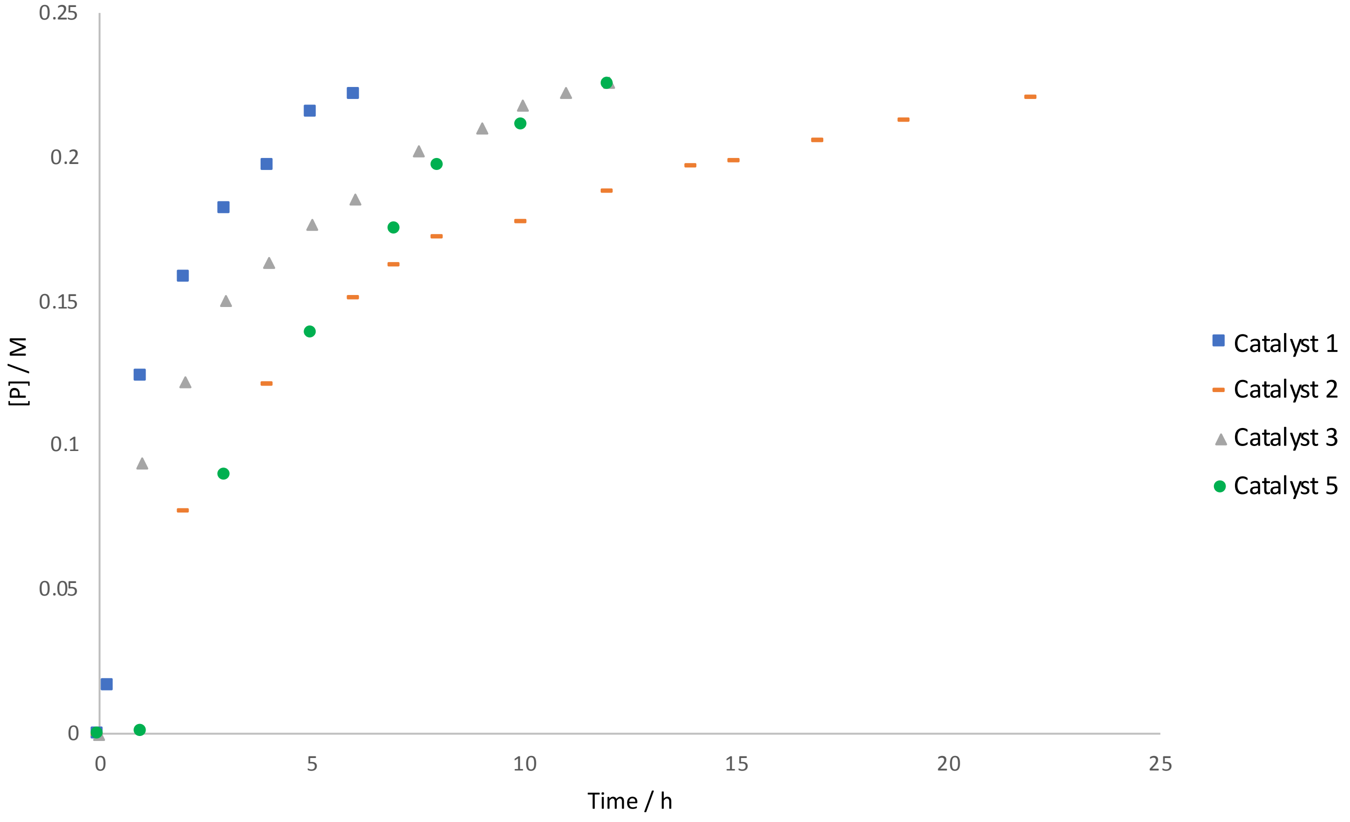

Figure S53: Plot of concentration versus time on the same graph for catalyst **4** and **6**

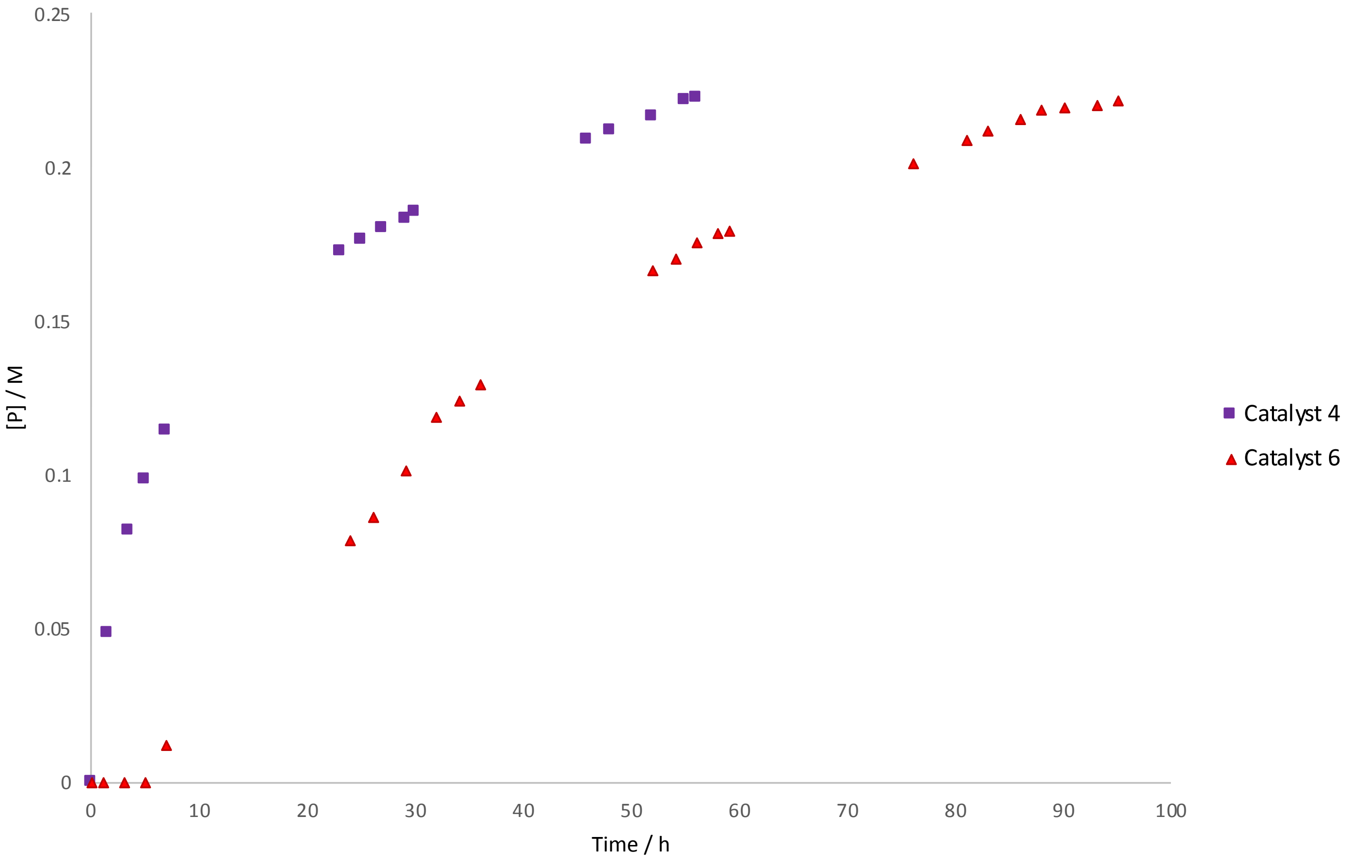

Figure S54: A speculative catalytic cycle with the formation of an aluminium dihydride - HBpin adduct as the active catalyst.

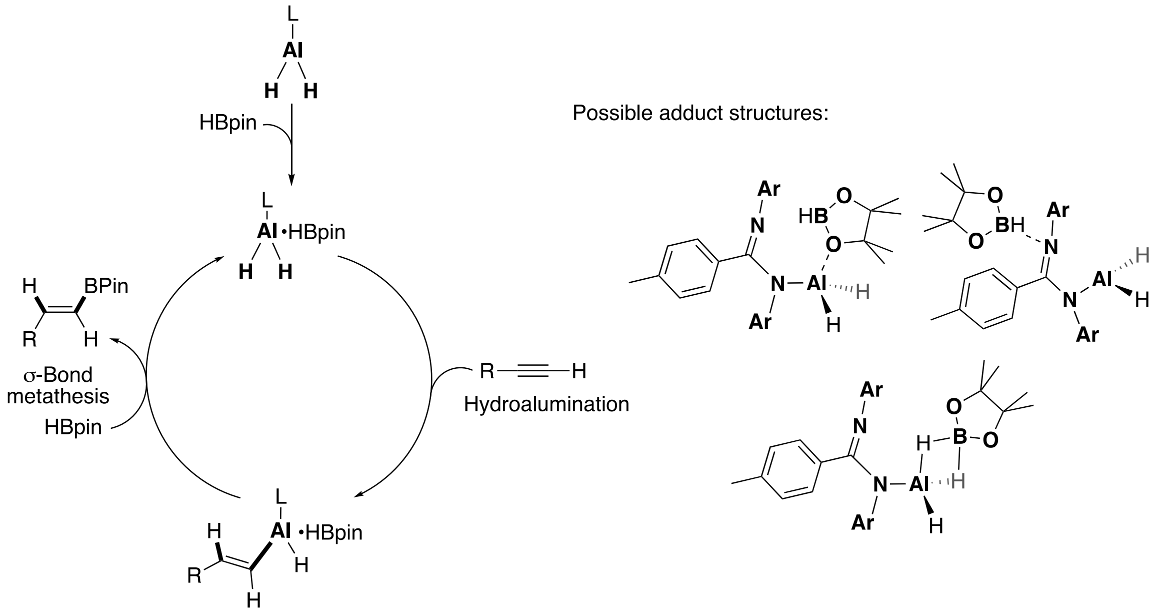

We propose an aluminium dihydride-HBpin adduct to be the active catalyst for the hydroboration of terminal alkynes with HBpin. The precise nature of the adduct (and subsequent reaction steps) are currently under further investigation. No hydrogen evolution is observed in the in situ ^{1}H NMR spectra indicating that the reaction may proceed via a hydroalumination type pathway.

5. X-ray Crystallography Data

All crystals were ran on a Agilent Oxford Diffraction SuperNova equipped with a microfocus Cu Kα X-ray source and an Atlas CCD detector. Full spheres of data were collected to 0.84 Å resolution with each 1° scan frame in ω collected twice. Total collection time varied depending on size and quality of crystal, and sample temperature. The Cryojet5® used for these measurements is the original prototype device developed by Oxford Instruments and the Pt-resistance sensor is located in the copper-block heat exchanger and not in the nozzle of the instrument close to the sample (in contrast to the CryojetHT® used in the PXRD experiments). Thus the temperatures quoted in these SXD experiments should be treated as nominal (despite stability to much better than 0.1 °C). Using Olex2,[4] the structure was solved with the ShelXT[5] structure solution program using Intrinsic Phasing and refined with the ShelXL[6] refinement package using Least Squares minimisation.

Crystal data for Ar*Ar^{mes}AlH$_2$ (2):

$C_{100}H_{94}Al_2N_4$ M =1405.75, monoclinic, C2/c, a = 22.8043(2) Å, b = 12.75660(10) Å, c = 32.3911(3) Å, β = 104.1670(10)°, V = 9136.16(14) Å^3, Z = 4, T = 150.00(10) K, μ(CuKα) = 0.621 mm^{-1}, $\rho_{calc}g/cm^3$ = 1.022, 70840 reflections measured (8.002° ≤ 2Θ ≤ 145.398°), 9021 unique (R_{int} = 0.0821, R_{sigma} = 0.0342) which were used in all calculations. The final R_1 was 0.0512 (I > 2σ(I)) and wR_2 was 0.1505 (all data), 507 parameters. CCDC: 2054525.

Single crystals of **2** were grown from a benzene/hexane solution. **2** was found to crystallise in a *C*2/c space group. The structure of **2** was found to be disordered, with the mesityl group split over two positions which had both major (78.8%) and minor occupancy (21.2%). The thermal parameters of adjacent atoms in the major and minor components of the disordered fragment were then restrained to be similar. The atoms in the minor component were refined isotropically and the atoms in the major component were refined anisotropically. The asymmetric unit also contained a disordered molecule of hexane and one of benzene, which were modelled using a solvent mask (SQUEEZE).

Crystal data for Ar*Ar^{dipp}AlH$_2$ (3):

$C_{130}H_{130}Al_2N_4$, M =1802.33, orthorhombic, Aea2, a = 27.8353(8) Å, b = 20.6582(6) Å, c = 18.2376(5) Å, V = 10487.1(5) Å^3, Z = 4, T = 151(1) K, μ(CuKα) = 0.645 mm^{-1}, ρ_{calc}g/cm^3 = 1.142, 38212 reflections measured (7.204° ≤ 2Θ ≤ 145.436°), 10133 unique (R_{int} = 0.0188, R_{sigma} = 0.0150) which were used in all calculations. The final R_1 was 0.0287 (I > 2σ(I)) and wR_2 was 0.0750 (all data), 627 parameters. CCDC: 2054527.

Single crystals of **3** were grown from a benzene solution. **3** was found to crystallise in a *Aea2* space group.

Crystal data for Ar*Ar*AlH$_2$ (4):

$C_{74}H_{63}AlN_2$, M =1007.24, triclinic, P-1, a = 11.7022(2) Å, b = 13.6375(3) Å, c = 19.3373(4) Å, α = 74.497(2)°, β = 78.438(2)°, γ = 76.750(2)°, V = 2862.54(11) Å^3, Z = 2, T = 158.5(9) K, μ(CuKα) = 0.647 mm^{-1}, ρ_{calc}g/cm^3 = 1.169, 51500 reflections measured (6.846° ≤ 2Θ ≤ 145.582°), 11136 unique (R_{int} = 0.0294, R_{sigma} = 0.0191) which were

used in all calculations. The final R_1 was 0.0377 (I > 2σ(I)) and wR_2 was 0.1451 (all data), 913 parameters. CCDC: 2054529.

Single crystals of **4** were grown from a benzene/hexane solution. **4** was found to crystallise in a *P-1* space group.

Crystal data for amine-Ar*Ar^mes^AlH$_2$ (2'):

$C_{53}H_{56}AlN_3$, M =761.98, triclinic, P-1, a = 9.7344(2) Å, b = 12.4989(3) Å, c = 18.3934(4) Å, α = 80.575(2)°, β = 86.868(2)°, γ = 85.991(2)°, V = 2200.18(9) Å^3, Z = 2, T = 150.2(7) K, μ(CuKα) = 0.685 mm^{-1}, r_{calc}g/cm^3 = 1.150, 33455 reflections measured (7.184° ≤ 2Θ ≤ 145.424°), 8526 unique (R_{int} = 0.0291, R_{sigma} = 0.0225) which were used in all calculations. The final R_1 was 0.0395 (I > 2σ(I)) and wR_2 was 0.1469 (all data), 727 parameters. CCDC: 2054528.

Single crystals of **2'** were grown from a benzene/hexane solution. **2'** was found to crystallise in a *P-1* space group.

Crystal Data for Ar*Ar^{dipp}LAlMe$_2$ (5):

$C_{61}H_{71}AlN_2$ (M =859.17 g/mol): triclinic P-1, a = 11.8634(2) Å, b = 15.3910(4) Å, c = 15.5263(4) Å, α = 102.162(2)°, β = 109.645(2)°, γ = 92.471(2)°, V = 2589.94(11) Å^3, Z = 2, T = 149.9(4) K, μ(CuKα) = 0.625 mm^{-1}, ρ_{calc} g/cm^3 = 1.102, 41510 reflections measured (7.496° ≤ 2Θ ≤ 147.048°), 10269 unique (R_{int} = 0.0285, R_{sigma} = 0.0212) which were used in all calculations. The final R1 was 0.0513 (I > 2σ(I)) and wR2 was 0.1533 (all data), 587 parameters. CCDC: 2054526.

Single crystals of **5** were grown from a toluene/hexane solution. **5** was found to crystallise in a *P-1* space group. The asymmetric unit also contained a molecule of hexane.

Crystal data for (Ar*Ar^{mes})$_2$AlH (2''):

$C_{100}H_{91}AlN_4$ (M =1375.74 g/mol): monoclinic C2/c, a = 22.7932(3) Å, b = 22.7393(3) Å, c = 17.2859(2) Å, β = 92.9790(10)°, V = 8947.2(2) Å^3, Z = 4, T = 149.97(10) K, μ(CuKα) = 0.535 mm^{-1}, ρ_{calc}g/cm^3 = 1.021, 17676 reflections measured (7.648° ≤ 2Θ ≤ 145.338°), 8611 unique (R_{int} = 0.0228, R_{sigma} = 0.0261) which were used in all calculations. The final R_1 was 0.0397 (I > 2σ(I)) and wR_2 was 0.1117 (all data), 481 parameters. CCDC: 2054524.

Single crystals of **2''** were grown from a benzene/hexane solution. **2''** was found to crystallise in a *C2/c* space group. The asymmetric unit also contained two disordered molecules of hexane and one of benzene, which were modelled using a solvent mask (SQUEEZE).

Crystal data for Ar*Ar*Al(OC(CH$_3$)$_2$)$_2$ (7):

$C_{80}H_{73}AlN_2O_2$, M =1121.38, monoclinic, P2$_1$/c, a = 13.2305(2) Å, b = 22.8842(3) Å, c = 23.8160(3) Å, β = 103.2350(10)°, V = 7019.23(17) Å^3, Z = 4, T = 149.8(4) K, μ(CuKα) = 0.593 mm^{-1}, ρ_{calc}*g/cm^3* = 1.061, 51376

reflections measured (7.626° ≤ 2Θ ≤ 145.446°), 13771 unique (R_{int} = 0.0375, R_{sigma} = 0.0287) which were used in all calculations. The final R_1 was 0.0565 (I > 2σ(I)) and wR_2 was 0.1644 (all data), 773 parameters. CCDC: 2054530.

Single crystals of **7** were grown from a benzene/hexane solution. **7** was found to crystallise in a $P2_1/c$ space group. The asymmetric unit also contained three disordered molecules of benzene, which were modelled using a solvent mask (SQUEEZE).

6. Multinuclear NMR Data

Figure S55: ^{1}H NMR spectrum of **L1**

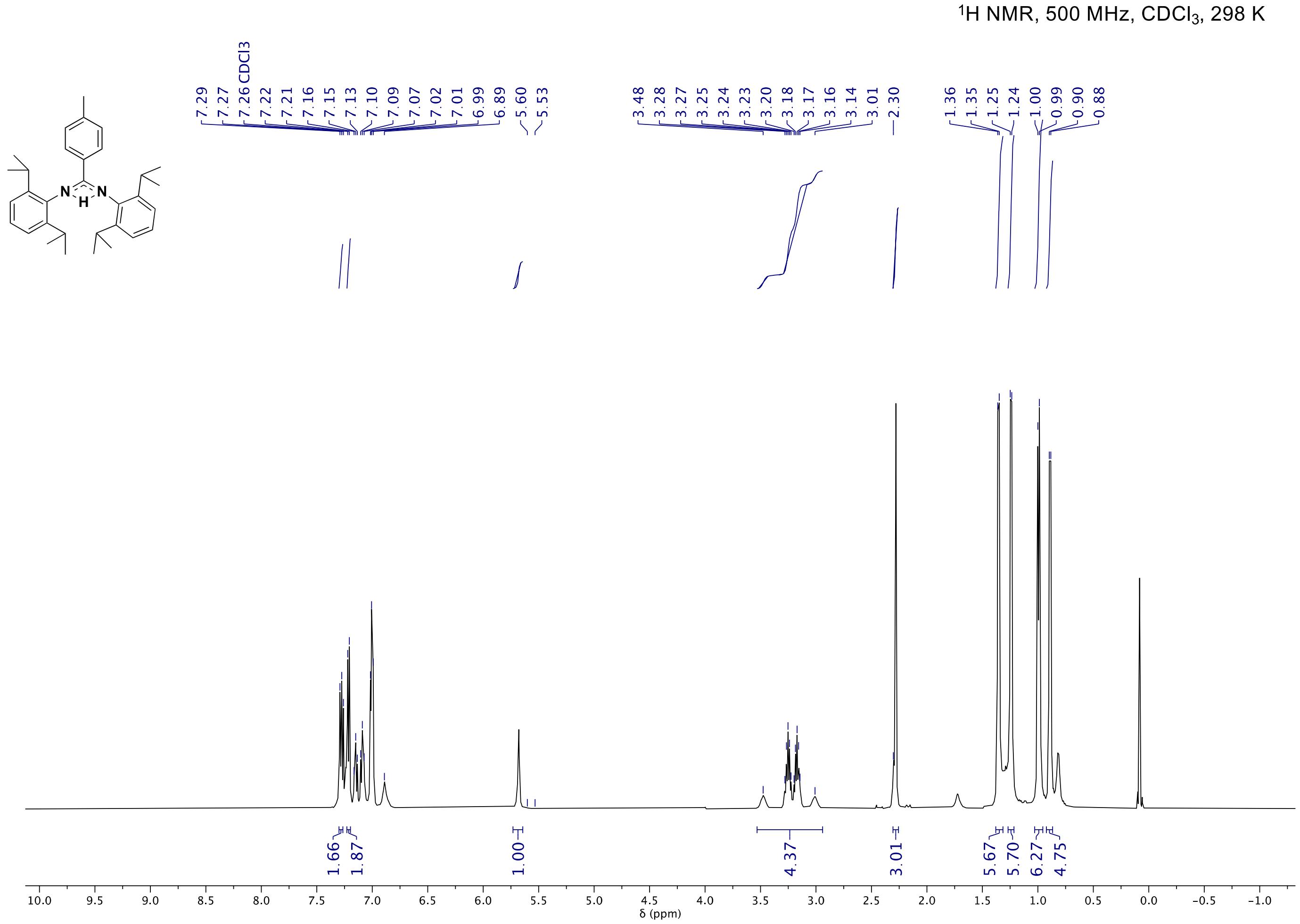

Figure S56: ^{13}C NMR spectrum of **L1**

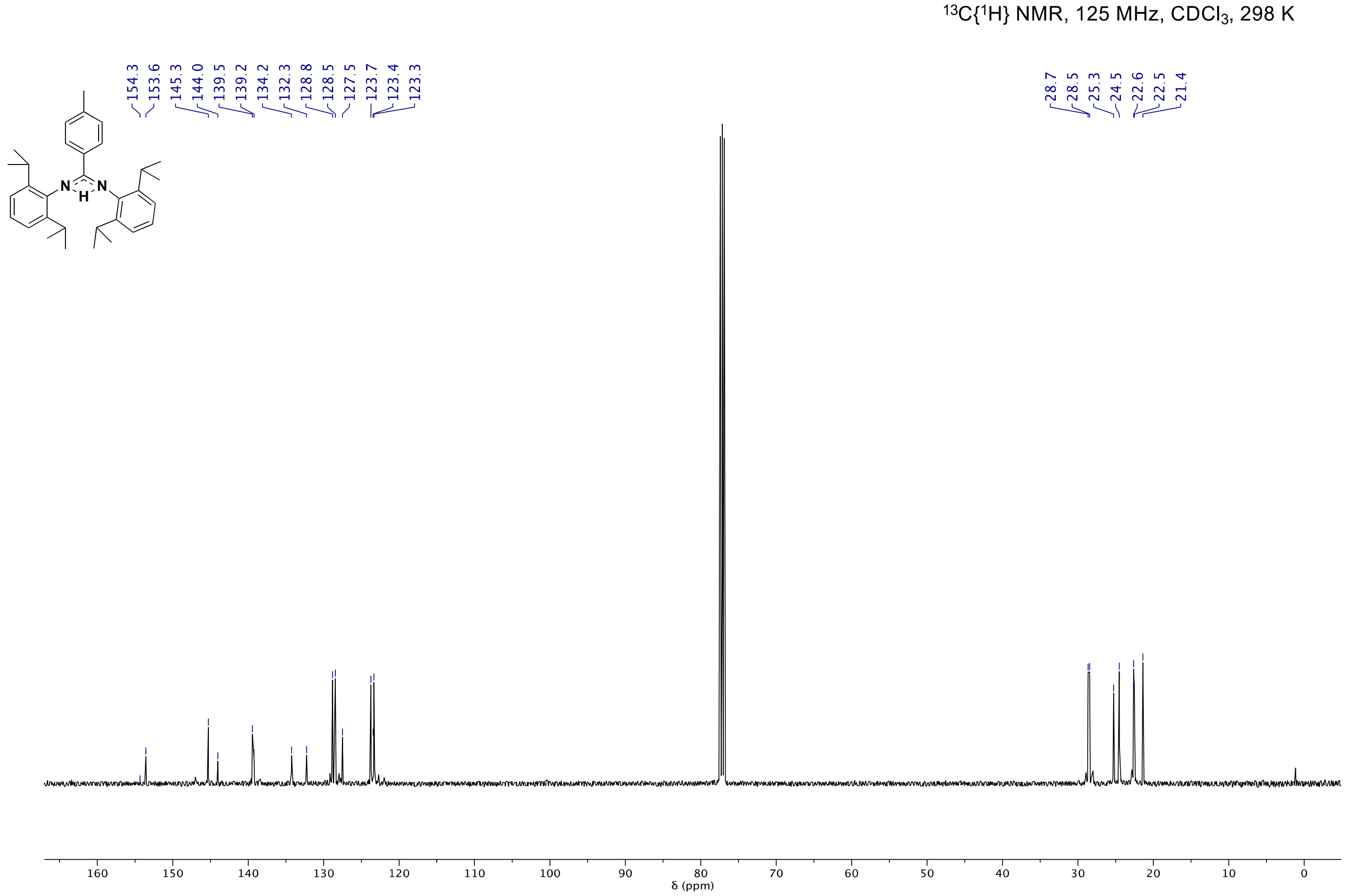

Figure S57: ^{1}H NMR spectrum of **L2**

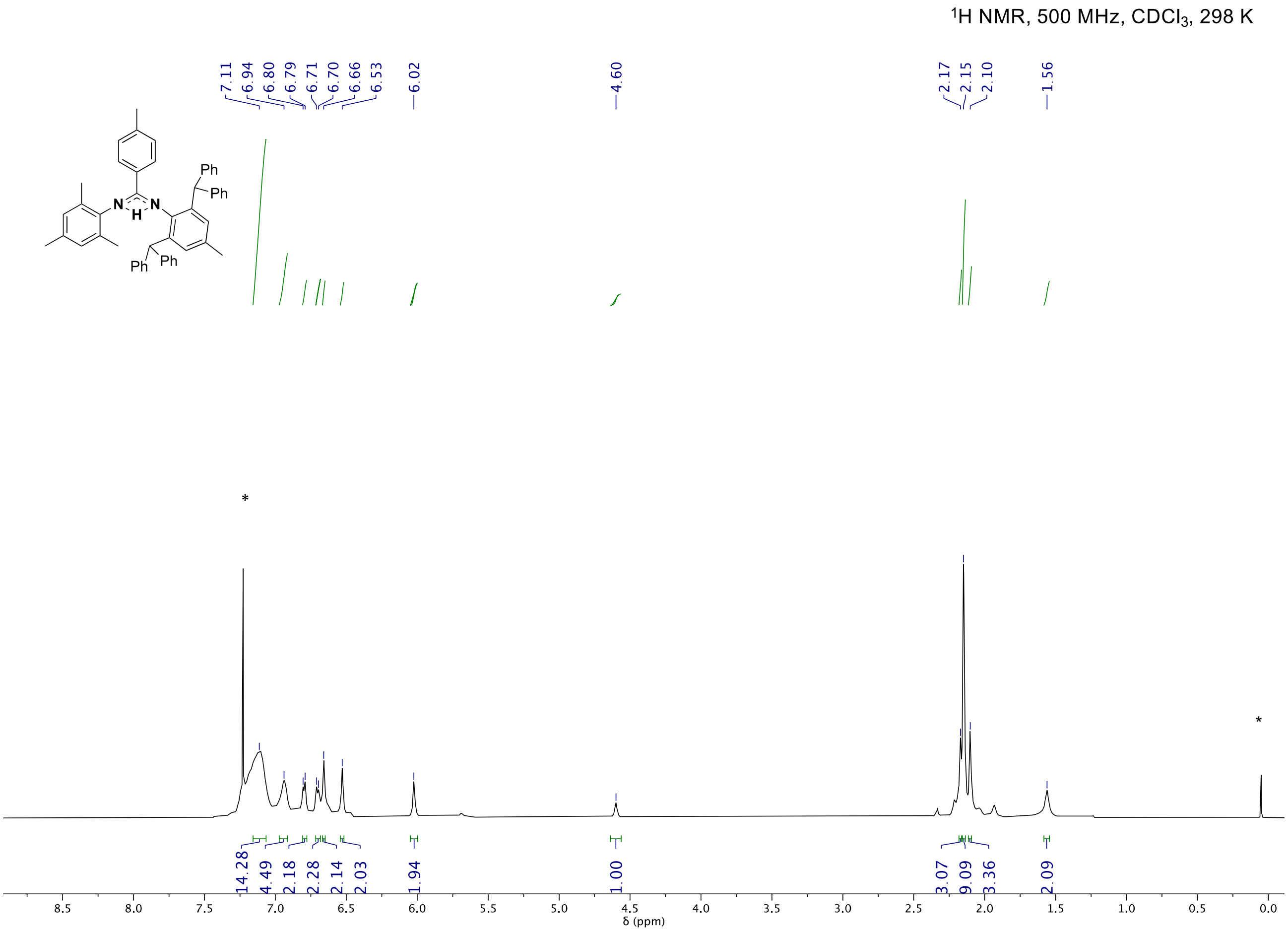

Figure S58: ^{13}C NMR spectrum of **L2**

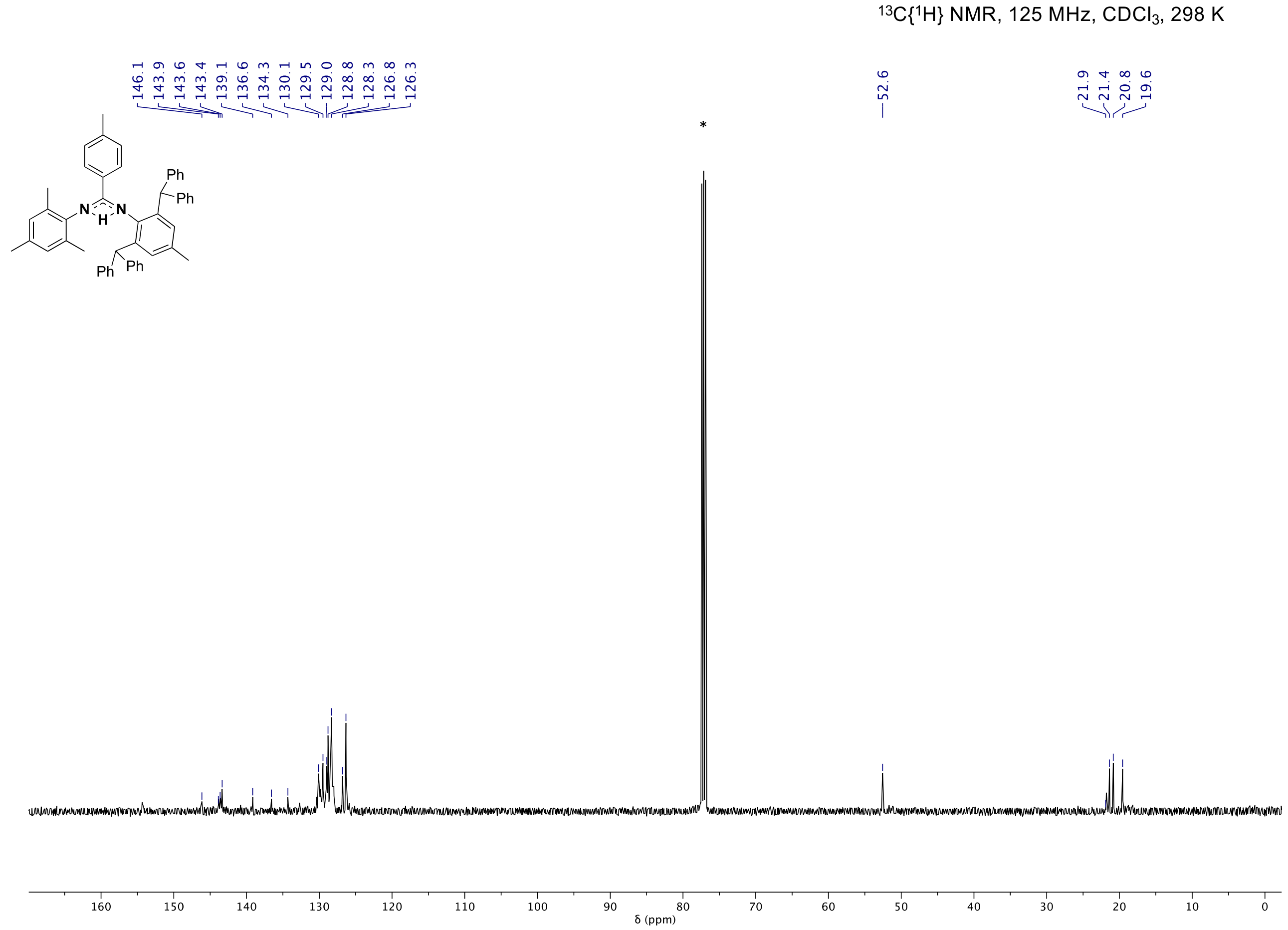

Figure S59: ^{1}H NMR spectrum of **L3**

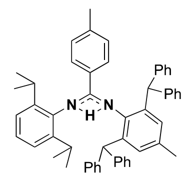

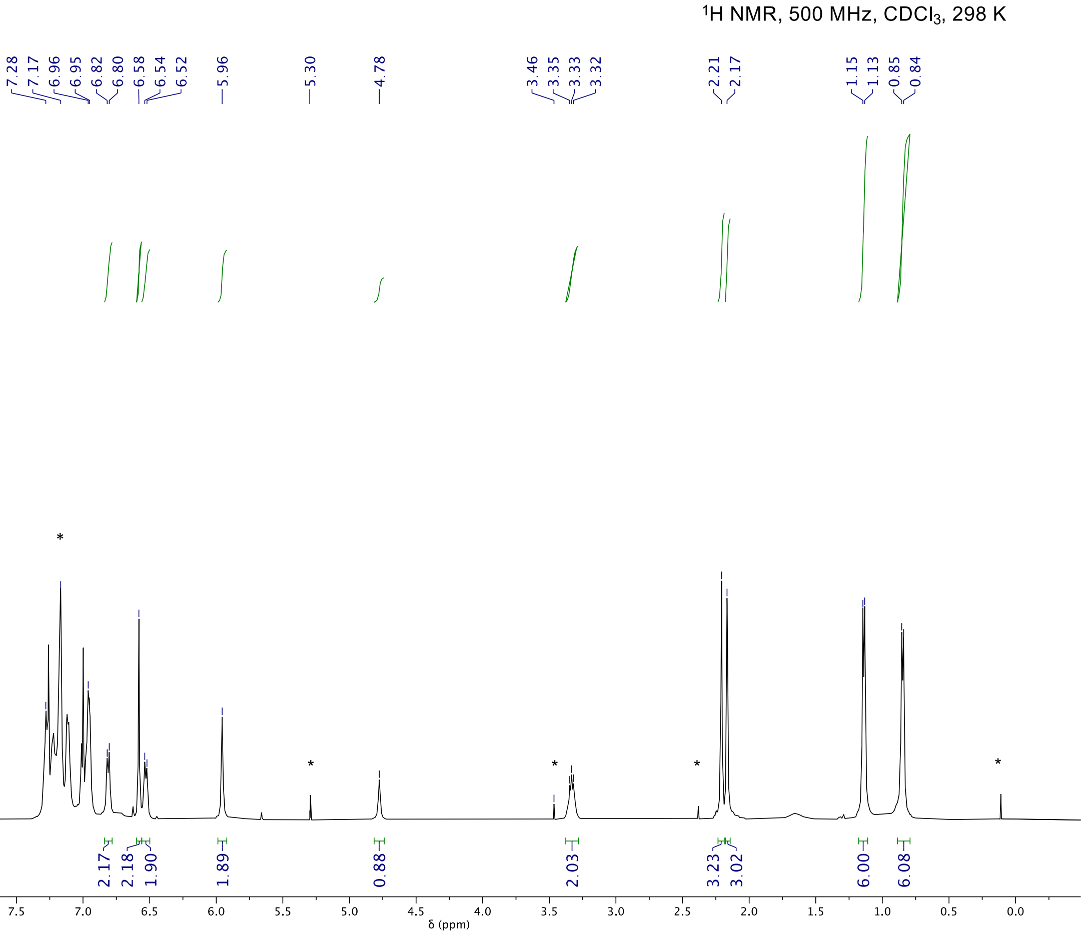

Figure S60: ^{13}C NMR spectrum of **L3**

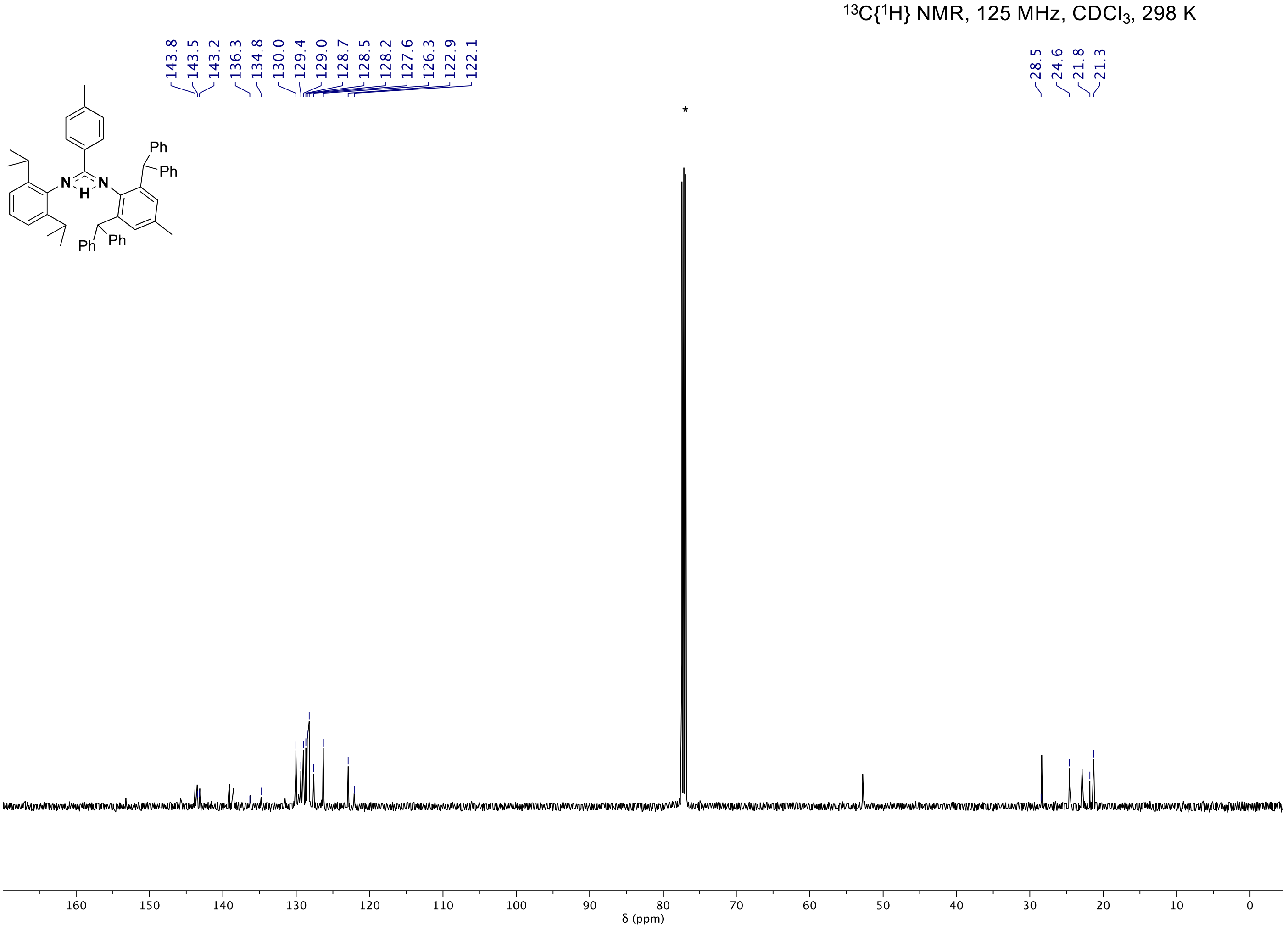

Figure S61: ^{1}H NMR spectrum of **L4**

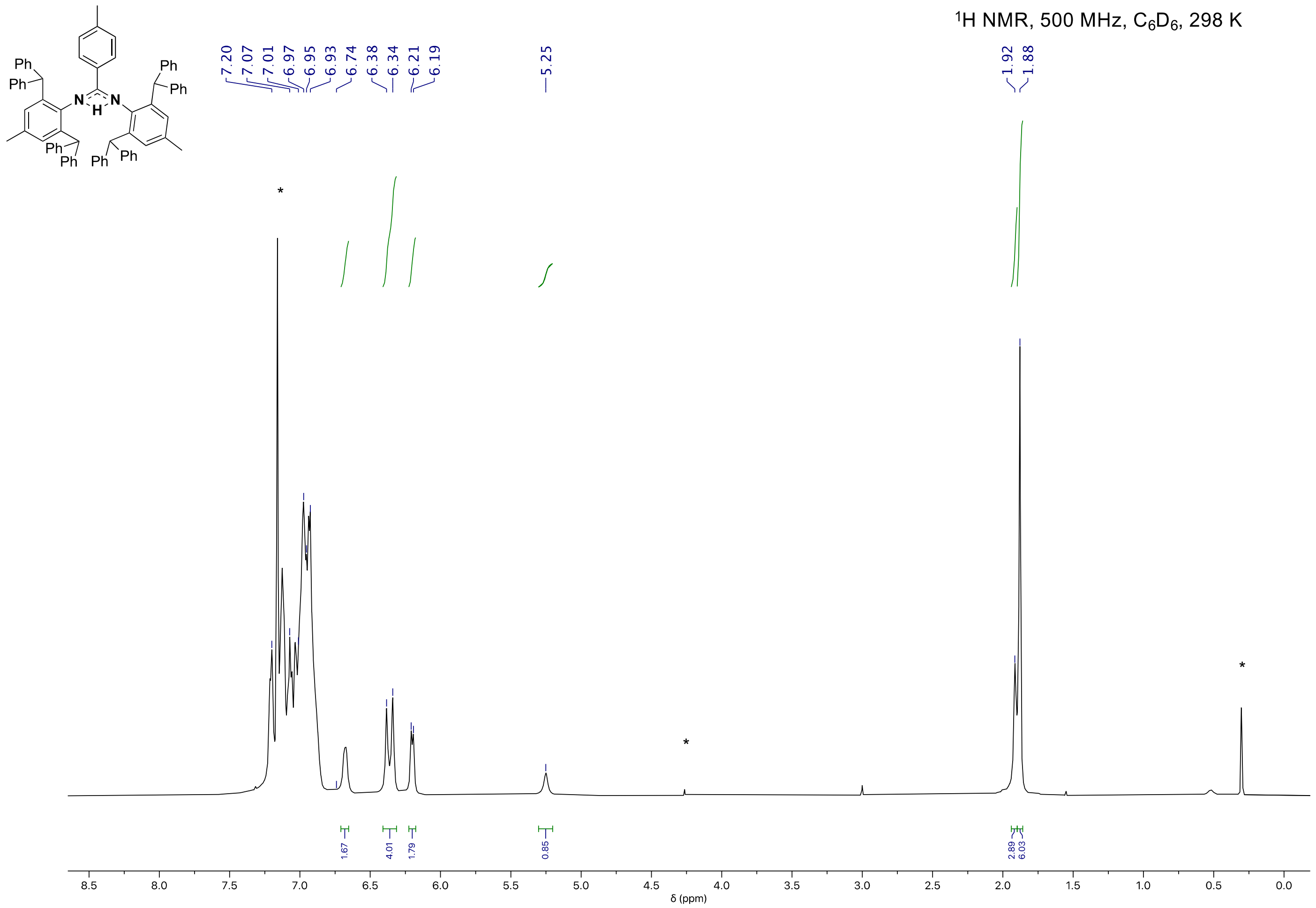

Figure S62: ^{13}C NMR spectrum of **L4**

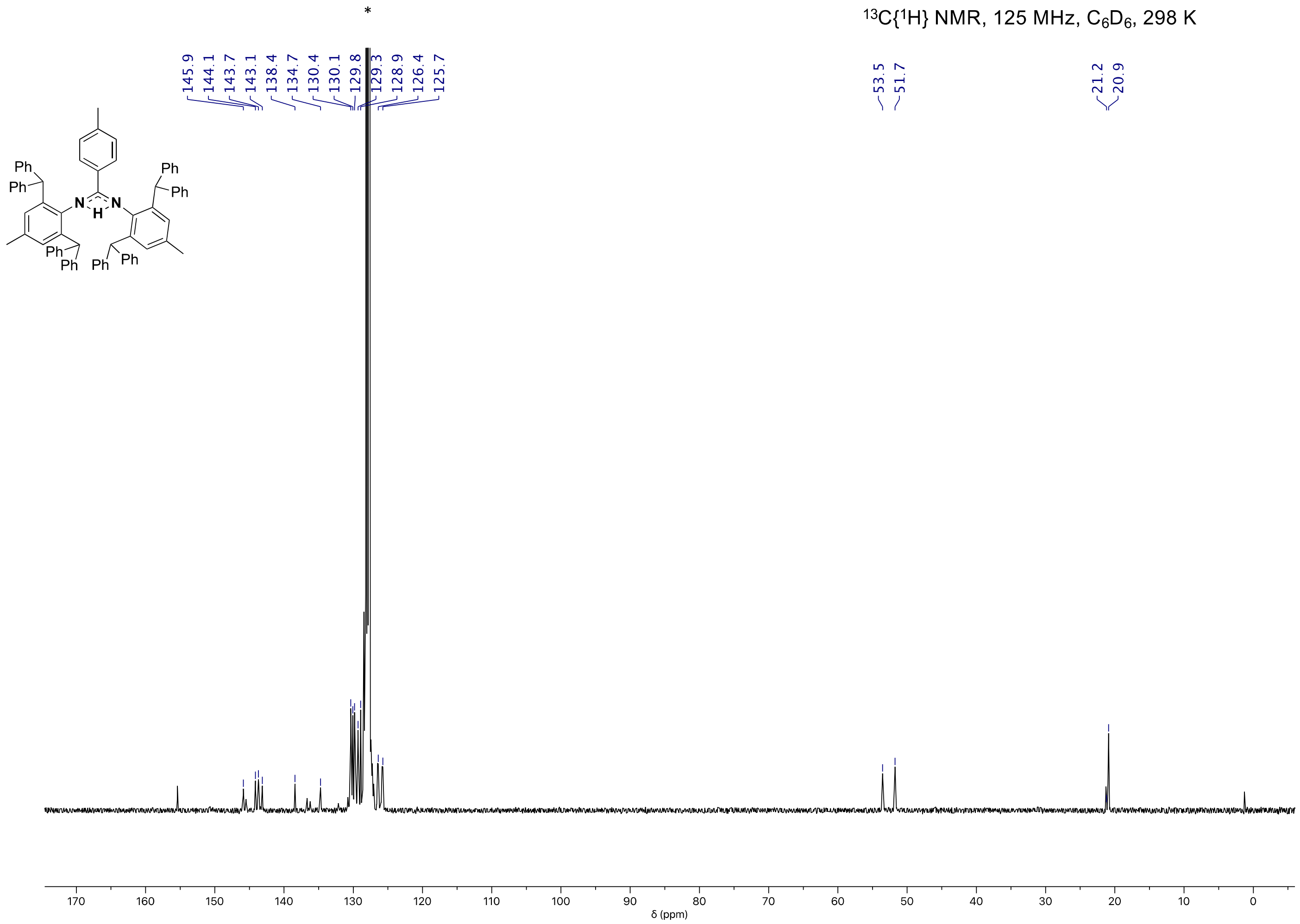

Figure S63: ^{1}H NMR spectrum of **1**

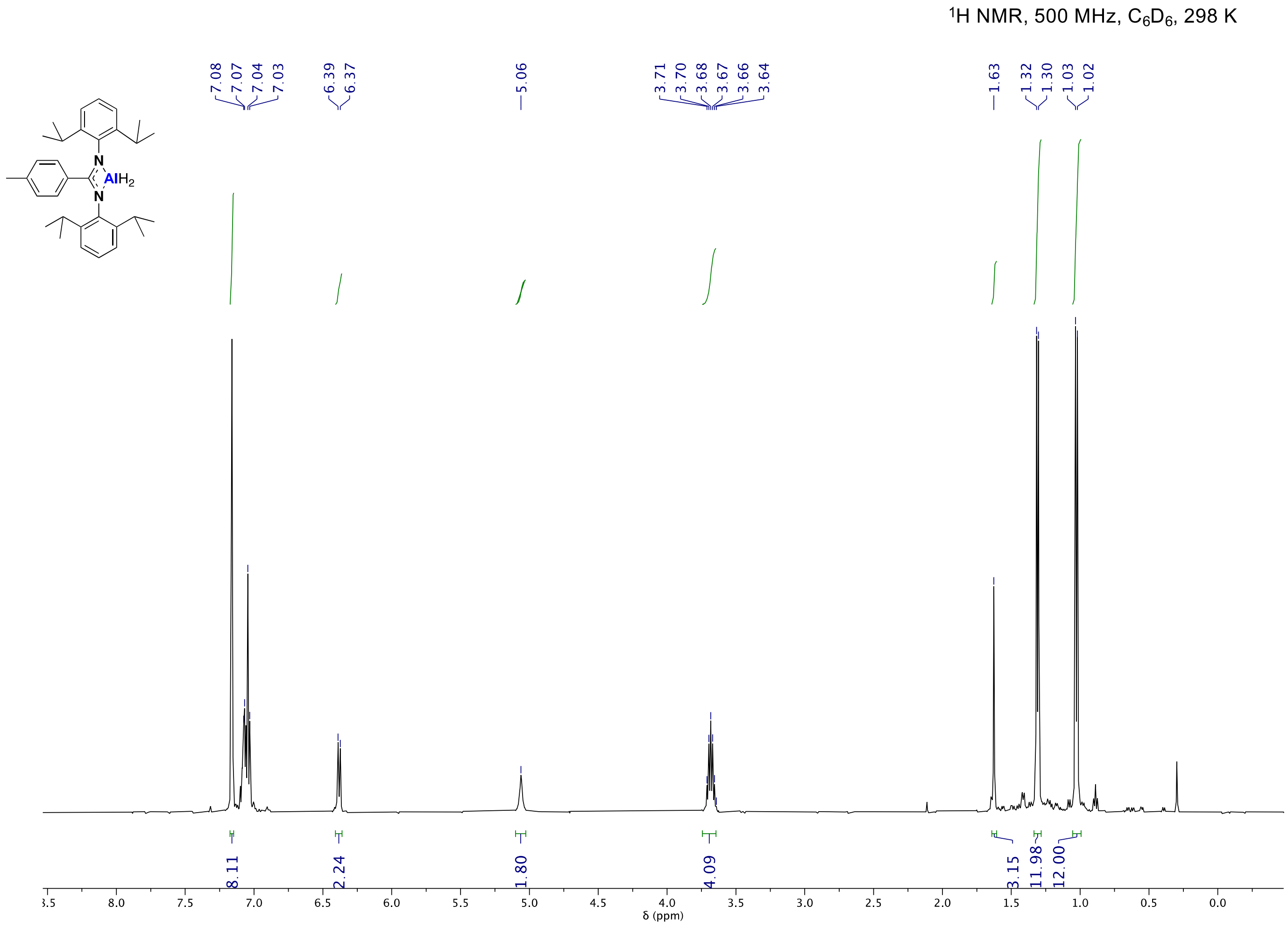

Figure S64: ^{13}C NMR spectrum of **1**

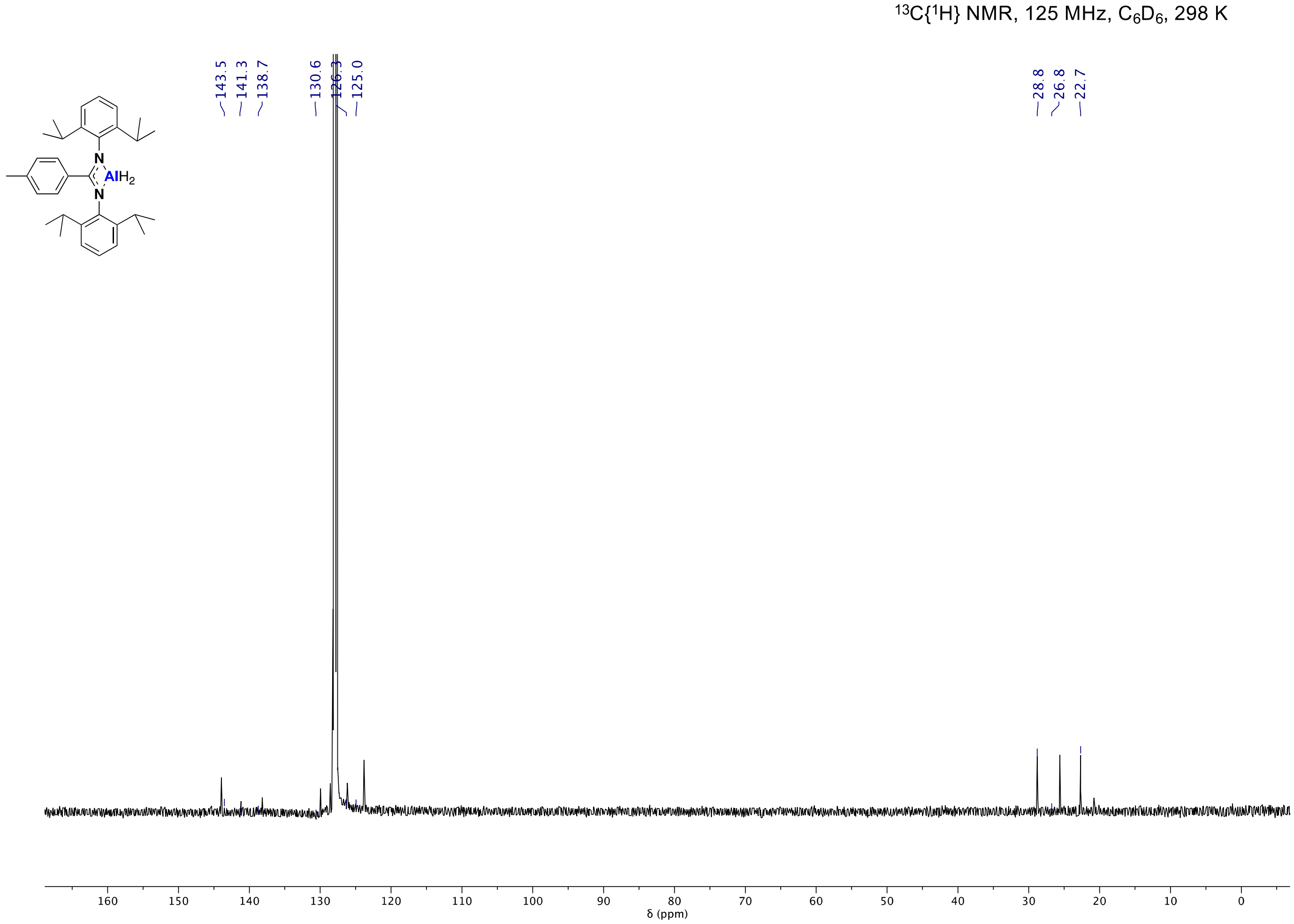

Figure S65: ^{1}H NMR spectrum of **2**

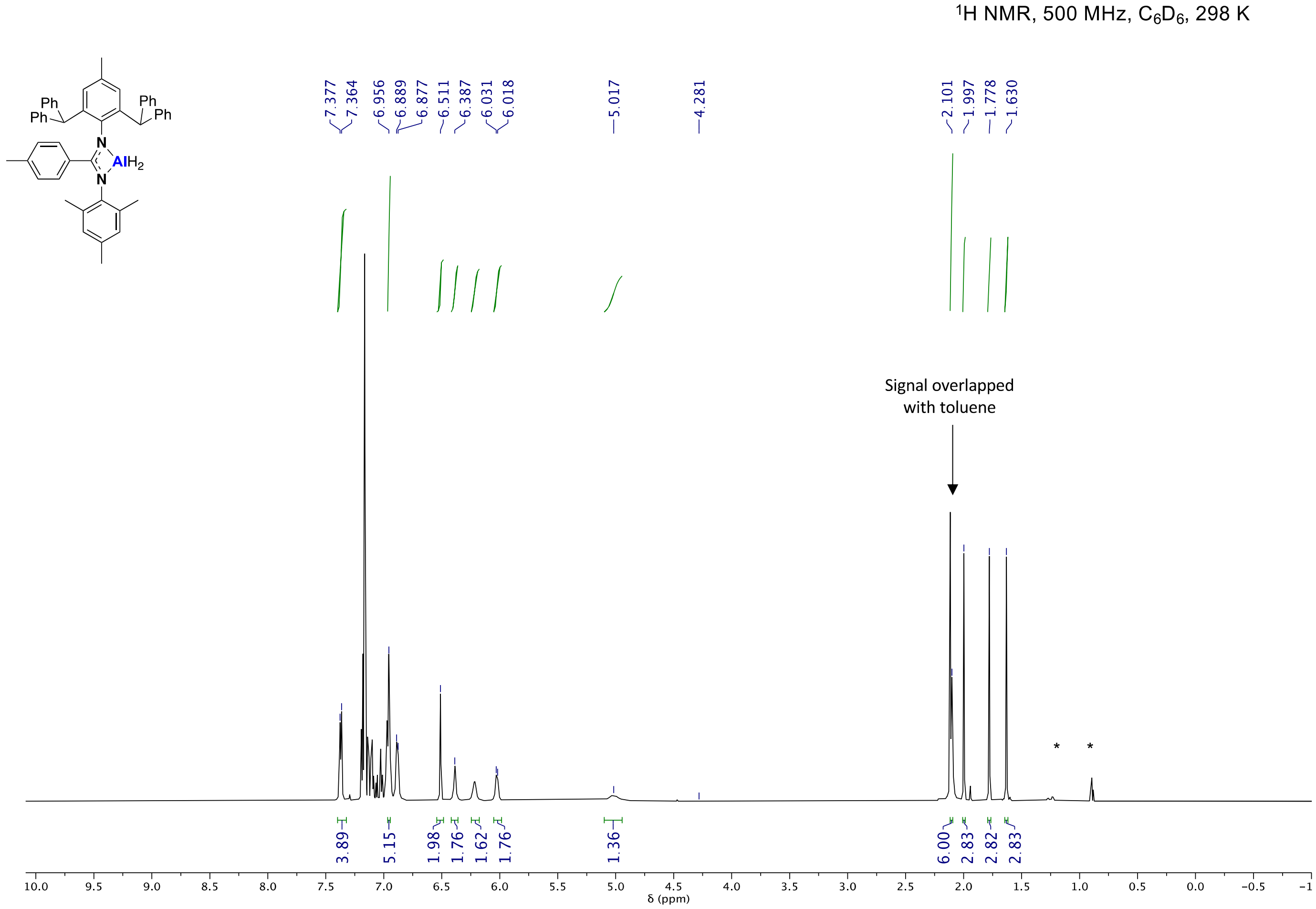

Figure S66: ^{13}C NMR spectrum of **2**

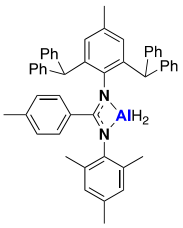

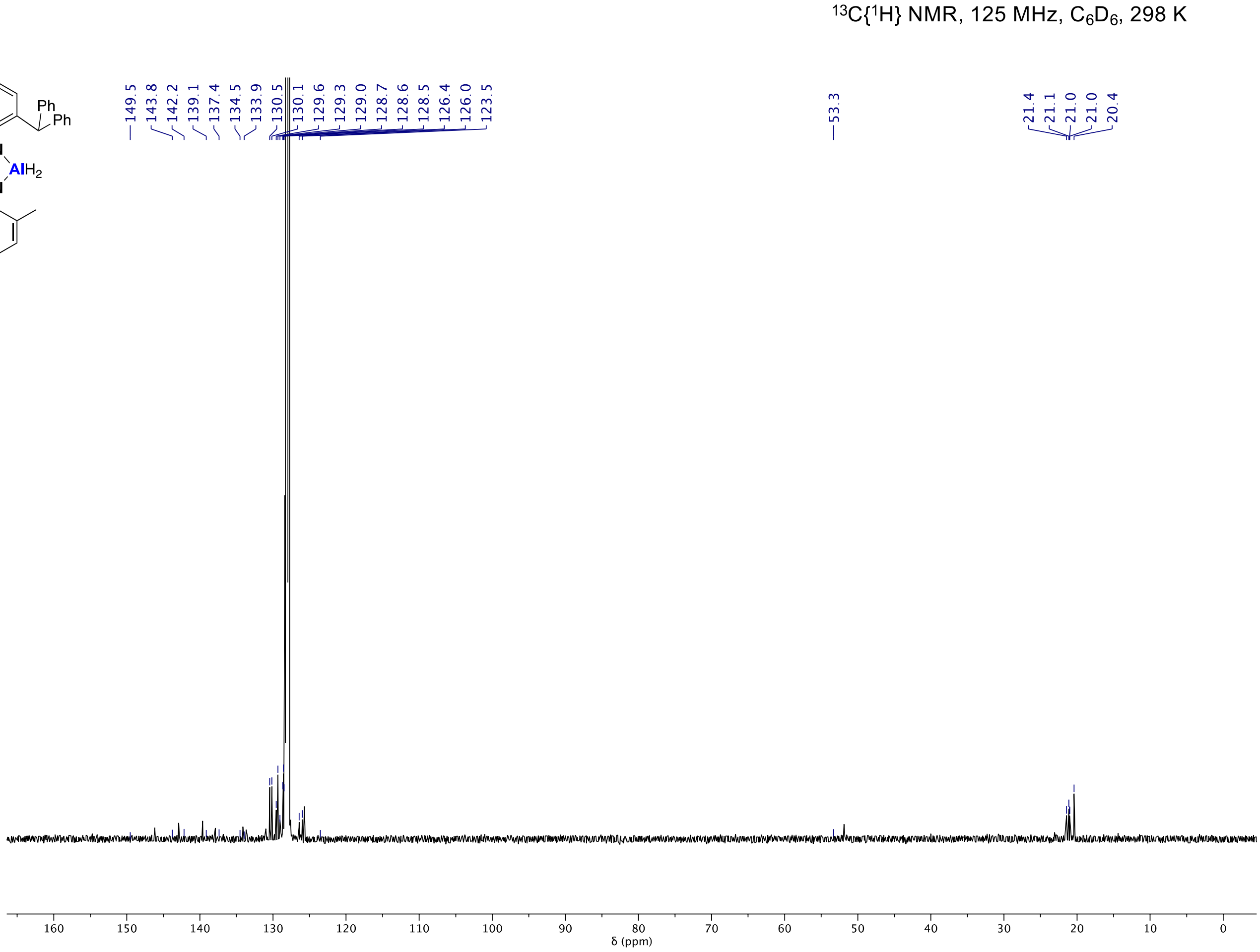

Figure S67: ^{1}H NMR spectrum of **3**

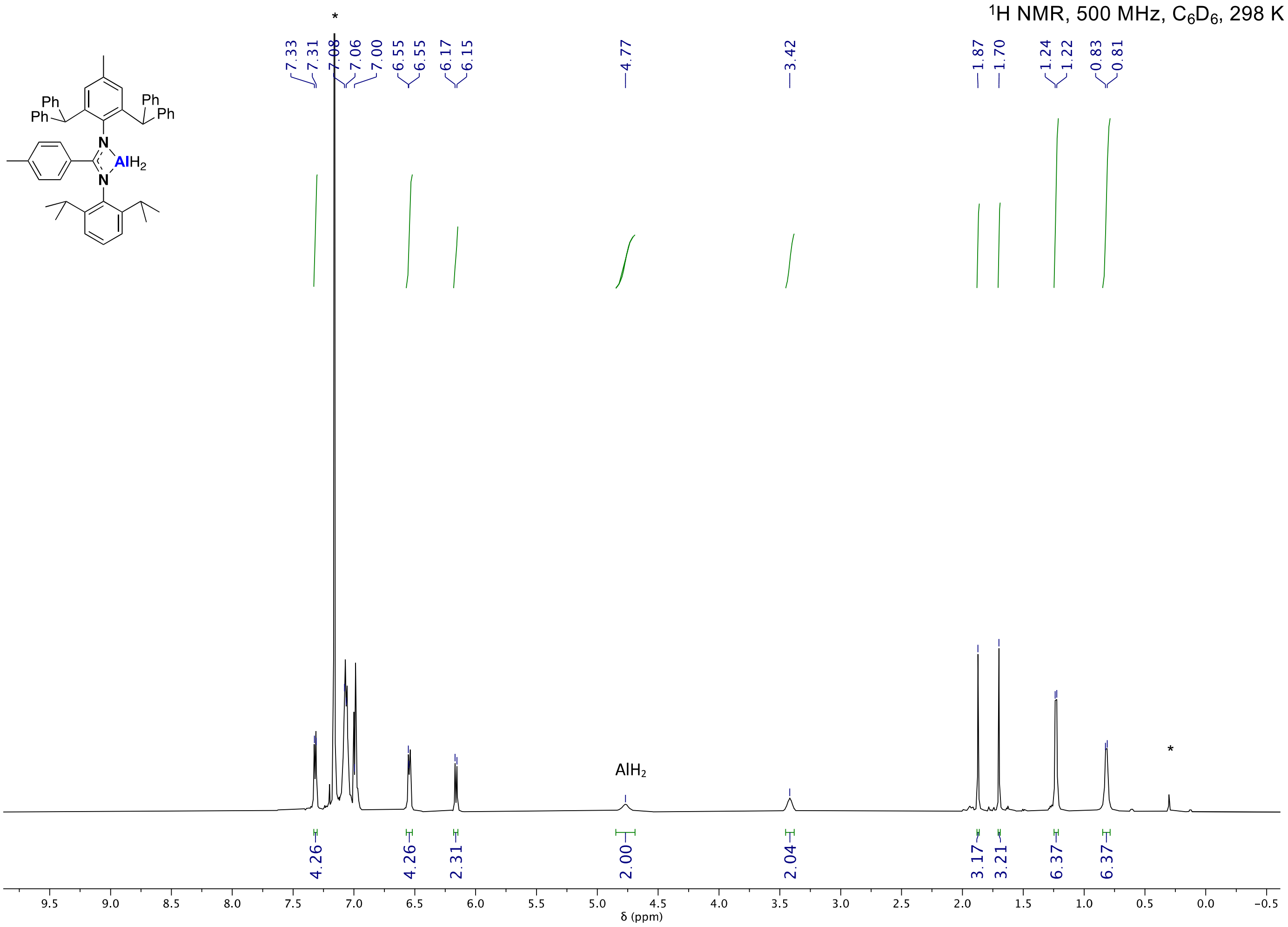

Figure S68: ^{13}C NMR spectrum of **3**

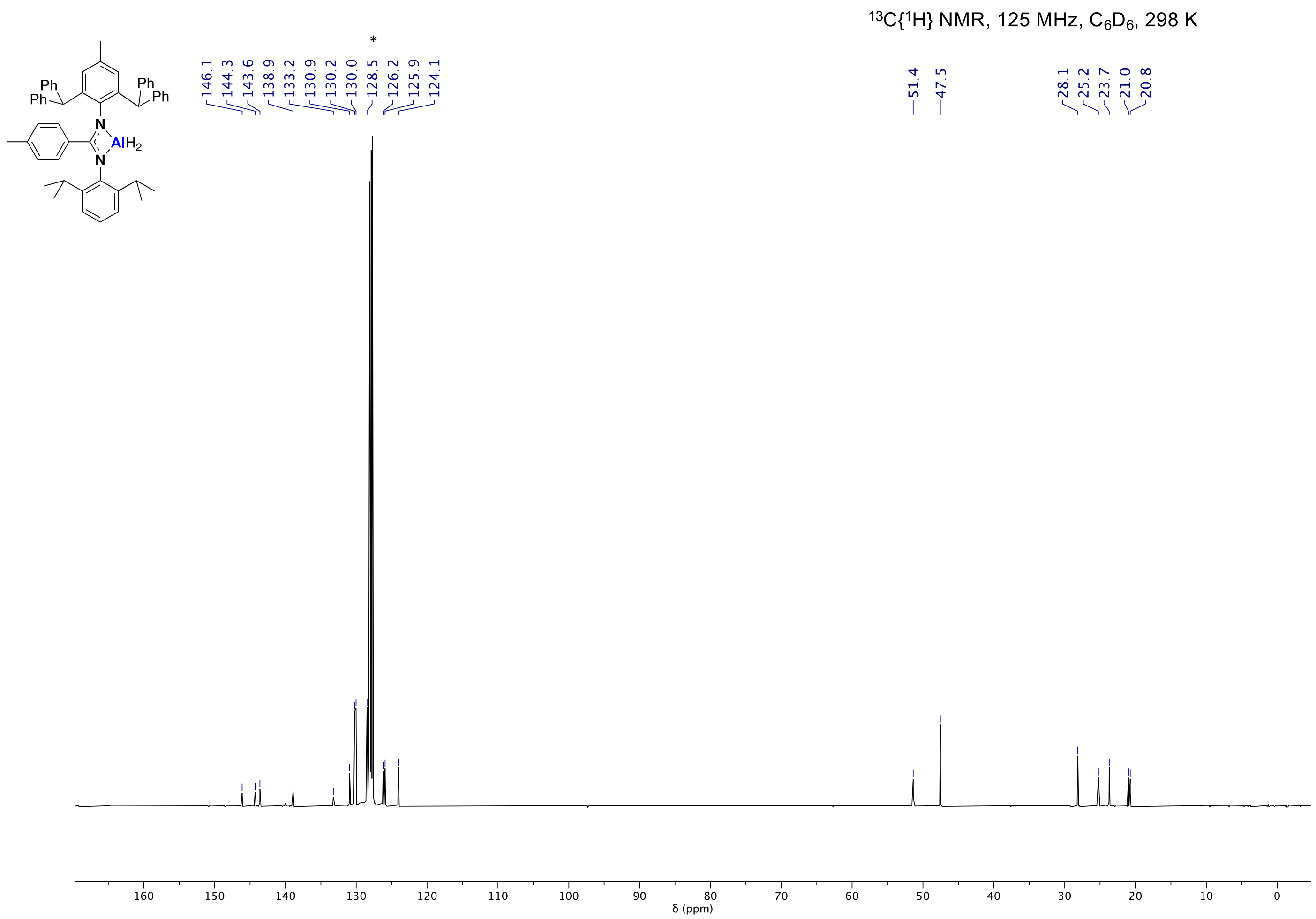

Figure S69: ^{13}C NMR spectrum of **4** (see Figure S2 for ^{1}H NMR spectrum)

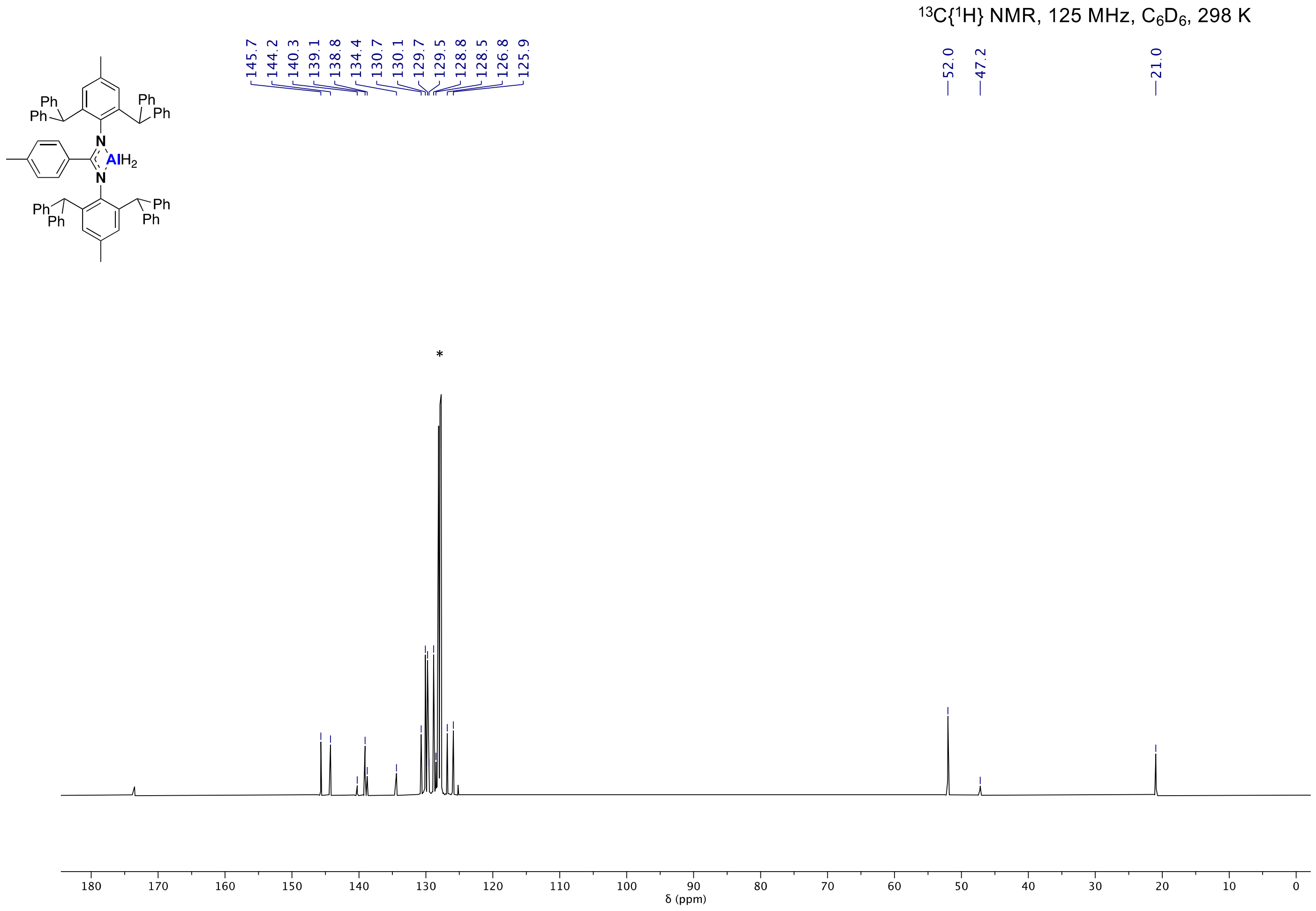

Figure S70: ^{1}H NMR spectrum of **2’**

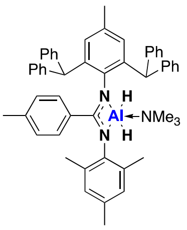

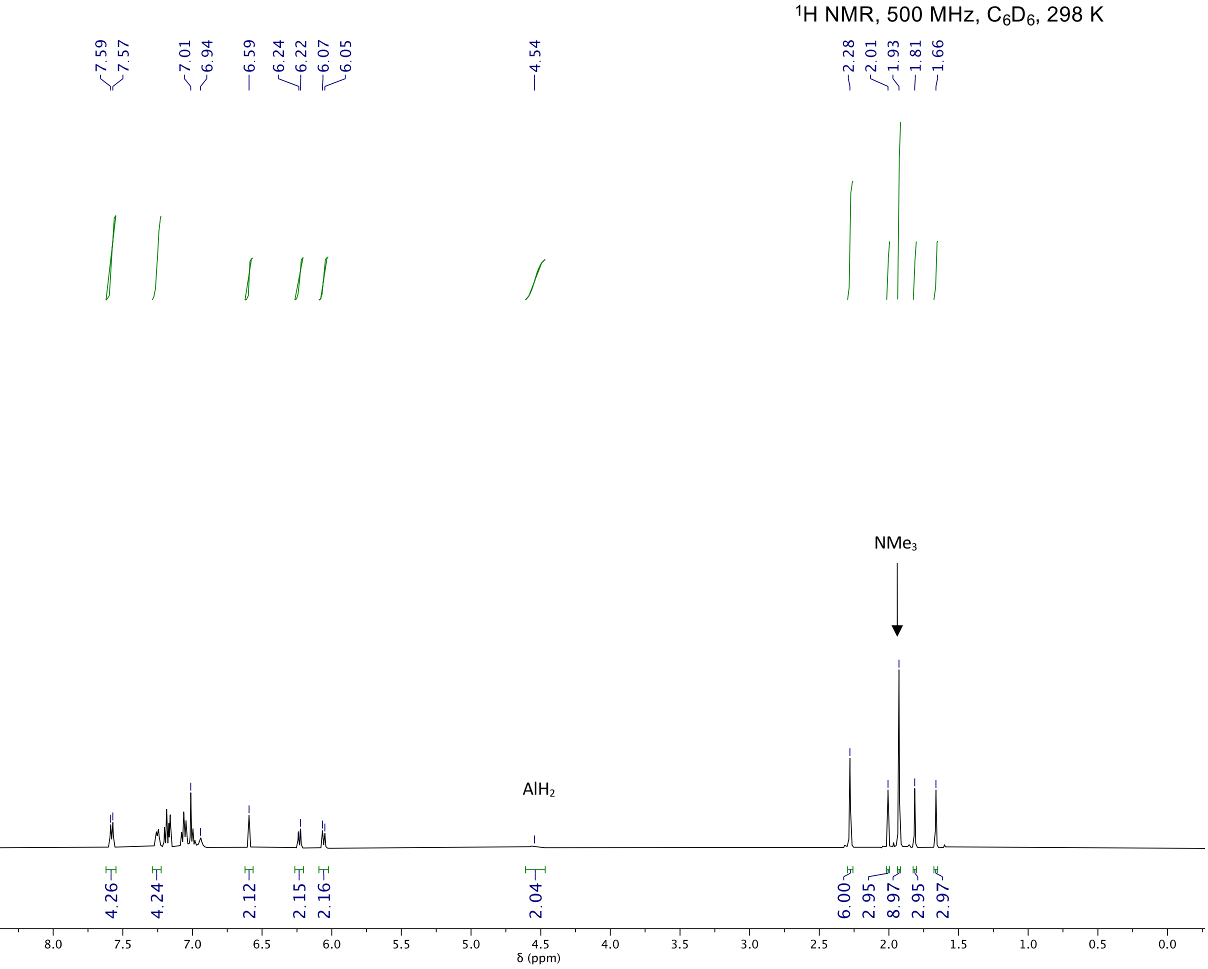

Figure S71: ^{13}C NMR spectrum of **2'**

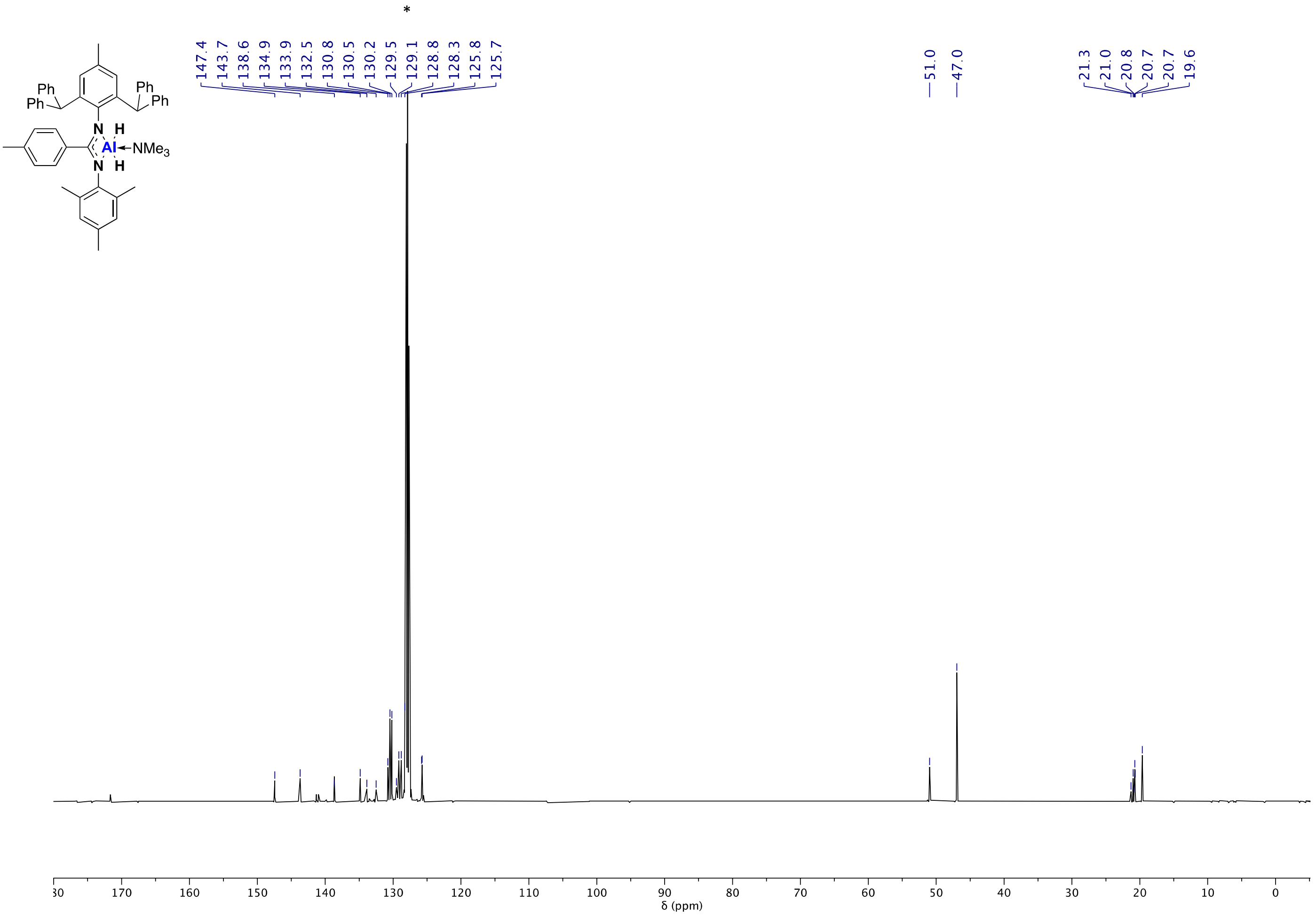

Figure S72: ^{1}H NMR spectrum of **2”**

^{1}H NMR, 500 MHz, C_6D_6, 298 and 363 K

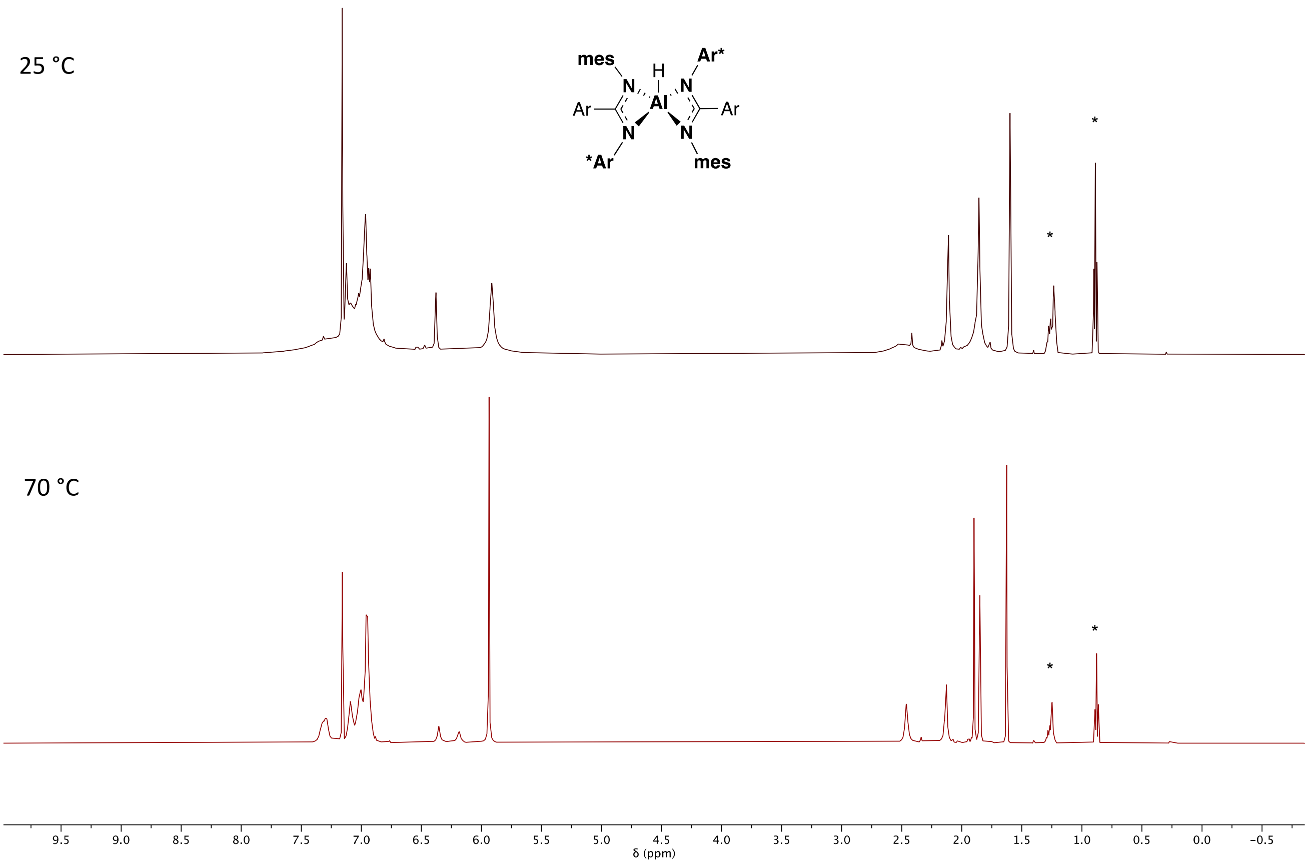

Figure S73: ^{13}C NMR spectrum of **2”**

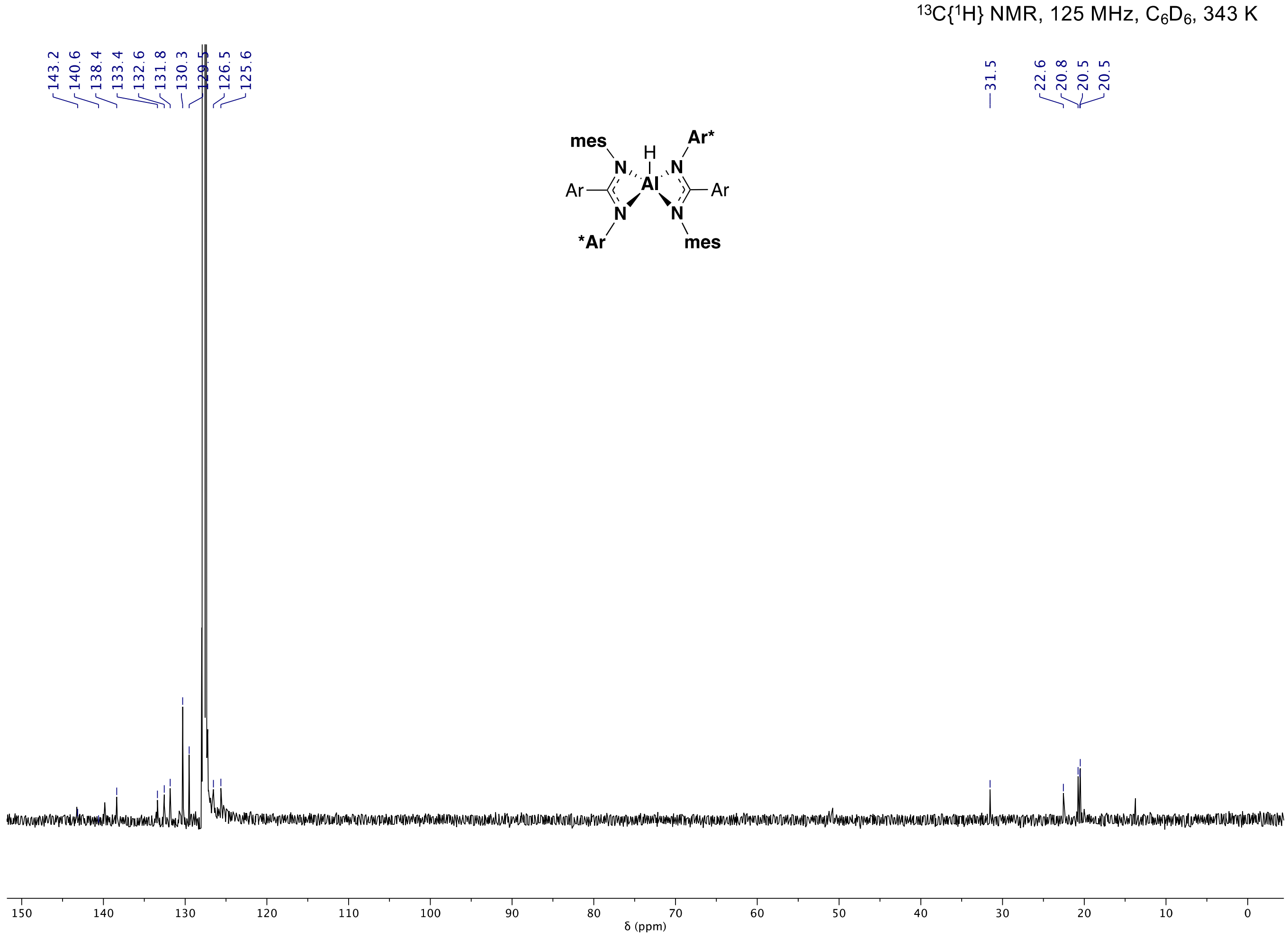

Figure S74: ^{1}H NMR spectrum of **5**

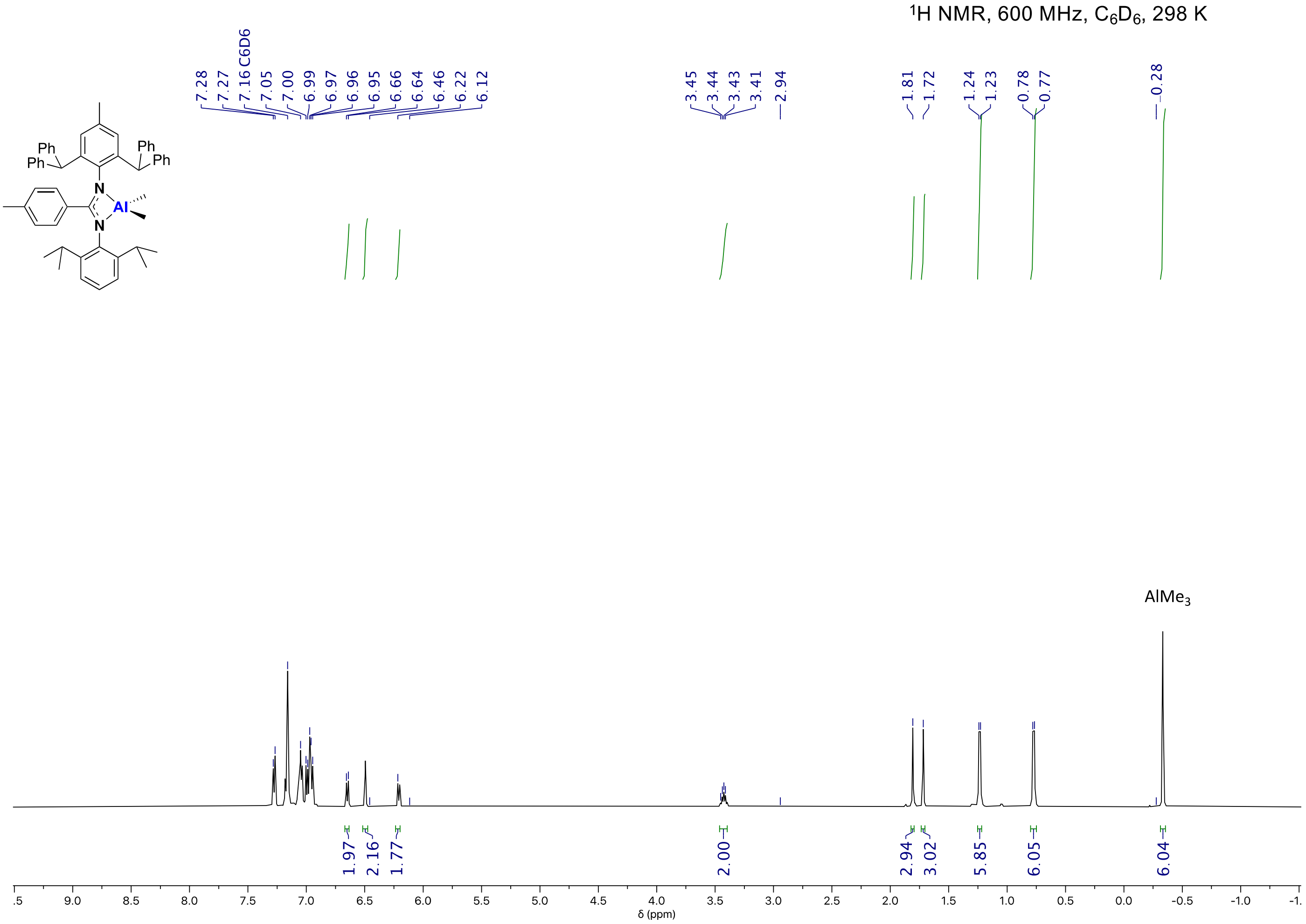

Figure S75: ^{13}C NMR spectrum of **5**

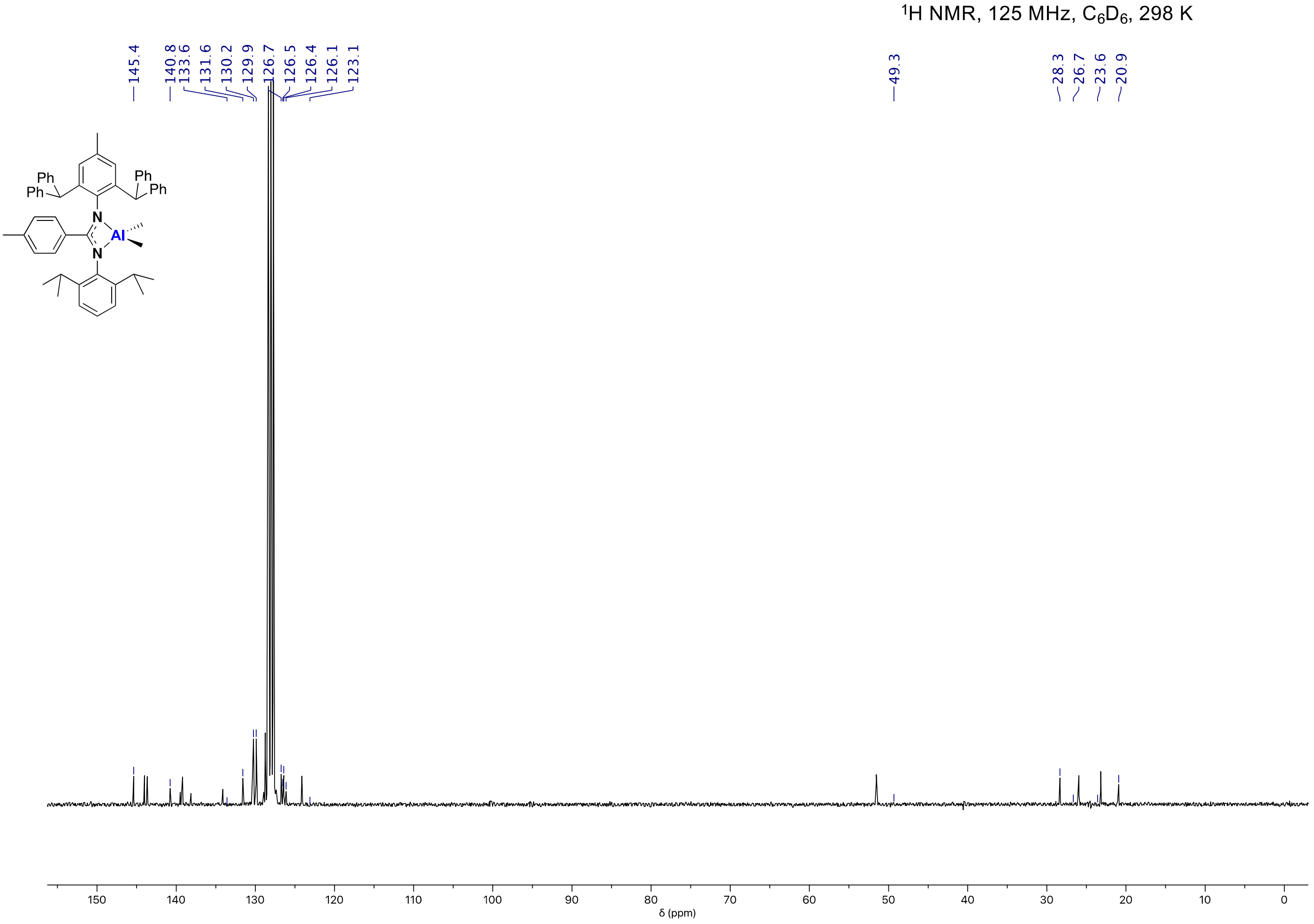

Figure S76: ^{1}H NMR spectrum of **6**

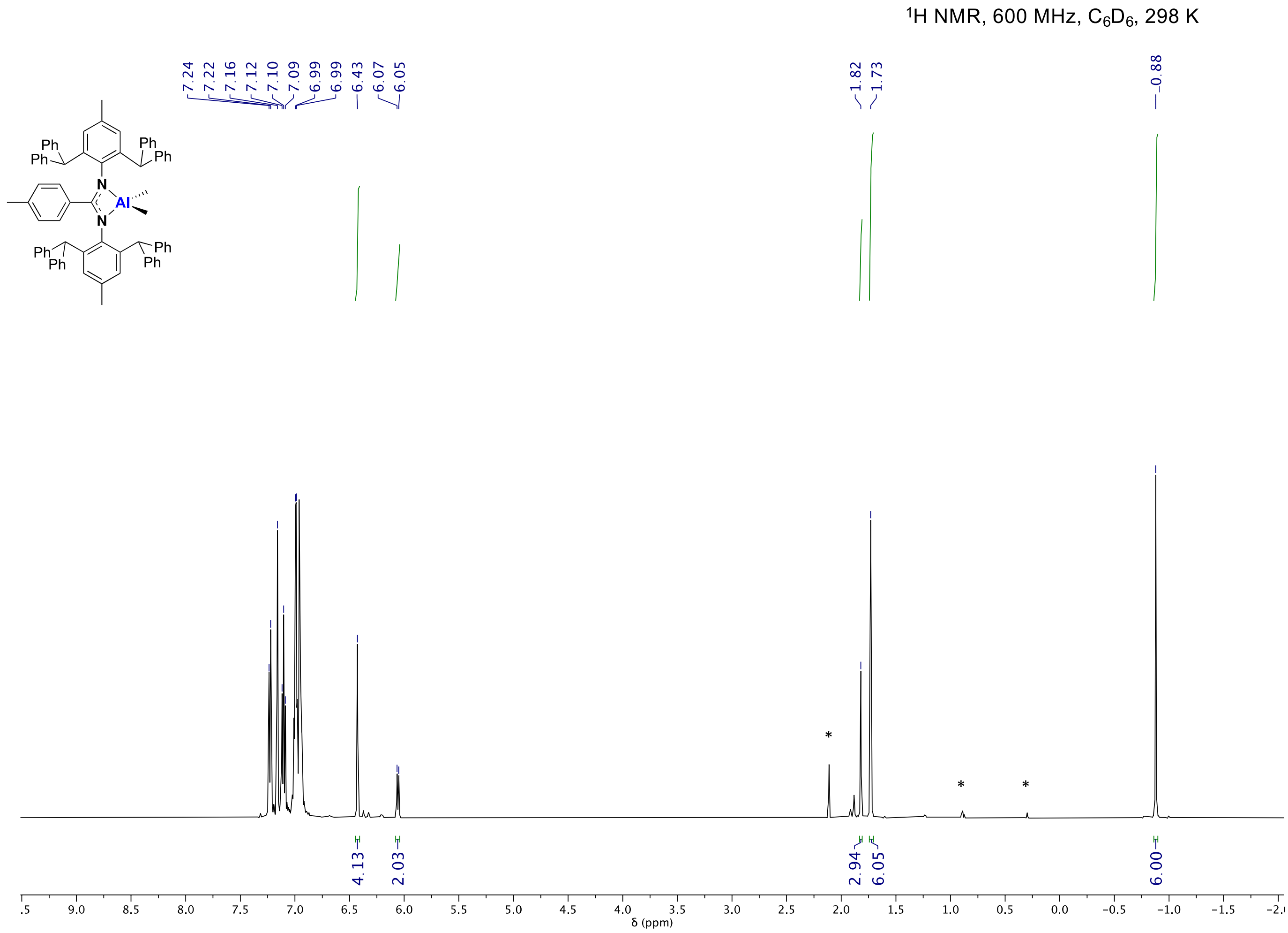

Figure S77: ^{1}H NMR spectrum of **6**

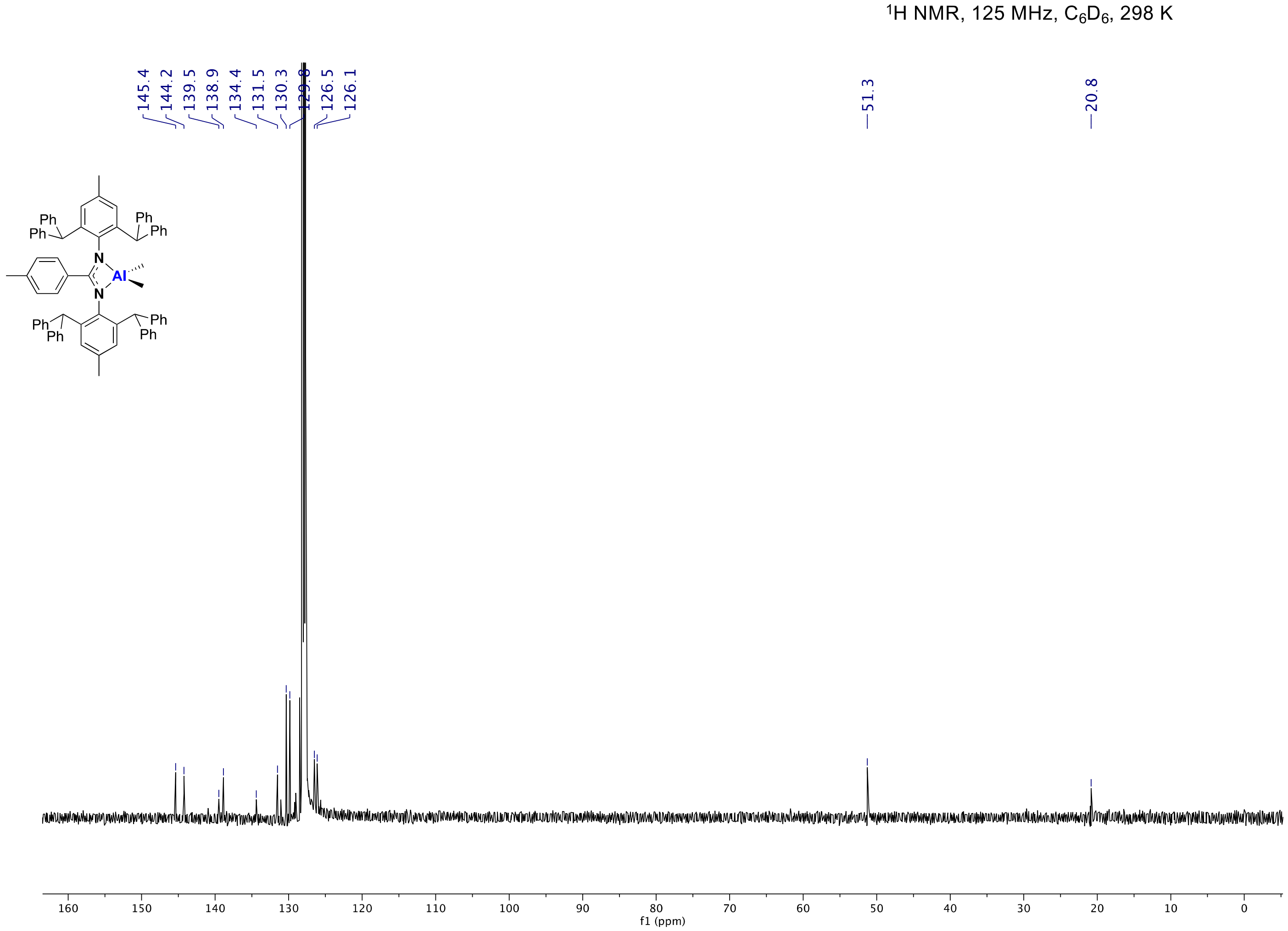

Figure S78: ^{1}H NMR spectrum of **7**

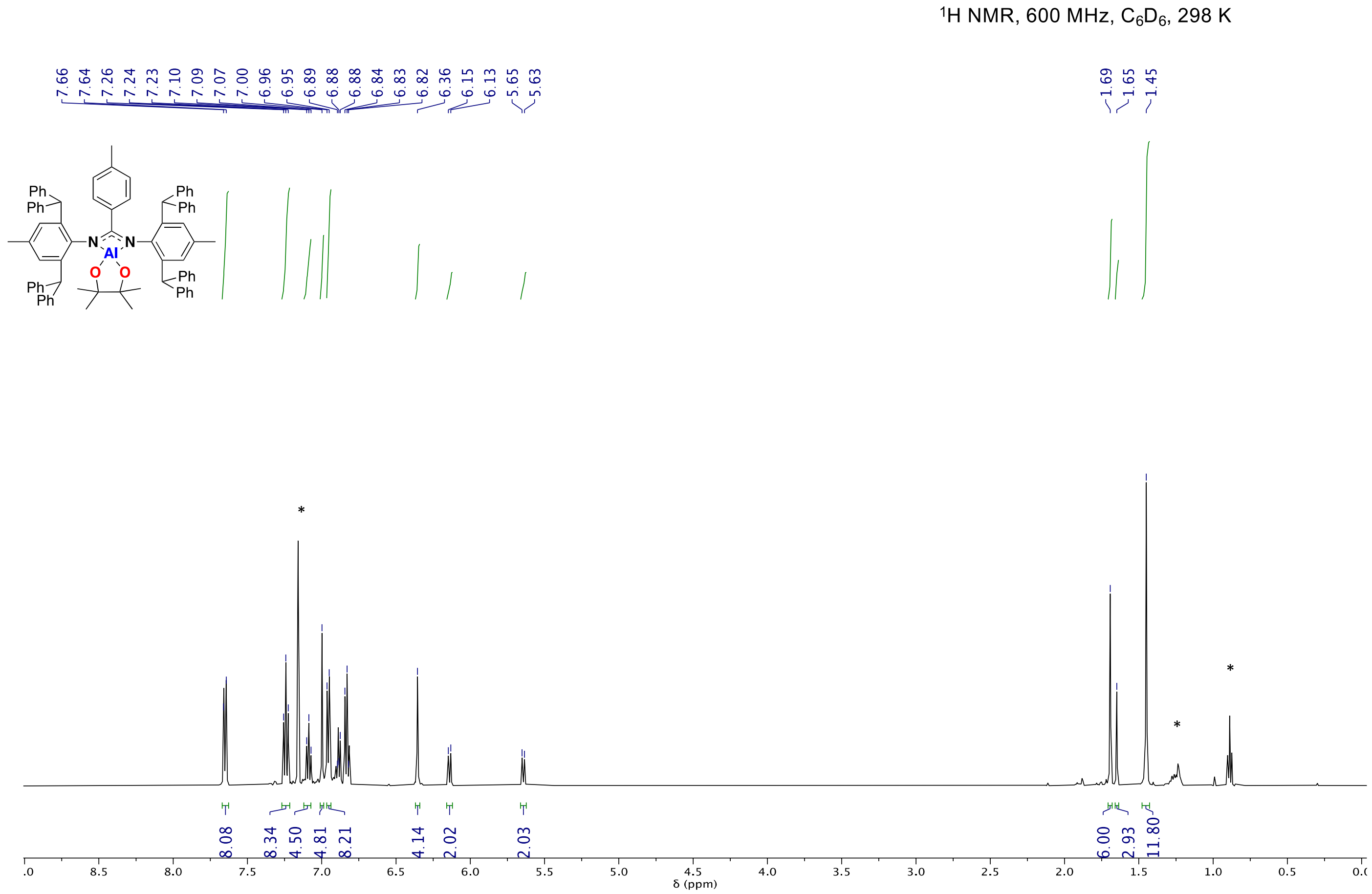

Figure S79: ^{13}C NMR spectrum of **7**

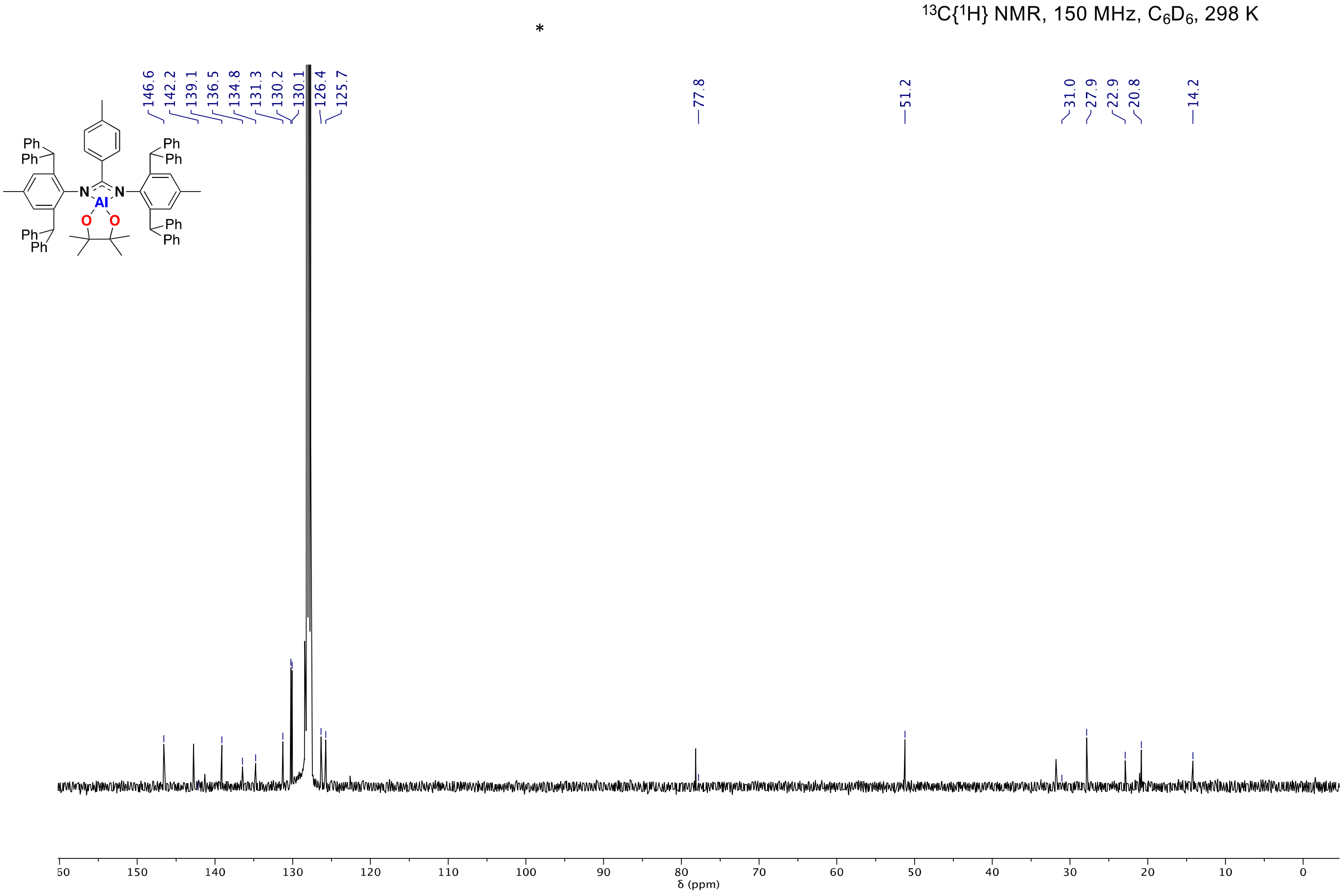

7. References

1 B. K. John Ruff and M. Frederick Hawthorne, *J. Am. Chem. Soc.*, 1960, **82**, 2141–2144.

2 A. Chartoire, C. Claver, M. Corpet, J. Krinsky, J. Mayen, D. Nelson, S. P. Nolan, I. Peñ, R. Woodward, R. E. Meadows and M. Domingo, *Org. Process Res. Dev.* , 2016, **20**, 551–557.

3 C. Cui, H. W. Roesky, H. Schmidt and M. Noltemeyer, *Angew. Chem. Int. Ed.*, 2000, **39**, 4274–4276.

4 O. V. Dolomanov, L. J. Bourhis, R. J. Gildea, J. A. K. Howard and H. Puschmann, *J. Appl. Crystallogr.*, 2009, **42**, 339–341.

5 S. G. M., *Acta Cryst. A*, **64**, 112–122.

6 G. M. Sheldrick, *Acta Cryst*, 2015, **A71**, 3–8.

8. XYZ Coordinates

2-mono

Al	-0.08018700	-0.72612200	-2.83913100
N	-0.39476000	-0.24845500	-0.95210700
N	1.47482100	-1.10339200	-1.70274400
C	-1.27803300	0.54892700	-0.17834500
C	1.39904300	-0.66665600	0.75640200
C	2.72837900	-0.29215900	0.98149000
C	0.83287600	-0.66887400	-0.61492900
C	3.24213100	-0.28936000	2.26808700
C	-2.57315600	0.05385500	0.08808900
C	2.73745700	-1.75269800	-1.73922700
C	-0.94310300	1.86036500	0.20529700
C	-3.14596000	2.13127800	1.22171100
C	-3.47729500	0.84915400	0.78444300
C	2.45668000	-0.67235200	3.35946500
C	1.13962900	-1.06400000	3.12360500
C	2.88118600	-3.07416900	-1.29591200
C	0.60865600	-1.05382000	1.83866900
C	1.35535800	2.75232200	0.96336800
C	1.03328700	2.56164200	2.30493600
C	-1.88490500	2.61916300	0.90513200
C	-2.97381400	-1.34972900	-0.36672100
C	3.82325400	-1.06290100	-2.30112800
C	0.37913500	2.51446500	-0.18891100
C	-4.45317500	-1.44456200	-0.73811900
C	-2.51643700	-2.40103800	0.63491800
C	3.02458700	-0.62568600	4.75330100
C	-2.77911900	-2.27697000	2.00320000
C	3.25842000	3.22501500	2.97805300
C	1.97769100	2.79860300	3.30449700
C	2.65095700	3.18146500	0.64601600
C	5.23328200	-3.01347400	-1.93458100
C	1.70594900	-3.83156500	-0.73725300
C	0.10902500	3.79129900	-0.98863700
C	4.13392600	-3.67917500	-1.39870700
C	-4.13542600	2.96311400	1.99718200
H	-5.15321000	2.81955300	1.62215300
H	-3.89541800	4.02810100	1.93218800
H	-4.13549100	2.68782400	3.05815500
C	-1.79921400	-3.51271300	0.19228100
C	-2.32733100	-3.23644700	2.90419600
C	5.05535600	-1.70622000	-2.38669600
C	-4.91474500	-0.67186300	-1.81077000
C	3.59424800	3.41345600	1.63749900
C	3.65675800	0.35467600	-2.78636100
C	-1.59896300	-4.33459500	2.45156100
C	-0.21426300	3.68313700	-2.34366800
C	-1.33882900	-4.47145000	1.09190600
C	0.14103600	5.05944300	-0.40693900
C	6.58398900	-3.67955500	-2.01044900
C	-6.24209800	-0.72321500	-2.21228500
C	-7.14193700	-1.55327700	-1.54468800
C	-0.50457600	4.81426800	-3.09819300
C	-0.15361400	6.19332700	-1.16028200
C	-0.47759600	6.07514800	-2.50756100
C	-5.35985300	-2.27380900	-0.08119800
C	-6.69575200	-2.32613700	-0.48114100
H	0.88421600	1.83393200	-0.88330800
H	-0.41881100	-1.36278900	1.67877300
H	-4.47977400	0.46647600	0.96838800
H	0.07413400	0.53278900	-3.80672900
H	3.35713400	0.01640200	0.15241200
H	0.51144100	-1.37952700	3.95401800
H	4.26839000	0.03095300	2.43269200
H	4.24794500	-4.70714300	-1.05722600
H	-1.62901000	3.64115100	1.17951100
H	-3.32421100	-1.40854300	2.36658900
H	-0.77033100	-5.32340700	0.72693500
H	-1.58994000	-3.61941200	-0.87089200
H	0.04385800	2.20769400	2.57847500
H	-2.42180500	-1.55747500	-1.28942800
H	2.91355900	3.34403500	-0.39850800
H	3.99193100	3.41139200	3.75848200
H	1.70314500	2.64329200	4.34551600
H	-2.53949800	-3.12293900	3.96444200
H	-0.23558900	2.69960100	-2.81173600
H	0.41306500	5.16370200	0.64100700
H	-0.86912800	-2.02823500	-3.31641700
H	5.89926800	-1.17148500	-2.82072700
H	-1.23687500	-5.07871200	3.15584500
H	-0.12330600	7.17339700	-0.69085100
H	-4.21813800	-0.01662800	-2.33121500
H	-6.57680100	-0.11637300	-3.04962000
H	2.33753800	-1.06347100	5.48240200
H	3.97585100	-1.16393200	4.81348800
H	3.21727300	0.41235800	5.04898000
H	-8.18198100	-1.59682500	-1.85681000
H	4.59285100	3.74649800	1.36600700
H	-0.70252000	6.96056900	-3.09631100
H	1.95102800	-4.89027800	-0.61385900
H	7.19853700	-3.42181100	-1.14022100
H	0.83876800	-3.75232000	-1.40237100
H	1.39819000	-3.44090400	0.23949100
H	3.34234100	1.02248000	-1.97371600
H	2.88806400	0.42953900	-3.56290800
H	6.48947100	-4.76899800	-2.03532500
H	7.13294500	-3.36581600	-2.90340000
H	4.59507200	0.73882500	-3.19510900
H	-0.74887500	4.70994700	-4.15220600
H	-5.02558500	-2.89408100	0.74551000
H	-7.38633700	-2.98078200	0.04446100

2’

Al	0.12930200	-1.45375000	-2.15808900
N	-0.41355600	-0.29797100	-0.50283200
N	1.56364400	-1.18010400	-0.81816900
N	1.26241000	-2.82853000	-3.51970800
C	-1.41775500	0.60337400	-0.08048300
C	1.23033200	0.02880700	1.35516400
C	2.49101200	0.60130200	1.55638500
C	0.78940800	-0.46218200	0.01845800
C	2.86542400	1.04976300	2.81313800
C	-2.68793800	0.09183400	0.26846600
C	2.88460000	-1.61133300	-0.53805200
C	-1.22987400	1.99741600	-0.13800600
C	-3.52376300	2.35261400	0.61761300
C	-3.70893800	0.97020800	0.61499300
C	2.00585000	0.93083100	3.90865300
C	0.75907800	0.34199700	3.70683300
C	3.11222300	-2.73417200	0.27529100
C	0.36968700	-0.09739700	2.44611600
C	0.92587800	3.30849600	0.39407900
C	0.51617400	3.53001000	1.70684800
C	-2.28589100	2.83988800	0.21982000
C	-2.93112100	-1.41684000	0.27499600
C	3.96434000	-0.94232500	-1.13987900
C	0.06117200	2.62093500	-0.66203500
C	-4.34926400	-1.77882000	-0.16015200
C	-2.50729800	-2.04234100	1.59627600
C	2.41817500	1.45881000	5.25761600
C	-2.91131000	-1.51870900	2.82869100
C	2.62952600	4.57818000	2.23643900
C	1.36102700	4.16215300	2.61971200
C	2.20729300	3.73383300	0.01880800
C	5.50743800	-2.53858900	-0.13482500
C	1.95830500	-3.44272300	0.93455100

C	-0.24769000	3.54187000	-1.84482300
C	4.42092500	-3.17782300	0.46035500
C	-4.64041600	3.28229400	1.01981200
H	-5.60649700	2.92340200	0.65181600
H	-4.47928700	4.28961900	0.62525600
H	-4.71525800	3.36224900	2.11059100
C	-1.66625500	-3.15632700	1.59039100
C	-2.47846200	-2.08995900	4.02186700
C	5.25728400	-1.42373500	-0.93229700
C	-4.72583200	-1.48553700	-1.47677900
C	3.05285600	4.35796300	0.92596600
C	3.73951300	0.30316600	-1.96219800
C	-1.62739800	-3.19274400	4.00255300
C	-0.48556600	2.96944800	-3.09760000
C	-1.22460300	-3.72602000	2.78221000
C	1.63878300	-4.06537800	-2.82195100
C	-0.33463000	4.92840600	-1.71407200
C	0.29338000	-3.14289400	-4.57951200
C	2.44847400	-2.20773400	-4.12253300
C	6.91501000	-3.02525800	0.10183100
C	-5.99502500	-1.79540100	-1.94489500
C	-6.92069400	-2.40898500	-1.10145000
C	-0.80617800	3.76483900	-4.19182400
C	-0.65984700	5.72599200	-2.80907200
C	-0.89631300	5.14779200	-4.05131500
C	-5.28031400	-2.39693100	0.67218200
C	-6.55793300	-2.70818400	0.20498600
H	0.66757600	1.80663400	-1.07378000
H	-0.60586400	-0.55213800	2.31623700
H	-4.68873400	0.56404800	0.86221700
H	0.02454400	-0.37963300	-3.35555400
H	3.17851900	0.71866300	0.72700800
H	0.07312400	0.22332600	4.54321600
H	3.83872300	1.51773900	2.94364000
H	4.59503400	-4.05197700	1.08678200
H	-2.13881300	3.91661200	0.14954700
H	-3.55086000	-0.63903100	2.85152700
H	-0.55849100	-4.58523500	2.75500100
H	-1.34345000	-3.56687000	0.63509200
H	-0.46334300	3.19129700	2.03066700
H	-2.26854000	-1.84281600	-0.48405100
H	2.53499300	3.57986400	-1.00866100
H	3.28566200	5.07144300	2.94920700
H	1.01902900	4.32383000	3.63960400
H	-2.80136700	-1.66775400	4.97040200
H	-0.41855700	1.88756900	-3.21086300
H	-0.13408100	5.39127000	-0.75045200
H	-0.89658000	-2.68103000	-1.94213600
H	6.09265900	-0.90732200	-1.40388400
H	-1.28020000	-3.63222700	4.93388600
H	-0.72324300	6.80465800	-2.68863600
H	-4.00658000	-1.00274900	-2.13636400
H	-6.26446400	-1.55988000	-2.97140100
H	1.69440400	1.19069500	6.03206700
H	3.39708300	1.06953700	5.55553200
H	2.49630700	2.55210300	5.23093200
H	-7.91545000	-2.65336300	-1.46484500
H	2.87225900	-2.85255300	-4.90677100
H	2.38538400	-3.84282100	-2.05483200
H	0.75096700	-4.49027000	-2.34551800
H	0.72882500	-3.84077700	-5.30915100
H	2.05778500	-4.80073000	-3.52475800
H	-0.59815100	-3.59131600	-4.13414400
H	3.20793800	-2.04422400	-3.35487100
H	4.04248700	4.67899400	0.61024800
H	0.00359000	-2.22021000	-5.08939100
H	-1.14593700	5.77041800	-4.90659400
H	2.16694900	-1.24709800	-4.56320000
H	2.28799500	-4.37565800	1.40082600
H	7.32400600	-2.60694000	1.02861300
H	1.16790700	-3.67260600	0.21079900
H	1.49979700	-2.81876500	1.71049200
H	3.54825100	1.17307800	-1.31931700
H	2.86958600	0.21087700	-2.61831700
H	6.94972700	-4.11516000	0.19216500
H	7.58117400	-2.73053600	-0.71422100
H	4.61866300	0.53306700	-2.57100400
H	-0.98471200	3.30221100	-5.15931500
H	-5.00924200	-2.64901300	1.69359900
H	-7.26883600	-3.19080200	0.87111600

2-dimer

Al	1.44232900	-0.16890400	1.17066900
N	3.24308300	0.34741700	0.45983300
C	3.21508400	-0.67871400	-0.38826300
C	4.34359600	1.14717500	0.85969100
C	2.87210100	5.63363600	-0.60887700
H	3.36126600	5.57155100	-1.57734300
C	5.41197100	-0.82246600	2.11465600
H	4.42998100	-1.25139900	1.88380500
C	4.33038100	-1.12396100	-1.25741800
C	4.29817000	2.52931500	0.57316900
C	2.71374100	4.48376700	0.16320400
C	5.55418000	-0.89778200	3.63734700
C	4.62553300	-2.48629600	-1.37259300
H	4.03820800	-3.21820300	-0.82536000
C	5.38132100	0.63499900	1.65934400
C	6.13748500	-0.62847700	-2.77737200
H	6.71610700	0.10661500	-3.33290600
C	5.09573600	-0.19678300	-1.96450300
H	4.86846700	0.86177900	-1.89770000
C	3.17209200	3.09822800	-0.28577300
H	2.30428100	2.44556900	-0.14418900
C	5.30487600	3.35983600	1.05411600
H	5.24441600	4.42853600	0.85257500
C	2.06200400	4.59414400	1.39682800
H	1.92522300	3.70136100	2.00550100
C	6.44392500	-1.70028000	1.40674600
C	6.37818500	1.50681400	2.10554600
H	7.16745600	1.11170800	2.74265600
C	3.51252900	3.02238700	-1.76817400
C	2.38983400	6.86419800	-0.16116100
H	2.52249200	7.74884200	-0.77941900
C	1.57954300	5.81671700	1.84512500
H	1.07193500	5.87731000	2.80468900
C	6.36792500	2.86414400	1.80841900
C	6.45297000	-1.98274100	-2.88244800
C	7.45850700	-1.20001000	0.59449900
H	7.52406100	-0.13190100	0.40880000
C	6.78060300	-1.12990700	4.26204200
H	7.66645300	-1.31116800	3.65757300
C	4.42426700	-0.69481500	4.43382900
H	3.45981600	-0.52215500	3.95844000
C	1.74083200	6.96100500	1.06390700
H	1.36362800	7.91923300	1.41137500
C	4.76293500	3.39309400	-2.27269600
H	5.53631000	3.73926200	-1.59090600
C	5.68022600	-2.90357100	-2.16891300
H	5.91796400	-3.96315700	-2.22964900
C	6.37373000	-3.08534300	1.60557000
H	5.58931600	-3.48858900	2.24435700
C	6.87756400	-1.14834000	5.65147100
H	7.84117800	-1.33082900	6.12059600
C	5.03674000	3.29592400	-3.63451200
H	6.01535400	3.58588400	-4.00909400
C	7.29153600	-3.94315000	1.01574800
H	7.21760200	-5.01422800	1.18665800
C	7.61973200	-2.45382300	-3.70941300
H	7.33834600	-3.29084400	-4.35606000
H	8.42800700	-2.80251200	-3.05550600

H	8.01622200	-1.65268800	-4.33904800
C	8.30834400	-3.43036400	0.21034400
H	9.03369800	-4.09774000	-0.24855900
C	2.54397900	2.56322700	-2.66031400
H	1.56245000	2.28740500	-2.28074800
C	7.46282000	3.77869500	2.29590200
H	8.21474300	3.94462500	1.51574100
H	7.97571100	3.35553500	3.16416500
H	7.06407800	4.75741000	2.57928400
C	5.74774700	-0.94235900	6.43568300
H	5.82254100	-0.96198800	7.51980900
C	8.38481000	-2.05943600	0.00199100
H	9.16941900	-1.64633700	-0.62809800
C	4.51855400	-0.71778000	5.82084600
H	3.62687400	-0.56304100	6.42294400
C	4.06486100	2.82434300	-4.51426100
H	4.28243400	2.74034600	-5.57571900
C	2.81461300	2.46118600	-4.02240900
H	2.04427400	2.09068900	-4.69548600
H	0.44998100	1.12115300	0.95980100
H	1.58582700	-0.68162500	2.66760200
N	2.02364000	-1.29260100	-0.30244600
C	1.52557000	-2.34583800	-1.11200300
C	1.17629500	-3.55198200	-0.48260300
C	0.59697200	-4.56946900	-1.24061200
H	0.29944200	-5.49117400	-0.74171600
C	0.37816300	-4.42951100	-2.60844700
C	0.75555500	-3.23139700	-3.21379000
H	0.58260200	-3.09823800	-4.28190600
C	1.30485000	-2.17354900	-2.49014100
C	1.61461200	-0.87021400	-3.17898500
H	1.29467800	-0.01864600	-2.56898900
H	1.09731700	-0.81300400	-4.14190400
H	2.68676400	-0.74527300	-3.36629600
C	1.42767200	-3.75334900	0.98960500
H	2.46098800	-3.50162600	1.25788900
H	1.23595300	-4.79144400	1.27331300
C	-0.30377300	-5.51196600	-3.40506100
H	-0.41006300	-6.42836300	-2.81743000
H	0.25199900	-5.75019100	-4.31808000
H	-1.30864300	-5.18859800	-3.70423200
Al	-1.44232800	0.16890100	1.17066900
N	-3.24308200	-0.34741800	0.45983300
C	-3.21508300	0.67871300	-0.38826300
C	-4.34359500	-1.14717700	0.85969000
C	-2.87210500	-5.63363600	-0.60888600
H	-3.36127400	-5.57155100	-1.57734900
C	-5.41196600	0.82246400	2.11465900
H	-4.42997500	1.25139500	1.88380800
C	-4.33038100	1.12396000	-1.25741700
C	-4.29817000	-2.52931600	0.57316600
C	-2.71374100	-4.48376700	0.16319400
C	-5.55417400	0.89777800	3.63735000
C	-4.62553600	2.48629600	-1.37258800
H	-4.03821200	3.21820200	-0.82535200
C	-5.38131800	-0.63500100	1.65934600
C	-6.13748300	0.62847800	-2.77737400
H	-6.71610300	-0.10661300	-3.33291000
C	-5.09573400	0.19678400	-1.96450500
H	-4.86846300	-0.86177900	-1.89770600
C	-3.17209500	-3.09822800	-0.28578100
H	-2.30428400	-2.44556800	-0.14420000
C	-5.30487600	-3.35983700	1.05411400
H	-5.24441700	-4.42853700	0.85257100
C	-2.06199800	-4.59414300	1.39681500
H	-1.92521400	-3.70136000	2.00548700
C	-6.44391900	1.70027900	1.40675000
C	-6.37818100	-1.50681700	2.10554900
H	-7.16745100	-1.11171100	2.74266200
C	-3.51253900	-3.02238600	-1.76818100
C	-2.38983500	-6.86419800	-0.16117200
H	-2.52249500	-7.74884200	-0.77942900
C	-1.57953300	-5.81671500	1.84511000
H	-1.07192100	-5.87730700	2.80467100
C	-6.36792300	-2.86414600	1.80842000
C	-6.45297000	1.98274200	-2.88244600
C	-7.45850500	1.20001200	0.59450600
H	-7.52406400	0.13190300	0.40881000
C	-6.78059700	1.12990600	4.26204600
H	-7.66644700	1.31117000	3.65757800
C	-4.42426100	0.69480800	4.43383100
H	-3.45981100	0.52214500	3.95844100
C	-1.74082600	-6.96100300	1.06389300
H	-1.36362000	-7.91923100	1.41135900
C	-4.76294600	-3.39309400	-2.27269800
H	-5.53631800	-3.73926300	-1.59090400
C	-5.68022800	2.90357100	-2.16890800
H	-5.91796800	3.96315700	-2.22964100
C	-6.37371700	3.08534300	1.60557100
H	-5.58930100	3.48858800	2.24435400
C	-6.87755600	1.14833800	5.65147500
H	-7.84117000	1.33083000	6.12060100
C	-5.03675700	-3.29592400	-3.63451200
H	-6.01537300	-3.58588300	-4.00908900
C	-7.29152200	3.94315200	1.01574800
H	-7.21758200	5.01423000	1.18665600
C	-7.61973300	2.45382400	-3.70941000
H	-7.33835200	3.29085700	-4.35604500
H	-8.42801400	2.80249700	-3.05550200
H	-8.01621300	1.65269400	-4.33905800
C	-8.30833400	3.43036900	0.21034800
H	-9.03368600	4.09774700	-0.24855400
C	-2.54399300	-2.56322600	-2.66032500
H	-1.56246300	-2.28740400	-2.28076300
C	-7.46281700	-3.77869700	2.29590400
H	-8.21475100	-3.94461300	1.51575000
H	-7.97569500	-3.35554600	3.16417900
H	-7.06407800	-4.75741800	2.57927000
C	-5.74774000	0.94235400	6.43568700
H	-5.82253300	0.96198200	7.51981300
C	-8.38480600	2.05944000	0.00199900
H	-9.16941800	1.64634300	-0.62808700
C	-4.51854800	0.71777200	5.82084800
H	-3.62686700	0.56303100	6.42294500
C	-4.06488300	-2.82434200	-4.51426500
H	-4.28246100	-2.74034400	-5.57572200
C	-2.81463300	-2.46118400	-4.02241900
H	-2.04429700	-2.09068700	-4.69549800
H	-0.44998100	-1.12115700	0.95980200
H	-1.58582700	0.68162300	2.66760100
N	-2.02364000	1.29260000	-0.30244600
C	-1.52557100	2.34583900	-1.11200100
C	-1.17629300	3.55198100	-0.48260000
C	-0.59697100	4.56947000	-1.24060700
H	-0.29943900	5.49117300	-0.74171000
C	-0.37816600	4.42951500	-2.60844400
C	-0.75556200	3.23140300	-3.21378800
H	-0.58261400	3.09824600	-4.28190500
C	-1.30485600	2.17355300	-2.49014100
C	-1.61462300	0.87022100	-3.17898800
H	-1.29469200	0.01865000	-2.56899400
H	-1.09733000	0.81301200	-4.14190700
H	-2.68677700	0.74528500	-3.36629700
C	-1.42766600	3.75334500	0.98961000
H	-2.46098000	3.50162000	1.25789600
H	-1.23594500	4.79143900	1.27331900
H	-0.77738700	3.11541600	1.59915100
C	0.30376800	5.51197100	-3.40505600
H	0.41004900	6.42837000	-2.81742800
H	-0.25199900	5.75019000	-4.31808000
H	1.30864300	5.18860700	-3.70422000
H	0.77739600	-3.11542100	1.59915000

0.0

C	4.24271700	0.19370300	-1.40697800
H	4.61757000	0.56023800	-2.36014800
C	-5.09559300	0.10359200	0.31446400
N	-1.09291900	-1.10465400	-0.06385500
C	-2.76704900	-1.32029200	-2.33908200
H	-1.90366700	-0.74649000	-2.69624400
N	1.09292300	-1.10465000	0.06390200
C	-1.99784500	-0.12352300	2.51179000
H	-1.40247200	-1.03802700	2.60920500
C	-4.61881300	-0.38786000	-0.89573400
H	-5.28582000	-0.47048600	-1.75111000
C	2.90520100	-0.19269800	-1.31054600
C	2.76709900	-1.32024300	2.33909900
C	3.28914400	-0.78183600	1.03173600
C	-4.24274400	0.19372100	1.40693900
H	-4.61761800	0.56027200	2.36009400
C	-2.90522500	-0.19267900	1.31053900
C	1.99780200	-0.12357200	-2.51178400
H	1.29842000	0.71777600	-2.44055200
C	4.61883300	-0.38783400	0.89569700
H	5.28585700	-0.47044500	1.75106100
C	2.43362000	-0.66336900	-0.07557400
C	-3.28912100	-0.78186100	-1.03174000
C	5.09558900	0.10359700	-0.31451900
C	-2.43362000	-0.66337200	0.07558500
C	0.00000100	-0.34136700	0.00001400
C	-0.00000100	1.14656100	0.00000100
C	0.85369900	1.85004800	0.85462300
C	-0.85370100	1.85003800	-0.85462800
C	0.84651000	3.24009200	0.85823000
H	1.52181900	1.30886600	1.51800800
C	-0.84651400	3.24008200	-0.85824900
H	-1.52182000	1.30884900	-1.51800800
H	1.50805300	3.77986900	1.52967700
H	-1.50805800	3.77985100	-1.52970200
C	-0.00000300	3.93605500	-0.00001300
Al	0.00000500	-2.74773400	0.00003800
H	0.09149200	-3.52693400	-1.38708700
H	-0.09148700	-3.52691500	1.38717300
H	-6.13514700	0.40589800	0.40839000
H	-3.54179600	-1.29066300	-3.10978800
H	-2.42466100	-2.35607100	-2.23841000
H	-2.58055600	0.00647100	3.42752300
H	-1.29846700	0.71782700	2.44054900
H	-0.00000400	5.02270700	-0.00001900
H	6.13513900	0.40590500	-0.40846900
H	3.54186700	-1.29061500	3.10978500
H	1.90373400	-0.74642400	2.69627700
H	2.42469400	-2.35601800	2.23844900
H	2.58049800	0.00640500	-3.42752900
H	1.40243200	-1.03808200	-2.60917000

Int-1

C	-2.80900800	2.22552700	0.99612300
H	-3.23887900	2.36678500	1.98566500
C	5.71041400	-1.76683200	0.11499900
N	1.62237500	-0.97429200	-0.41630100
C	2.36989400	-2.78213100	1.63265200
H	1.75201700	-2.04652200	2.16165000
N	-0.26829500	0.10079700	-0.67930800
C	3.51652100	0.33068500	-2.17981800
H	2.66810800	-0.08774600	-2.73253800
C	4.76186700	-2.38051600	0.92566900
H	5.07548200	-3.07933300	1.69824000
C	-1.83025100	1.24617100	0.82004600
C	-1.14533200	1.61000800	-2.92084400
C	-1.73386800	1.82711500	-1.55187400
C	5.30245500	-0.88721500	-0.87941500
H	6.04049000	-0.42013800	-1.52805200
C	3.95140800	-0.59731200	-1.07485000
C	-1.41844600	0.35930700	1.96577900
H	-0.41100300	0.59758500	2.32670600
C	-2.72668000	2.78284700	-1.34104900
H	-3.07928100	3.37430500	-2.18336000
C	-1.27572500	1.07476200	-0.45733600
C	3.40368800	-2.11537400	0.76186000
C	-3.25500500	2.99399500	-0.07126900
C	3.00816600	-1.20501900	-0.23184700
C	0.97905700	0.16795300	-0.19849300
C	1.56665700	1.35514500	0.48314900
C	1.36240500	2.64038000	-0.02700500
C	2.33585900	1.18746500	1.63801200
C	1.92661800	3.74109400	0.60741700
H	0.76323100	2.77871000	-0.92202300
C	2.88528600	2.29240100	2.27828900
H	2.50357500	0.19057900	2.03510500
H	1.76839400	4.73583700	0.20071100
H	3.47658600	2.15355500	3.17886400
C	2.68457900	3.56923900	1.76219700
Al	0.02461700	-1.74403700	-1.32946600
H	-0.60000300	-2.92162200	-0.45929400
H	0.29564000	-1.87347900	-2.89563800
H	6.76656200	-1.98364700	0.25127100
H	2.84488600	-3.42846000	2.37566100
H	1.67847000	-3.38911400	1.03850300
H	4.33547100	0.50252700	-2.88340900
H	3.20478500	1.30586300	-1.78733200
H	3.11980500	4.43153400	2.26010700
H	-4.02046600	3.75032000	0.08202600
H	-1.55277300	2.32602700	-3.63967100
H	-0.05361900	1.71004800	-2.91233800
H	-1.36115700	0.59859700	-3.28103200
H	-2.11131200	0.46996800	2.80430700
H	-1.42194200	-0.69288700	1.65821800
C	-2.71535900	-1.72096800	-2.82042300
C	-3.18381300	-1.68381400	-1.70735300
H	-2.32020400	-1.77635500	-3.81102000
C	-3.75767500	-1.65235300	-0.38775200
C	-4.50821100	-0.54568700	0.02228600
C	-3.56742800	-2.72812500	0.48917100
C	-5.06547400	-0.51917200	1.29517200
H	-4.62798400	0.29745000	-0.65271100
C	-4.12700600	-2.69258200	1.76019300
H	-2.97082300	-3.57627100	0.16708800
C	-4.87746800	-1.59032100	2.16441000
H	-5.64139500	0.34682500	1.61025700
H	-3.97586400	-3.52782900	2.43835700
H	-5.31411100	-1.56620700	3.15943600

TS-1

C	1.87330800	2.91970300	-1.50025800
H	1.97173700	3.44630200	-2.44687600
C	-5.79460200	-2.40992500	0.36640900
N	-1.82360100	-1.14366800	-0.13061800
C	-3.20772200	-2.12103700	-2.41151200
H	-2.82752300	-1.14193900	-2.72811500
N	-0.00405900	0.05813500	-0.06847900
C	-2.99022100	-0.99346200	2.51214400
H	-1.98830100	-1.43576700	2.54151300
C	-5.18671500	-2.47397700	-0.88252200
H	-5.73686000	-2.86267000	-1.73675100
C	0.97955800	1.85241500	-1.40786100
C	1.52079600	0.80119500	2.23049600
C	1.63616700	1.54908600	0.92771000
C	-5.08268200	-1.92466500	1.45565000
H	-5.54905600	-1.88931200	2.43785300

C	-3.76437300	-1.48857000	1.31761700
C	0.16825500	1.42401100	-2.60306900
H	-0.89827800	1.63557100	-2.46206500
C	2.51680900	2.62345500	0.79819900
H	3.11267000	2.92464600	1.65745100
C	0.86097400	1.17836000	-0.18300100
C	-3.87190400	-2.04802500	-1.06040600
C	2.63766500	3.30711100	-0.40698500
C	-3.17265600	-1.53881200	0.04600400
C	-1.34755400	0.08720000	-0.03830100
C	-2.16500800	1.32568700	0.06733000
C	-1.79905700	2.34544600	0.95087700
C	-3.30960700	1.47527800	-0.72158300
C	-2.57448100	3.49493000	1.04884500
H	-0.90982100	2.23864600	1.56425800
C	-4.07202500	2.63414200	-0.63250700
H	-3.60274600	0.68416500	-1.40530300
H	-2.28765200	4.27938300	1.74326100
H	-4.95589000	2.74545900	-1.25396500
C	-3.70817400	3.64267700	0.25498000
Al	0.07372700	-1.86300100	-0.33956700
H	0.07479900	-2.82733500	-1.59089100
H	0.15769800	-2.75553200	1.20058400
H	-6.81961900	-2.74829100	0.49191400
H	-3.90900500	-2.47634800	-3.17121300
H	-2.34606500	-2.79640200	-2.39480400
H	-3.50728600	-1.25070700	3.44031900
H	-2.86794700	0.09604100	2.49078600
H	-4.30908500	4.54510100	0.32813400
H	3.32892800	4.14126200	-0.49365900
H	2.05582400	1.32387300	3.02808600
H	0.47637100	0.67714000	2.53850600
H	1.94630100	-0.20325200	2.13125800
H	0.49629200	1.95047100	-3.50298200
H	0.26546500	0.34733800	-2.78413800
C	2.19558100	-1.98255600	0.17241000
C	3.39677900	-1.76195200	0.14296400
H	1.14098700	-2.51725200	0.93890400
C	4.79665100	-1.44831300	0.10303500
C	5.21099000	-0.11002800	0.15012700
C	5.75568700	-2.46522300	0.01538900
C	6.56454800	0.19961000	0.10988600
H	4.46121000	0.67557800	0.21130900
C	7.10765700	-2.14718200	-0.01741300
H	5.42846800	-3.50008300	-0.02560700
C	7.51432800	-0.81599800	0.02875400
H	6.88024900	1.23888900	0.14282400
H	7.84697400	-2.94076500	-0.08285900
H	8.57250200	-0.56994900	-0.00012900

Int-2

C	3.10857500	-4.01697300	-0.88801600
H	4.05654300	-4.28639900	-1.34860800
C	0.71675000	5.12004900	0.21191000
N	0.61758200	1.05675700	-0.83258900
C	1.80805300	2.86664400	-2.65569000
H	2.57591100	2.09734200	-2.51149900
N	0.79677000	-1.12067700	-0.70718800
C	-0.58234400	1.85636300	1.67862400
H	-1.28093900	1.22042800	1.12194200
C	1.25736300	4.68991400	-0.99459200
H	1.69893800	5.40867600	-1.68141000
C	2.59736000	-2.73484300	-1.09304000
C	-0.66753200	-2.96037900	0.88571700
C	0.65527900	-3.33536000	0.26847900
C	0.13731600	4.20003900	1.07664500
H	-0.30572000	4.53762800	2.01104800
C	0.09541900	2.84148100	0.76110100
C	3.33628400	-1.74230100	-1.95349900
H	3.80942500	-0.95885900	-1.34967000
C	1.19814100	-4.60646100	0.44827000
H	0.65124300	-5.33560500	1.04203100
C	1.37393000	-2.39982200	-0.49330300
C	1.23951800	3.34031800	-1.34261600
C	2.42002000	-4.94767100	-0.12079000
C	0.67281700	2.42059700	-0.44625300
C	1.26556300	0.04342000	-0.25206200
C	2.35025900	0.19091200	0.75467100
C	2.39204300	-0.65191500	1.86908300
C	3.33269000	1.17235300	0.59400700
C	3.40131300	-0.50850600	2.81398900
H	1.63290400	-1.41762500	1.99725500
C	4.34891200	1.30140400	1.53352100
H	3.30227200	1.83312800	-0.26717200
H	3.42357300	-1.16255600	3.68086200
H	5.11283500	2.06170400	1.39879200
C	4.38223300	0.46445000	2.64531700
Al	-0.49488700	-0.19034400	-1.85591200
H	-0.22370600	-0.28230200	-3.41507400
H	-1.47620100	2.81996200	-2.37277700
H	0.73557400	6.17497800	0.47203700
H	2.25385700	3.69672700	-3.21000800
H	1.03359500	2.41271000	-3.28486900
H	-1.13829000	2.38078200	2.46065400
H	0.14212600	1.19766500	2.17210300
H	5.17401400	0.57111800	3.38187300
H	2.83038800	-5.94318900	0.02584400
H	-1.07181800	-3.79072200	1.47073100
H	-0.57177400	-2.09022300	1.54658600
H	-1.40661300	-2.68016700	0.12769500
H	4.12326000	-2.23974300	-2.52633600
H	2.65827400	-1.24844500	-2.65830400
C	-2.30047600	-0.28582500	-1.15666800
C	-3.42545600	-0.32265400	-0.68756200
H	-2.03952100	2.34022500	-2.26729700
C	-4.74917700	-0.36126300	-0.12857400
C	-5.00987700	0.23698500	1.11104200
C	-5.79230400	-0.99608600	-0.81465700
C	-6.28921900	0.19959600	1.65132700
H	-4.19943200	0.73034700	1.64069400
C	-7.06941400	-1.03115800	-0.26862800
H	-5.58842500	-1.45819600	-1.77620100
C	-7.32129900	-0.43431500	0.96406000
H	-6.48202800	0.66732500	2.61308100
H	-7.87237900	-1.52612000	-0.80817100
H	-8.32124400	-0.46264400	1.38857200

Int-3

C	-0.38812700	4.16388300	1.19734700
H	-0.09540800	4.83781400	1.99962000
C	4.44035200	-3.89312800	-0.16267100
N	1.31042100	-1.15804800	0.42418900
C	2.62866500	-2.17864600	2.71330200
H	2.68670500	-1.09117600	2.84230000
N	0.03713700	0.61056900	0.21913600
C	1.98375800	-1.91713000	-2.29302700
H	0.90202700	-1.93073300	-2.12003800
C	4.07015200	-3.50960500	1.12162000
H	4.58581500	-3.92552700	1.98449900
C	0.05441200	2.83995900	1.23053300
C	-1.62073100	1.46689300	-1.91901200
C	-1.16783300	2.42294100	-0.84743400
C	3.76855000	-3.36948500	-1.25969000
H	4.04320600	-3.68181700	-2.26494300
C	2.72932300	-2.45223600	-1.09761000
C	0.90568900	2.34357300	2.37149400
H	1.94800700	2.20125500	2.06282500
C	-1.58690300	3.75209900	-0.84638200
H	-2.23252500	4.10341300	-1.64817000
C	-0.33123300	1.97892300	0.19014200
C	3.03851900	-2.59498300	1.32394900
C	-1.19858600	4.62219800	0.16630100

C	2.38434700	-2.05656700	0.20384600
C	1.27673400	0.12773900	0.08391800
C	2.44788400	0.91934300	-0.38179900
C	2.31273900	1.81868300	-1.44338600
C	3.69118400	0.76681700	0.23868200
C	3.41041900	2.55023900	-1.88193900
H	1.34854400	1.94286800	-1.92743400
C	4.78253400	1.51147800	-0.19350400
H	3.80300400	0.06567200	1.06049300
H	3.29921200	3.24234000	-2.71171900
H	5.74398700	1.39204800	0.29764200
C	4.64446800	2.40054000	-1.25561400
Al	-0.63487800	-1.13626800	0.84581400
H	-0.90827300	-1.22065200	2.41080000
H	-1.45628800	-1.97336300	-0.23113200
H	5.24510800	-4.60896900	-0.30720900
H	3.26854200	-2.64793800	3.46531900
H	1.59033500	-2.45593200	2.92503900
H	2.19813900	-2.51760600	-3.18106400
H	2.26804400	-0.88179100	-2.51524800
H	5.50009700	2.97755300	-1.59593000
H	-1.53662000	5.65492700	0.15725100
H	-2.13268900	1.99879800	-2.72536400
H	-0.78182400	0.90688100	-2.34692100
H	-2.31600000	0.72812100	-1.50311100
H	0.89945600	3.05967400	3.19749300
H	0.54214200	1.38028500	2.74688900
C	-3.24845300	0.89870000	1.31867100
C	-4.03350500	0.12709100	0.82138600
C	-5.01542600	-0.75585400	0.24632900
C	-4.63338600	-1.79793200	-0.60634800
C	-6.37160700	-0.56262400	0.53743900
C	-5.59905600	-2.63216300	-1.15730600
H	-3.57916200	-1.95000700	-0.82180600
C	-7.33008700	-1.40075500	-0.01790100
H	-6.66203900	0.24743100	1.20020400
C	-6.94630100	-2.43644600	-0.86615200
H	-5.29609800	-3.44090300	-1.81667700
H	-8.38047100	-1.24516300	0.21265800
H	-7.69754500	-3.09166500	-1.29894400
H	-2.56789300	1.60672200	1.74269200

TS-2

C	-0.71873300	-4.54694400	1.08101400
H	-1.32035600	-5.16825700	1.74096500
C	-3.01455000	4.57372500	-0.18437200
N	-0.86891800	1.06322200	0.64767700
C	-2.41658500	2.20362100	2.73499600
H	-2.76990000	1.16585500	2.69365400
N	-0.04071700	-0.95014400	0.46619200
C	-0.66856200	2.19535900	-2.00582700
H	0.31599300	1.92265400	-1.60939900
C	-3.06820600	3.99779600	1.08028900
H	-3.66073600	4.46257000	1.86555300
C	-0.79943900	-3.15918100	1.19770700
C	1.68643500	-2.09131900	-1.50932000
C	0.84220800	-2.94844600	-0.60080000
C	-2.24465400	3.98255900	-1.17772000
H	-2.18489000	4.43942000	-2.16340700
C	-1.52966900	2.80965000	-0.93213300
C	-1.69637000	-2.52809000	2.23164100
H	-2.55486300	-2.02844700	1.76736800
C	0.89620800	-4.33946600	-0.68712700
H	1.55569100	-4.79826900	-1.42085700
C	-0.02068600	-2.36341700	0.34124300
C	-2.36743100	2.82730700	1.36384800
C	0.12080300	-5.13793900	0.14514600
C	-1.61292000	2.22651100	0.34228600
C	-1.06895300	-0.14256700	0.15101600
C	-2.26007100	-0.56723700	-0.63620300
C	-2.11463700	-1.38798400	-1.75837300
C	-3.53711200	-0.14431700	-0.25563700
C	-3.23051000	-1.77185100	-2.49328400
H	-1.12696800	-1.72461300	-2.05801600
C	-4.65195800	-0.54335100	-0.98387700
H	-3.65725500	0.49738800	0.61213700
H	-3.10721100	-2.40435100	-3.36782100
H	-5.64081700	-0.21482800	-0.67690000
C	-4.50005600	-1.35372800	-2.10519000
Al	0.87532000	0.39193000	1.55796300
H	0.66228500	0.70004500	3.10166000
H	1.99326100	1.37590200	0.81343500
H	-3.56360200	5.48849700	-0.39125900
H	-3.08288200	2.76566700	3.39509700
H	-1.42204500	2.16942200	3.19128200
H	-0.52649400	2.89474400	-2.83419000
H	-1.12015100	1.28344600	-2.41434900
H	-5.37191600	-1.65989600	-2.67709400
H	0.17377400	-6.22062200	0.06681500
H	2.14032400	-2.69459100	-2.30046000
H	1.09828800	-1.29343600	-1.97607800
H	2.48965400	-1.59662500	-0.95295600
H	-2.08407400	-3.28350800	2.92018300
H	-1.16011400	-1.77304500	2.81787900
C	2.71102900	-0.80235100	1.77577000
C	3.18441000	0.12723300	1.08303000
C	4.22215200	0.77757600	0.28622600
C	4.15927700	2.09182400	-0.17343800
C	5.35402300	0.00041500	0.00471000
C	5.21664700	2.62578700	-0.90139600
H	3.27599300	2.68655000	0.04184800
C	6.40695100	0.53976700	-0.72225800
H	5.39679600	-1.02492900	0.36187200
C	6.34126400	1.85431200	-1.17762800
H	5.15925100	3.65151800	-1.25478800
H	7.27992500	-0.07037800	-0.93697800
H	7.16426100	2.27489400	-1.74892600
H	2.91525000	-1.74094200	2.25710000

Int-4

C	4.58801800	2.65042700	0.77985400
H	5.54587400	2.51945800	1.27860500
C	-1.62497300	-4.55270600	-0.02359500
N	0.12898900	-0.83571000	0.84789100
C	0.08820100	-2.79291500	2.88247100
H	1.13040200	-2.46039500	2.81555600
N	1.25223900	1.03091800	0.62126900
C	-0.96977600	-1.27915000	-1.84101000
H	-1.12889100	-0.27546100	-1.43306100
C	-1.08708300	-4.29823200	1.23314600
H	-1.12897700	-5.06016400	2.00839300
C	3.56696900	1.73147300	1.02604400
C	0.77761100	3.19105100	-1.14180200
C	2.11680700	3.00667300	-0.47541200
C	-1.57602400	-3.57097400	-1.00373800
H	-2.00884600	-3.76073600	-1.98374200
C	-0.98764400	-2.32836400	-0.75754500
C	3.77803000	0.58502500	1.98114000
H	3.88011300	-0.36829800	1.44932900
C	3.16023900	3.90411700	-0.69438800
H	2.99970500	4.75166100	-1.35715800
C	2.33691500	1.91148900	0.37552400
C	-0.49176600	-3.07216400	1.51979800
C	4.39296700	3.72691400	-0.07596700
C	-0.43285800	-2.09302500	0.51246400
C	1.17240300	-0.24753800	0.25364500
C	2.12560000	-0.93613400	-0.65720500
C	2.61225800	-0.29120100	-1.79783300
C	2.54477200	-2.23799300	-0.36789700
C	3.50077500	-0.94636700	-2.64254800
H	2.29207200	0.72095600	-2.02614600
C	3.44460800	-2.88373100	-1.20781100

H	2.16600300	-2.74294300	0.51623200
H	3.86972700	-0.44206700	-3.53103000
H	3.77061800	-3.89308500	-0.97381300
C	3.92002000	-2.24058300	-2.34716400
Al	-0.30874200	0.82433500	1.82073000
H	0.14002500	0.85778600	3.35009300
H	-2.66443300	-0.25638200	0.37423400
H	-2.08999000	-5.51187400	-0.23460300
H	0.04965000	-3.68588500	3.51189600
H	-0.45640800	-1.99312200	3.39711900
H	-1.75636200	-1.47565000	-2.57504500
H	-0.01278100	-1.27176600	-2.37538000
H	4.61881300	-2.74880200	-3.00612900
H	5.19827600	4.43473900	-0.25316100
H	0.77990800	4.07640000	-1.78307700
H	0.50939500	2.32310300	-1.75606500
H	-0.02686100	3.30149200	-0.40630900
H	4.68730700	0.73593200	2.56888400
H	2.93369500	0.48717200	2.67233700
C	-2.01936400	1.56336800	1.21257500
C	-2.91388200	0.79028700	0.57358700
C	-4.25183500	1.16852100	0.06464000
C	-4.95416300	0.24995300	-0.72261500
C	-4.84804500	2.40672800	0.33003000
C	-6.20842000	0.55693300	-1.23942700
H	-4.50280600	-0.71918900	-0.93008500
C	-6.10083800	2.71636000	-0.18314100
H	-4.32901400	3.13179700	0.95169600
C	-6.78614500	1.79366000	-0.97162000
H	-6.73584100	-0.17131900	-1.85016400
H	-6.54910900	3.68219000	0.03577900
H	-7.76710100	2.03790800	-1.37061200
H	-2.30939500	2.60233300	1.39661600
